# Supplementary material for: Drivers and assemblies of soil eukaryotic microbes among different soil habitat types in a semi-arid mountain in China
Source: PeerJ. 2018 Dec 5;6:e6042. doi: 10.7717/peerj.6042 (PMC6286657; doi:10.7717/peerj.6042)
Supplement: Supplemental Information 1 [file peerj-06-6042-s001.docx]

Table S1 More detailed information about the PCR steps.

| First PCR | | |  | Second PCR | | | | |
| --- | --- | --- | --- | --- | --- | --- | --- | --- |
| 5xBuffer | 10μL | |  | 5xBuffer | | 8μL | | |
| dNTP (10mM) | 1μL | |  | dNTP (10mM) | | 1μL | | |
| DNA polymerase | 1U | |  | DNA polymerase | | 0.8 U | | |
| F/R Inner primer(10 uM) | Each 1μL | |  | F/R Outer primer(10 uM) | | Each 1μL | | |
| Template | 5ng-50ng | |  | Template | | 5uL | | |
| ddH_2_O | fill up to 50 μL | |  | ddH_2_O | | fill up to e0 μL | | |
| First PCR system | | |  | Second PCR system | | | |  |
| PCR instrument(Applied Biosystems 9700 , USA) | | |  | PCR instrument(Applied Biosystems 9700 , USA) | | | | |
|  | 94℃ | 2min |  |  | 94℃ | | 2min | |
| 33 cycles | 94℃ | 30s |  | 8 cycles | 94℃ | | 30s | |
|  | 55℃ | 30s |  |  | 56℃ | | 30s | |
|  | 72℃ | 30s |  |  | 72℃ | | 30s | |
|  | 72℃ | 5min |  |  | 72℃ | | 5min | |
|  | 10℃ | heat preservation |  |  | 10℃ | | heat preservation | |

The electrophoresis (1.5% agarose gel in 0.5*TBE) and gel extraction kit (Axygen Biosciences, USA) can separate and purify the PCR products. F/R Inner primer: 5'-TTCCCTACACGACGCTCTTCCGATCT3';5'-GAGTTCCTTGGCACCCGAGAATTCCA3'. F/R Outer primer: 5'-AATGATACGGCGACCACCGAGATCTACACTCTTTCCCTACACGACGCTC -3';5'-CAAGCAGAAGACGGCATACGAGATGTGACTGGAGTTCCTTGGCACCCGAGA-3.
