# Supplementary material for: Drivers and assemblies of soil eukaryotic microbes among different soil habitat types in a semi-arid mountain in China
Source: PeerJ. 2018 Dec 5;6:e6042. doi: 10.7717/peerj.6042 (PMC6286657; doi:10.7717/peerj.6042)
Supplement: Supplemental Information 2 [file peerj-06-6042-s002.docx]

| Sample name | barcode1 | barcode2 |
| --- | --- | --- |
| Grass 1.fastq | ATGCATCC | TCAGACGG |
| Grass 2.fastq | ATGCATCC | TTGGAATG |
| Grass 3.fastq | ATGCATCC | CCCAGTCG |
| Forest 1_R1.fastq | ATGCATCC | AGAGTGTC |
| Forest 2_R1.fastq | ATGCATCC | TTCGCCGC |
| Forest 3_R1.fastq | ATGCATCC | GTGTCTTA |
| Farmland 1_R1.fastq | ATGCATCC | TAGGTGTT |
| Farmland 2.fastq | ATGCATCC | TTGACGTC |
| Farmland 3.fastq | ATGCATCC | GAAGACCA |
| Shrub 1.fastq | ATGCATCC | CTTAGAGT |
| Shrub 2.fastq | ATGCATCC | CATCAGAA |
| Shrub 3.fastq | ATGCATCC | CAGAAGCG |

Table S2. The barcodes in each Sample.
