# Supplementary material for: Drivers and assemblies of soil eukaryotic microbes among different soil habitat types in a semi-arid mountain in China
Source: PeerJ. 2018 Dec 5;6:e6042. doi: 10.7717/peerj.6042 (PMC6286657; doi:10.7717/peerj.6042)
Supplement: Supplemental Information 3 [file peerj-06-6042-s003.docx]

Table S3. The elemental analysis of O horizon soil.

| Sample | MgO (%) | SiO_2_ (%) | CaO (%) | Ti (PPM) | V (PPM) | Mn (PPM) | Fe_2_O_3_ (%) | Ni (PPM) | Cu (PPM) | Zn (PPM) | Pb (PPM) | U (PPM) |
| --- | --- | --- | --- | --- | --- | --- | --- | --- | --- | --- | --- | --- |
| forest1 | 2.33 | 57.66 | 3.59 | 3825.9 | 81.7 | 577 | 4.72 | 30.1 | 25.6 | 62.8 | 44.3 | 2.5 |
| forest2 | 2.31 | 60.74 | 3.91 | 3859.4 | 83.5 | 569 | 4.81 | 28.4 | 27.9 | 69.5 | 43.8 | 2.8 |
| forest3 | 2.44 | 59.5 | 3.95 | 3901.5 | 80 | 571 | 4.52 | 26.5 | 26.1 | 67.1 | 42.1 | 2.8 |
| farmland1 | 2.17 | 57.02 | 1.54 | 3681.1 | 75.7 | 576 | 4.7 | 26.7 | 20 | 61.1 | 23.3 | 0.7 |
| farmland2 | 2.13 | 57.28 | 1.73 | 3647.5 | 70.8 | 497 | 4.89 | 26.5 | 22.1 | 61.3 | 22.9 | 0.5 |
| farmland3 | 2.13 | 55.42 | 1.89 | 3695.5 | 73.8 | 535 | 4.83 | 24.9 | 19.5 | 53.2 | 24.4 | 0.4 |
| shrub1 | 2.21 | 58.46 | 2.08 | 3812.2 | 81.8 | 429 | 4.39 | 40.1 | 43.6 | 74.2 | 39.8 | 1.1 |
| shrub2 | 2.27 | 58.19 | 1.93 | 3845.4 | 80.8 | 468 | 4.51 | 39.1 | 45.2 | 76.9 | 39.2 | 1.2 |
| shrub3 | 2.13 | 58.34 | 2.02 | 3810.7 | 86.8 | 453 | 4.55 | 38.9 | 43.4 | 73.1 | 40.6 | 1.5 |
| grass1 | 2.57 | 57.9 | 2.86 | 4381.9 | 104.5 | 609 | 6.14 | 42.7 | 61.9 | 104.6 | 34.7 | 3.3 |
| grass2 | 2.53 | 58.82 | 2.81 | 4226.1 | 101.9 | 688 | 5.15 | 45.6 | 62.6 | 106.4 | 35.4 | 3.3 |
| grass3 | 2.52 | 62.81 | 2.97 | 4183.4 | 92.3 | 623 | 5.94 | 45.9 | 60.3 | 95.1 | 41.7 | 3.7 |

Note: Using PCA (principal component analysis) to compress the element data of term organic matter layer, the comp.1 and comp.2 axes (Cumulative Proportion ＞ 80%） are defined as TOM (term organic matter layer)1 and TOM2.

The results of PCA:

Comp.1 Comp.2

Standard deviation 2.7713530 1.4128615

Proportion of Variance 0.6400331 0.1663481

Cumulative Proportion 0.6400331 0.8063812

Comp.1 Comp.2

[1] 0.3952405 -1.8919874

[2] -0.3597412 -2.5095802

[3] -0.1011271 -2.5899028

[4] 3.0096398 0.9263883

[5] 3.4258977 0.9270077

[6] 3.6254524 0.8662455

[7] 1.1249281 0.4899736

[8] 0.7988064 0.6603870

[9] 0.9342136 0.5245014

[10] -4.4231814 1.6669533

[11] -.0478505 1.0726758

[12] -4.3822783 -0.1426623
