# Supplementary material for: Drivers and assemblies of soil eukaryotic microbes among different soil habitat types in a semi-arid mountain in China
Source: PeerJ. 2018 Dec 5;6:e6042. doi: 10.7717/peerj.6042 (PMC6286657; doi:10.7717/peerj.6042)
Supplement: Supplemental Information 4 [file peerj-06-6042-s004.doc]

Table S4 Results of indicator species analysis showing soil eukaryotic microbes characteristic (p.value < 0.05) in each habitat type.

| OTUID |  | forest | farmland | shrub | grass | index | stat | p.value |
| --- | --- | --- | --- | --- | --- | --- | --- | --- |
| OTU_361 | Rhizaria,Cercozoa | 0 | 0 | 1 | 0 | 3 | 0.8132 | 0.011 |
| OTU_255 | Alveolata,Ciliophora | 0 | 0 | 1 | 0 | 3 | 0.9798 | 0.014 |
| OTU_185 | Alveolata,Ciliophora | 0 | 0 | 1 | 0 | 3 | 0.7498 | 0.014 |
| OTU_670 | Alveolata,Ciliophora | 0 | 0 | 1 | 0 | 3 | 0.9747 | 0.014 |
| OTU_935 | Alveolata,other | 0 | 0 | 1 | 0 | 3 | 1.0000 | 0.014 |
| OTU_618 | Amoebozoa | 0 | 0 | 0 | 1 | 4 | 1.0000 | 0.014 |
| OTU_1061 | Amoebozoa | 0 | 0 | 0 | 1 | 4 | 0.9636 | 0.014 |
| OTU_365 | Amoebozoa | 0 | 0 | 1 | 0 | 3 | 0.8750 | 0.014 |
| OTU_870 | Amoebozoa | 0 | 0 | 1 | 0 | 3 | 0.9089 | 0.014 |
| OTU_926 | Amoebozoa | 0 | 0 | 1 | 0 | 3 | 0.9063 | 0.014 |
| OTU_740 | chlorophyta | 0 | 0 | 1 | 0 | 3 | 0.9806 | 0.014 |
| OTU_65 | Ascomycota | 0 | 0 | 1 | 0 | 3 | 0.9929 | 0.014 |
| OTU_155 | Basidiomycota | 0 | 0 | 0 | 1 | 4 | 1.0000 | 0.014 |
| OTU_118 | Basidiomycota | 0 | 0 | 0 | 1 | 4 | 0.9413 | 0.014 |
| OTU_62 | Basidiomycota | 0 | 0 | 0 | 1 | 4 | 0.9219 | 0.014 |
| OTU_499 | Basidiomycota | 0 | 0 | 1 | 0 | 3 | 1.0000 | 0.014 |
| OTU_280 | Basidiomycota | 0 | 0 | 1 | 0 | 3 | 0.9914 | 0.014 |
| OTU_133 | Chytridiomycota | 0 | 0 | 0 | 1 | 4 | 0.9810 | 0.014 |
| OTU_336 | Chytridiomycota | 0 | 0 | 0 | 1 | 4 | 1.0000 | 0.014 |
| OTU_1079 | Cryptomycota | 0 | 0 | 0 | 1 | 4 | 1.0000 | 0.014 |
| OTU_144 | Fungi,other | 0 | 0 | 1 | 0 | 3 | 1.0000 | 0.014 |
| OTU_778 | Fungi,other | 0 | 0 | 0 | 1 | 4 | 0.8898 | 0.014 |
| OTU_848 | Glomeromycota | 0 | 0 | 1 | 0 | 3 | 1.0000 | 0.014 |
| OTU_304 | Metazoa,Arthropoda | 0 | 0 | 1 | 0 | 3 | 1.0000 | 0.014 |
| OTU_399 | Metazoa,Arthropoda | 0 | 0 | 1 | 0 | 3 | 1.0000 | 0.014 |
| OTU_32 | Metazoa,Arthropoda | 0 | 0 | 0 | 1 | 4 | 1.0000 | 0.014 |
| OTU_95 | Metazoa,Arthropoda | 0 | 0 | 1 | 0 | 3 | 0.9962 | 0.014 |
| OTU_260 | Metazoa,Arthropoda | 0 | 0 | 1 | 0 | 3 | 1.0000 | 0.014 |
| OTU_352 | Metazoa,Arthropoda | 0 | 0 | 1 | 0 | 3 | 1.0000 | 0.014 |
| OTU_426 | Metazoa,Arthropoda | 0 | 0 | 0 | 1 | 4 | 1.0000 | 0.014 |
| OTU_25 | Metazoa,Arthropoda | 0 | 0 | 1 | 0 | 3 | 1.0000 | 0.014 |
| OTU_34 | Metazoa,Arthropoda | 0 | 0 | 1 | 0 | 3 | 1.0000 | 0.014 |
| OTU_442 | Metazoa,Arthropoda | 0 | 0 | 1 | 0 | 3 | 0.9661 | 0.014 |
| OTU_71 | Metazoa,Nematoda | 0 | 0 | 0 | 1 | 4 | 0.8980 | 0.014 |
| OTU_158 | Metazoa,Nematoda | 0 | 0 | 1 | 0 | 3 | 1.0000 | 0.014 |
| OTU_479 | Metazoa,Nematoda | 0 | 0 | 1 | 0 | 3 | 0.9701 | 0.014 |
| OTU_654 | Metazoa,Nematoda | 0 | 0 | 1 | 0 | 3 | 0.9199 | 0.014 |
| OTU_898 | Metazoa,Nematoda | 0 | 0 | 0 | 1 | 4 | 0.9843 | 0.014 |
| OTU_17 | Metazoa,Nematoda | 0 | 0 | 1 | 0 | 3 | 0.9984 | 0.014 |
| OTU_583 | Metazoa,Nematoda | 0 | 0 | 1 | 0 | 3 | 1.0000 | 0.014 |
| OTU_110 | Metazoa,other | 0 | 0 | 1 | 0 | 3 | 0.9071 | 0.014 |
| OTU_53 | Metazoa,other | 0 | 0 | 1 | 0 | 3 | 0.9988 | 0.014 |
| OTU_148 | Metazoa,other | 0 | 0 | 1 | 0 | 3 | 0.9317 | 0.014 |
| OTU_817 | Metazoa,other | 0 | 0 | 1 | 0 | 3 | 1.0000 | 0.014 |
| OTU_977 | Metazoa,other | 0 | 0 | 1 | 0 | 3 | 1.0000 | 0.014 |
| OTU_745 | Metazoa,other | 0 | 0 | 1 | 0 | 3 | 1.0000 | 0.014 |
| OTU_541 | Metazoa,other | 0 | 0 | 1 | 0 | 3 | 1.0000 | 0.014 |
| OTU_189 | Rhizaria,Cercozoa | 0 | 0 | 1 | 0 | 3 | 0.8885 | 0.014 |
| OTU_966 | Rhizaria,Cercozoa | 0 | 0 | 1 | 0 | 3 | 0.9682 | 0.014 |
| OTU_340 | Rhizaria,other | 0 | 0 | 1 | 0 | 3 | 0.8895 | 0.014 |
| OTU_342 | Rhizaria,other | 0 | 0 | 0 | 1 | 4 | 0.8246 | 0.014 |
| OTU_815 | Rhizaria,other | 0 | 0 | 0 | 1 | 4 | 1.0000 | 0.014 |
| OTU_511 | Rhizaria,other | 0 | 0 | 1 | 0 | 3 | 0.9129 | 0.014 |
| OTU_710 | Rhizaria,other | 0 | 0 | 0 | 1 | 4 | 1.0000 | 0.014 |
| OTU_52 | Rhizaria,other | 0 | 0 | 1 | 0 | 3 | 0.9885 | 0.014 |
| OTU_202 | Rhizaria,other | 0 | 0 | 1 | 0 | 3 | 0.9899 | 0.014 |
| OTU_318 | Rhizaria,other | 0 | 0 | 0 | 1 | 4 | 0.9449 | 0.014 |
| OTU_473 | Rhizaria,other | 0 | 0 | 0 | 1 | 4 | 0.9586 | 0.014 |
| OTU_936 | Rhizaria,other | 0 | 0 | 1 | 0 | 3 | 0.9512 | 0.014 |
| OTU_124 | Rhizaria,other | 0 | 0 | 1 | 0 | 3 | 0.9184 | 0.014 |
| OTU_248 | Rhizaria,other | 0 | 0 | 0 | 1 | 4 | 0.9824 | 0.014 |
| OTU_28 | Rhizaria,other | 0 | 0 | 1 | 0 | 3 | 0.9892 | 0.014 |
| OTU_92 | Rhizaria,other | 0 | 0 | 0 | 1 | 4 | 0.8429 | 0.014 |
| OTU_294 | Rhizaria,other | 0 | 0 | 1 | 0 | 3 | 0.9858 | 0.014 |
| OTU_982 | Rhizaria,other | 0 | 0 | 0 | 1 | 4 | 0.9186 | 0.014 |
| OTU_694 | Rhizaria,other | 0 | 0 | 0 | 1 | 4 | 0.9860 | 0.014 |
| OTU_192 | Rhizaria,other | 0 | 0 | 0 | 1 | 4 | 0.7488 | 0.014 |
| OTU_217 | Stramenopiles | 0 | 0 | 0 | 1 | 4 | 0.9757 | 0.014 |
| OTU_452 | Stramenopiles | 0 | 0 | 0 | 1 | 4 | 1.0000 | 0.014 |
| OTU_514 | Stramenopiles | 0 | 0 | 1 | 0 | 3 | 0.7400 | 0.014 |
| OTU_960 | Stramenopiles | 0 | 0 | 0 | 1 | 4 | 0.9837 | 0.014 |
| OTU_45 | Stramenopiles | 0 | 0 | 0 | 1 | 4 | 0.8318 | 0.014 |
| OTU_353 | unclassified | 0 | 0 | 1 | 0 | 3 | 0.9710 | 0.014 |
| OTU_230 | unclassified | 0 | 0 | 0 | 1 | 4 | 0.8439 | 0.014 |
| OTU_20 | unclassified | 0 | 0 | 0 | 1 | 4 | 0.9991 | 0.014 |
| OTU_212 | unclassified | 0 | 0 | 1 | 0 | 3 | 1.0000 | 0.014 |
| OTU_226 | unclassified | 0 | 0 | 0 | 1 | 4 | 0.8275 | 0.014 |
| OTU_284 | unclassified | 0 | 0 | 1 | 0 | 3 | 1.0000 | 0.014 |
| OTU_345 | unclassified | 0 | 0 | 0 | 1 | 4 | 1.0000 | 0.014 |
| OTU_360 | unclassified | 0 | 0 | 0 | 1 | 4 | 1.0000 | 0.014 |
| OTU_435 | unclassified | 0 | 0 | 1 | 0 | 3 | 0.9220 | 0.014 |
| OTU_492 | unclassified | 0 | 0 | 1 | 0 | 3 | 0.9649 | 0.014 |
| OTU_585 | unclassified | 0 | 0 | 1 | 0 | 3 | 0.8959 | 0.014 |
| OTU_703 | unclassified | 0 | 0 | 1 | 0 | 3 | 1.0000 | 0.014 |
| OTU_810 | unclassified | 0 | 0 | 0 | 1 | 4 | 0.9701 | 0.014 |
| OTU_924 | unclassified | 0 | 0 | 1 | 0 | 3 | 1.0000 | 0.014 |
| OTU_74 | Alveolata,Apicomplexa | 1 | 0 | 0 | 0 | 1 | 1.0000 | 0.017 |
| OTU_240 | Alveolata,Apicomplexa | 1 | 0 | 0 | 0 | 1 | 0.9949 | 0.017 |
| OTU_299 | Alveolata,Ciliophora | 1 | 0 | 0 | 0 | 1 | 0.8452 | 0.017 |
| OTU_826 | Alveolata,Ciliophora | 1 | 0 | 0 | 0 | 1 | 0.9393 | 0.017 |
| OTU_706 | Alveolata,Dinophyceae | 1 | 0 | 0 | 0 | 1 | 1.0000 | 0.017 |
| OTU_417 | Amoebozoa | 1 | 0 | 0 | 0 | 1 | 0.8473 | 0.017 |
| OTU_764 | Amoebozoa | 1 | 0 | 0 | 0 | 1 | 0.8272 | 0.017 |
| OTU_374 | Amoebozoa | 1 | 0 | 0 | 0 | 1 | 0.8793 | 0.017 |
| OTU_831 | Amoebozoa | 1 | 0 | 0 | 0 | 1 | 0.9177 | 0.017 |
| OTU_481 | Amoebozoa | 1 | 0 | 0 | 0 | 1 | 0.7547 | 0.017 |
| OTU_43 | Amoebozoa | 1 | 0 | 0 | 0 | 1 | 0.7578 | 0.017 |
| OTU_909 | Amoebozoa | 1 | 0 | 0 | 0 | 1 | 0.9780 | 0.017 |
| OTU_42 | chlorophyta | 1 | 0 | 0 | 0 | 1 | 0.9965 | 0.017 |
| OTU_159 | chlorophyta | 1 | 0 | 0 | 0 | 1 | 0.8244 | 0.017 |
| OTU_276 | chlorophyta | 1 | 0 | 0 | 0 | 1 | 0.9606 | 0.017 |
| OTU_672 | chlorophyta | 1 | 0 | 0 | 0 | 1 | 1.0000 | 0.017 |
| OTU_130 | chlorophyta | 1 | 0 | 0 | 0 | 1 | 0.9592 | 0.017 |
| OTU_216 | chlorophyta | 1 | 0 | 0 | 0 | 1 | 1.0000 | 0.017 |
| OTU_241 | Ascomycota | 1 | 0 | 0 | 0 | 1 | 1.0000 | 0.017 |
| OTU_259 | Basidiomycota | 1 | 0 | 0 | 0 | 1 | 1.0000 | 0.017 |
| OTU_107 | Basidiomycota | 1 | 0 | 0 | 0 | 1 | 0.9962 | 0.017 |
| OTU_821 | Basidiomycota | 1 | 0 | 0 | 0 | 1 | 0.9535 | 0.017 |
| OTU_972 | Chytridiomycota | 1 | 0 | 0 | 0 | 1 | 1.0000 | 0.017 |
| OTU_387 | Cryptomycota | 1 | 0 | 0 | 0 | 1 | 0.9428 | 0.017 |
| OTU_576 | Cryptomycota | 1 | 0 | 0 | 0 | 1 | 0.9333 | 0.017 |
| OTU_736 | Fungi,other | 1 | 0 | 0 | 0 | 1 | 0.9785 | 0.017 |
| OTU_125 | Fungi,other | 1 | 0 | 0 | 0 | 1 | 0.9301 | 0.017 |
| OTU_650 | Fungi,other | 1 | 0 | 0 | 0 | 1 | 1.0000 | 0.017 |
| OTU_518 | Fungi,other | 1 | 0 | 0 | 0 | 1 | 1.0000 | 0.017 |
| OTU_201 | Glomeromycota | 1 | 0 | 0 | 0 | 1 | 0.8586 | 0.017 |
| OTU_330 | Glomeromycota | 1 | 0 | 0 | 0 | 1 | 0.9619 | 0.017 |
| OTU_444 | Glomeromycota | 1 | 0 | 0 | 0 | 1 | 0.7231 | 0.017 |
| OTU_534 | Glomeromycota | 1 | 0 | 0 | 0 | 1 | 1.0000 | 0.017 |
| OTU_356 | Metazoa,Arthropoda | 1 | 0 | 0 | 0 | 1 | 1.0000 | 0.017 |
| OTU_566 | Metazoa,Arthropoda | 1 | 0 | 0 | 0 | 1 | 1.0000 | 0.017 |
| OTU_295 | Metazoa,Arthropoda | 1 | 0 | 0 | 0 | 1 | 0.9910 | 0.017 |
| OTU_24 | Metazoa,Arthropoda | 1 | 0 | 0 | 0 | 1 | 0.9995 | 0.017 |
| OTU_844 | Metazoa,Arthropoda | 1 | 0 | 0 | 0 | 1 | 1.0000 | 0.017 |
| OTU_41 | Metazoa,Arthropoda | 1 | 0 | 0 | 0 | 1 | 0.9914 | 0.017 |
| OTU_12 | Metazoa,Nematoda | 1 | 0 | 0 | 0 | 1 | 0.8140 | 0.017 |
| OTU_372 | Rhizaria,Cercozoa | 1 | 0 | 0 | 0 | 1 | 0.7352 | 0.017 |
| OTU_633 | Rhizaria,Cercozoa | 1 | 0 | 0 | 0 | 1 | 0.9780 | 0.017 |
| OTU_184 | Rhizaria,Cercozoa | 1 | 0 | 0 | 0 | 1 | 0.8742 | 0.017 |
| OTU_328 | Rhizaria,Cercozoa | 1 | 0 | 0 | 0 | 1 | 0.9211 | 0.017 |
| OTU_866 | Rhizaria,Cercozoa | 1 | 0 | 0 | 0 | 1 | 0.8452 | 0.017 |
| OTU_897 | Rhizaria,Cercozoa | 1 | 0 | 0 | 0 | 1 | 0.9500 | 0.017 |
| OTU_950 | Rhizaria,Cercozoa | 1 | 0 | 0 | 0 | 1 | 0.9258 | 0.017 |
| OTU_1095 | Rhizaria,Cercozoa | 1 | 0 | 0 | 0 | 1 | 0.8062 | 0.017 |
| OTU_467 | Rhizaria,Cercozoa | 1 | 0 | 0 | 0 | 1 | 1.0000 | 0.017 |
| OTU_363 | Rhizaria,other | 1 | 0 | 0 | 0 | 1 | 0.8882 | 0.017 |
| OTU_391 | Rhizaria,other | 1 | 0 | 0 | 0 | 1 | 0.9315 | 0.017 |
| OTU_91 | Rhizaria,other | 1 | 0 | 0 | 0 | 1 | 0.8812 | 0.017 |
| OTU_829 | Rhizaria,other | 1 | 0 | 0 | 0 | 1 | 0.9428 | 0.017 |
| OTU_287 | Rhizaria,other | 1 | 0 | 0 | 0 | 1 | 0.6737 | 0.017 |
| OTU_302 | Rhizaria,other | 1 | 0 | 0 | 0 | 1 | 0.7153 | 0.017 |
| OTU_457 | Rhizaria,other | 1 | 0 | 0 | 0 | 1 | 1.0000 | 0.017 |
| OTU_551 | Rhizaria,other | 1 | 0 | 0 | 0 | 1 | 0.8997 | 0.017 |
| OTU_896 | Rhizaria,other | 1 | 0 | 0 | 0 | 1 | 0.9129 | 0.017 |
| OTU_990 | Rhizaria,other | 1 | 0 | 0 | 0 | 1 | 0.8980 | 0.017 |
| OTU_1002 | Rhizaria,other | 1 | 0 | 0 | 0 | 1 | 1.0000 | 0.017 |
| OTU_837 | Rhizaria,other | 1 | 0 | 0 | 0 | 1 | 0.8771 | 0.017 |
| OTU_1063 | Rhizaria,other | 1 | 0 | 0 | 0 | 1 | 1.0000 | 0.017 |
| OTU_72 | Rhizaria,other | 1 | 0 | 0 | 0 | 1 | 0.6860 | 0.017 |
| OTU_293 | Rhizaria,other | 1 | 0 | 0 | 0 | 1 | 0.9798 | 0.017 |
| OTU_87 | Rhizaria,other | 1 | 0 | 0 | 0 | 1 | 0.7692 | 0.017 |
| OTU_327 | Stramenopiles | 1 | 0 | 0 | 0 | 1 | 0.7157 | 0.017 |
| OTU_405 | Stramenopiles | 1 | 0 | 0 | 0 | 1 | 0.8803 | 0.017 |
| OTU_262 | Stramenopiles | 1 | 0 | 0 | 0 | 1 | 0.8660 | 0.017 |
| OTU_313 | Stramenopiles | 1 | 0 | 0 | 0 | 1 | 0.9014 | 0.017 |
| OTU_1090 | Stramenopiles | 1 | 0 | 0 | 0 | 1 | 1.0000 | 0.017 |
| OTU_1052 | Stramenopiles | 1 | 0 | 0 | 0 | 1 | 0.9847 | 0.017 |
| OTU_48 | Stramenopiles | 1 | 0 | 0 | 0 | 1 | 0.6886 | 0.017 |
| OTU_412 | unclassified | 1 | 0 | 0 | 0 | 1 | 0.8689 | 0.017 |
| OTU_9 | unclassified | 1 | 0 | 0 | 0 | 1 | 1.0000 | 0.017 |
| OTU_307 | unclassified | 1 | 0 | 0 | 0 | 1 | 0.9209 | 0.017 |
| OTU_379 | unclassified | 1 | 0 | 0 | 0 | 1 | 0.9478 | 0.017 |
| OTU_433 | unclassified | 1 | 0 | 0 | 0 | 1 | 0.9871 | 0.017 |
| OTU_450 | unclassified | 1 | 0 | 0 | 0 | 1 | 1.0000 | 0.017 |
| OTU_472 | unclassified | 1 | 0 | 0 | 0 | 1 | 0.8660 | 0.017 |
| OTU_577 | unclassified | 1 | 0 | 0 | 0 | 1 | 0.9770 | 0.017 |
| OTU_601 | unclassified | 1 | 0 | 0 | 0 | 1 | 0.8745 | 0.017 |
| OTU_700 | unclassified | 1 | 0 | 0 | 0 | 1 | 0.9718 | 0.017 |
| OTU_858 | unclassified | 1 | 0 | 0 | 0 | 1 | 0.9177 | 0.017 |
| OTU_884 | unclassified | 1 | 0 | 0 | 0 | 1 | 1.0000 | 0.017 |
| OTU_197 | Basidiomycota | 0 | 0 | 1 | 0 | 3 | 0.7984 | 0.019 |
| OTU_834 | Stramenopiles | 0 | 1 | 0 | 0 | 2 | 0.8035 | 0.021 |
| OTU_136 | Alveolata,Apicomplexa | 0 | 1 | 0 | 0 | 2 | 1.0000 | 0.022 |
| OTU_164 | Amoebozoa | 0 | 1 | 0 | 0 | 2 | 1.0000 | 0.022 |
| OTU_227 | Amoebozoa | 0 | 1 | 0 | 0 | 2 | 0.9338 | 0.022 |
| OTU_914 | Amoebozoa | 0 | 1 | 0 | 0 | 2 | 1.0000 | 0.022 |
| OTU_232 | chlorophyta | 0 | 1 | 0 | 0 | 2 | 0.8434 | 0.022 |
| OTU_906 | chlorophyta | 0 | 1 | 0 | 0 | 2 | 0.8528 | 0.022 |
| OTU_562 | Ascomycota | 0 | 1 | 0 | 0 | 2 | 1.0000 | 0.022 |
| OTU_505 | Ascomycota | 0 | 1 | 0 | 0 | 2 | 1.0000 | 0.022 |
| OTU_920 | Chytridiomycota | 0 | 1 | 0 | 0 | 2 | 0.9405 | 0.022 |
| OTU_61 | Metazoa,Arthropoda | 0 | 1 | 0 | 0 | 2 | 1.0000 | 0.022 |
| OTU_31 | Metazoa,Arthropoda | 0 | 1 | 0 | 0 | 2 | 0.8508 | 0.022 |
| OTU_167 | Metazoa,Arthropoda | 0 | 1 | 0 | 0 | 2 | 0.9806 | 0.022 |
| OTU_128 | Metazoa,Arthropoda | 0 | 1 | 0 | 0 | 2 | 0.9424 | 0.022 |
| OTU_634 | Metazoa,Arthropoda | 0 | 1 | 0 | 0 | 2 | 1.0000 | 0.022 |
| OTU_98 | Metazoa,Nematoda | 0 | 1 | 0 | 0 | 2 | 1.0000 | 0.022 |
| OTU_160 | Metazoa,Nematoda | 0 | 1 | 0 | 0 | 2 | 1.0000 | 0.022 |
| OTU_193 | Metazoa,Nematoda | 0 | 1 | 0 | 0 | 2 | 0.9836 | 0.022 |
| OTU_763 | Metazoa,Nematoda | 0 | 1 | 0 | 0 | 2 | 1.0000 | 0.022 |
| OTU_181 | Metazoa,Nematoda | 0 | 1 | 0 | 0 | 2 | 0.9927 | 0.022 |
| OTU_242 | Metazoa,Nematoda | 0 | 1 | 0 | 0 | 2 | 0.9794 | 0.022 |
| OTU_213 | Metazoa,Nematoda | 0 | 1 | 0 | 0 | 2 | 1.0000 | 0.022 |
| OTU_44 | Metazoa,Nematoda | 0 | 1 | 0 | 0 | 2 | 1.0000 | 0.022 |
| OTU_292 | Metazoa,Nematoda | 0 | 1 | 0 | 0 | 2 | 0.9726 | 0.022 |
| OTU_822 | Metazoa,Nematoda | 0 | 1 | 0 | 0 | 2 | 1.0000 | 0.022 |
| OTU_873 | Metazoa,Nematoda | 0 | 1 | 0 | 0 | 2 | 1.0000 | 0.022 |
| OTU_129 | Metazoa,other | 0 | 1 | 0 | 0 | 2 | 1.0000 | 0.022 |
| OTU_820 | Rhizaria,other | 0 | 1 | 0 | 0 | 2 | 1.0000 | 0.022 |
| OTU_768 | Rhizaria,other | 0 | 1 | 0 | 0 | 2 | 0.9089 | 0.022 |
| OTU_861 | Stramenopiles | 0 | 1 | 0 | 0 | 2 | 0.9129 | 0.022 |
| OTU_984 | Stramenopiles | 0 | 1 | 0 | 0 | 2 | 1.0000 | 0.022 |
| OTU_370 | Stramenopiles | 0 | 1 | 0 | 0 | 2 | 0.7591 | 0.022 |
| OTU_251 | Stramenopiles | 0 | 1 | 0 | 0 | 2 | 0.9459 | 0.022 |
| OTU_546 | unclassified | 0 | 1 | 0 | 0 | 2 | 0.9661 | 0.022 |
| OTU_872 | unclassified | 0 | 1 | 0 | 0 | 2 | 1.0000 | 0.022 |
| OTU_958 | unclassified | 0 | 1 | 0 | 0 | 2 | 0.9520 | 0.022 |
| OTU_900 | Rhizaria,other | 0 | 0 | 0 | 1 | 4 | 0.9672 | 0.023 |
| OTU_704 | unclassified | 0 | 0 | 1 | 0 | 3 | 0.8819 | 0.026 |
| OTU_385 | Fungi,other | 1 | 0 | 0 | 0 | 1 | 0.9813 | 0.028 |
| OTU_448 | Rhizaria,Cercozoa | 1 | 0 | 0 | 0 | 1 | 0.9089 | 0.028 |
| OTU_66 | Rhizaria,other | 1 | 0 | 0 | 0 | 1 | 0.6806 | 0.028 |
| OTU_462 | Rhizaria,other | 0 | 0 | 1 | 0 | 3 | 0.8452 | 0.029 |
| OTU_149 | Metazoa,Nematoda | 0 | 0 | 1 | 0 | 3 | 0.9791 | 0.03 |
| OTU_539 | Amoebozoa | 1 | 0 | 0 | 0 | 1 | 0.8987 | 0.032 |
| OTU_29 | Metazoa,Nematoda | 1 | 0 | 0 | 0 | 1 | 0.9782 | 0.032 |
| OTU_137 | Rhizaria,Cercozoa | 1 | 0 | 0 | 0 | 1 | 0.8689 | 0.032 |
| OTU_249 | Rhizaria,other | 0 | 0 | 0 | 1 | 4 | 0.9290 | 0.032 |
| OTU_502 | unclassified | 0 | 0 | 0 | 1 | 4 | 0.8898 | 0.032 |
| OTU_285 | Amoebozoa | 0 | 0 | 1 | 0 | 3 | 0.9233 | 0.033 |
| OTU_421 | Amoebozoa | 0 | 1 | 0 | 0 | 2 | 0.9045 | 0.033 |
| OTU_656 | Glomeromycota | 1 | 0 | 0 | 0 | 1 | 0.9411 | 0.033 |
| OTU_801 | Rhizaria,Cercozoa | 1 | 0 | 0 | 0 | 1 | 0.8619 | 0.033 |
| OTU_865 | Rhizaria,other | 0 | 1 | 0 | 0 | 2 | 0.9258 | 0.033 |
| OTU_58 | unclassified | 0 | 0 | 1 | 0 | 3 | 0.8040 | 0.033 |
| OTU_572 | Alveolata,Ciliophora | 0 | 0 | 0 | 1 | 4 | 0.9075 | 0.034 |
| OTU_308 | Alveolata,Ciliophora | 1 | 0 | 0 | 0 | 1 | 0.9428 | 0.034 |
| OTU_347 | Chytridiomycota | 1 | 0 | 0 | 0 | 1 | 0.9852 | 0.034 |
| OTU_67 | Metazoa,Nematoda | 0 | 0 | 1 | 0 | 3 | 0.9930 | 0.034 |
| OTU_622 | Rhizaria,other | 0 | 1 | 0 | 0 | 2 | 0.9636 | 0.034 |
| OTU_524 | Rhizaria,other | 1 | 0 | 0 | 0 | 1 | 0.9158 | 0.034 |
| OTU_606 | Stramenopiles | 1 | 0 | 0 | 0 | 1 | 0.9177 | 0.034 |
| OTU_214 | Amoebozoa | 1 | 0 | 0 | 0 | 1 | 0.7507 | 0.035 |
| OTU_432 | Metazoa,Nematoda | 1 | 0 | 0 | 0 | 1 | 0.8978 | 0.035 |
| OTU_930 | Amoebozoa | 1 | 0 | 0 | 0 | 1 | 0.9309 | 0.036 |
| OTU_170 | Ascomycota | 0 | 0 | 1 | 0 | 3 | 0.7773 | 0.036 |
| OTU_497 | Basidiomycota | 0 | 0 | 0 | 1 | 4 | 0.8692 | 0.036 |
| OTU_803 | Fungi,other | 0 | 0 | 1 | 0 | 3 | 0.9415 | 0.036 |
| OTU_218 | Metazoa,Arthropoda | 0 | 0 | 1 | 0 | 3 | 0.9765 | 0.036 |
| OTU_221 | Metazoa,Arthropoda | 0 | 0 | 1 | 0 | 3 | 0.9030 | 0.036 |
| OTU_513 | Metazoa,Arthropoda | 0 | 1 | 0 | 0 | 2 | 0.9022 | 0.036 |
| OTU_759 | Metazoa,other | 0 | 0 | 0 | 1 | 4 | 0.8944 | 0.036 |
| OTU_491 | Rhizaria,Cercozoa | 0 | 0 | 1 | 0 | 3 | 0.9220 | 0.036 |
| OTU_371 | Rhizaria,Cercozoa | 1 | 0 | 0 | 0 | 1 | 0.8597 | 0.036 |
| OTU_73 | Rhizaria,Cercozoa | 0 | 0 | 1 | 0 | 3 | 0.7215 | 0.036 |
| OTU_142 | Rhizaria,other | 1 | 0 | 0 | 0 | 1 | 0.8720 | 0.036 |
| OTU_178 | Rhizaria,other | 1 | 0 | 0 | 0 | 1 | 0.8980 | 0.036 |
| OTU_651 | Stramenopiles | 0 | 0 | 0 | 1 | 4 | 0.9177 | 0.036 |
| OTU_646 | Alveolata,Ciliophora | 0 | 0 | 0 | 1 | 4 | 0.6755 | 0.038 |
| OTU_162 | chlorophyta | 0 | 1 | 0 | 0 | 2 | 0.7481 | 0.038 |
| OTU_396 | Chytridiomycota | 0 | 1 | 0 | 0 | 2 | 0.8416 | 0.038 |
| OTU_69 | Metazoa,other | 0 | 0 | 1 | 0 | 3 | 0.9819 | 0.038 |
| OTU_833 | Stramenopiles | 0 | 0 | 0 | 1 | 4 | 0.9258 | 0.038 |
| OTU_470 | Stramenopiles | 0 | 0 | 1 | 0 | 3 | 0.8030 | 0.038 |
| OTU_324 | chlorophyta | 1 | 0 | 0 | 0 | 1 | 0.8878 | 0.039 |
| OTU_274 | Metazoa,other | 1 | 0 | 0 | 0 | 1 | 0.8740 | 0.039 |
| OTU_581 | unclassified | 1 | 0 | 0 | 0 | 1 | 0.7817 | 0.039 |
| OTU_952 | Glomeromycota | 0 | 0 | 1 | 0 | 3 | 0.9852 | 0.04 |
| OTU_94 | Alveolata,Ciliophora | 0 | 1 | 0 | 0 | 2 | 0.9480 | 0.041 |
| OTU_1046 | chlorophyta | 1 | 0 | 0 | 0 | 1 | 0.8485 | 0.041 |
| OTU_713 | Ascomycota | 1 | 0 | 0 | 0 | 1 | 0.8165 | 0.041 |
| OTU_410 | Rhizaria,other | 0 | 0 | 0 | 1 | 4 | 0.8452 | 0.041 |
| OTU_722 | unclassified | 0 | 0 | 0 | 1 | 4 | 0.7601 | 0.041 |
| OTU_398 | Amoebozoa | 0 | 0 | 1 | 0 | 3 | 0.9374 | 0.042 |
| OTU_1072 | Basidiomycota | 0 | 1 | 0 | 0 | 2 | 0.7947 | 0.042 |
| OTU_267 | Alveolata,Ciliophora | 0 | 0 | 1 | 0 | 3 | 0.6837 | 0.043 |
| OTU_747 | Rhizaria,other | 0 | 0 | 1 | 0 | 3 | 0.8597 | 0.043 |
| OTU_621 | Rhizaria,other | 1 | 0 | 0 | 0 | 1 | 0.7564 | 0.043 |
| OTU_770 | Rhizaria,other | 0 | 1 | 0 | 0 | 2 | 0.9177 | 0.043 |
| OTU_840 | Stramenopiles | 1 | 0 | 0 | 0 | 1 | 0.9045 | 0.043 |
| OTU_268 | Metazoa,Arthropoda | 1 | 0 | 0 | 0 | 1 | 0.8533 | 0.045 |
| OTU_250 | Metazoa,Nematoda | 0 | 1 | 0 | 0 | 2 | 0.8510 | 0.046 |
| OTU_586 | unclassified | 0 | 0 | 0 | 1 | 4 | 0.8710 | 0.046 |
| OTU_243 | unclassified | 0 | 1 | 0 | 0 | 2 | 0.7966 | 0.047 |
| OTU_90 | Basidiomycota | 0 | 0 | 0 | 1 | 4 | 0.8884 | 0.048 |
| OTU_951 | unclassified | 0 | 0 | 0 | 1 | 4 | 0.9129 | 0.048 |
| OTU_3 | Alveolata,other | 0 | 0 | 1 | 0 | 3 | 0.7493 | 0.049 |
| OTU_275 | Amoebozoa | 0 | 1 | 0 | 0 | 2 | 0.8341 | 0.049 |
| OTU_519 | Stramenopiles | 0 | 0 | 1 | 0 | 3 | 0.8718 | 0.049 |
| OTU_855 | unclassified | 0 | 1 | 0 | 0 | 2 | 0.8660 | 0.049 |
| OTU_579 | Rhizaria,Cercozoa | 1 | 0 | 0 | 0 | 1 | 0.9574 | 0.05 |
| OTU_959 | Rhizaria,other | 0 | 0 | 0 | 1 | 4 | 0.8885 | 0.05 |

Note: 1 represent the indicator species of this group.
