# Supplementary material for: Drivers and assemblies of soil eukaryotic microbes among different soil habitat types in a semi-arid mountain in China
Source: PeerJ. 2018 Dec 5;6:e6042. doi: 10.7717/peerj.6042 (PMC6286657; doi:10.7717/peerj.6042)
Supplement: Supplemental Information 5 [file peerj-06-6042-s005.docx]

Table S5. The information about all OTUs in our soil samples.

| OTUId | F-1 | F-2 | F-3 | FL_1 | FL_2 | FL_3 | S_1 | S_2 | S_3 | G_1 | G_2 | G_3 | Taxonomy |
| --- | --- | --- | --- | --- | --- | --- | --- | --- | --- | --- | --- | --- | --- |
| OTU_74 | 190 | 65 | 130 | 0 | 0 | 0 | 0 | 0 | 0 | 0 | 0 | 0 | All_GROUP:sk__Eukaryota(100);sknr0__SAR(98);sknr1__Alveolata(98);p__Apicomplexa(84); |
| OTU_979 | 0 | 0 | 0 | 0 | 14 | 0 | 0 | 0 | 0 | 0 | 0 | 0 | All_GROUP:sk__Eukaryota(100);sknr0__SAR(91);sknr1__Alveolata(91);p__Apicomplexa(89); |
| OTU_298 | 0 | 27 | 0 | 4 | 7 | 0 | 0 | 0 | 0 | 0 | 0 | 0 | All_GROUP:sk__Eukaryota(100);sknr0__SAR(100);sknr1__Alveolata(100);p__Apicomplexa(100);pnr0__Conoidasida(100);c__Gregarinasina(100);o__Eugregarinorida(100);g__Leidyana(93);s__Leidyana_erratica(93); |
| OTU_529 | 0 | 0 | 0 | 0 | 0 | 0 | 0 | 0 | 14 | 0 | 0 | 0 | All_GROUP:sk__Eukaryota(100);sknr0__SAR(100);sknr1__Alveolata(100);p__Apicomplexa(100);pnr0__Conoidasida(100);c__Gregarinasina(100);o__Eugregarinorida(100);g__Leidyana(95);s__Leidyana_erratica(95); |
| OTU_451 | 0 | 0 | 75 | 11 | 15 | 0 | 0 | 0 | 0 | 0 | 0 | 0 | All_GROUP:sk__Eukaryota(100);sknr0__SAR(100);sknr1__Alveolata(100);p__Apicomplexa(100);pnr0__Conoidasida(100);c__Gregarinasina(100);o__Eugregarinorida(100);g__Stenophora(100);s__Stenophora_robusta(100); |
| OTU_136 | 0 | 0 | 0 | 24 | 30 | 27 | 0 | 0 | 0 | 0 | 0 | 0 | All_GROUP:sk__Eukaryota(100);sknr0__SAR(100);sknr1__Alveolata(100);p__Apicomplexa(100);pnr0__Conoidasida(100);c__Gregarinasina(100);o__Eugregarinorida(100);g__Gregarina(90); |
| OTU_57 | 0 | 113 | 63 | 27 | 12 | 4 | 136 | 0 | 70 | 0 | 0 | 0 | All_GROUP:sk__Eukaryota(100);sknr0__SAR(97);sknr1__Alveolata(96);p__Apicomplexa(96);pnr0__Conoidasida(95);c__Gregarinasina(95);o__Eugregarinorida(95); |
| OTU_116 | 14 | 14 | 0 | 0 | 6 | 1 | 0 | 74 | 40 | 0 | 0 | 0 | All_GROUP:sk__Eukaryota(100);sknr0__SAR(99);sknr1__Alveolata(99);p__Apicomplexa(99);pnr0__Conoidasida(99);c__Gregarinasina(99);o__Eugregarinorida(99); |
| OTU_344 | 0 | 9 | 0 | 0 | 0 | 0 | 80 | 0 | 0 | 0 | 0 | 0 | All_GROUP:sk__Eukaryota(100);sknr0__SAR(99);sknr1__Alveolata(98);p__Apicomplexa(98);pnr0__Conoidasida(98);c__Gregarinasina(96);o__Eugregarinorida(94); |
| OTU_578 | 0 | 0 | 11 | 0 | 3 | 1 | 4 | 0 | 15 | 0 | 0 | 0 | All_GROUP:sk__Eukaryota(100);sknr0__SAR(98);sknr1__Alveolata(97);p__Apicomplexa(97);pnr0__Conoidasida(97);c__Gregarinasina(96);o__Eugregarinorida(96); |
| OTU_240 | 22 | 7 | 68 | 0 | 0 | 0 | 0 | 0 | 0 | 0 | 0 | 1 | All_GROUP:sk__Eukaryota(100);sknr0__SAR(97);sknr1__Alveolata(97);p__Apicomplexa(90);pnr0__Conoidasida(89); |
| OTU_358 | 0 | 0 | 0 | 0 | 0 | 0 | 0 | 15 | 0 | 0 | 0 | 56 | All_GROUP:sk__Eukaryota(100);sknr0__SAR(90);sknr1__Alveolata(87);p__Apicomplexa(86);pnr0__Conoidasida(80); |
| OTU_1017 | 0 | 0 | 0 | 0 | 0 | 0 | 8 | 0 | 0 | 0 | 0 | 0 | All_GROUP:sk__Eukaryota(100);sknr0__SAR(100);sknr1__Alveolata(100);p__Apicomplexa(100);pnr0__Conoidasida(100);c__Coccidia(100);subo__Adeleorina(100);g__Adelina(100);s__Adelina_grylli(100); |
| OTU_121 | 109 | 53 | 24 | 3 | 6 | 1 | 39 | 38 | 27 | 48 | 7 | 215 | All_GROUP:sk__Eukaryota(100);sknr0__SAR(100);sknr1__Alveolata(100);sknr2__Ciliophora(100);subp__Intramacronucleata(100);c__Spirotrichea(100); |
| OTU_143 | 19 | 17 | 24 | 0 | 27 | 3 | 0 | 5 | 0 | 10 | 52 | 18 | All_GROUP:sk__Eukaryota(100);sknr0__SAR(100);sknr1__Alveolata(100);sknr2__Ciliophora(100);subp__Intramacronucleata(100);c__Spirotrichea(100); |
| OTU_255 | 0 | 0 | 0 | 1 | 0 | 0 | 36 | 6 | 78 | 1 | 3 | 0 | All_GROUP:sk__Eukaryota(100);sknr0__SAR(100);sknr1__Alveolata(100);sknr2__Ciliophora(100);subp__Intramacronucleata(100);c__Spirotrichea(100); |
| OTU_263 | 4 | 1 | 51 | 1 | 1 | 2 | 0 | 0 | 0 | 1 | 2 | 5 | All_GROUP:sk__Eukaryota(100);sknr0__SAR(100);sknr1__Alveolata(100);sknr2__Ciliophora(100);subp__Intramacronucleata(100);c__Spirotrichea(100); |
| OTU_765 | 11 | 0 | 0 | 3 | 1 | 2 | 0 | 0 | 0 | 3 | 20 | 1 | All_GROUP:sk__Eukaryota(100);sknr0__SAR(100);sknr1__Alveolata(100);sknr2__Ciliophora(100);subp__Intramacronucleata(100);c__Spirotrichea(100); |
| OTU_933 | 1 | 7 | 7 | 0 | 0 | 1 | 3 | 0 | 3 | 0 | 0 | 0 | All_GROUP:sk__Eukaryota(100);sknr0__SAR(100);sknr1__Alveolata(100);sknr2__Ciliophora(100);subp__Intramacronucleata(100);c__Spirotrichea(100); |
| OTU_1044 | 16 | 0 | 0 | 0 | 0 | 0 | 0 | 0 | 0 | 0 | 0 | 0 | All_GROUP:sk__Eukaryota(100);sknr0__SAR(100);sknr1__Alveolata(100);sknr2__Ciliophora(100);subp__Intramacronucleata(100);c__Spirotrichea(100); |
| OTU_545 | 3 | 3 | 2 | 0 | 1 | 1 | 1 | 3 | 0 | 5 | 0 | 0 | All_GROUP:sk__Eukaryota(100);sknr0__SAR(100);sknr1__Alveolata(100);sknr2__Ciliophora(100);subp__Intramacronucleata(100);subpnr0__Conthreep(100);c__Colpodea(100);o__Bursariomorphida(100);g__Bryometopus(100);s__Bryometopus_pseudochilodon(82); |
| OTU_362 | 1 | 0 | 0 | 0 | 0 | 0 | 0 | 0 | 59 | 0 | 0 | 2 | All_GROUP:sk__Eukaryota(100);sknr0__SAR(100);sknr1__Alveolata(100);sknr2__Ciliophora(100);subp__Intramacronucleata(100);subpnr0__Conthreep(100);c__Colpodea(100);o__Colpodida(100);g__Colpoda(84);s__Colpoda_magna(84); |
| OTU_808 | 49 | 1 | 4 | 2 | 11 | 9 | 23 | 5 | 8 | 4 | 4 | 0 | All_GROUP:sk__Eukaryota(100);sknr0__SAR(100);sknr1__Alveolata(100);sknr2__Ciliophora(100);subp__Intramacronucleata(100);subpnr0__Conthreep(100);c__Colpodea(100);o__Colpodida(100);g__Bromeliothrix(99);s__Colpoda_steinii(99); |
| OTU_224 | 17 | 1 | 3 | 4 | 12 | 3 | 37 | 0 | 2 | 2 | 0 | 0 | All_GROUP:sk__Eukaryota(100);sknr0__SAR(100);sknr1__Alveolata(100);sknr2__Ciliophora(100);subp__Intramacronucleata(100);subpnr0__Conthreep(100);c__Colpodea(100);o__Colpodida(100);g__Hausmanniella(91);s__Hausmanniella_discoidea(91); |
| OTU_496 | 3 | 2 | 0 | 3 | 0 | 4 | 2 | 3 | 2 | 8 | 3 | 15 | All_GROUP:sk__Eukaryota(100);sknr0__SAR(100);sknr1__Alveolata(100);sknr2__Ciliophora(100);subp__Intramacronucleata(100);subpnr0__Conthreep(100);c__Nassophorea(100);g__Leptopharynx(98);s__Leptopharynx_sp._TS-2011(91); |
| OTU_805 | 22 | 0 | 0 | 0 | 0 | 0 | 0 | 0 | 0 | 0 | 0 | 0 | All_GROUP:sk__Eukaryota(100);sknr0__SAR(100);sknr1__Alveolata(100);sknr2__Ciliophora(100);subp__Intramacronucleata(100);subpnr0__Conthreep(100);c__Nassophorea(98);g__Colpodidium(98);s__Parafurgasonia_sp._s-011(85); |
| OTU_696 | 34 | 0 | 0 | 0 | 0 | 0 | 0 | 0 | 0 | 0 | 0 | 0 | All_GROUP:sk__Eukaryota(100);sknr0__SAR(100);sknr1__Alveolata(100);sknr2__Ciliophora(100);subp__Intramacronucleata(100);subpnr0__Conthreep(100);c__Nassophorea(100);g__Pseudomicrothorax(97);s__Pseudomicrothorax_dubius(97); |
| OTU_291 | 59 | 22 | 23 | 37 | 19 | 34 | 13 | 23 | 42 | 39 | 17 | 24 | All_GROUP:sk__Eukaryota(100);sknr0__SAR(100);sknr1__Alveolata(100);sknr2__Ciliophora(100);subp__Intramacronucleata(100);subpnr0__Conthreep(100);c__Colpodea(100);o__Colpodida(100);s__uncultured_Eimeriidae(100); |
| OTU_267 | 38 | 49 | 20 | 20 | 8 | 16 | 59 | 29 | 70 | 7 | 6 | 16 | All_GROUP:sk__Eukaryota(100);sknr0__SAR(100);sknr1__Alveolata(100);sknr2__Ciliophora(100);subp__Intramacronucleata(100);subpnr0__Conthreep(100);c__Colpodea(100);o__Cyrtolophosidida(100);s__uncultured_Eimeriidae(99); |
| OTU_792 | 0 | 0 | 0 | 0 | 0 | 0 | 2 | 0 | 5 | 0 | 0 | 0 | All_GROUP:sk__Eukaryota(100);sknr0__SAR(100);sknr1__Alveolata(100);sknr2__Ciliophora(100);subp__Intramacronucleata(100);subpnr0__Conthreep(100);c__Colpodea(100);o__Cyrtolophosidida(100);s__uncultured_Eimeriidae(80); |
| OTU_572 | 0 | 0 | 0 | 1 | 0 | 0 | 2 | 0 | 0 | 3 | 2 | 9 | All_GROUP:sk__Eukaryota(100);sknr0__SAR(100);sknr1__Alveolata(100);sknr2__Ciliophora(100);subp__Intramacronucleata(100);subpnr0__Conthreep(100);c__Nassophorea(100);s__uncultured_Microthoracida(96); |
| OTU_308 | 19 | 27 | 2 | 6 | 0 | 0 | 0 | 0 | 0 | 0 | 0 | 0 | All_GROUP:sk__Eukaryota(100);sknr0__SAR(100);sknr1__Alveolata(100);sknr2__Ciliophora(100);subp__Intramacronucleata(100);subpnr0__Conthreep(100);c__Colpodea(100);o__Bursariomorphida(100);g__Bryometopus(100); |
| OTU_1003 | 6 | 0 | 0 | 0 | 0 | 0 | 0 | 0 | 0 | 0 | 0 | 0 | All_GROUP:sk__Eukaryota(100);sknr0__SAR(100);sknr1__Alveolata(100);sknr2__Ciliophora(100);subp__Intramacronucleata(100);subpnr0__Conthreep(100);c__Prostomatea(99);g__Cryptocaryon(97); |
| OTU_607 | 0 | 7 | 0 | 6 | 0 | 1 | 0 | 0 | 7 | 0 | 0 | 14 | All_GROUP:sk__Eukaryota(100);sknr0__SAR(100);sknr1__Alveolata(100);sknr2__Ciliophora(100);subp__Intramacronucleata(100);subpnr0__Conthreep(100);c__Colpodea(100);o__Colpodida(100);g__Maryna(95); |
| OTU_231 | 9 | 16 | 7 | 14 | 25 | 32 | 15 | 11 | 35 | 7 | 2 | 4 | All_GROUP:sk__Eukaryota(100);sknr0__SAR(100);sknr1__Alveolata(100);sknr2__Ciliophora(100);subp__Intramacronucleata(100);subpnr0__Conthreep(100);c__Colpodea(100);o__Colpodida(100); |
| OTU_624 | 0 | 0 | 0 | 0 | 0 | 0 | 7 | 0 | 16 | 0 | 0 | 0 | All_GROUP:sk__Eukaryota(100);sknr0__SAR(100);sknr1__Alveolata(100);sknr2__Ciliophora(100);subp__Intramacronucleata(100);subpnr0__Conthreep(100);c__Colpodea(100);o__Colpodida(100); |
| OTU_664 | 3 | 0 | 0 | 1 | 0 | 0 | 3 | 0 | 2 | 0 | 0 | 0 | All_GROUP:sk__Eukaryota(100);sknr0__SAR(100);sknr1__Alveolata(100);sknr2__Ciliophora(100);subp__Intramacronucleata(100);subpnr0__Conthreep(100);c__Colpodea(100);o__Colpodida(100); |
| OTU_667 | 3 | 0 | 0 | 0 | 1 | 0 | 0 | 3 | 0 | 0 | 0 | 0 | All_GROUP:sk__Eukaryota(100);sknr0__SAR(100);sknr1__Alveolata(100);sknr2__Ciliophora(100);subp__Intramacronucleata(100);subpnr0__Conthreep(100);c__Colpodea(100);o__Colpodida(98); |
| OTU_680 | 5 | 0 | 7 | 0 | 0 | 2 | 0 | 0 | 0 | 0 | 0 | 0 | All_GROUP:sk__Eukaryota(100);sknr0__SAR(100);sknr1__Alveolata(100);sknr2__Ciliophora(100);subp__Intramacronucleata(100);subpnr0__Conthreep(100);c__Colpodea(100);o__Colpodida(100); |
| OTU_697 | 0 | 1 | 0 | 0 | 1 | 0 | 0 | 3 | 24 | 0 | 0 | 0 | All_GROUP:sk__Eukaryota(100);sknr0__SAR(100);sknr1__Alveolata(100);sknr2__Ciliophora(100);subp__Intramacronucleata(100);subpnr0__Conthreep(100);c__Colpodea(100);o__Colpodida(100); |
| OTU_735 | 2 | 1 | 2 | 1 | 0 | 1 | 0 | 2 | 3 | 0 | 4 | 10 | All_GROUP:sk__Eukaryota(100);sknr0__SAR(100);sknr1__Alveolata(100);sknr2__Ciliophora(100);subp__Intramacronucleata(100);subpnr0__Conthreep(100);c__Colpodea(100);o__Colpodida(100); |
| OTU_769 | 0 | 3 | 2 | 1 | 0 | 0 | 10 | 2 | 22 | 0 | 4 | 6 | All_GROUP:sk__Eukaryota(100);sknr0__SAR(100);sknr1__Alveolata(100);sknr2__Ciliophora(100);subp__Intramacronucleata(100);subpnr0__Conthreep(100);c__Colpodea(100);o__Colpodida(100); |
| OTU_800 | 7 | 0 | 2 | 0 | 2 | 0 | 0 | 19 | 0 | 0 | 2 | 0 | All_GROUP:sk__Eukaryota(100);sknr0__SAR(100);sknr1__Alveolata(100);sknr2__Ciliophora(100);subp__Intramacronucleata(100);subpnr0__Conthreep(100);c__Colpodea(100);o__Colpodida(100); |
| OTU_997 | 8 | 0 | 2 | 2 | 1 | 0 | 0 | 0 | 0 | 0 | 0 | 0 | All_GROUP:sk__Eukaryota(100);sknr0__SAR(100);sknr1__Alveolata(100);sknr2__Ciliophora(100);subp__Intramacronucleata(100);subpnr0__Conthreep(100);c__Colpodea(100);o__Colpodida(100); |
| OTU_1016 | 16 | 0 | 0 | 0 | 0 | 0 | 0 | 0 | 0 | 0 | 0 | 0 | All_GROUP:sk__Eukaryota(100);sknr0__SAR(100);sknr1__Alveolata(100);sknr2__Ciliophora(100);subp__Intramacronucleata(100);subpnr0__Conthreep(100);c__Colpodea(100);o__Colpodida(100); |
| OTU_1023 | 9 | 6 | 0 | 1 | 0 | 0 | 0 | 0 | 0 | 0 | 0 | 0 | All_GROUP:sk__Eukaryota(100);sknr0__SAR(100);sknr1__Alveolata(100);sknr2__Ciliophora(100);subp__Intramacronucleata(100);subpnr0__Conthreep(100);c__Colpodea(100);o__Colpodida(100); |
| OTU_1056 | 1 | 2 | 0 | 5 | 0 | 4 | 0 | 0 | 0 | 0 | 5 | 1 | All_GROUP:sk__Eukaryota(100);sknr0__SAR(100);sknr1__Alveolata(100);sknr2__Ciliophora(100);subp__Intramacronucleata(100);subpnr0__Conthreep(100);c__Colpodea(100);o__Colpodida(100); |
| OTU_1062 | 0 | 12 | 0 | 0 | 0 | 0 | 0 | 0 | 0 | 2 | 0 | 0 | All_GROUP:sk__Eukaryota(100);sknr0__SAR(100);sknr1__Alveolata(100);sknr2__Ciliophora(100);subp__Intramacronucleata(100);subpnr0__Conthreep(100);c__Colpodea(100);o__Colpodida(100); |
| OTU_584 | 0 | 0 | 0 | 0 | 0 | 0 | 1 | 1 | 4 | 1 | 0 | 1 | All_GROUP:sk__Eukaryota(100);sknr0__SAR(100);sknr1__Alveolata(100);sknr2__Ciliophora(100);subp__Intramacronucleata(100);subpnr0__Conthreep(100);c__Colpodea(100);o__Cyrtolophosidida(100); |
| OTU_612 | 7 | 5 | 1 | 2 | 1 | 2 | 0 | 1 | 14 | 0 | 0 | 1 | All_GROUP:sk__Eukaryota(100);sknr0__SAR(100);sknr1__Alveolata(100);sknr2__Ciliophora(100);subp__Intramacronucleata(100);subpnr0__Conthreep(100);c__Colpodea(100);o__Cyrtolophosidida(98); |
| OTU_646 | 8 | 17 | 10 | 4 | 0 | 1 | 9 | 0 | 7 | 17 | 10 | 20 | All_GROUP:sk__Eukaryota(100);sknr0__SAR(100);sknr1__Alveolata(100);sknr2__Ciliophora(100);subp__Intramacronucleata(100);subpnr0__Conthreep(100);c__Colpodea(100);o__Cyrtolophosidida(100); |
| OTU_925 | 0 | 0 | 0 | 0 | 0 | 0 | 8 | 0 | 0 | 0 | 0 | 0 | All_GROUP:sk__Eukaryota(100);sknr0__SAR(100);sknr1__Alveolata(100);sknr2__Ciliophora(100);subp__Intramacronucleata(100);subpnr0__Conthreep(100);c__Nassophorea(99); |
| OTU_321 | 2 | 3 | 0 | 0 | 0 | 1 | 1 | 3 | 40 | 0 | 0 | 0 | All_GROUP:sk__Eukaryota(100);sknr0__SAR(99);sknr1__Alveolata(99);sknr2__Ciliophora(99);subp__Intramacronucleata(99);subpnr0__Conthreep(99);c__Oligohymenophorea(98); |
| OTU_702 | 23 | 0 | 1 | 0 | 0 | 0 | 1 | 5 | 0 | 0 | 0 | 0 | All_GROUP:sk__Eukaryota(100);sknr0__SAR(98);sknr1__Alveolata(98);sknr2__Ciliophora(98);subp__Intramacronucleata(98);subpnr0__Conthreep(98);c__Prostomatea(80); |
| OTU_877 | 0 | 19 | 19 | 0 | 0 | 0 | 0 | 0 | 0 | 0 | 0 | 0 | All_GROUP:sk__Eukaryota(100);sknr0__SAR(100);sknr1__Alveolata(100);sknr2__Ciliophora(100);subp__Intramacronucleata(100);subpnr0__Conthreep(100);c__Oligohymenophorea(99);cnr0__CV1-2A-17(98); |
| OTU_1001 | 0 | 2 | 0 | 1 | 0 | 0 | 0 | 1 | 4 | 0 | 0 | 0 | All_GROUP:sk__Eukaryota(100);sknr0__SAR(100);sknr1__Alveolata(100);sknr2__Ciliophora(100);subp__Intramacronucleata(100);subpnr0__Conthreep(100);c__Oligohymenophorea(100);cnr0__CV1-2A-17(98); |
| OTU_616 | 9 | 0 | 0 | 0 | 0 | 0 | 0 | 0 | 0 | 0 | 0 | 0 | All_GROUP:sk__Eukaryota(100);sknr0__SAR(100);sknr1__Alveolata(100);sknr2__Ciliophora(100);subp__Intramacronucleata(100);subpnr0__Conthreep(100);c__Phyllopharyngea(100);subc__Cyrtophoria(100);g__Trithigmostoma(100); |
| OTU_247 | 6 | 67 | 51 | 29 | 19 | 6 | 2 | 2 | 1 | 47 | 8 | 1 | All_GROUP:sk__Eukaryota(100);sknr0__SAR(100);sknr1__Alveolata(100);sknr2__Ciliophora(100);subp__Intramacronucleata(100);subpnr0__Conthreep(100);c__Phyllopharyngea(100);subc__Cyrtophoria(100);g__Trithigmostoma(100);gun0__uncultured_eukaryote(97); |
| OTU_299 | 17 | 10 | 18 | 1 | 1 | 3 | 3 | 0 | 2 | 8 | 0 | 0 | All_GROUP:sk__Eukaryota(100);sknr0__SAR(100);sknr1__Alveolata(100);sknr2__Ciliophora(100);subp__Intramacronucleata(100);subpnr0__Conthreep(100);c__Oligohymenophorea(100);subc__Peritrichia(100); |
| OTU_315 | 49 | 0 | 3 | 34 | 19 | 15 | 1 | 0 | 0 | 29 | 1 | 1 | All_GROUP:sk__Eukaryota(100);sknr0__SAR(87);sknr1__Alveolata(87);sknr2__Ciliophora(87);subp__Intramacronucleata(87);subpnr0__Conthreep(87);c__Oligohymenophorea(87);subc__Peritrichia(87); |
| OTU_459 | 43 | 0 | 17 | 0 | 0 | 0 | 3 | 13 | 0 | 0 | 0 | 0 | All_GROUP:sk__Eukaryota(100);sknr0__SAR(100);sknr1__Alveolata(100);sknr2__Ciliophora(100);subp__Intramacronucleata(100);subpnr0__Conthreep(100);c__Oligohymenophorea(100);subc__Peritrichia(100);g__Zoothamnium(83);gun0__uncultured_eukaryote(83); |
| OTU_108 | 54 | 27 | 21 | 10 | 3 | 8 | 24 | 41 | 34 | 7 | 10 | 22 | All_GROUP:sk__Eukaryota(100);sknr0__SAR(100);sknr1__Alveolata(100);sknr2__Ciliophora(100);subp__Intramacronucleata(100);subpnr0__Conthreep(100);c__Colpodea(100);cnr0__Platyophryida(100);g__Platyophrya(95); |
| OTU_548 | 84 | 17 | 3 | 3 | 3 | 5 | 13 | 61 | 27 | 14 | 5 | 67 | All_GROUP:sk__Eukaryota(100);sknr0__SAR(100);sknr1__Alveolata(100);sknr2__Ciliophora(100);subp__Intramacronucleata(100);subpnr0__Conthreep(100);c__Colpodea(100);cnr0__Platyophryida(100);g__Platyophrya(100); |
| OTU_512 | 31 | 0 | 0 | 1 | 1 | 4 | 0 | 0 | 0 | 0 | 0 | 0 | All_GROUP:sk__Eukaryota(100);sknr0__SAR(100);sknr1__Alveolata(100);sknr2__Ciliophora(100);subp__Intramacronucleata(100);subpnr0__Conthreep(100);c__Colpodea(100);cnr0__Platyophryida(98); |
| OTU_185 | 20 | 22 | 13 | 4 | 5 | 6 | 29 | 48 | 27 | 2 | 3 | 6 | All_GROUP:sk__Eukaryota(100);sknr0__SAR(100);sknr1__Alveolata(100);sknr2__Ciliophora(100);subp__Intramacronucleata(100);subpnr0__Conthreep(100);c__Colpodea(100);o__Colpodida(100);onr0__Pseudoplatyophyra(100);s__Prorodontidae_environmental_sample(100); |
| OTU_314 | 40 | 27 | 32 | 27 | 27 | 22 | 8 | 3 | 55 | 16 | 6 | 29 | All_GROUP:sk__Eukaryota(100);sknr0__SAR(100);sknr1__Alveolata(100);sknr2__Ciliophora(100);subp__Intramacronucleata(100);subpnr0__Conthreep(100);c__Colpodea(100);o__Colpodida(100);onr0__Pseudoplatyophyra(100);s__Pseudoplatyophrya_nana(98); |
| OTU_839 | 17 | 7 | 0 | 0 | 0 | 0 | 2 | 0 | 2 | 0 | 0 | 0 | All_GROUP:sk__Eukaryota(100);sknr0__SAR(100);sknr1__Alveolata(100);sknr2__Ciliophora(100);subp__Intramacronucleata(100);subpnr0__Conthreep(100);c__Oligohymenophorea(100);subc__Scuticociliatia(100);g__Homalogastra(100);s__Orchitophryidae_environmental_sample(82); |
| OTU_60 | 0 | 0 | 1 | 0 | 0 | 0 | 16 | 7 | 20 | 6 | 5 | 34 | All_GROUP:sk__Eukaryota(100);sknr0__SAR(100);sknr1__Alveolata(100);sknr2__Ciliophora(100);subp__Intramacronucleata(100);subpnr0__Conthreep(100);c__Oligohymenophorea(100);subc__Scuticociliatia(100);g__Uronemella(100);s__uncultured_ciliate(100); |
| OTU_282 | 38 | 9 | 7 | 0 | 1 | 3 | 7 | 0 | 0 | 20 | 5 | 14 | All_GROUP:sk__Eukaryota(100);sknr0__SAR(100);sknr1__Alveolata(100);sknr2__Ciliophora(100);subp__Intramacronucleata(100);subpnr0__Conthreep(100);c__Oligohymenophorea(100);subc__Scuticociliatia(100);g__Homalogastra(92); |
| OTU_637 | 0 | 0 | 0 | 0 | 0 | 0 | 19 | 0 | 0 | 0 | 0 | 0 | All_GROUP:sk__Eukaryota(100);sknr0__SAR(100);sknr1__Alveolata(100);sknr2__Ciliophora(100);subp__Intramacronucleata(100);subpnr0__Conthreep(100);c__Oligohymenophorea(100);subc__Scuticociliatia(99); |
| OTU_503 | 0 | 0 | 0 | 0 | 0 | 0 | 0 | 0 | 0 | 0 | 0 | 41 | All_GROUP:sk__Eukaryota(100);sknr0__SAR(100);sknr1__Alveolata(100);sknr2__Ciliophora(100);subp__Intramacronucleata(100);subpnr0__Conthreep(100);c__Oligohymenophorea(100);subc__Scuticociliatia(100);subcnr0__uncultured(100);s__uncultured_Scuticociliatia(100); |
| OTU_826 | 2 | 3 | 10 | 0 | 0 | 1 | 0 | 0 | 1 | 0 | 0 | 0 | All_GROUP:sk__Eukaryota(100);sknr0__SAR(100);sknr1__Alveolata(100);sknr2__Ciliophora(100);subp__Intramacronucleata(100);subpnr0__Conthreep(100);c__Colpodea(100);o__Cyrtolophosidida(100);g__Pseudocyrtolophosis(92);gun0__uncultured_eukaryote(92); |
| OTU_173 | 72 | 0 | 0 | 0 | 0 | 0 | 0 | 0 | 0 | 0 | 0 | 0 | All_GROUP:sk__Eukaryota(100);sknr0__SAR(100);sknr1__Alveolata(100);sknr2__Ciliophora(100);subp__Intramacronucleata(100);c__Litostomatea(100);subc__Haptoria(97); |
| OTU_335 | 12 | 0 | 4 | 1 | 8 | 3 | 1 | 1 | 15 | 0 | 6 | 0 | All_GROUP:sk__Eukaryota(100);sknr0__SAR(100);sknr1__Alveolata(100);sknr2__Ciliophora(100);subp__Intramacronucleata(100);c__Litostomatea(100);subc__Haptoria(100); |
| OTU_381 | 0 | 1 | 2 | 0 | 0 | 0 | 5 | 2 | 20 | 6 | 2 | 27 | All_GROUP:sk__Eukaryota(100);sknr0__SAR(100);sknr1__Alveolata(100);sknr2__Ciliophora(100);subp__Intramacronucleata(100);c__Litostomatea(100);subc__Haptoria(100); |
| OTU_493 | 0 | 3 | 1 | 0 | 0 | 2 | 0 | 0 | 0 | 11 | 0 | 0 | All_GROUP:sk__Eukaryota(100);sknr0__SAR(100);sknr1__Alveolata(100);sknr2__Ciliophora(100);subp__Intramacronucleata(100);c__Litostomatea(100);subc__Haptoria(98); |
| OTU_544 | 17 | 12 | 0 | 0 | 1 | 1 | 0 | 4 | 0 | 0 | 1 | 0 | All_GROUP:sk__Eukaryota(100);sknr0__SAR(100);sknr1__Alveolata(100);sknr2__Ciliophora(100);subp__Intramacronucleata(100);c__Litostomatea(100);subc__Haptoria(100); |
| OTU_623 | 0 | 4 | 0 | 0 | 0 | 0 | 0 | 0 | 0 | 0 | 0 | 0 | All_GROUP:sk__Eukaryota(100);sknr0__SAR(100);sknr1__Alveolata(100);sknr2__Ciliophora(100);subp__Intramacronucleata(100);c__Litostomatea(100);subc__Haptoria(98); |
| OTU_639 | 0 | 0 | 16 | 0 | 2 | 32 | 0 | 0 | 0 | 0 | 0 | 0 | All_GROUP:sk__Eukaryota(100);sknr0__SAR(100);sknr1__Alveolata(100);sknr2__Ciliophora(100);subp__Intramacronucleata(100);c__Litostomatea(100);subc__Haptoria(100); |
| OTU_665 | 0 | 0 | 0 | 1 | 2 | 3 | 0 | 0 | 0 | 3 | 1 | 0 | All_GROUP:sk__Eukaryota(100);sknr0__SAR(100);sknr1__Alveolata(100);sknr2__Ciliophora(100);subp__Intramacronucleata(100);c__Litostomatea(100);subc__Haptoria(99); |
| OTU_683 | 5 | 13 | 0 | 0 | 0 | 9 | 0 | 0 | 0 | 0 | 0 | 0 | All_GROUP:sk__Eukaryota(100);sknr0__SAR(100);sknr1__Alveolata(100);sknr2__Ciliophora(100);subp__Intramacronucleata(100);c__Litostomatea(100);subc__Haptoria(99); |
| OTU_1010 | 0 | 0 | 0 | 1 | 2 | 0 | 0 | 0 | 0 | 0 | 0 | 17 | All_GROUP:sk__Eukaryota(100);sknr0__SAR(100);sknr1__Alveolata(100);sknr2__Ciliophora(100);subp__Intramacronucleata(100);c__Litostomatea(100);subc__Haptoria(100); |
| OTU_1019 | 0 | 0 | 0 | 0 | 0 | 0 | 0 | 0 | 10 | 0 | 1 | 0 | All_GROUP:sk__Eukaryota(100);sknr0__SAR(100);sknr1__Alveolata(100);sknr2__Ciliophora(100);subp__Intramacronucleata(100);c__Litostomatea(100);subc__Haptoria(96); |
| OTU_941 | 0 | 0 | 0 | 6 | 3 | 0 | 0 | 0 | 0 | 0 | 0 | 1 | All_GROUP:sk__Eukaryota(100);sknr0__SAR(100);sknr1__Alveolata(100);sknr2__Ciliophora(100);subp__Intramacronucleata(100);c__Litostomatea(100);subc__Haptoria(100);subcnr0__uncultured(85); |
| OTU_1022 | 9 | 0 | 1 | 0 | 0 | 0 | 0 | 0 | 0 | 0 | 0 | 0 | All_GROUP:sk__Eukaryota(100);sknr0__SAR(100);sknr1__Alveolata(100);sknr2__Ciliophora(100);subp__Intramacronucleata(100);c__Litostomatea(100);subc__Haptoria(100);subcnr0__uncultured(93);subcun1__uncultured_eukaryote(93); |
| OTU_350 | 1 | 0 | 51 | 0 | 0 | 1 | 0 | 0 | 0 | 0 | 2 | 8 | All_GROUP:sk__Eukaryota(100);sknr0__SAR(100);sknr1__Alveolata(100);sknr2__Ciliophora(100);subp__Intramacronucleata(100);c__Spirotrichea(100);subc__Hypotrichia(93);g__Pseudourostyla(87);s__Pseudourostyla_cristata(87); |
| OTU_670 | 0 | 0 | 0 | 0 | 1 | 0 | 5 | 12 | 2 | 0 | 0 | 0 | All_GROUP:sk__Eukaryota(100);sknr0__SAR(100);sknr1__Alveolata(100);sknr2__Ciliophora(100);subp__Intramacronucleata(100);c__Spirotrichea(100);subc__Hypotrichia(100);g__Holosticha(100);s__uncultured_Oxytrichidae(100); |
| OTU_186 | 5 | 1 | 1 | 15 | 10 | 2 | 0 | 0 | 10 | 28 | 4 | 15 | All_GROUP:sk__Eukaryota(100);sknr0__SAR(100);sknr1__Alveolata(100);sknr2__Ciliophora(100);subp__Intramacronucleata(100);c__Spirotrichea(100);subc__Hypotrichia(100);s__uncultured_Oxytrichidae(98); |
| OTU_238 | 5 | 9 | 34 | 1 | 1 | 8 | 2 | 3 | 1 | 15 | 0 | 0 | All_GROUP:sk__Eukaryota(100);sknr0__SAR(100);sknr1__Alveolata(100);sknr2__Ciliophora(100);subp__Intramacronucleata(100);c__Spirotrichea(100);subc__Hypotrichia(100);g__Urostyla(98); |
| OTU_35 | 204 | 489 | 190 | 118 | 119 | 207 | 101 | 292 | 144 | 170 | 245 | 142 | All_GROUP:sk__Eukaryota(100);sknr0__SAR(100);sknr1__Alveolata(100);sknr2__Ciliophora(100);subp__Intramacronucleata(100);c__Spirotrichea(100);subc__Hypotrichia(95); |
| OTU_93 | 62 | 2 | 2 | 0 | 0 | 0 | 7 | 1 | 0 | 52 | 9 | 110 | All_GROUP:sk__Eukaryota(100);sknr0__SAR(100);sknr1__Alveolata(100);sknr2__Ciliophora(100);subp__Intramacronucleata(100);c__Spirotrichea(100);subc__Hypotrichia(83); |
| OTU_94 | 0 | 1 | 1 | 7 | 59 | 5 | 1 | 5 | 0 | 0 | 0 | 0 | All_GROUP:sk__Eukaryota(100);sknr0__SAR(100);sknr1__Alveolata(100);sknr2__Ciliophora(100);subp__Intramacronucleata(100);c__Spirotrichea(100);subc__Hypotrichia(98); |
| OTU_225 | 0 | 0 | 0 | 0 | 0 | 0 | 0 | 0 | 81 | 0 | 0 | 0 | All_GROUP:sk__Eukaryota(100);sknr0__SAR(100);sknr1__Alveolata(100);sknr2__Ciliophora(100);subp__Intramacronucleata(100);c__Spirotrichea(100);subc__Hypotrichia(96); |
| OTU_256 | 0 | 10 | 1 | 0 | 0 | 0 | 1 | 1 | 19 | 2 | 27 | 9 | All_GROUP:sk__Eukaryota(100);sknr0__SAR(100);sknr1__Alveolata(100);sknr2__Ciliophora(100);subp__Intramacronucleata(100);c__Spirotrichea(100);subc__Hypotrichia(100); |
| OTU_520 | 0 | 0 | 0 | 0 | 0 | 0 | 0 | 10 | 0 | 0 | 0 | 0 | All_GROUP:sk__Eukaryota(100);sknr0__SAR(100);sknr1__Alveolata(100);sknr2__Ciliophora(100);subp__Intramacronucleata(100);c__Spirotrichea(100);subc__Hypotrichia(90); |
| OTU_588 | 0 | 3 | 0 | 5 | 0 | 8 | 0 | 0 | 0 | 0 | 0 | 0 | All_GROUP:sk__Eukaryota(100);sknr0__SAR(100);sknr1__Alveolata(100);sknr2__Ciliophora(100);subp__Intramacronucleata(100);c__Spirotrichea(100);subc__Hypotrichia(100); |
| OTU_252 | 7 | 11 | 0 | 1 | 0 | 0 | 1 | 18 | 9 | 0 | 0 | 0 | All_GROUP:sk__Eukaryota(100);sknr0__SAR(100);sknr1__Alveolata(100);sknr2__Ciliophora(100);subp__Postciliodesmatophora(100);c__Heterotrichea(100);g__Blepharisma(100); |
| OTU_1084 | 0 | 12 | 0 | 0 | 0 | 0 | 0 | 0 | 0 | 0 | 0 | 0 | All_GROUP:sk__Eukaryota(100);sknr0__SAR(100);sknr1__Alveolata(100);sknr2__Ciliophora(100);subp__Postciliodesmatophora(100);c__Heterotrichea(100);g__Blepharisma(100); |
| OTU_334 | 29 | 1 | 3 | 1 | 3 | 0 | 0 | 0 | 1 | 1 | 2 | 0 | All_GROUP:sk__Eukaryota(100);sknr0__SAR(100);sknr1__Alveolata(100);c__Dinoflagellata(87);c__Dinophyceae(82); |
| OTU_706 | 15 | 11 | 13 | 0 | 0 | 0 | 0 | 0 | 0 | 0 | 0 | 0 | All_GROUP:sk__Eukaryota(100);sknr0__SAR(100);sknr1__Alveolata(100);c__Dinoflagellata(87);c__Dinophyceae(85); |
| OTU_3 | 551 | 298 | 348 | 13 | 5 | 5 | 2292 | 1498 | 2744 | 1463 | 2312 | 110 | All_GROUP:sk__Eukaryota(99);sknr0__SAR(84);sknr1__Alveolata(80); |
| OTU_86 | 0 | 0 | 0 | 0 | 0 | 0 | 0 | 0 | 187 | 0 | 0 | 0 | All_GROUP:sk__Eukaryota(100);sknr0__SAR(94);sknr1__Alveolata(90); |
| OTU_556 | 1 | 10 | 2 | 0 | 0 | 0 | 0 | 0 | 0 | 0 | 0 | 8 | All_GROUP:sk__Eukaryota(100);sknr0__SAR(100);sknr1__Alveolata(100); |
| OTU_935 | 0 | 0 | 0 | 0 | 0 | 0 | 10 | 6 | 1 | 0 | 0 | 0 | All_GROUP:sk__Eukaryota(100);sknr0__SAR(93);sknr1__Alveolata(93); |
| OTU_591 | 21 | 0 | 0 | 0 | 0 | 0 | 0 | 0 | 0 | 0 | 0 | 0 | All_GROUP:sk__Eukaryota(100);sknr0__SAR(99);sknr1__Alveolata(99);sknr2__Protalveolata(99);f__Perkinsidae(95);fnr0__A31(95); |
| OTU_734 | 0 | 0 | 0 | 0 | 0 | 0 | 4 | 8 | 0 | 0 | 0 | 0 | All_GROUP:sk__Eukaryota(100);sknr0__Amoebozoa(90); |
| OTU_743 | 0 | 2 | 0 | 0 | 0 | 0 | 1 | 7 | 7 | 0 | 0 | 5 | All_GROUP:sk__Eukaryota(100);sknr0__Amoebozoa(84); |
| OTU_484 | 3 | 0 | 5 | 0 | 0 | 0 | 22 | 2 | 0 | 3 | 0 | 0 | All_GROUP:sk__Eukaryota(100);sknr0__Amoebozoa(100);sknr1__Cavosteliida(100);g__Schizoplasmodiopsis(100);s__Schizoplasmodiopsis_vulgaris(100); |
| OTU_779 | 0 | 0 | 0 | 2 | 2 | 0 | 0 | 0 | 0 | 2 | 2 | 1 | All_GROUP:sk__Eukaryota(100);sknr0__Amoebozoa(100);sknr1__Cavosteliida(100);g__Schizoplasmodiopsis(100);s__Schizoplasmodiopsis_vulgaris(100); |
| OTU_417 | 13 | 7 | 8 | 5 | 2 | 4 | 0 | 0 | 0 | 0 | 0 | 0 | All_GROUP:sk__Eukaryota(100);sknr0__Amoebozoa(100);sknr1__Cavosteliida(100);sknr2__MPE1-14(100);skun3__uncultured_eukaryote(99); |
| OTU_645 | 4 | 0 | 0 | 0 | 0 | 0 | 3 | 1 | 6 | 0 | 0 | 0 | All_GROUP:sk__Eukaryota(100);sknr0__Amoebozoa(100);sknr1__Cavosteliida(99);sknr2__MPE1-14(99);skun3__uncultured_eukaryote(97); |
| OTU_351 | 10 | 33 | 6 | 3 | 4 | 4 | 1 | 0 | 0 | 9 | 1 | 10 | All_GROUP:sk__Eukaryota(100);sknr0__Amoebozoa(100);sknr1__Discosea(100);sknr2__Flabellinia(100);sknr3__Dactylopodida(100);s__Amoebozoa_sp._Tmp4(95); |
| OTU_618 | 0 | 0 | 0 | 0 | 0 | 0 | 0 | 0 | 0 | 3 | 3 | 6 | All_GROUP:sk__Eukaryota(100);sknr0__Amoebozoa(100);sknr1__Discosea(100);sknr2__Flabellinia(100);sknr3__Dactylopodida(100);s__Amoebozoa_sp._Tmp4(95); |
| OTU_1061 | 0 | 0 | 0 | 0 | 0 | 1 | 0 | 0 | 1 | 17 | 2 | 7 | All_GROUP:sk__Eukaryota(100);sknr0__Amoebozoa(100);sknr1__Discosea(100);sknr2__Longamoebia(100);sknr3__Centramoebida(100);g__Acanthamoeba(99); |
| OTU_530 | 4 | 0 | 4 | 0 | 1 | 1 | 4 | 3 | 3 | 1 | 4 | 7 | All_GROUP:sk__Eukaryota(100);sknr0__Amoebozoa(100);sknr1__Gracilipodida(100);g__Filamoeba(100);s__Filamoeba_nolandi(100); |
| OTU_365 | 0 | 2 | 1 | 4 | 1 | 5 | 7 | 6 | 36 | 2 | 0 | 0 | All_GROUP:sk__Eukaryota(100);sknr0__Amoebozoa(100);sknr1__Gracilipodida(100);g__Flamella(100);s__Flamella_arnhemensis(100); |
| OTU_303 | 0 | 0 | 8 | 1 | 2 | 0 | 8 | 1 | 15 | 5 | 3 | 23 | All_GROUP:sk__Eukaryota(100);sknr0__Amoebozoa(100);sknr1__Gracilipodida(100);g__Flamella(100);s__Flamella_balnearia(100); |
| OTU_273 | 34 | 13 | 6 | 6 | 3 | 5 | 6 | 0 | 2 | 6 | 2 | 1 | All_GROUP:sk__Eukaryota(100);sknr0__Amoebozoa(100);sknr1__Gracilipodida(100);g__Flamella(100);s__Lobosea_sp._Borok(100); |
| OTU_398 | 0 | 2 | 0 | 0 | 0 | 0 | 17 | 2 | 10 | 0 | 2 | 0 | All_GROUP:sk__Eukaryota(100);sknr0__Amoebozoa(84);sknr1__Gracilipodida(81);s__soil_amoeba_AND16(81); |
| OTU_489 | 29 | 2 | 0 | 0 | 0 | 1 | 0 | 0 | 0 | 0 | 0 | 0 | All_GROUP:sk__Eukaryota(100);sknr0__Amoebozoa(100);sknr1__Gracilipodida(88);s__soil_amoeba_AND16(88); |
| OTU_570 | 7 | 2 | 1 | 4 | 2 | 0 | 0 | 0 | 0 | 6 | 1 | 5 | All_GROUP:sk__Eukaryota(100);sknr0__Amoebozoa(100);sknr1__Gracilipodida(100);s__soil_amoeba_AND16(100); |
| OTU_685 | 2 | 3 | 5 | 1 | 3 | 3 | 0 | 0 | 0 | 2 | 1 | 2 | All_GROUP:sk__Eukaryota(100);sknr0__Amoebozoa(94);sknr1__Gracilipodida(86);s__soil_amoeba_AND16(86); |
| OTU_690 | 23 | 2 | 12 | 4 | 3 | 3 | 0 | 10 | 10 | 7 | 2 | 1 | All_GROUP:sk__Eukaryota(100);sknr0__Amoebozoa(100);sknr1__Gracilipodida(100);s__soil_amoeba_AND16(100); |
| OTU_764 | 4 | 5 | 4 | 3 | 0 | 1 | 0 | 0 | 0 | 0 | 0 | 2 | All_GROUP:sk__Eukaryota(100);sknr0__Amoebozoa(94);sknr1__Gracilipodida(84);s__soil_amoeba_AND16(84); |
| OTU_766 | 6 | 0 | 0 | 2 | 3 | 0 | 0 | 0 | 0 | 0 | 1 | 3 | All_GROUP:sk__Eukaryota(100);sknr0__Amoebozoa(95);sknr1__Gracilipodida(84);s__soil_amoeba_AND16(84); |
| OTU_559 | 5 | 10 | 2 | 0 | 0 | 0 | 7 | 6 | 14 | 3 | 0 | 2 | All_GROUP:sk__Eukaryota(100);sknr0__Amoebozoa(95);sknr1__Gracilipodida(89);g__Telaepolella(87);s__Telaepolella_tubasferens(87); |
| OTU_628 | 9 | 0 | 0 | 2 | 3 | 4 | 0 | 0 | 0 | 0 | 0 | 0 | All_GROUP:sk__Eukaryota(100);sknr0__Amoebozoa(100);sknr1__Gracilipodida(100);g__Telaepolella(100);s__Telaepolella_tubasferens(100); |
| OTU_818 | 0 | 8 | 0 | 8 | 3 | 4 | 0 | 0 | 0 | 0 | 2 | 0 | All_GROUP:sk__Eukaryota(100);sknr0__Amoebozoa(100);sknr1__Gracilipodida(100);g__Flamella(100);s__uncultured_Eimeriidae(100); |
| OTU_904 | 0 | 5 | 0 | 1 | 0 | 1 | 5 | 0 | 7 | 0 | 2 | 0 | All_GROUP:sk__Eukaryota(100);sknr0__Amoebozoa(100);sknr1__Gracilipodida(100);g__Flamella(100);s__uncultured_Eimeriidae(100); |
| OTU_393 | 0 | 6 | 0 | 1 | 1 | 5 | 5 | 0 | 0 | 0 | 0 | 0 | All_GROUP:sk__Eukaryota(100);sknr0__Amoebozoa(100);sknr1__Gracilipodida(100);g__Flamella(100);s__uncultured_marine_picoeukaryote(100); |
| OTU_119 | 23 | 26 | 12 | 24 | 10 | 21 | 10 | 1 | 5 | 9 | 6 | 9 | All_GROUP:sk__Eukaryota(100);sknr0__Amoebozoa(100);sknr1__Gracilipodida(100);g__Filamoeba(100); |
| OTU_554 | 1 | 9 | 1 | 5 | 1 | 3 | 0 | 3 | 0 | 1 | 2 | 1 | All_GROUP:sk__Eukaryota(100);sknr0__Amoebozoa(100);sknr1__Gracilipodida(100);g__Filamoeba(100); |
| OTU_728 | 18 | 6 | 0 | 1 | 0 | 0 | 0 | 0 | 1 | 0 | 4 | 0 | All_GROUP:sk__Eukaryota(100);sknr0__Amoebozoa(100);sknr1__Gracilipodida(100);g__Filamoeba(100); |
| OTU_1069 | 0 | 6 | 0 | 0 | 2 | 0 | 0 | 0 | 0 | 0 | 0 | 0 | All_GROUP:sk__Eukaryota(100);sknr0__Amoebozoa(100);sknr1__Gracilipodida(100);g__Filamoeba(100); |
| OTU_510 | 14 | 14 | 1 | 1 | 1 | 6 | 0 | 0 | 0 | 8 | 0 | 0 | All_GROUP:sk__Eukaryota(100);sknr0__Amoebozoa(100);sknr1__Gracilipodida(100);g__Flamella(100); |
| OTU_539 | 3 | 2 | 16 | 1 | 2 | 1 | 1 | 0 | 0 | 0 | 0 | 0 | All_GROUP:sk__Eukaryota(100);sknr0__Amoebozoa(100);sknr1__Gracilipodida(100);g__Flamella(100); |
| OTU_482 | 25 | 2 | 1 | 0 | 0 | 0 | 0 | 0 | 0 | 0 | 0 | 37 | All_GROUP:sk__Eukaryota(100);sknr0__Amoebozoa(100);sknr1__Gracilipodida(100);g__Flamella(100);gnr0__Lobosea_sp._H9a_3E(100); |
| OTU_751 | 7 | 3 | 6 | 1 | 0 | 3 | 9 | 3 | 5 | 5 | 1 | 4 | All_GROUP:sk__Eukaryota(100);sknr0__Amoebozoa(100);sknr1__LEMD255(100);s__uncultured_Eimeriidae(100); |
| OTU_389 | 5 | 11 | 7 | 3 | 0 | 2 | 0 | 0 | 1 | 2 | 3 | 10 | All_GROUP:sk__Eukaryota(100);sknr0__Amoebozoa(100);sknr1__LEMD255(100);skun2__uncultured_eukaryote(100); |
| OTU_870 | 0 | 1 | 0 | 0 | 1 | 0 | 7 | 3 | 9 | 2 | 0 | 0 | All_GROUP:sk__Eukaryota(100);sknr0__Amoebozoa(100);sknr1__LKM74(100);s__uncultured_freshwater_eukaryote(100); |
| OTU_1043 | 0 | 0 | 2 | 0 | 0 | 0 | 0 | 0 | 0 | 7 | 0 | 12 | All_GROUP:sk__Eukaryota(100);sknr0__Amoebozoa(100);sknr1__Schizoplasmodiida(100);g__Phalansterium(100);s__Phalansterium_sp._1_JFP-2013(86); |
| OTU_368 | 0 | 1 | 8 | 0 | 0 | 0 | 41 | 0 | 0 | 0 | 16 | 9 | All_GROUP:sk__Eukaryota(100);sknr0__Amoebozoa(100);sknr1__Schizoplasmodiida(100);g__Phalansterium(100);s__Phalansterium_sp._SR1-9H(92); |
| OTU_1015 | 0 | 0 | 0 | 0 | 0 | 0 | 1 | 0 | 7 | 0 | 0 | 0 | All_GROUP:sk__Eukaryota(100);sknr0__Amoebozoa(100);sknr1__Schizoplasmodiida(100);g__Phalansterium(100); |
| OTU_1085 | 3 | 0 | 0 | 0 | 0 | 0 | 0 | 0 | 15 | 0 | 0 | 0 | All_GROUP:sk__Eukaryota(100);sknr0__Amoebozoa(100);sknr1__Schizoplasmodiida(100);g__Phalansterium(100); |
| OTU_285 | 2 | 1 | 5 | 0 | 0 | 0 | 5 | 16 | 31 | 1 | 0 | 0 | All_GROUP:sk__Eukaryota(100);sknr0__Amoebozoa(87);sknr1__Schizoplasmodiida(81); |
| OTU_319 | 14 | 28 | 21 | 0 | 0 | 0 | 7 | 0 | 15 | 10 | 0 | 14 | All_GROUP:sk__Eukaryota(100);sknr0__Amoebozoa(96);sknr1__Schizoplasmodiida(95); |
| OTU_355 | 1 | 5 | 0 | 0 | 0 | 0 | 16 | 1 | 19 | 22 | 1 | 6 | All_GROUP:sk__Eukaryota(100);sknr0__Amoebozoa(95);sknr1__Schizoplasmodiida(92); |
| OTU_374 | 13 | 26 | 36 | 1 | 2 | 3 | 5 | 1 | 3 | 3 | 1 | 3 | All_GROUP:sk__Eukaryota(100);sknr0__Amoebozoa(95);sknr1__Schizoplasmodiida(95); |
| OTU_687 | 34 | 2 | 0 | 0 | 0 | 0 | 8 | 0 | 2 | 12 | 0 | 1 | All_GROUP:sk__Eukaryota(100);sknr0__Amoebozoa(92);sknr1__Schizoplasmodiida(91); |
| OTU_831 | 5 | 5 | 6 | 0 | 0 | 0 | 0 | 0 | 3 | 0 | 0 | 0 | All_GROUP:sk__Eukaryota(100);sknr0__Amoebozoa(93);sknr1__Schizoplasmodiida(93); |
| OTU_940 | 0 | 0 | 16 | 0 | 1 | 1 | 0 | 0 | 4 | 4 | 0 | 0 | All_GROUP:sk__Eukaryota(100);sknr0__Amoebozoa(96);sknr1__Schizoplasmodiida(96); |
| OTU_967 | 3 | 0 | 1 | 0 | 0 | 0 | 0 | 0 | 0 | 3 | 0 | 0 | All_GROUP:sk__Eukaryota(100);sknr0__Amoebozoa(96);sknr1__Schizoplasmodiida(96); |
| OTU_676 | 0 | 3 | 3 | 3 | 2 | 2 | 1 | 0 | 22 | 0 | 0 | 0 | All_GROUP:sk__Eukaryota(100);sknr0__Amoebozoa(100);sknr1__Tubulinea(100);o__Leptomyxida(100);g__Leptomyxa(99);s__Ripidomyxa_sp._RP009(84); |
| OTU_481 | 46 | 19 | 21 | 2 | 6 | 9 | 18 | 4 | 17 | 0 | 5 | 4 | All_GROUP:sk__Eukaryota(100);sknr0__Amoebozoa(100);sknr1__Tubulinea(100);o__Leptomyxida(100);s__uncultured_Eimeriidae(98); |
| OTU_569 | 0 | 0 | 0 | 1 | 1 | 0 | 28 | 0 | 16 | 0 | 1 | 4 | All_GROUP:sk__Eukaryota(100);sknr0__Amoebozoa(100);sknr1__Tubulinea(100);o__Leptomyxida(100);s__uncultured_Eimeriidae(100); |
| OTU_1039 | 0 | 4 | 1 | 0 | 2 | 2 | 0 | 0 | 1 | 0 | 0 | 0 | All_GROUP:sk__Eukaryota(100);sknr0__Amoebozoa(100);sknr1__Tubulinea(100);o__Leptomyxida(100);g__Leptomyxa(82); |
| OTU_210 | 8 | 2 | 3 | 3 | 1 | 0 | 4 | 0 | 2 | 44 | 6 | 1 | All_GROUP:sk__Eukaryota(100);sknr0__Amoebozoa(100);sknr1__Tubulinea(100);o__Leptomyxida(100); |
| OTU_421 | 0 | 2 | 0 | 5 | 3 | 1 | 0 | 0 | 0 | 0 | 0 | 0 | All_GROUP:sk__Eukaryota(100);sknr0__Amoebozoa(100);sknr1__Tubulinea(100);o__Leptomyxida(100); |
| OTU_699 | 4 | 0 | 10 | 0 | 0 | 0 | 0 | 0 | 0 | 0 | 0 | 0 | All_GROUP:sk__Eukaryota(100);sknr0__Amoebozoa(100);sknr1__Tubulinea(100);o__Leptomyxida(100); |
| OTU_733 | 0 | 0 | 0 | 0 | 0 | 0 | 0 | 0 | 12 | 0 | 0 | 0 | All_GROUP:sk__Eukaryota(100);sknr0__Amoebozoa(100);sknr1__Tubulinea(100);o__Leptomyxida(100); |
| OTU_789 | 2 | 0 | 0 | 1 | 1 | 1 | 0 | 0 | 5 | 0 | 0 | 0 | All_GROUP:sk__Eukaryota(100);sknr0__Amoebozoa(100);sknr1__Tubulinea(100);o__Leptomyxida(100); |
| OTU_1088 | 4 | 2 | 0 | 0 | 0 | 1 | 0 | 1 | 0 | 0 | 0 | 0 | All_GROUP:sk__Eukaryota(100);sknr0__Amoebozoa(100);sknr1__Tubulinea(100);o__Leptomyxida(100); |
| OTU_214 | 23 | 14 | 34 | 0 | 1 | 1 | 4 | 3 | 12 | 22 | 2 | 10 | All_GROUP:sk__Eukaryota(100);sknr0__Amoebozoa(89);sknr1__Tubulinea(89); |
| OTU_543 | 15 | 0 | 2 | 0 | 0 | 0 | 1 | 0 | 0 | 0 | 0 | 0 | All_GROUP:sk__Eukaryota(100);sknr0__Amoebozoa(94);sknr1__Tubulinea(94); |
| OTU_600 | 5 | 3 | 1 | 4 | 1 | 1 | 6 | 0 | 2 | 2 | 4 | 2 | All_GROUP:sk__Eukaryota(100);sknr0__Amoebozoa(93);sknr1__Tubulinea(91); |
| OTU_930 | 8 | 2 | 3 | 0 | 0 | 0 | 0 | 0 | 0 | 0 | 0 | 2 | All_GROUP:sk__Eukaryota(100);sknr0__Amoebozoa(96);sknr1__Tubulinea(96); |
| OTU_939 | 13 | 5 | 0 | 1 | 0 | 0 | 2 | 1 | 9 | 3 | 0 | 0 | All_GROUP:sk__Eukaryota(100);sknr0__Amoebozoa(95);sknr1__Tubulinea(95); |
| OTU_176 | 7 | 6 | 11 | 26 | 12 | 30 | 14 | 53 | 34 | 51 | 16 | 40 | All_GROUP:sk__Eukaryota(100);sknr0__Amoebozoa(100);sknr1__Tubulinea(100);o__Arcellinida(100);onr0__Echinamoebida(100);g__Vermamoeba(100);s__Vermamoeba_vermiformis(96); |
| OTU_329 | 15 | 16 | 21 | 12 | 20 | 8 | 2 | 6 | 6 | 8 | 2 | 4 | All_GROUP:sk__Eukaryota(100);sknr0__Amoebozoa(100);sknr1__Tubulinea(100);o__Arcellinida(100);onr0__Echinamoebida(100);g__Vermamoeba(100);s__Vermamoeba_vermiformis(100); |
| OTU_887 | 0 | 0 | 0 | 0 | 0 | 0 | 0 | 8 | 0 | 0 | 0 | 2 | All_GROUP:sk__Eukaryota(100);sknr0__Amoebozoa(95);sknr1__Tubulinea(95);o__Arcellinida(95);onr0__Echinamoebida(95);g__Echinamoeba(95); |
| OTU_83 | 86 | 44 | 2 | 3 | 10 | 3 | 3 | 0 | 28 | 14 | 9 | 27 | All_GROUP:sk__Eukaryota(100);sknr0__Amoebozoa(100);sknr1__Tubulinea(100);sknr2__Euamoebida(100);g__Glaeseria(100); |
| OTU_508 | 5 | 1 | 2 | 0 | 4 | 2 | 11 | 3 | 13 | 12 | 7 | 1 | All_GROUP:sk__Eukaryota(100);sknr0__Amoebozoa(99);sknr1__Tubulinea(99);sknr2__Euamoebida(98);g__Glaeseria(95); |
| OTU_630 | 3 | 2 | 1 | 5 | 0 | 0 | 0 | 0 | 0 | 0 | 0 | 0 | All_GROUP:sk__Eukaryota(100);sknr0__Amoebozoa(98);sknr1__Tubulinea(98);sknr2__Euamoebida(97);g__Glaeseria(95); |
| OTU_707 | 8 | 2 | 4 | 2 | 2 | 0 | 2 | 0 | 0 | 0 | 0 | 0 | All_GROUP:sk__Eukaryota(100);sknr0__Amoebozoa(91);sknr1__Tubulinea(91);sknr2__Euamoebida(91);g__Glaeseria(91); |
| OTU_882 | 23 | 1 | 0 | 0 | 0 | 0 | 0 | 0 | 2 | 1 | 0 | 2 | All_GROUP:sk__Eukaryota(100);sknr0__Amoebozoa(96);sknr1__Tubulinea(94);sknr2__Euamoebida(94);g__Glaeseria(94); |
| OTU_996 | 14 | 0 | 0 | 0 | 0 | 0 | 0 | 0 | 0 | 0 | 0 | 0 | All_GROUP:sk__Eukaryota(100);sknr0__Amoebozoa(98);sknr1__Tubulinea(98);sknr2__Euamoebida(98);g__Glaeseria(98); |
| OTU_1048 | 0 | 0 | 0 | 0 | 0 | 0 | 1 | 0 | 0 | 3 | 0 | 14 | All_GROUP:sk__Eukaryota(100);sknr0__Amoebozoa(99);sknr1__Tubulinea(98);sknr2__Euamoebida(97);g__Glaeseria(97); |
| OTU_1087 | 4 | 0 | 0 | 0 | 0 | 0 | 0 | 0 | 0 | 1 | 2 | 0 | All_GROUP:sk__Eukaryota(100);sknr0__Amoebozoa(100);sknr1__Tubulinea(100);sknr2__Euamoebida(100);g__Glaeseria(100); |
| OTU_434 | 8 | 9 | 3 | 2 | 6 | 5 | 6 | 1 | 4 | 3 | 0 | 1 | All_GROUP:sk__Eukaryota(100);sknr0__Amoebozoa(85);sknr1__Tubulinea(84);sknr2__Euamoebida(81); |
| OTU_140 | 0 | 1 | 0 | 0 | 2 | 2 | 92 | 0 | 0 | 0 | 0 | 0 | All_GROUP:sk__Eukaryota(100);sknr0__Amoebozoa(100);sknr1__Tubulinea(100);sknr2__Euamoebida(100);sknr3__BOLA868(100);s__uncultured_Lobosea(100); |
| OTU_164 | 0 | 0 | 0 | 37 | 51 | 35 | 0 | 0 | 0 | 0 | 0 | 0 | All_GROUP:sk__Eukaryota(100);sknr0__Amoebozoa(100);sknr1__Tubulinea(100);sknr2__Euamoebida(100);sknr3__BOLA868(100);s__uncultured_Lobosea(100); |
| OTU_269 | 46 | 10 | 9 | 0 | 0 | 0 | 1 | 4 | 15 | 0 | 0 | 0 | All_GROUP:sk__Eukaryota(100);sknr0__Amoebozoa(92);sknr1__Tubulinea(92);sknr2__Euamoebida(92);sknr3__BOLA868(92);s__uncultured_Lobosea(92); |
| OTU_275 | 17 | 2 | 2 | 16 | 24 | 8 | 0 | 0 | 0 | 0 | 0 | 0 | All_GROUP:sk__Eukaryota(100);sknr0__Amoebozoa(100);sknr1__Tubulinea(100);sknr2__Euamoebida(100);sknr3__BOLA868(100); |
| OTU_533 | 0 | 0 | 0 | 0 | 0 | 0 | 3 | 17 | 2 | 4 | 1 | 0 | All_GROUP:sk__Eukaryota(100);sknr0__Amoebozoa(98);sknr1__Tubulinea(98);sknr2__Euamoebida(98);sknr3__BOLA868(98); |
| OTU_969 | 6 | 1 | 3 | 1 | 2 | 2 | 0 | 0 | 0 | 0 | 0 | 0 | All_GROUP:sk__Eukaryota(100);sknr0__Amoebozoa(100);sknr1__Tubulinea(100);sknr2__Euamoebida(100);sknr3__BOLA868(100); |
| OTU_43 | 135 | 331 | 102 | 32 | 38 | 27 | 95 | 46 | 69 | 44 | 15 | 55 | All_GROUP:sk__Eukaryota(100);sknr0__Amoebozoa(100);sknr1__Tubulinea(100);sknr2__Euamoebida(100);sknr3__BOLA868(100);skun4__uncultured_eukaryote(98); |
| OTU_208 | 86 | 7 | 7 | 0 | 0 | 2 | 2 | 1 | 13 | 10 | 2 | 1 | All_GROUP:sk__Eukaryota(100);sknr0__Amoebozoa(100);sknr1__Tubulinea(100);sknr2__Euamoebida(100);sknr3__BOLA868(100);skun4__uncultured_eukaryote(92); |
| OTU_227 | 1 | 0 | 2 | 27 | 42 | 40 | 7 | 1 | 2 | 0 | 0 | 3 | All_GROUP:sk__Eukaryota(100);sknr0__Amoebozoa(100);sknr1__Tubulinea(100);sknr2__Euamoebida(100);sknr3__BOLA868(100);skun4__uncultured_eukaryote(89); |
| OTU_471 | 5 | 0 | 4 | 0 | 1 | 0 | 0 | 0 | 0 | 0 | 0 | 0 | All_GROUP:sk__Eukaryota(100);sknr0__Amoebozoa(100);sknr1__Tubulinea(100);sknr2__Euamoebida(100);sknr3__BOLA868(100);skun4__uncultured_eukaryote(96); |
| OTU_673 | 10 | 2 | 0 | 0 | 0 | 0 | 0 | 0 | 0 | 0 | 0 | 0 | All_GROUP:sk__Eukaryota(100);sknr0__Amoebozoa(100);sknr1__Tubulinea(100);sknr2__Euamoebida(100);sknr3__BOLA868(100);skun4__uncultured_eukaryote(96); |
| OTU_717 | 0 | 6 | 1 | 1 | 0 | 0 | 1 | 0 | 1 | 0 | 0 | 1 | All_GROUP:sk__Eukaryota(100);sknr0__Amoebozoa(100);sknr1__Tubulinea(100);sknr2__Euamoebida(100);sknr3__BOLA868(100);skun4__uncultured_eukaryote(100); |
| OTU_753 | 0 | 2 | 0 | 0 | 0 | 3 | 0 | 5 | 0 | 0 | 0 | 0 | All_GROUP:sk__Eukaryota(100);sknr0__Amoebozoa(100);sknr1__Tubulinea(100);sknr2__Euamoebida(100);sknr3__BOLA868(100);skun4__uncultured_eukaryote(98); |
| OTU_880 | 0 | 0 | 0 | 0 | 0 | 0 | 0 | 0 | 0 | 0 | 0 | 16 | All_GROUP:sk__Eukaryota(100);sknr0__Amoebozoa(100);sknr1__Tubulinea(100);sknr2__Euamoebida(100);sknr3__BOLA868(100);skun4__uncultured_eukaryote(99); |
| OTU_891 | 0 | 0 | 0 | 2 | 0 | 0 | 1 | 0 | 1 | 3 | 7 | 1 | All_GROUP:sk__Eukaryota(100);sknr0__Amoebozoa(100);sknr1__Tubulinea(100);sknr2__Euamoebida(100);sknr3__BOLA868(100);skun4__uncultured_eukaryote(91); |
| OTU_909 | 5 | 13 | 26 | 0 | 0 | 2 | 0 | 0 | 0 | 0 | 0 | 0 | All_GROUP:sk__Eukaryota(100);sknr0__Amoebozoa(100);sknr1__Tubulinea(100);sknr2__Euamoebida(100);sknr3__BOLA868(100);skun4__uncultured_eukaryote(85); |
| OTU_911 | 4 | 3 | 1 | 0 | 0 | 0 | 2 | 5 | 3 | 3 | 0 | 2 | All_GROUP:sk__Eukaryota(100);sknr0__Amoebozoa(100);sknr1__Tubulinea(100);sknr2__Euamoebida(100);sknr3__BOLA868(100);skun4__uncultured_eukaryote(99); |
| OTU_914 | 0 | 0 | 0 | 3 | 1 | 1 | 0 | 0 | 0 | 0 | 0 | 0 | All_GROUP:sk__Eukaryota(100);sknr0__Amoebozoa(100);sknr1__Tubulinea(100);sknr2__Euamoebida(100);sknr3__BOLA868(100);skun4__uncultured_eukaryote(99); |
| OTU_615 | 16 | 7 | 3 | 0 | 0 | 3 | 1 | 2 | 13 | 12 | 7 | 28 | All_GROUP:sk__Eukaryota(100);sknr0__Amoebozoa(100);sknr1__Tubulinea(100);o__Arcellinida(100);subo__Phryganellina(100);subonr0__Cryptodifflugia(100);s__Cryptodifflugia_operculata(97); |
| OTU_926 | 0 | 0 | 0 | 0 | 0 | 0 | 12 | 4 | 7 | 1 | 1 | 3 | All_GROUP:sk__Eukaryota(100);sknr0__Amoebozoa(100);sknr1__Tubulinea(100);o__Arcellinida(100);subo__Phryganellina(100);subonr0__Cryptodifflugia(100);s__Cryptodifflugia_operculata(100); |
| OTU_988 | 0 | 1 | 7 | 0 | 0 | 0 | 3 | 0 | 0 | 0 | 0 | 6 | All_GROUP:sk__Eukaryota(100);sknr0__Amoebozoa(100);sknr1__WIM5(100);s__uncultured_Acanthamoeba(100); |
| OTU_598 | 0 | 0 | 0 | 1 | 3 | 0 | 0 | 0 | 0 | 1 | 0 | 3 | All_GROUP:sk__Eukaryota(100);sknr0__Amoebozoa(100);sknr1__WIM5(99);skun2__uncultured_eukaryote(99); |
| OTU_876 | 0 | 2 | 0 | 0 | 0 | 0 | 0 | 0 | 0 | 0 | 1 | 5 | All_GROUP:sk__Eukaryota(100);sknr0__Amoebozoa(100);sknr1__WIM5(100);sknr2__WIM_1_lineage(100);skun3__uncultured_eukaryote(100); |
| OTU_338 | 10 | 3 | 21 | 3 | 4 | 7 | 0 | 0 | 1 | 3 | 0 | 0 | All_GROUP:sk__Eukaryota(100);sknr0__Archaeplastida(100);sknr1__Chloroplastida(100);p__Chlorophyta(100);c__Chlorophyceae(100);o__Chlamydomonadales(99);g__Chlamydomonas(97);s__Chlamydomonas_reinhardtii(97); |
| OTU_627 | 6 | 1 | 4 | 0 | 0 | 0 | 1 | 6 | 3 | 1 | 0 | 0 | All_GROUP:sk__Eukaryota(100);sknr0__Archaeplastida(100);sknr1__Chloroplastida(100);p__Chlorophyta(100);c__Trebouxiophyceae(100);o__Chlorellales(99);g__Chlorella(99);s__'Chlorella'_mirabilis(99); |
| OTU_1046 | 2 | 11 | 5 | 1 | 1 | 1 | 0 | 0 | 0 | 3 | 0 | 1 | All_GROUP:sk__Eukaryota(100);sknr0__Archaeplastida(100);sknr1__Chloroplastida(100);p__Chlorophyta(100);c__Chlorophyceae(100);o__Chlamydomonadales(100);g__Chlorococcum(99);s__Chlorococcum_echinozygotum(99); |
| OTU_264 | 24 | 17 | 4 | 10 | 5 | 11 | 5 | 0 | 5 | 0 | 0 | 12 | All_GROUP:sk__Eukaryota(100);sknr0__Archaeplastida(100);sknr1__Chloroplastida(100);p__Chlorophyta(100);c__Chlorophyceae(100);o__Chlamydomonadales(100);g__Chlorosarcinopsis(100);s__Chlorosarcinopsis_bastropiensis(100); |
| OTU_42 | 145 | 91 | 186 | 0 | 0 | 0 | 0 | 0 | 3 | 0 | 0 | 0 | All_GROUP:sk__Eukaryota(100);sknr0__Archaeplastida(100);sknr1__Chloroplastida(100);p__Chlorophyta(100);c__Trebouxiophyceae(100);o__Microthamniales(100);g__Dictyochloropsis(100);s__Dictyochloropsis_splendida(100); |
| OTU_542 | 6 | 0 | 9 | 0 | 0 | 0 | 0 | 0 | 0 | 0 | 0 | 0 | All_GROUP:sk__Eukaryota(100);sknr0__Archaeplastida(100);sknr1__Chloroplastida(100);p__Chlorophyta(100);c__Trebouxiophyceae(100);o__Microthamniales(100);g__Elliptochloris(100);s__Elliptochloris_subsphaerica(96); |
| OTU_980 | 4 | 0 | 26 | 0 | 1 | 0 | 2 | 0 | 0 | 0 | 0 | 0 | All_GROUP:sk__Eukaryota(100);sknr0__Archaeplastida(100);sknr1__Chloroplastida(100);p__Chlorophyta(100);c__Trebouxiophyceae(97);o__Ctenocladales(95);g__Leptosira(95);s__Leptosira_terrestris(95); |
| OTU_232 | 5 | 12 | 11 | 24 | 26 | 19 | 0 | 0 | 0 | 0 | 0 | 0 | All_GROUP:sk__Eukaryota(100);sknr0__Archaeplastida(100);sknr1__Chloroplastida(100);p__Chlorophyta(100);c__Chlorophyceae(100);o__Chlamydomonadales(98);g__Protosiphon(98);s__Protosiphon_botryoides(98); |
| OTU_159 | 45 | 26 | 16 | 0 | 0 | 0 | 9 | 12 | 13 | 2 | 4 | 1 | All_GROUP:sk__Eukaryota(100);sknr0__Archaeplastida(100);sknr1__Chloroplastida(100);p__Chlorophyta(100);c__Trebouxiophyceae(100);s__uncultured_Dunaliellaceae(84); |
| OTU_81 | 5 | 42 | 71 | 40 | 9 | 9 | 13 | 0 | 11 | 42 | 9 | 23 | All_GROUP:sk__Eukaryota(100);sknr0__Archaeplastida(100);sknr1__Chloroplastida(100);p__Chlorophyta(100);c__Chlorophyceae(100);s__uncultured_Eimeriidae(93); |
| OTU_326 | 22 | 8 | 4 | 5 | 10 | 6 | 0 | 0 | 0 | 1 | 6 | 0 | All_GROUP:sk__Eukaryota(100);sknr0__Archaeplastida(100);sknr1__Chloroplastida(100);p__Chlorophyta(100);c__Chlorophyceae(100);s__uncultured_Eimeriidae(99); |
| OTU_500 | 0 | 1 | 0 | 0 | 0 | 0 | 0 | 26 | 0 | 0 | 0 | 0 | All_GROUP:sk__Eukaryota(100);sknr0__Archaeplastida(100);sknr1__Chloroplastida(100);p__Chlorophyta(100);c__Trebouxiophyceae(100);s__uncultured_Eimeriidae(100); |
| OTU_312 | 0 | 1 | 8 | 18 | 18 | 16 | 6 | 0 | 5 | 0 | 0 | 37 | All_GROUP:sk__Eukaryota(100);sknr0__Archaeplastida(100);sknr1__Chloroplastida(100);p__Chlorophyta(100);c__Chlorophyceae(86);s__uncultured_Scenedesmaceae(82); |
| OTU_272 | 2 | 0 | 7 | 17 | 10 | 10 | 1 | 0 | 17 | 0 | 0 | 0 | All_GROUP:sk__Eukaryota(100);sknr0__Archaeplastida(100);sknr1__Chloroplastida(100);p__Chlorophyta(100);c__Trebouxiophyceae(100);o__Chlorellales(92);g__Chlorella(82); |
| OTU_906 | 1 | 0 | 3 | 13 | 7 | 4 | 3 | 0 | 1 | 0 | 1 | 0 | All_GROUP:sk__Eukaryota(100);sknr0__Archaeplastida(100);sknr1__Chloroplastida(100);p__Chlorophyta(100);c__Trebouxiophyceae(100);o__Chlorellales(99);g__Chlorella(93); |
| OTU_807 | 2 | 3 | 8 | 12 | 7 | 8 | 0 | 0 | 0 | 4 | 0 | 0 | All_GROUP:sk__Eukaryota(100);sknr0__Archaeplastida(100);sknr1__Chloroplastida(100);p__Chlorophyta(100);c__Chlorophyceae(100);o__Chlamydomonadales(89);g__Chlorococcum(87); |
| OTU_1008 | 0 | 0 | 6 | 0 | 0 | 0 | 0 | 0 | 0 | 0 | 0 | 0 | All_GROUP:sk__Eukaryota(100);sknr0__Archaeplastida(100);sknr1__Chloroplastida(100);p__Chlorophyta(100);c__Chlorophyceae(100);o__Sphaeropleales(92);g__Desmodesmus(92); |
| OTU_1066 | 0 | 0 | 0 | 0 | 1 | 2 | 0 | 0 | 0 | 0 | 5 | 11 | All_GROUP:sk__Eukaryota(100);sknr0__Archaeplastida(100);sknr1__Chloroplastida(100);p__Chlorophyta(100);c__Chlorophyceae(100);o__Sphaeropleales(100);g__Pseudomuriella(100); |
| OTU_222 | 16 | 12 | 7 | 9 | 12 | 4 | 5 | 0 | 15 | 0 | 0 | 0 | All_GROUP:sk__Eukaryota(100);sknr0__Archaeplastida(100);sknr1__Chloroplastida(100);p__Chlorophyta(100);c__Chlorophyceae(100);o__Chlamydomonadales(98); |
| OTU_276 | 50 | 124 | 53 | 3 | 7 | 1 | 0 | 0 | 2 | 2 | 3 | 1 | All_GROUP:sk__Eukaryota(100);sknr0__Archaeplastida(100);sknr1__Chloroplastida(100);p__Chlorophyta(100);c__Chlorophyceae(98);o__Sphaeropleales(82); |
| OTU_382 | 54 | 11 | 3 | 0 | 0 | 0 | 8 | 11 | 7 | 1 | 2 | 3 | All_GROUP:sk__Eukaryota(100);sknr0__Archaeplastida(100);sknr1__Chloroplastida(100);p__Chlorophyta(100);c__Chlorophyceae(100);o__Sphaeropleales(84); |
| OTU_204 | 8 | 8 | 0 | 25 | 23 | 13 | 0 | 0 | 0 | 5 | 7 | 28 | All_GROUP:sk__Eukaryota(100);sknr0__Archaeplastida(100);sknr1__Chloroplastida(100);p__Chlorophyta(100);c__Chlorophyceae(100); |
| OTU_428 | 0 | 0 | 2 | 20 | 2 | 1 | 0 | 0 | 0 | 0 | 0 | 0 | All_GROUP:sk__Eukaryota(100);sknr0__Archaeplastida(100);sknr1__Chloroplastida(100);p__Chlorophyta(100);c__Chlorophyceae(97); |
| OTU_644 | 0 | 2 | 5 | 3 | 3 | 1 | 0 | 0 | 0 | 0 | 0 | 0 | All_GROUP:sk__Eukaryota(100);sknr0__Archaeplastida(100);sknr1__Chloroplastida(100);p__Chlorophyta(83);c__Chlorophyceae(82); |
| OTU_672 | 9 | 3 | 2 | 0 | 0 | 0 | 0 | 0 | 0 | 0 | 0 | 0 | All_GROUP:sk__Eukaryota(100);sknr0__Archaeplastida(100);sknr1__Chloroplastida(100);p__Chlorophyta(100);c__Chlorophyceae(95); |
| OTU_688 | 15 | 0 | 3 | 1 | 2 | 0 | 0 | 0 | 0 | 1 | 0 | 1 | All_GROUP:sk__Eukaryota(100);sknr0__Archaeplastida(100);sknr1__Chloroplastida(100);p__Chlorophyta(100);c__Chlorophyceae(100); |
| OTU_740 | 0 | 0 | 0 | 0 | 0 | 0 | 9 | 10 | 6 | 0 | 0 | 1 | All_GROUP:sk__Eukaryota(100);sknr0__Archaeplastida(100);sknr1__Chloroplastida(100);p__Chlorophyta(100);c__Chlorophyceae(99); |
| OTU_1006 | 0 | 0 | 0 | 0 | 0 | 0 | 0 | 1 | 10 | 0 | 0 | 0 | All_GROUP:sk__Eukaryota(100);sknr0__Archaeplastida(100);sknr1__Chloroplastida(100);p__Chlorophyta(99);c__Chlorophyceae(94); |
| OTU_130 | 14 | 19 | 82 | 0 | 0 | 0 | 2 | 0 | 2 | 0 | 2 | 4 | All_GROUP:sk__Eukaryota(100);sknr0__Archaeplastida(100);sknr1__Chloroplastida(100);p__Chlorophyta(100);c__Trebouxiophyceae(100); |
| OTU_216 | 2 | 26 | 33 | 0 | 0 | 0 | 0 | 0 | 0 | 0 | 0 | 0 | All_GROUP:sk__Eukaryota(100);sknr0__Archaeplastida(100);sknr1__Chloroplastida(100);p__Chlorophyta(100);c__Trebouxiophyceae(96); |
| OTU_324 | 13 | 38 | 42 | 0 | 0 | 0 | 15 | 3 | 3 | 3 | 1 | 0 | All_GROUP:sk__Eukaryota(100);sknr0__Archaeplastida(100);sknr1__Chloroplastida(100);p__Chlorophyta(99);c__Trebouxiophyceae(96); |
| OTU_162 | 19 | 3 | 10 | 17 | 28 | 16 | 8 | 0 | 3 | 1 | 0 | 4 | All_GROUP:sk__Eukaryota(100);sknr0__Archaeplastida(100);sknr1__Chloroplastida(100);p__Chlorophyta(100);c__Ulvophyceae(100); |
| OTU_862 | 1 | 1 | 11 | 0 | 0 | 0 | 0 | 14 | 0 | 1 | 3 | 2 | All_GROUP:sk__Eukaryota(100);sknr0__Archaeplastida(100);sknr1__Chloroplastida(100);p__Chlorophyta(100);c__Trebouxiophyceae(100);cnr0__Incertae_Sedis(100);g__Chloropyrula(100);s__Chloropyrula_uraliensis(100); |
| OTU_241 | 10 | 18 | 5 | 0 | 0 | 0 | 0 | 0 | 0 | 0 | 0 | 0 | All_GROUP:sk__Eukaryota(100);sknr0__Opisthokonta(95);sknr1__Nucletmycea(92);k__Fungi(92);subk__Dikarya(92);p__Ascomycota(92); |
| OTU_191 | 20 | 28 | 34 | 16 | 10 | 13 | 24 | 17 | 15 | 5 | 19 | 32 | All_GROUP:sk__Eukaryota(100);sknr0__Opisthokonta(100);sknr1__Nucletmycea(100);k__Fungi(100);subk__Dikarya(100);p__Ascomycota(100);subp__Pezizomycotina(100);c__Sordariomycetes(100);o__Hypocreales(100);f__Nectriaceae(94); |
| OTU_989 | 2 | 1 | 2 | 2 | 2 | 12 | 0 | 2 | 0 | 0 | 1 | 0 | All_GROUP:sk__Eukaryota(100);sknr0__Opisthokonta(100);sknr1__Nucletmycea(100);k__Fungi(100);subk__Dikarya(100);p__Ascomycota(100);subp__Pezizomycotina(100);c__Pezizomycetes(100);o__Pezizales(100); |
| OTU_418 | 14 | 20 | 19 | 19 | 13 | 12 | 3 | 4 | 13 | 6 | 2 | 5 | All_GROUP:sk__Eukaryota(100);sknr0__Opisthokonta(100);sknr1__Nucletmycea(100);k__Fungi(100);subk__Dikarya(100);p__Ascomycota(100);subp__Pezizomycotina(100);c__Sordariomycetes(100);o__Sordariales(100); |
| OTU_596 | 1 | 1 | 0 | 0 | 0 | 0 | 0 | 16 | 0 | 0 | 0 | 3 | All_GROUP:sk__Eukaryota(100);sknr0__Opisthokonta(100);sknr1__Nucletmycea(100);k__Fungi(100);subk__Dikarya(100);p__Ascomycota(100);subp__Pezizomycotina(100);c__Sordariomycetes(98); |
| OTU_713 | 9 | 18 | 25 | 1 | 0 | 1 | 7 | 0 | 3 | 9 | 0 | 5 | All_GROUP:sk__Eukaryota(100);sknr0__Opisthokonta(100);sknr1__Nucletmycea(100);k__Fungi(100);subk__Dikarya(100);p__Ascomycota(100);subp__Pezizomycotina(100);c__Sordariomycetes(96); |
| OTU_170 | 21 | 6 | 12 | 0 | 0 | 0 | 38 | 20 | 29 | 14 | 0 | 4 | All_GROUP:sk__Eukaryota(100);sknr0__Opisthokonta(100);sknr1__Nucletmycea(100);k__Fungi(100);subk__Dikarya(99);p__Ascomycota(99);subp__Pezizomycotina(99);subpnr0__Incertae_Sedis(99);subpnr1__Incertae_Sedis(99);subpnr2__Incertae_Sedis(99);g__Calcarisporiella(99); |
| OTU_788 | 0 | 0 | 0 | 0 | 0 | 0 | 0 | 20 | 0 | 0 | 0 | 0 | All_GROUP:sk__Eukaryota(100);sknr0__Opisthokonta(100);sknr1__Nucletmycea(100);k__Fungi(100);subk__Dikarya(100);p__Ascomycota(100);subp__Saccharomycotina(100);c__Saccharomycetes(100);o__Saccharomycetales(100);s__Candida_sp._BG02-7-21-004Q-1-2(97); |
| OTU_562 | 0 | 0 | 0 | 14 | 17 | 11 | 0 | 0 | 0 | 0 | 0 | 0 | All_GROUP:sk__Eukaryota(100);sknr0__Opisthokonta(100);sknr1__Nucletmycea(100);k__Fungi(100);subk__Dikarya(100);p__Ascomycota(100);subp__Saccharomycotina(100);c__Saccharomycetes(100);o__Saccharomycetales(100);onr0__Incertae_Sedis(100);g__Candida(100);s__Candida_intermedia(100); |
| OTU_505 | 0 | 0 | 0 | 24 | 31 | 20 | 0 | 0 | 0 | 0 | 0 | 0 | All_GROUP:sk__Eukaryota(100);sknr0__Opisthokonta(100);sknr1__Nucletmycea(100);k__Fungi(100);subk__Dikarya(100);p__Ascomycota(100);subp__Saccharomycotina(100);c__Saccharomycetes(100);o__Saccharomycetales(100);onr0__Incertae_Sedis(93);g__Candida(93);s__Candida_quercitrusa(93); |
| OTU_65 | 0 | 0 | 0 | 0 | 0 | 0 | 65 | 78 | 66 | 0 | 0 | 3 | All_GROUP:sk__Eukaryota(100);sknr0__Opisthokonta(100);sknr1__Nucletmycea(100);k__Fungi(100);subk__Dikarya(100);p__Ascomycota(100);subp__Taphrinomycotina(98);c__Archaeorhizomycetes(98);o__Archaeorhizomycetales(98);f__Archaeorhizomycetaceae(98);g__Archaeorhizomyces(98); |
| OTU_88 | 74 | 11 | 61 | 0 | 0 | 0 | 37 | 45 | 39 | 4 | 3 | 39 | All_GROUP:sk__Eukaryota(100);sknr0__Opisthokonta(100);sknr1__Nucletmycea(100);k__Fungi(100);subk__Dikarya(100);p__Ascomycota(100);subp__Taphrinomycotina(100);c__Archaeorhizomycetes(100);o__Archaeorhizomycetales(100);f__Archaeorhizomycetaceae(100);g__Archaeorhizomyces(100); |
| OTU_991 | 0 | 0 | 19 | 0 | 0 | 0 | 1 | 0 | 0 | 0 | 0 | 0 | All_GROUP:sk__Eukaryota(100);sknr0__Opisthokonta(96);sknr1__Nucletmycea(96);k__Fungi(96);subk__Dikarya(95);p__Basidiomycota(84); |
| OTU_497 | 1 | 0 | 0 | 0 | 0 | 0 | 2 | 6 | 2 | 3 | 7 | 24 | All_GROUP:sk__Eukaryota(100);sknr0__Opisthokonta(100);sknr1__Nucletmycea(100);k__Fungi(100);subk__Dikarya(100);p__Basidiomycota(100);pnr0__Agaricomycotina(100);c__Tremellomycetes(100);o__Tremellales(100);f__Tremellaceae(100);g__Bullera(100);s__Bullera_miyagiana(100); |
| OTU_1072 | 2 | 1 | 0 | 4 | 3 | 5 | 3 | 0 | 0 | 0 | 0 | 1 | All_GROUP:sk__Eukaryota(100);sknr0__Opisthokonta(100);sknr1__Nucletmycea(100);k__Fungi(100);subk__Dikarya(100);p__Basidiomycota(100);pnr0__Agaricomycotina(100);c__Tremellomycetes(100);o__Tremellales(100);f__Tremellaceae(100);g__Cryptococcus(100);s__Cryptococcus_flavescens(83); |
| OTU_55 | 0 | 0 | 0 | 0 | 1 | 0 | 78 | 87 | 93 | 7 | 4 | 193 | All_GROUP:sk__Eukaryota(100);sknr0__Opisthokonta(100);sknr1__Nucletmycea(100);k__Fungi(100);subk__Dikarya(100);p__Basidiomycota(100);pnr0__Agaricomycotina(100);c__Agaricomycetes(100);o__Geastrales(93);f__Geastraceae(93);g__Geastrum(93);s__Geastrum_saccatum(93); |
| OTU_155 | 0 | 0 | 0 | 0 | 0 | 0 | 0 | 0 | 0 | 38 | 20 | 37 | All_GROUP:sk__Eukaryota(100);sknr0__Opisthokonta(100);sknr1__Nucletmycea(100);k__Fungi(100);subk__Dikarya(100);p__Basidiomycota(100);pnr0__Agaricomycotina(100);c__Tremellomycetes(100);o__Cystofilobasidiales(100);f__Cystofilobasidiaceae(100);g__Guehomyces(95);s__Guehomyces_pullulans(95); |
| OTU_259 | 12 | 10 | 30 | 0 | 0 | 0 | 0 | 0 | 0 | 0 | 0 | 0 | All_GROUP:sk__Eukaryota(100);sknr0__Opisthokonta(100);sknr1__Nucletmycea(100);k__Fungi(100);subk__Dikarya(100);p__Basidiomycota(100);pnr0__Agaricomycotina(100);c__Agaricomycetes(100);o__Boletales(100);f__Sclerodermataceae(100);g__Scleroderma(100);s__Scleroderma_laeve(99); |
| OTU_90 | 0 | 0 | 1 | 0 | 0 | 0 | 24 | 9 | 17 | 45 | 17 | 129 | All_GROUP:sk__Eukaryota(100);sknr0__Opisthokonta(100);sknr1__Nucletmycea(100);k__Fungi(100);subk__Dikarya(100);p__Basidiomycota(100);pnr0__Agaricomycotina(100);c__Agaricomycetes(100);s__uncultured_Auriculariaceae(81); |
| OTU_118 | 2 | 0 | 2 | 2 | 1 | 2 | 0 | 0 | 0 | 12 | 10 | 48 | All_GROUP:sk__Eukaryota(100);sknr0__Opisthokonta(100);sknr1__Nucletmycea(100);k__Fungi(100);subk__Dikarya(100);p__Basidiomycota(100);pnr0__Agaricomycotina(100);c__Tremellomycetes(100);o__Tremellales(100);f__Tremellaceae(100);g__Cryptococcus(100); |
| OTU_62 | 18 | 16 | 13 | 0 | 0 | 0 | 1 | 1 | 1 | 69 | 44 | 170 | All_GROUP:sk__Eukaryota(100);sknr0__Opisthokonta(100);sknr1__Nucletmycea(100);k__Fungi(100);subk__Dikarya(100);p__Basidiomycota(100);pnr0__Agaricomycotina(100);c__Agaricomycetes(100);o__Russulales(93);f__Russulaceae(93);g__Russula(88); |
| OTU_878 | 3 | 1 | 2 | 0 | 0 | 0 | 0 | 0 | 3 | 0 | 0 | 0 | All_GROUP:sk__Eukaryota(100);sknr0__Opisthokonta(100);sknr1__Nucletmycea(100);k__Fungi(100);subk__Dikarya(100);p__Basidiomycota(100);pnr0__Agaricomycotina(100);c__Agaricomycetes(100);o__Phallales(90);f__Phallaceae(90); |
| OTU_56 | 21 | 340 | 15 | 0 | 1 | 5 | 14 | 15 | 7 | 0 | 2 | 32 | All_GROUP:sk__Eukaryota(100);sknr0__Opisthokonta(100);sknr1__Nucletmycea(100);k__Fungi(100);subk__Dikarya(100);p__Basidiomycota(100);pnr0__Agaricomycotina(100);c__Agaricomycetes(100); |
| OTU_107 | 58 | 19 | 55 | 0 | 0 | 0 | 0 | 0 | 0 | 0 | 0 | 1 | All_GROUP:sk__Eukaryota(100);sknr0__Opisthokonta(100);sknr1__Nucletmycea(100);k__Fungi(100);subk__Dikarya(100);p__Basidiomycota(100);pnr0__Agaricomycotina(100);c__Agaricomycetes(100); |
| OTU_271 | 3 | 0 | 3 | 1 | 1 | 0 | 8 | 5 | 6 | 2 | 7 | 14 | All_GROUP:sk__Eukaryota(100);sknr0__Opisthokonta(100);sknr1__Nucletmycea(100);k__Fungi(100);subk__Dikarya(100);p__Basidiomycota(100);pnr0__Agaricomycotina(100);c__Agaricomycetes(100); |
| OTU_499 | 0 | 0 | 0 | 0 | 0 | 0 | 23 | 6 | 13 | 0 | 0 | 0 | All_GROUP:sk__Eukaryota(100);sknr0__Opisthokonta(100);sknr1__Nucletmycea(100);k__Fungi(100);subk__Dikarya(100);p__Basidiomycota(100);pnr0__Agaricomycotina(100);c__Agaricomycetes(100); |
| OTU_532 | 2 | 0 | 0 | 0 | 0 | 0 | 0 | 19 | 0 | 1 | 3 | 1 | All_GROUP:sk__Eukaryota(100);sknr0__Opisthokonta(100);sknr1__Nucletmycea(100);k__Fungi(100);subk__Dikarya(100);p__Basidiomycota(100);pnr0__Agaricomycotina(100);c__Agaricomycetes(100); |
| OTU_557 | 0 | 0 | 0 | 0 | 0 | 0 | 3 | 0 | 19 | 0 | 0 | 0 | All_GROUP:sk__Eukaryota(100);sknr0__Opisthokonta(100);sknr1__Nucletmycea(100);k__Fungi(100);subk__Dikarya(100);p__Basidiomycota(100);pnr0__Agaricomycotina(100);c__Agaricomycetes(100); |
| OTU_821 | 12 | 3 | 15 | 0 | 0 | 2 | 0 | 0 | 1 | 0 | 0 | 0 | All_GROUP:sk__Eukaryota(100);sknr0__Opisthokonta(100);sknr1__Nucletmycea(100);k__Fungi(100);subk__Dikarya(100);p__Basidiomycota(100);pnr0__Agaricomycotina(100);c__Agaricomycetes(100); |
| OTU_836 | 3 | 0 | 1 | 0 | 0 | 2 | 1 | 0 | 0 | 1 | 0 | 8 | All_GROUP:sk__Eukaryota(100);sknr0__Opisthokonta(100);sknr1__Nucletmycea(100);k__Fungi(100);subk__Dikarya(100);p__Basidiomycota(100);pnr0__Agaricomycotina(100);c__Agaricomycetes(100); |
| OTU_1007 | 2 | 1 | 4 | 0 | 2 | 3 | 1 | 0 | 1 | 0 | 0 | 0 | All_GROUP:sk__Eukaryota(100);sknr0__Opisthokonta(100);sknr1__Nucletmycea(100);k__Fungi(100);subk__Dikarya(100);p__Basidiomycota(100);pnr0__Agaricomycotina(100);c__Tremellomycetes(100);o__Tremellales(100);onr0__Incertae_Sedis(95);g__Hannaella(90); |
| OTU_197 | 7 | 5 | 17 | 0 | 0 | 0 | 17 | 7 | 27 | 0 | 0 | 0 | All_GROUP:sk__Eukaryota(100);sknr0__Opisthokonta(100);sknr1__Nucletmycea(100);k__Fungi(100);subk__Dikarya(100);p__Basidiomycota(100);pnr0__Agaricomycotina(100);c__Agaricomycetes(100);o__Trechisporales(100);f__Hydnodontaceae(100);fnr0__uncultured(92);s__uncultured_soil_fungus(89); |
| OTU_280 | 0 | 0 | 0 | 0 | 0 | 0 | 38 | 24 | 53 | 2 | 0 | 0 | All_GROUP:sk__Eukaryota(100);sknr0__Opisthokonta(100);sknr1__Nucletmycea(100);k__Fungi(100);subk__Dikarya(100);p__Basidiomycota(100);subp__Pucciniomycotina(100); |
| OTU_790 | 0 | 0 | 0 | 0 | 0 | 0 | 0 | 0 | 14 | 1 | 0 | 0 | All_GROUP:sk__Eukaryota(100);sknr0__Opisthokonta(100);sknr1__Nucletmycea(100);k__Fungi(100);subk__Dikarya(100);p__Basidiomycota(100);subp__Pucciniomycotina(100);subc__Microbotryomycetes(84); |
| OTU_802 | 0 | 0 | 0 | 0 | 0 | 4 | 0 | 1 | 19 | 0 | 0 | 0 | All_GROUP:sk__Eukaryota(100);sknr0__Opisthokonta(100);sknr1__Nucletmycea(100);k__Fungi(100);p__Blastocladiomycota(99);pnr0__Incertae_Sedis(99);c__Blastocladiomycetes(99);o__Blastocladiales(99);f__Blastocladiaceae(81); |
| OTU_963 | 0 | 0 | 0 | 0 | 0 | 0 | 0 | 0 | 0 | 6 | 3 | 0 | All_GROUP:sk__Eukaryota(100);sknr0__Opisthokonta(100);sknr1__Nucletmycea(100);k__Fungi(100);p__Chytridiomycota(99);pnr0__Incertae_Sedis(99);c__Chytridiomycetes(99);o__Chytridiales(93);f__Synchytriaceae(91);g__Synchytrium(91); |
| OTU_188 | 0 | 0 | 0 | 0 | 0 | 0 | 21 | 0 | 0 | 0 | 0 | 57 | All_GROUP:sk__Eukaryota(100);sknr0__Opisthokonta(100);sknr1__Nucletmycea(100);k__Fungi(100);p__Chytridiomycota(100);pnr0__Incertae_Sedis(100);c__Chytridiomycetes(100);o__Chytridiales(94); |
| OTU_332 | 0 | 7 | 1 | 0 | 0 | 0 | 0 | 18 | 0 | 0 | 0 | 0 | All_GROUP:sk__Eukaryota(100);sknr0__Opisthokonta(100);sknr1__Nucletmycea(100);k__Fungi(100);p__Chytridiomycota(100);pnr0__Incertae_Sedis(100);c__Chytridiomycetes(100);o__Rhizophydiales(96); |
| OTU_814 | 14 | 0 | 9 | 6 | 0 | 0 | 0 | 0 | 0 | 0 | 0 | 0 | All_GROUP:sk__Eukaryota(100);sknr0__Opisthokonta(99);sknr1__Nucletmycea(99);k__Fungi(99);p__Chytridiomycota(99);pnr0__Incertae_Sedis(99);c__Chytridiomycetes(99);o__Rhizophydiales(88); |
| OTU_133 | 0 | 1 | 2 | 0 | 0 | 1 | 0 | 0 | 0 | 47 | 18 | 37 | All_GROUP:sk__Eukaryota(100);sknr0__Opisthokonta(100);sknr1__Nucletmycea(100);k__Fungi(100);p__Chytridiomycota(100);pnr0__Incertae_Sedis(100);c__Chytridiomycetes(100); |
| OTU_233 | 83 | 31 | 37 | 52 | 35 | 30 | 51 | 23 | 22 | 40 | 18 | 54 | All_GROUP:sk__Eukaryota(100);sknr0__Opisthokonta(100);sknr1__Nucletmycea(100);k__Fungi(100);p__Chytridiomycota(100);pnr0__Incertae_Sedis(100);c__Chytridiomycetes(100); |
| OTU_265 | 9 | 0 | 51 | 2 | 0 | 0 | 1 | 0 | 0 | 0 | 0 | 0 | All_GROUP:sk__Eukaryota(100);sknr0__Opisthokonta(100);sknr1__Nucletmycea(100);k__Fungi(100);p__Chytridiomycota(99);pnr0__Incertae_Sedis(99);c__Chytridiomycetes(99); |
| OTU_336 | 0 | 0 | 0 | 0 | 0 | 0 | 0 | 0 | 0 | 42 | 20 | 15 | All_GROUP:sk__Eukaryota(100);sknr0__Opisthokonta(100);sknr1__Nucletmycea(100);k__Fungi(100);p__Chytridiomycota(100);pnr0__Incertae_Sedis(100);c__Chytridiomycetes(90); |
| OTU_347 | 30 | 1 | 2 | 1 | 0 | 0 | 0 | 0 | 0 | 0 | 0 | 0 | All_GROUP:sk__Eukaryota(100);sknr0__Opisthokonta(100);sknr1__Nucletmycea(100);k__Fungi(100);p__Chytridiomycota(98);pnr0__Incertae_Sedis(98);c__Chytridiomycetes(98); |
| OTU_396 | 0 | 2 | 0 | 6 | 8 | 3 | 5 | 0 | 0 | 0 | 0 | 0 | All_GROUP:sk__Eukaryota(100);sknr0__Opisthokonta(98);sknr1__Nucletmycea(94);k__Fungi(94);p__Chytridiomycota(85);pnr0__Incertae_Sedis(85);c__Chytridiomycetes(85); |
| OTU_427 | 1 | 11 | 0 | 0 | 0 | 0 | 0 | 0 | 0 | 0 | 0 | 0 | All_GROUP:sk__Eukaryota(100);sknr0__Opisthokonta(99);sknr1__Nucletmycea(99);k__Fungi(99);p__Chytridiomycota(98);pnr0__Incertae_Sedis(98);c__Chytridiomycetes(98); |
| OTU_595 | 37 | 2 | 3 | 13 | 0 | 2 | 7 | 2 | 1 | 1 | 1 | 2 | All_GROUP:sk__Eukaryota(100);sknr0__Opisthokonta(100);sknr1__Nucletmycea(100);k__Fungi(100);p__Chytridiomycota(100);pnr0__Incertae_Sedis(100);c__Chytridiomycetes(100); |
| OTU_675 | 0 | 0 | 0 | 4 | 0 | 0 | 0 | 0 | 4 | 0 | 0 | 0 | All_GROUP:sk__Eukaryota(100);sknr0__Opisthokonta(100);sknr1__Nucletmycea(99);k__Fungi(99);p__Chytridiomycota(99);pnr0__Incertae_Sedis(99);c__Chytridiomycetes(99); |
| OTU_720 | 11 | 0 | 0 | 0 | 0 | 0 | 0 | 0 | 0 | 0 | 0 | 0 | All_GROUP:sk__Eukaryota(100);sknr0__Opisthokonta(94);sknr1__Nucletmycea(91);k__Fungi(91);p__Chytridiomycota(82);pnr0__Incertae_Sedis(82);c__Chytridiomycetes(82); |
| OTU_856 | 0 | 0 | 1 | 0 | 0 | 7 | 0 | 0 | 0 | 0 | 0 | 0 | All_GROUP:sk__Eukaryota(100);sknr0__Opisthokonta(100);sknr1__Nucletmycea(100);k__Fungi(100);p__Chytridiomycota(100);pnr0__Incertae_Sedis(100);c__Chytridiomycetes(100); |
| OTU_920 | 0 | 1 | 1 | 8 | 6 | 9 | 0 | 0 | 0 | 1 | 0 | 0 | All_GROUP:sk__Eukaryota(100);sknr0__Opisthokonta(100);sknr1__Nucletmycea(100);k__Fungi(100);p__Chytridiomycota(100);pnr0__Incertae_Sedis(100);c__Chytridiomycetes(100); |
| OTU_972 | 2 | 28 | 2 | 0 | 0 | 0 | 0 | 0 | 0 | 0 | 0 | 0 | All_GROUP:sk__Eukaryota(100);sknr0__Opisthokonta(100);sknr1__Nucletmycea(99);k__Fungi(99);p__Chytridiomycota(98);pnr0__Incertae_Sedis(98);c__Chytridiomycetes(98); |
| OTU_1025 | 1 | 2 | 9 | 6 | 1 | 3 | 0 | 0 | 0 | 0 | 0 | 0 | All_GROUP:sk__Eukaryota(100);sknr0__Opisthokonta(100);sknr1__Nucletmycea(100);k__Fungi(100);p__Chytridiomycota(100);pnr0__Incertae_Sedis(100);c__Chytridiomycetes(100); |
| OTU_1035 | 0 | 9 | 0 | 0 | 0 | 0 | 2 | 0 | 0 | 0 | 0 | 0 | All_GROUP:sk__Eukaryota(100);sknr0__Opisthokonta(99);sknr1__Nucletmycea(97);k__Fungi(97);p__Chytridiomycota(96);pnr0__Incertae_Sedis(96);c__Chytridiomycetes(96); |
| OTU_387 | 14 | 4 | 6 | 0 | 0 | 0 | 2 | 1 | 0 | 0 | 0 | 0 | All_GROUP:sk__Eukaryota(100);sknr0__Opisthokonta(100);sknr1__Nucletmycea(100);k__Fungi(100);p__Cryptomycota(100);pnr0__Incertae_Sedis(99);pnr1__Incertae_Sedis(99);pnr2__Incertae_Sedis(99);pnr3__Incertae_Sedis(99);pnr4__Paramicrosporidium(99); |
| OTU_401 | 0 | 0 | 0 | 0 | 0 | 0 | 0 | 53 | 0 | 0 | 0 | 0 | All_GROUP:sk__Eukaryota(100);sknr0__Opisthokonta(100);sknr1__Nucletmycea(99);k__Fungi(99);p__Cryptomycota(96);pnr0__Incertae_Sedis(96);pnr1__Incertae_Sedis(96);pnr2__Incertae_Sedis(96);pnr3__Incertae_Sedis(96);pnr4__Paramicrosporidium(96); |
| OTU_404 | 0 | 0 | 0 | 0 | 0 | 0 | 14 | 0 | 6 | 3 | 3 | 17 | All_GROUP:sk__Eukaryota(100);sknr0__Opisthokonta(100);sknr1__Nucletmycea(100);k__Fungi(100);p__Cryptomycota(100);pnr0__Incertae_Sedis(100);pnr1__Incertae_Sedis(100);pnr2__Incertae_Sedis(100);pnr3__Incertae_Sedis(100);pnr4__Paramicrosporidium(100); |
| OTU_741 | 0 | 0 | 10 | 0 | 0 | 0 | 0 | 0 | 0 | 0 | 0 | 0 | All_GROUP:sk__Eukaryota(100);sknr0__Opisthokonta(98);sknr1__Nucletmycea(98);k__Fungi(98);p__Cryptomycota(92);pnr0__Incertae_Sedis(92);pnr1__Incertae_Sedis(92);pnr2__Incertae_Sedis(92);pnr3__Incertae_Sedis(92);pnr4__Paramicrosporidium(92); |
| OTU_1079 | 0 | 0 | 0 | 0 | 0 | 0 | 0 | 0 | 0 | 5 | 1 | 3 | All_GROUP:sk__Eukaryota(100);sknr0__Opisthokonta(100);sknr1__Nucletmycea(99);k__Fungi(99);p__Cryptomycota(99);pnr0__Incertae_Sedis(97);pnr1__Incertae_Sedis(97);pnr2__Incertae_Sedis(97);pnr3__Incertae_Sedis(97);pnr4__Paramicrosporidium(97); |
| OTU_565 | 0 | 0 | 0 | 0 | 21 | 5 | 0 | 0 | 0 | 0 | 0 | 0 | All_GROUP:sk__Eukaryota(100);sknr0__Opisthokonta(100);sknr1__Nucletmycea(100);k__Fungi(100);p__Cryptomycota(99);pnr0__Incertae_Sedis(99);pnr1__Incertae_Sedis(99);pnr2__Incertae_Sedis(99);pnr3__Incertae_Sedis(99);g__Rozella(99);gun0__uncultured_fungus(99); |
| OTU_746 | 0 | 0 | 0 | 0 | 0 | 0 | 4 | 0 | 2 | 0 | 0 | 0 | All_GROUP:sk__Eukaryota(100);sknr0__Opisthokonta(100);sknr1__Nucletmycea(100);k__Fungi(100);p__Cryptomycota(100);pnr0__LKM11(100);s__uncultured_Cryptomycota(93); |
| OTU_626 | 0 | 2 | 0 | 0 | 0 | 0 | 1 | 0 | 0 | 0 | 1 | 0 | All_GROUP:sk__Eukaryota(100);sknr0__Opisthokonta(100);sknr1__Nucletmycea(100);k__Fungi(100);p__Cryptomycota(100);pnr0__LKM11(100);s__uncultured_rhizosphere_zygomycete(100); |
| OTU_22 | 0 | 0 | 13 | 652 | 498 | 589 | 569 | 748 | 927 | 30 | 2 | 18 | All_GROUP:sk__Eukaryota(100);sknr0__Opisthokonta(91);sknr1__Nucletmycea(88);k__Fungi(88);p__Cryptomycota(85);pnr0__LKM11(84); |
| OTU_568 | 0 | 0 | 0 | 0 | 2 | 0 | 0 | 0 | 0 | 0 | 1 | 0 | All_GROUP:sk__Eukaryota(100);sknr0__Opisthokonta(100);sknr1__Nucletmycea(100);k__Fungi(100);p__Cryptomycota(100);pnr0__LKM11(100); |
| OTU_576 | 18 | 3 | 6 | 0 | 0 | 0 | 2 | 0 | 0 | 0 | 2 | 0 | All_GROUP:sk__Eukaryota(100);sknr0__Opisthokonta(100);sknr1__Nucletmycea(100);k__Fungi(100);p__Cryptomycota(100);pnr0__LKM11(100); |
| OTU_853 | 0 | 0 | 0 | 7 | 0 | 0 | 0 | 0 | 0 | 0 | 0 | 0 | All_GROUP:sk__Eukaryota(100);sknr0__Opisthokonta(100);sknr1__Nucletmycea(100);k__Fungi(100);p__Cryptomycota(100);pnr0__LKM11(100);pun1__uncultured_eukaryote(87); |
| OTU_114 | 55 | 28 | 49 | 0 | 0 | 0 | 8 | 29 | 28 | 229 | 44 | 55 | All_GROUP:sk__Eukaryota(100);sknr0__Opisthokonta(97);sknr1__Nucletmycea(88);k__Fungi(88); |
| OTU_144 | 0 | 0 | 0 | 0 | 0 | 0 | 26 | 23 | 52 | 0 | 0 | 0 | All_GROUP:sk__Eukaryota(100);sknr0__Opisthokonta(90);sknr1__Nucletmycea(85);k__Fungi(85); |
| OTU_385 | 10 | 15 | 1 | 0 | 0 | 1 | 0 | 0 | 0 | 0 | 0 | 0 | All_GROUP:sk__Eukaryota(100);sknr0__Opisthokonta(90);sknr1__Nucletmycea(90);k__Fungi(90); |
| OTU_456 | 6 | 1 | 2 | 0 | 0 | 0 | 0 | 0 | 0 | 0 | 2 | 37 | All_GROUP:sk__Eukaryota(100);sknr0__Opisthokonta(97);sknr1__Nucletmycea(89);k__Fungi(89); |
| OTU_625 | 0 | 0 | 0 | 4 | 3 | 0 | 0 | 0 | 0 | 0 | 4 | 4 | All_GROUP:sk__Eukaryota(100);sknr0__Opisthokonta(95);sknr1__Nucletmycea(88);k__Fungi(87); |
| OTU_635 | 0 | 0 | 0 | 0 | 0 | 0 | 0 | 1 | 5 | 0 | 0 | 0 | All_GROUP:sk__Eukaryota(100);sknr0__Opisthokonta(87);sknr1__Nucletmycea(82);k__Fungi(82); |
| OTU_661 | 18 | 3 | 3 | 0 | 0 | 0 | 11 | 3 | 11 | 0 | 0 | 0 | All_GROUP:sk__Eukaryota(100);sknr0__Opisthokonta(88);sknr1__Nucletmycea(85);k__Fungi(85); |
| OTU_736 | 16 | 18 | 11 | 0 | 0 | 2 | 0 | 0 | 0 | 0 | 0 | 0 | All_GROUP:sk__Eukaryota(100);sknr0__Opisthokonta(92);sknr1__Nucletmycea(86);k__Fungi(86); |
| OTU_761 | 0 | 0 | 0 | 0 | 0 | 0 | 0 | 0 | 3 | 0 | 0 | 0 | All_GROUP:sk__Eukaryota(100);sknr0__Opisthokonta(93);sknr1__Nucletmycea(90);k__Fungi(90); |
| OTU_773 | 8 | 0 | 3 | 0 | 0 | 0 | 17 | 5 | 4 | 11 | 4 | 2 | All_GROUP:sk__Eukaryota(100);sknr0__Opisthokonta(94);sknr1__Nucletmycea(93);k__Fungi(92); |
| OTU_835 | 2 | 0 | 0 | 0 | 0 | 0 | 0 | 0 | 0 | 1 | 0 | 0 | All_GROUP:sk__Eukaryota(100);sknr0__Opisthokonta(90);sknr1__Nucletmycea(86);k__Fungi(86); |
| OTU_871 | 0 | 0 | 0 | 3 | 0 | 0 | 0 | 0 | 0 | 0 | 0 | 0 | All_GROUP:sk__Eukaryota(100);sknr0__Opisthokonta(94);sknr1__Nucletmycea(93);k__Fungi(90); |
| OTU_1026 | 0 | 24 | 0 | 0 | 0 | 0 | 0 | 0 | 0 | 0 | 0 | 0 | All_GROUP:sk__Eukaryota(100);sknr0__Opisthokonta(97);sknr1__Nucletmycea(80);k__Fungi(80); |
| OTU_1068 | 1 | 2 | 1 | 0 | 0 | 0 | 1 | 0 | 0 | 6 | 2 | 0 | All_GROUP:sk__Eukaryota(100);sknr0__Opisthokonta(92);sknr1__Nucletmycea(89);k__Fungi(84); |
| OTU_778 | 3 | 0 | 0 | 0 | 0 | 0 | 1 | 0 | 1 | 6 | 4 | 9 | All_GROUP:sk__Eukaryota(100);sknr0__Opisthokonta(99);sknr1__Nucletmycea(99);k__Fungi(99);subk__Dikarya(97); |
| OTU_888 | 6 | 0 | 0 | 0 | 0 | 0 | 0 | 10 | 5 | 0 | 0 | 0 | All_GROUP:sk__Eukaryota(100);sknr0__Opisthokonta(100);sknr1__Nucletmycea(98);k__Fungi(98);subk__Dikarya(98); |
| OTU_719 | 0 | 0 | 0 | 0 | 0 | 0 | 3 | 0 | 18 | 15 | 1 | 0 | All_GROUP:sk__Eukaryota(100);sknr0__Opisthokonta(99);sknr1__Nucletmycea(99);k__Fungi(99);knr0__Incertae_Sedis(99);subp__Kickxellomycotina(99);subpnr0__Incertae_Sedis(99);o__Kickxellales(99);f__Kickxellaceae(99);g__Martensiomyces(88);s__Martensiomyces_pterosporus(88); |
| OTU_279 | 14 | 27 | 4 | 0 | 1 | 0 | 9 | 5 | 43 | 12 | 9 | 32 | All_GROUP:sk__Eukaryota(100);sknr0__Opisthokonta(100);sknr1__Nucletmycea(100);k__Fungi(100);knr0__Incertae_Sedis(100);subp__Kickxellomycotina(100);subpnr0__Incertae_Sedis(100);o__Kickxellales(100);f__Kickxellaceae(100);g__Ramicandelaber(100); |
| OTU_809 | 0 | 10 | 0 | 0 | 0 | 0 | 0 | 0 | 0 | 0 | 0 | 0 | All_GROUP:sk__Eukaryota(100);sknr0__Opisthokonta(100);sknr1__Nucletmycea(100);k__Fungi(100);knr0__Incertae_Sedis(100);subp__Kickxellomycotina(100);subpnr0__Incertae_Sedis(100);o__Kickxellales(100);f__Kickxellaceae(100); |
| OTU_125 | 26 | 37 | 46 | 6 | 1 | 1 | 2 | 4 | 3 | 0 | 0 | 0 | All_GROUP:sk__Eukaryota(100);sknr0__Opisthokonta(98);sknr1__Nucletmycea(98);k__Fungi(98);knr0__Incertae_Sedis(98);subp__Mucoromycotina(98);subpnr0__Incertae_Sedis(98);o__Mortierellales(98);s__Mortierella_sp._MS-6(92); |
| OTU_1080 | 1 | 2 | 3 | 3 | 1 | 0 | 16 | 0 | 0 | 0 | 0 | 0 | All_GROUP:sk__Eukaryota(100);sknr0__Opisthokonta(100);sknr1__Nucletmycea(100);k__Fungi(100);knr0__Incertae_Sedis(100);subp__Mucoromycotina(100);subpnr0__Incertae_Sedis(100);o__Mortierellales(100);s__Mortierella_sp._MS-6(100); |
| OTU_803 | 0 | 0 | 0 | 0 | 0 | 5 | 19 | 1 | 19 | 0 | 0 | 0 | All_GROUP:sk__Eukaryota(100);sknr0__Opisthokonta(100);sknr1__Nucletmycea(100);k__Fungi(100);knr0__Incertae_Sedis(100);subp__Mucoromycotina(100);subpnr0__Incertae_Sedis(100);o__Mortierellales(100);s__uncultured_Eimeriidae(91); |
| OTU_650 | 5 | 12 | 3 | 0 | 0 | 0 | 0 | 0 | 0 | 0 | 0 | 0 | All_GROUP:sk__Eukaryota(100);sknr0__Opisthokonta(100);sknr1__Nucletmycea(100);k__Fungi(100);knr0__Incertae_Sedis(88);subp__Mucoromycotina(88);subpnr0__Incertae_Sedis(88);o__Endogonales(87); |
| OTU_19 | 123 | 126 | 153 | 90 | 150 | 156 | 301 | 292 | 225 | 104 | 95 | 465 | All_GROUP:sk__Eukaryota(100);sknr0__Opisthokonta(100);sknr1__Nucletmycea(100);k__Fungi(100);knr0__Incertae_Sedis(100);subp__Mucoromycotina(100);subpnr0__Incertae_Sedis(100);o__Mortierellales(100); |
| OTU_228 | 15 | 7 | 4 | 15 | 12 | 17 | 11 | 6 | 0 | 7 | 1 | 59 | All_GROUP:sk__Eukaryota(100);sknr0__Opisthokonta(86);sknr1__Nucletmycea(86);k__Fungi(86);knr0__Incertae_Sedis(86);subp__Mucoromycotina(86);subpnr0__Incertae_Sedis(86);o__Mortierellales(86); |
| OTU_386 | 37 | 11 | 6 | 7 | 8 | 9 | 2 | 1 | 1 | 1 | 0 | 0 | All_GROUP:sk__Eukaryota(100);sknr0__Opisthokonta(98);sknr1__Nucletmycea(98);k__Fungi(98);knr0__Incertae_Sedis(98);subp__Mucoromycotina(98);subpnr0__Incertae_Sedis(98);o__Mortierellales(98); |
| OTU_207 | 16 | 15 | 20 | 7 | 12 | 10 | 44 | 34 | 29 | 15 | 2 | 69 | All_GROUP:sk__Eukaryota(100);sknr0__Opisthokonta(99);sknr1__Nucletmycea(99);k__Fungi(99);knr0__Incertae_Sedis(99);subp__Mucoromycotina(99);subpnr0__Incertae_Sedis(99);o__Mortierellales(99);oun0__uncultured_fungus(90); |
| OTU_852 | 11 | 0 | 0 | 0 | 0 | 0 | 0 | 0 | 0 | 0 | 0 | 0 | All_GROUP:sk__Eukaryota(100);sknr0__Opisthokonta(100);sknr1__Nucletmycea(100);k__Fungi(100);knr0__Incertae_Sedis(100);subp__Zoopagomycotina(100);subpnr0__Incertae_Sedis(100);o__Zoopagales(100);s__uncultured_Eimeriidae(88); |
| OTU_455 | 3 | 20 | 6 | 0 | 0 | 0 | 0 | 3 | 4 | 0 | 0 | 4 | All_GROUP:sk__Eukaryota(100);sknr0__Opisthokonta(100);sknr1__Nucletmycea(100);k__Fungi(100);knr0__LKM15(100); |
| OTU_994 | 0 | 0 | 14 | 0 | 0 | 0 | 0 | 0 | 0 | 0 | 0 | 8 | All_GROUP:sk__Eukaryota(100);sknr0__Opisthokonta(100);sknr1__Nucletmycea(100);k__Fungi(100);knr0__LKM15(99);kun1__uncultured_eukaryote(93); |
| OTU_1094 | 0 | 14 | 8 | 1 | 0 | 0 | 0 | 0 | 0 | 0 | 0 | 0 | All_GROUP:sk__Eukaryota(100);sknr0__Opisthokonta(100);sknr1__Nucletmycea(100);k__Fungi(100);knr0__LKM15(100);kun1__uncultured_eukaryote(94); |
| OTU_518 | 5 | 1 | 16 | 0 | 0 | 0 | 0 | 0 | 0 | 0 | 0 | 0 | All_GROUP:sk__Eukaryota(100);sknr0__Opisthokonta(100);sknr1__Nucletmycea(100);k__Fungi(100);knr0__LKM15(100);kun1__uncultured_fungus(86); |
| OTU_797 | 0 | 0 | 0 | 0 | 0 | 10 | 0 | 0 | 0 | 0 | 0 | 0 | All_GROUP:sk__Eukaryota(100);sknr0__Opisthokonta(100);sknr1__Nucletmycea(100);k__Fungi(100);knr0__LKM15(100);kun1__uncultured_fungus(93); |
| OTU_755 | 1 | 7 | 3 | 0 | 0 | 2 | 0 | 0 | 0 | 10 | 3 | 6 | All_GROUP:sk__Eukaryota(100);sknr0__Opisthokonta(100);sknr1__Nucletmycea(100);k__Fungi(100);p__Glomeromycota(100);pnr0__Incertae_Sedis(100);c__Glomeromycetes(100);o__Glomerales(100);f__Glomeraceae(88);g__Glomus(87);s__Claroideoglomus_etunicatum(87); |
| OTU_952 | 0 | 0 | 0 | 0 | 0 | 0 | 29 | 3 | 1 | 1 | 0 | 0 | All_GROUP:sk__Eukaryota(100);sknr0__Opisthokonta(100);sknr1__Nucletmycea(100);k__Fungi(100);p__Glomeromycota(100);pnr0__Incertae_Sedis(100);c__Glomeromycetes(100);o__Diversisporales(100);s__Diversispora_sp._W2423(97); |
| OTU_1014 | 16 | 0 | 0 | 0 | 3 | 0 | 0 | 0 | 0 | 0 | 0 | 0 | All_GROUP:sk__Eukaryota(100);sknr0__Opisthokonta(100);sknr1__Nucletmycea(100);k__Fungi(100);p__Glomeromycota(100);pnr0__Incertae_Sedis(100);c__Glomeromycetes(100);o__Glomerales(100);f__Glomeraceae(100);g__Funneliformis(100);s__Funneliformis_mosseae(96); |
| OTU_77 | 4 | 20 | 12 | 2 | 1 | 1 | 16 | 26 | 34 | 20 | 17 | 222 | All_GROUP:sk__Eukaryota(100);sknr0__Opisthokonta(100);sknr1__Nucletmycea(100);k__Fungi(100);p__Glomeromycota(100);pnr0__Incertae_Sedis(100);c__Glomeromycetes(100);o__Glomerales(100);f__Glomeraceae(97);g__Rhizophagus(97);s__Rhizophagus_intraradices(86); |
| OTU_677 | 0 | 0 | 2 | 0 | 0 | 0 | 4 | 5 | 9 | 1 | 7 | 12 | All_GROUP:sk__Eukaryota(100);sknr0__Opisthokonta(100);sknr1__Nucletmycea(100);k__Fungi(100);p__Glomeromycota(100);pnr0__Incertae_Sedis(100);c__Glomeromycetes(100);o__Glomerales(100);s__uncultured_Glomus(93); |
| OTU_196 | 6 | 13 | 1 | 0 | 0 | 0 | 17 | 43 | 30 | 38 | 6 | 7 | All_GROUP:sk__Eukaryota(100);sknr0__Opisthokonta(100);sknr1__Nucletmycea(100);k__Fungi(100);p__Glomeromycota(100);pnr0__Incertae_Sedis(100);c__Glomeromycetes(100);o__Glomerales(100);s__uncultured_mycorrhizal_fungus(98); |
| OTU_656 | 3 | 14 | 14 | 0 | 0 | 0 | 0 | 0 | 0 | 0 | 0 | 4 | All_GROUP:sk__Eukaryota(100);sknr0__Opisthokonta(100);sknr1__Nucletmycea(100);k__Fungi(100);p__Glomeromycota(100);pnr0__Incertae_Sedis(100);c__Glomeromycetes(100);o__Glomerales(100);s__uncultured_mycorrhizal_fungus(94); |
| OTU_795 | 0 | 15 | 4 | 0 | 2 | 0 | 5 | 0 | 3 | 0 | 0 | 0 | All_GROUP:sk__Eukaryota(100);sknr0__Opisthokonta(100);sknr1__Nucletmycea(100);k__Fungi(100);p__Glomeromycota(100);pnr0__Incertae_Sedis(100);c__Glomeromycetes(100);o__Glomerales(100);s__uncultured_mycorrhizal_fungus(100); |
| OTU_824 | 13 | 4 | 6 | 1 | 0 | 1 | 11 | 2 | 7 | 0 | 0 | 1 | All_GROUP:sk__Eukaryota(100);sknr0__Opisthokonta(100);sknr1__Nucletmycea(100);k__Fungi(100);p__Glomeromycota(100);pnr0__Incertae_Sedis(100);c__Glomeromycetes(100);o__Glomerales(100);s__uncultured_mycorrhizal_fungus(93); |
| OTU_478 | 51 | 13 | 0 | 2 | 0 | 1 | 1 | 0 | 3 | 0 | 0 | 7 | All_GROUP:sk__Eukaryota(100);sknr0__Opisthokonta(100);sknr1__Nucletmycea(100);k__Fungi(100);p__Glomeromycota(100);pnr0__Incertae_Sedis(100);c__Glomeromycetes(100);o__Glomerales(99);f__Glomeraceae(91); |
| OTU_112 | 29 | 30 | 39 | 10 | 8 | 2 | 41 | 25 | 51 | 1 | 3 | 4 | All_GROUP:sk__Eukaryota(100);sknr0__Opisthokonta(100);sknr1__Nucletmycea(100);k__Fungi(100);p__Glomeromycota(100);pnr0__Incertae_Sedis(100);c__Glomeromycetes(100);o__Glomerales(100); |
| OTU_194 | 18 | 0 | 5 | 0 | 0 | 0 | 30 | 26 | 55 | 21 | 13 | 66 | All_GROUP:sk__Eukaryota(100);sknr0__Opisthokonta(100);sknr1__Nucletmycea(100);k__Fungi(100);p__Glomeromycota(100);pnr0__Incertae_Sedis(100);c__Glomeromycetes(100);o__Glomerales(100); |
| OTU_201 | 28 | 27 | 60 | 0 | 2 | 2 | 12 | 10 | 9 | 2 | 0 | 4 | All_GROUP:sk__Eukaryota(100);sknr0__Opisthokonta(100);sknr1__Nucletmycea(100);k__Fungi(100);p__Glomeromycota(100);pnr0__Incertae_Sedis(100);c__Glomeromycetes(100);o__Glomerales(100); |
| OTU_286 | 23 | 22 | 21 | 1 | 1 | 0 | 20 | 8 | 26 | 7 | 7 | 12 | All_GROUP:sk__Eukaryota(100);sknr0__Opisthokonta(100);sknr1__Nucletmycea(100);k__Fungi(100);p__Glomeromycota(100);pnr0__Incertae_Sedis(100);c__Glomeromycetes(100);o__Glomerales(100); |
| OTU_297 | 59 | 1 | 0 | 0 | 0 | 0 | 2 | 0 | 2 | 0 | 0 | 0 | All_GROUP:sk__Eukaryota(100);sknr0__Opisthokonta(100);sknr1__Nucletmycea(100);k__Fungi(100);p__Glomeromycota(100);pnr0__Incertae_Sedis(100);c__Glomeromycetes(100);o__Glomerales(100); |
| OTU_300 | 38 | 20 | 16 | 0 | 0 | 0 | 13 | 2 | 24 | 1 | 0 | 5 | All_GROUP:sk__Eukaryota(100);sknr0__Opisthokonta(100);sknr1__Nucletmycea(100);k__Fungi(100);p__Glomeromycota(100);pnr0__Incertae_Sedis(100);c__Glomeromycetes(100);o__Glomerales(100); |
| OTU_330 | 53 | 18 | 28 | 0 | 0 | 0 | 1 | 0 | 1 | 0 | 2 | 4 | All_GROUP:sk__Eukaryota(100);sknr0__Opisthokonta(100);sknr1__Nucletmycea(100);k__Fungi(100);p__Glomeromycota(100);pnr0__Incertae_Sedis(100);c__Glomeromycetes(100);o__Glomerales(100); |
| OTU_409 | 2 | 0 | 0 | 0 | 0 | 0 | 2 | 3 | 23 | 3 | 5 | 9 | All_GROUP:sk__Eukaryota(100);sknr0__Opisthokonta(100);sknr1__Nucletmycea(100);k__Fungi(100);p__Glomeromycota(100);pnr0__Incertae_Sedis(100);c__Glomeromycetes(100);o__Glomerales(100); |
| OTU_444 | 21 | 21 | 15 | 6 | 4 | 11 | 9 | 6 | 5 | 9 | 1 | 1 | All_GROUP:sk__Eukaryota(100);sknr0__Opisthokonta(100);sknr1__Nucletmycea(100);k__Fungi(100);p__Glomeromycota(99);pnr0__Incertae_Sedis(99);c__Glomeromycetes(99);o__Glomerales(99); |
| OTU_534 | 26 | 9 | 8 | 0 | 0 | 0 | 0 | 0 | 0 | 0 | 0 | 0 | All_GROUP:sk__Eukaryota(100);sknr0__Opisthokonta(100);sknr1__Nucletmycea(100);k__Fungi(100);p__Glomeromycota(100);pnr0__Incertae_Sedis(100);c__Glomeromycetes(100);o__Glomerales(100); |
| OTU_648 | 7 | 1 | 9 | 0 | 0 | 0 | 0 | 2 | 1 | 0 | 0 | 0 | All_GROUP:sk__Eukaryota(100);sknr0__Opisthokonta(100);sknr1__Nucletmycea(100);k__Fungi(100);p__Glomeromycota(100);pnr0__Incertae_Sedis(100);c__Glomeromycetes(100);o__Glomerales(100); |
| OTU_737 | 2 | 8 | 12 | 0 | 0 | 0 | 2 | 5 | 3 | 0 | 0 | 0 | All_GROUP:sk__Eukaryota(100);sknr0__Opisthokonta(100);sknr1__Nucletmycea(100);k__Fungi(100);p__Glomeromycota(100);pnr0__Incertae_Sedis(100);c__Glomeromycetes(100);o__Glomerales(100); |
| OTU_760 | 0 | 0 | 0 | 2 | 2 | 1 | 19 | 6 | 9 | 7 | 4 | 12 | All_GROUP:sk__Eukaryota(100);sknr0__Opisthokonta(100);sknr1__Nucletmycea(100);k__Fungi(100);p__Glomeromycota(100);pnr0__Incertae_Sedis(100);c__Glomeromycetes(100);o__Glomerales(100); |
| OTU_791 | 10 | 0 | 3 | 0 | 0 | 0 | 3 | 0 | 2 | 0 | 0 | 5 | All_GROUP:sk__Eukaryota(100);sknr0__Opisthokonta(100);sknr1__Nucletmycea(100);k__Fungi(100);p__Glomeromycota(100);pnr0__Incertae_Sedis(100);c__Glomeromycetes(100);o__Glomerales(100); |
| OTU_848 | 0 | 0 | 0 | 0 | 0 | 0 | 8 | 10 | 21 | 0 | 0 | 0 | All_GROUP:sk__Eukaryota(100);sknr0__Opisthokonta(100);sknr1__Nucletmycea(100);k__Fungi(100);p__Glomeromycota(100);pnr0__Incertae_Sedis(100);c__Glomeromycetes(100);o__Glomerales(100); |
| OTU_1004 | 0 | 8 | 4 | 0 | 0 | 0 | 0 | 0 | 0 | 0 | 0 | 0 | All_GROUP:sk__Eukaryota(100);sknr0__Opisthokonta(100);sknr1__Nucletmycea(100);k__Fungi(100);p__Glomeromycota(100);pnr0__Incertae_Sedis(100);c__Glomeromycetes(100);o__Glomerales(100); |
| OTU_1092 | 10 | 10 | 4 | 0 | 0 | 0 | 1 | 0 | 5 | 7 | 1 | 1 | All_GROUP:sk__Eukaryota(100);sknr0__Opisthokonta(100);sknr1__Nucletmycea(100);k__Fungi(100);p__Glomeromycota(100);pnr0__Incertae_Sedis(100);c__Glomeromycetes(100);o__Glomerales(100); |
| OTU_61 | 0 | 0 | 0 | 337 | 57 | 1 | 0 | 0 | 0 | 0 | 0 | 0 | All_GROUP:sk__Eukaryota(100);sknr0__Opisthokonta(100);sknr1__Holozoa(100);sknr2__Metazoa_(Animalia)(100);sknr3__Eumetazoa(100);sknr4__Bilateria(100);p__Arthropoda(100); |
| OTU_679 | 0 | 0 | 0 | 0 | 0 | 0 | 0 | 0 | 0 | 14 | 0 | 0 | All_GROUP:sk__Eukaryota(100);sknr0__Opisthokonta(100);sknr1__Holozoa(100);sknr2__Metazoa_(Animalia)(100);sknr3__Eumetazoa(100);sknr4__Bilateria(100);p__Arthropoda(100);subp__Chelicerata(100);c__Arachnida(100);o__Araneae(98); |
| OTU_126 | 0 | 0 | 277 | 0 | 0 | 0 | 7 | 7 | 4 | 0 | 0 | 0 | All_GROUP:sk__Eukaryota(100);sknr0__Opisthokonta(100);sknr1__Holozoa(100);sknr2__Metazoa_(Animalia)(100);sknr3__Eumetazoa(100);sknr4__Bilateria(100);p__Arthropoda(98);subp__Chelicerata(93);c__Arachnida(84); |
| OTU_304 | 0 | 0 | 0 | 0 | 0 | 0 | 8 | 20 | 19 | 0 | 0 | 0 | All_GROUP:sk__Eukaryota(100);sknr0__Opisthokonta(99);sknr1__Holozoa(99);sknr2__Metazoa_(Animalia)(99);sknr3__Eumetazoa(99);sknr4__Bilateria(98);p__Arthropoda(89);subp__Chelicerata(82);c__Arachnida(82); |
| OTU_356 | 53 | 1 | 1 | 0 | 0 | 0 | 0 | 0 | 0 | 0 | 0 | 0 | All_GROUP:sk__Eukaryota(100);sknr0__Opisthokonta(100);sknr1__Holozoa(100);sknr2__Metazoa_(Animalia)(100);sknr3__Eumetazoa(100);sknr4__Bilateria(100);p__Arthropoda(96);subp__Chelicerata(88);c__Arachnida(86); |
| OTU_399 | 0 | 0 | 0 | 0 | 0 | 0 | 4 | 16 | 6 | 0 | 0 | 0 | All_GROUP:sk__Eukaryota(100);sknr0__Opisthokonta(100);sknr1__Holozoa(100);sknr2__Metazoa_(Animalia)(100);sknr3__Eumetazoa(100);sknr4__Bilateria(100);p__Arthropoda(99);subp__Chelicerata(87);c__Arachnida(87); |
| OTU_566 | 1 | 7 | 26 | 0 | 0 | 0 | 0 | 0 | 0 | 0 | 0 | 0 | All_GROUP:sk__Eukaryota(100);sknr0__Opisthokonta(100);sknr1__Holozoa(100);sknr2__Metazoa_(Animalia)(100);sknr3__Eumetazoa(100);sknr4__Bilateria(100);p__Arthropoda(88);subp__Chelicerata(81);c__Arachnida(81); |
| OTU_666 | 0 | 0 | 25 | 0 | 0 | 0 | 1 | 0 | 0 | 0 | 0 | 0 | All_GROUP:sk__Eukaryota(100);sknr0__Opisthokonta(100);sknr1__Holozoa(100);sknr2__Metazoa_(Animalia)(100);sknr3__Eumetazoa(100);sknr4__Bilateria(100);p__Arthropoda(94);subp__Chelicerata(85);c__Arachnida(84); |
| OTU_786 | 0 | 0 | 0 | 1 | 3 | 0 | 0 | 0 | 0 | 2 | 0 | 0 | All_GROUP:sk__Eukaryota(100);sknr0__Opisthokonta(100);sknr1__Holozoa(100);sknr2__Metazoa_(Animalia)(100);sknr3__Eumetazoa(100);sknr4__Bilateria(100);p__Arthropoda(94);subp__Chelicerata(92);c__Arachnida(92); |
| OTU_892 | 0 | 0 | 1 | 0 | 0 | 0 | 1 | 0 | 9 | 0 | 0 | 0 | All_GROUP:sk__Eukaryota(100);sknr0__Opisthokonta(100);sknr1__Holozoa(100);sknr2__Metazoa_(Animalia)(100);sknr3__Eumetazoa(100);sknr4__Bilateria(100);p__Arthropoda(99);subp__Chelicerata(97);c__Arachnida(97); |
| OTU_995 | 0 | 0 | 2 | 0 | 0 | 0 | 0 | 0 | 0 | 0 | 0 | 0 | All_GROUP:sk__Eukaryota(100);sknr0__Opisthokonta(100);sknr1__Holozoa(100);sknr2__Metazoa_(Animalia)(100);sknr3__Eumetazoa(100);sknr4__Bilateria(100);p__Arthropoda(100);subp__Chelicerata(95);c__Arachnida(95); |
| OTU_84 | 19 | 1 | 54 | 2 | 1 | 3 | 157 | 17 | 19 | 174 | 32 | 19 | All_GROUP:sk__Eukaryota(100);sknr0__Opisthokonta(100);sknr1__Holozoa(100);sknr2__Metazoa_(Animalia)(100);sknr3__Eumetazoa(100);sknr4__Bilateria(100);p__Arthropoda(100);subp__Chelicerata(100);c__Arachnida(100);subc__Acari(100);s__Brachychthoniidae_gen._sp._3_AD1301(100); |
| OTU_198 | 3 | 1 | 11 | 0 | 0 | 0 | 77 | 4 | 8 | 0 | 0 | 0 | All_GROUP:sk__Eukaryota(100);sknr0__Opisthokonta(100);sknr1__Holozoa(100);sknr2__Metazoa_(Animalia)(100);sknr3__Eumetazoa(100);sknr4__Bilateria(100);p__Arthropoda(100);subp__Chelicerata(100);c__Arachnida(100);subc__Acari(100);s__Eupodidae_sp._AMUENV025(94); |
| OTU_218 | 3 | 0 | 1 | 0 | 0 | 0 | 5 | 3 | 74 | 0 | 0 | 0 | All_GROUP:sk__Eukaryota(100);sknr0__Opisthokonta(100);sknr1__Holozoa(100);sknr2__Metazoa_(Animalia)(100);sknr3__Eumetazoa(100);sknr4__Bilateria(100);p__Arthropoda(100);subp__Chelicerata(100);c__Arachnida(100);subc__Acari(100);s__Galumna_lanceata(91); |
| OTU_32 | 0 | 0 | 0 | 0 | 0 | 0 | 0 | 0 | 0 | 35 | 645 | 136 | All_GROUP:sk__Eukaryota(100);sknr0__Opisthokonta(100);sknr1__Holozoa(100);sknr2__Metazoa_(Animalia)(100);sknr3__Eumetazoa(100);sknr4__Bilateria(100);p__Arthropoda(100);subp__Chelicerata(100);c__Arachnida(100);subc__Acari(100);s__Haemaphysalis_formosensis(82); |
| OTU_14 | 82 | 25 | 489 | 0 | 0 | 0 | 78 | 592 | 539 | 92 | 20 | 3 | All_GROUP:sk__Eukaryota(100);sknr0__Opisthokonta(100);sknr1__Holozoa(100);sknr2__Metazoa_(Animalia)(100);sknr3__Eumetazoa(100);sknr4__Bilateria(100);p__Arthropoda(100);subp__Chelicerata(100);c__Arachnida(100);subc__Acari(100);s__Limulidae_environmental_sample(100); |
| OTU_407 | 0 | 34 | 0 | 0 | 0 | 0 | 0 | 0 | 0 | 0 | 0 | 0 | All_GROUP:sk__Eukaryota(100);sknr0__Opisthokonta(100);sknr1__Holozoa(100);sknr2__Metazoa_(Animalia)(100);sknr3__Eumetazoa(100);sknr4__Bilateria(100);p__Arthropoda(100);subp__Chelicerata(100);c__Arachnida(100);subc__Acari(100);s__Oribatula_sakamorii(99); |
| OTU_10 | 2753 | 3 | 60 | 74 | 247 | 137 | 57 | 36 | 11 | 0 | 0 | 13 | All_GROUP:sk__Eukaryota(100);sknr0__Opisthokonta(100);sknr1__Holozoa(100);sknr2__Metazoa_(Animalia)(100);sknr3__Eumetazoa(100);sknr4__Bilateria(100);p__Arthropoda(100);subp__Chelicerata(100);c__Arachnida(100);subc__Acari(100);s__Oribatula_tibialis(92); |
| OTU_31 | 29 | 226 | 20 | 309 | 357 | 432 | 0 | 129 | 8 | 0 | 1 | 6 | All_GROUP:sk__Eukaryota(100);sknr0__Opisthokonta(100);sknr1__Holozoa(100);sknr2__Metazoa_(Animalia)(100);sknr3__Eumetazoa(100);sknr4__Bilateria(100);p__Arthropoda(100);subp__Chelicerata(100);c__Arachnida(100);subc__Acari(100);s__Protoribates_hakonensis(85); |
| OTU_295 | 5 | 88 | 17 | 0 | 0 | 0 | 0 | 2 | 0 | 0 | 0 | 0 | All_GROUP:sk__Eukaryota(100);sknr0__Opisthokonta(100);sknr1__Holozoa(100);sknr2__Metazoa_(Animalia)(100);sknr3__Eumetazoa(100);sknr4__Bilateria(100);p__Arthropoda(100);subp__Chelicerata(100);c__Arachnida(100);subc__Acari(100);s__Scheloribates_pallidulus(100); |
| OTU_26 | 27 | 20 | 0 | 0 | 0 | 0 | 65 | 0 | 920 | 204 | 0 | 44 | All_GROUP:sk__Eukaryota(100);sknr0__Opisthokonta(100);sknr1__Holozoa(100);sknr2__Metazoa_(Animalia)(100);sknr3__Eumetazoa(100);sknr4__Bilateria(100);p__Arthropoda(100);subp__Chelicerata(100);c__Arachnida(100);subc__Acari(100);s__Stigmalychus_sp._AD1318(100); |
| OTU_167 | 4 | 0 | 1 | 78 | 21 | 26 | 0 | 0 | 0 | 0 | 0 | 0 | All_GROUP:sk__Eukaryota(100);sknr0__Opisthokonta(100);sknr1__Holozoa(100);sknr2__Metazoa_(Animalia)(100);sknr3__Eumetazoa(100);sknr4__Bilateria(100);p__Arthropoda(100);subp__Chelicerata(100);c__Arachnida(100);subc__Acari(100);s__Tectocepheus_sarekensis(98); |
| OTU_24 | 2 | 1093 | 8 | 0 | 0 | 1 | 0 | 0 | 0 | 0 | 0 | 0 | All_GROUP:sk__Eukaryota(100);sknr0__Opisthokonta(100);sknr1__Holozoa(100);sknr2__Metazoa_(Animalia)(100);sknr3__Eumetazoa(100);sknr4__Bilateria(100);p__Arthropoda(100);subp__Chelicerata(100);c__Arachnida(100);subc__Acari(100); |
| OTU_38 | 30 | 21 | 55 | 0 | 3 | 0 | 163 | 35 | 6 | 71 | 25 | 87 | All_GROUP:sk__Eukaryota(100);sknr0__Opisthokonta(100);sknr1__Holozoa(100);sknr2__Metazoa_(Animalia)(100);sknr3__Eumetazoa(100);sknr4__Bilateria(100);p__Arthropoda(100);subp__Chelicerata(100);c__Arachnida(100);subc__Acari(100); |
| OTU_50 | 0 | 6 | 6 | 0 | 3 | 2 | 12 | 0 | 318 | 0 | 2 | 0 | All_GROUP:sk__Eukaryota(100);sknr0__Opisthokonta(100);sknr1__Holozoa(100);sknr2__Metazoa_(Animalia)(100);sknr3__Eumetazoa(100);sknr4__Bilateria(100);p__Arthropoda(99);subp__Chelicerata(99);c__Arachnida(99);subc__Acari(97); |
| OTU_95 | 0 | 0 | 0 | 0 | 0 | 0 | 25 | 25 | 82 | 1 | 0 | 0 | All_GROUP:sk__Eukaryota(100);sknr0__Opisthokonta(100);sknr1__Holozoa(100);sknr2__Metazoa_(Animalia)(100);sknr3__Eumetazoa(100);sknr4__Bilateria(100);p__Arthropoda(100);subp__Chelicerata(100);c__Arachnida(100);subc__Acari(100); |
| OTU_96 | 0 | 0 | 0 | 0 | 0 | 0 | 96 | 0 | 12 | 0 | 182 | 0 | All_GROUP:sk__Eukaryota(100);sknr0__Opisthokonta(100);sknr1__Holozoa(100);sknr2__Metazoa_(Animalia)(100);sknr3__Eumetazoa(100);sknr4__Bilateria(100);p__Arthropoda(100);subp__Chelicerata(100);c__Arachnida(100);subc__Acari(100); |
| OTU_128 | 2 | 0 | 13 | 14 | 21 | 84 | 0 | 0 | 0 | 0 | 0 | 0 | All_GROUP:sk__Eukaryota(100);sknr0__Opisthokonta(100);sknr1__Holozoa(100);sknr2__Metazoa_(Animalia)(100);sknr3__Eumetazoa(100);sknr4__Bilateria(100);p__Arthropoda(100);subp__Chelicerata(100);c__Arachnida(100);subc__Acari(100); |
| OTU_153 | 0 | 0 | 0 | 0 | 0 | 0 | 0 | 2 | 36 | 18 | 8 | 2 | All_GROUP:sk__Eukaryota(100);sknr0__Opisthokonta(100);sknr1__Holozoa(100);sknr2__Metazoa_(Animalia)(100);sknr3__Eumetazoa(100);sknr4__Bilateria(100);p__Arthropoda(100);subp__Chelicerata(100);c__Arachnida(100);subc__Acari(100); |
| OTU_221 | 21 | 3 | 0 | 0 | 0 | 0 | 65 | 4 | 37 | 0 | 0 | 0 | All_GROUP:sk__Eukaryota(100);sknr0__Opisthokonta(100);sknr1__Holozoa(100);sknr2__Metazoa_(Animalia)(100);sknr3__Eumetazoa(100);sknr4__Bilateria(100);p__Arthropoda(100);subp__Chelicerata(100);c__Arachnida(100);subc__Acari(100); |
| OTU_260 | 0 | 0 | 0 | 0 | 0 | 0 | 13 | 3 | 44 | 0 | 0 | 0 | All_GROUP:sk__Eukaryota(100);sknr0__Opisthokonta(100);sknr1__Holozoa(100);sknr2__Metazoa_(Animalia)(100);sknr3__Eumetazoa(100);sknr4__Bilateria(100);p__Arthropoda(100);subp__Chelicerata(100);c__Arachnida(100);subc__Acari(100); |
| OTU_301 | 0 | 0 | 9 | 35 | 5 | 5 | 0 | 0 | 6 | 0 | 13 | 0 | All_GROUP:sk__Eukaryota(100);sknr0__Opisthokonta(100);sknr1__Holozoa(100);sknr2__Metazoa_(Animalia)(100);sknr3__Eumetazoa(100);sknr4__Bilateria(100);p__Arthropoda(100);subp__Chelicerata(100);c__Arachnida(100);subc__Acari(100); |
| OTU_352 | 0 | 0 | 0 | 0 | 0 | 0 | 11 | 23 | 14 | 0 | 0 | 0 | All_GROUP:sk__Eukaryota(100);sknr0__Opisthokonta(100);sknr1__Holozoa(100);sknr2__Metazoa_(Animalia)(100);sknr3__Eumetazoa(100);sknr4__Bilateria(100);p__Arthropoda(100);subp__Chelicerata(100);c__Arachnida(100);subc__Acari(100); |
| OTU_495 | 7 | 4 | 0 | 1 | 0 | 6 | 0 | 0 | 29 | 5 | 0 | 5 | All_GROUP:sk__Eukaryota(100);sknr0__Opisthokonta(100);sknr1__Holozoa(100);sknr2__Metazoa_(Animalia)(100);sknr3__Eumetazoa(100);sknr4__Bilateria(100);p__Arthropoda(100);subp__Chelicerata(100);c__Arachnida(100);subc__Acari(100); |
| OTU_513 | 0 | 8 | 0 | 1 | 25 | 9 | 0 | 0 | 0 | 0 | 0 | 0 | All_GROUP:sk__Eukaryota(100);sknr0__Opisthokonta(100);sknr1__Holozoa(100);sknr2__Metazoa_(Animalia)(100);sknr3__Eumetazoa(100);sknr4__Bilateria(100);p__Arthropoda(100);subp__Chelicerata(100);c__Arachnida(100);subc__Acari(100); |
| OTU_636 | 0 | 0 | 0 | 0 | 0 | 0 | 4 | 0 | 17 | 0 | 0 | 0 | All_GROUP:sk__Eukaryota(100);sknr0__Opisthokonta(100);sknr1__Holozoa(100);sknr2__Metazoa_(Animalia)(100);sknr3__Eumetazoa(100);sknr4__Bilateria(100);p__Arthropoda(98);subp__Chelicerata(95);c__Arachnida(95);subc__Acari(81); |
| OTU_657 | 2 | 6 | 0 | 0 | 0 | 0 | 4 | 0 | 11 | 12 | 0 | 0 | All_GROUP:sk__Eukaryota(100);sknr0__Opisthokonta(100);sknr1__Holozoa(100);sknr2__Metazoa_(Animalia)(100);sknr3__Eumetazoa(100);sknr4__Bilateria(100);p__Arthropoda(100);subp__Chelicerata(100);c__Arachnida(100);subc__Acari(99); |
| OTU_806 | 0 | 2 | 20 | 0 | 0 | 0 | 0 | 0 | 0 | 0 | 0 | 0 | All_GROUP:sk__Eukaryota(100);sknr0__Opisthokonta(100);sknr1__Holozoa(100);sknr2__Metazoa_(Animalia)(100);sknr3__Eumetazoa(100);sknr4__Bilateria(100);p__Arthropoda(100);subp__Chelicerata(100);c__Arachnida(100);subc__Acari(100); |
| OTU_828 | 0 | 0 | 0 | 0 | 0 | 0 | 0 | 0 | 0 | 0 | 14 | 0 | All_GROUP:sk__Eukaryota(100);sknr0__Opisthokonta(100);sknr1__Holozoa(100);sknr2__Metazoa_(Animalia)(100);sknr3__Eumetazoa(100);sknr4__Bilateria(100);p__Arthropoda(100);subp__Chelicerata(100);c__Arachnida(100);subc__Acari(100); |
| OTU_842 | 0 | 0 | 0 | 0 | 0 | 1 | 4 | 2 | 11 | 3 | 10 | 0 | All_GROUP:sk__Eukaryota(100);sknr0__Opisthokonta(100);sknr1__Holozoa(100);sknr2__Metazoa_(Animalia)(100);sknr3__Eumetazoa(100);sknr4__Bilateria(100);p__Arthropoda(100);subp__Chelicerata(100);c__Arachnida(100);subc__Acari(100); |
| OTU_844 | 5 | 13 | 2 | 0 | 0 | 0 | 0 | 0 | 0 | 0 | 0 | 0 | All_GROUP:sk__Eukaryota(100);sknr0__Opisthokonta(100);sknr1__Holozoa(100);sknr2__Metazoa_(Animalia)(100);sknr3__Eumetazoa(100);sknr4__Bilateria(100);p__Arthropoda(100);subp__Chelicerata(99);c__Arachnida(99);subc__Acari(96); |
| OTU_893 | 0 | 1 | 0 | 0 | 0 | 0 | 18 | 0 | 5 | 0 | 0 | 0 | All_GROUP:sk__Eukaryota(100);sknr0__Opisthokonta(100);sknr1__Holozoa(100);sknr2__Metazoa_(Animalia)(100);sknr3__Eumetazoa(100);sknr4__Bilateria(100);p__Arthropoda(100);subp__Chelicerata(100);c__Arachnida(100);subc__Acari(100); |
| OTU_1064 | 2 | 1 | 13 | 0 | 0 | 1 | 0 | 0 | 0 | 0 | 0 | 3 | All_GROUP:sk__Eukaryota(100);sknr0__Opisthokonta(100);sknr1__Holozoa(100);sknr2__Metazoa_(Animalia)(100);sknr3__Eumetazoa(100);sknr4__Bilateria(100);p__Arthropoda(100);subp__Chelicerata(100);c__Arachnida(100);subc__Acari(100); |
| OTU_1027 | 0 | 1 | 0 | 3 | 2 | 8 | 0 | 0 | 9 | 0 | 0 | 0 | All_GROUP:sk__Eukaryota(100);sknr0__Opisthokonta(100);sknr1__Holozoa(100);sknr2__Metazoa_(Animalia)(100);sknr3__Eumetazoa(100);sknr4__Bilateria(100);p__Arthropoda(100);subp__Chelicerata(100);c__Arachnida(100);subc__Acari(100);subcnr0__Puccinia_striiformis_f._sp._tritici_87/7(97); |
| OTU_41 | 439 | 65 | 14 | 0 | 0 | 0 | 0 | 2 | 0 | 0 | 0 | 7 | All_GROUP:sk__Eukaryota(100);sknr0__Opisthokonta(100);sknr1__Holozoa(100);sknr2__Metazoa_(Animalia)(100);sknr3__Eumetazoa(100);sknr4__Bilateria(100);p__Arthropoda(100);supc__Hexapoda(100);c__Ellipura(100);o__Collembola(100);s__Folsomides_parvulus(100); |
| OTU_166 | 0 | 0 | 0 | 0 | 0 | 0 | 0 | 160 | 0 | 0 | 0 | 0 | All_GROUP:sk__Eukaryota(100);sknr0__Opisthokonta(100);sknr1__Holozoa(100);sknr2__Metazoa_(Animalia)(100);sknr3__Eumetazoa(100);sknr4__Bilateria(100);p__Arthropoda(100);supc__Hexapoda(100);c__Ellipura(100);o__Protura(100);s__Kenyentulus_ciliciocalyci(84); |
| OTU_102 | 0 | 73 | 5 | 0 | 0 | 0 | 43 | 7 | 31 | 58 | 16 | 19 | All_GROUP:sk__Eukaryota(100);sknr0__Opisthokonta(100);sknr1__Holozoa(100);sknr2__Metazoa_(Animalia)(100);sknr3__Eumetazoa(100);sknr4__Bilateria(100);p__Arthropoda(100);supc__Hexapoda(100);c__Ellipura(100);o__Protura(100);s__Neocondeellum_dolichotarsum(99); |
| OTU_426 | 0 | 0 | 0 | 0 | 0 | 0 | 0 | 0 | 0 | 3 | 43 | 4 | All_GROUP:sk__Eukaryota(100);sknr0__Opisthokonta(100);sknr1__Holozoa(100);sknr2__Metazoa_(Animalia)(100);sknr3__Eumetazoa(100);sknr4__Bilateria(100);p__Arthropoda(100);supc__Hexapoda(100);c__Ellipura(100);o__Protura(100);s__Paranisentomon_triglobulum(85); |
| OTU_109 | 5 | 371 | 50 | 0 | 0 | 0 | 0 | 1 | 0 | 100 | 0 | 35 | All_GROUP:sk__Eukaryota(100);sknr0__Opisthokonta(100);sknr1__Holozoa(100);sknr2__Metazoa_(Animalia)(100);sknr3__Eumetazoa(100);sknr4__Bilateria(100);p__Arthropoda(100);supc__Hexapoda(100);c__Ellipura(100);o__Collembola(100);s__Poduridae_environmental_sample(95); |
| OTU_25 | 0 | 0 | 0 | 0 | 0 | 0 | 502 | 304 | 301 | 0 | 0 | 0 | All_GROUP:sk__Eukaryota(100);sknr0__Opisthokonta(100);sknr1__Holozoa(100);sknr2__Metazoa_(Animalia)(100);sknr3__Eumetazoa(100);sknr4__Bilateria(100);p__Arthropoda(100);supc__Hexapoda(100);c__Ellipura(100);o__Collembola(100); |
| OTU_36 | 0 | 729 | 17 | 0 | 3 | 0 | 2 | 2 | 5 | 0 | 0 | 2 | All_GROUP:sk__Eukaryota(100);sknr0__Opisthokonta(100);sknr1__Holozoa(100);sknr2__Metazoa_(Animalia)(100);sknr3__Eumetazoa(100);sknr4__Bilateria(100);p__Arthropoda(100);supc__Hexapoda(100);c__Ellipura(100);o__Collembola(100); |
| OTU_131 | 0 | 56 | 39 | 0 | 0 | 0 | 0 | 0 | 0 | 0 | 0 | 0 | All_GROUP:sk__Eukaryota(100);sknr0__Opisthokonta(100);sknr1__Holozoa(100);sknr2__Metazoa_(Animalia)(100);sknr3__Eumetazoa(100);sknr4__Bilateria(100);p__Arthropoda(100);supc__Hexapoda(99);c__Ellipura(99);o__Collembola(99); |
| OTU_320 | 3 | 4 | 4 | 2 | 0 | 0 | 8 | 13 | 25 | 1 | 4 | 19 | All_GROUP:sk__Eukaryota(100);sknr0__Opisthokonta(100);sknr1__Holozoa(100);sknr2__Metazoa_(Animalia)(100);sknr3__Eumetazoa(100);sknr4__Bilateria(100);p__Arthropoda(100);supc__Hexapoda(100);c__Ellipura(100);o__Collembola(100); |
| OTU_634 | 0 | 0 | 0 | 7 | 9 | 1 | 0 | 0 | 0 | 0 | 0 | 0 | All_GROUP:sk__Eukaryota(100);sknr0__Opisthokonta(100);sknr1__Holozoa(100);sknr2__Metazoa_(Animalia)(100);sknr3__Eumetazoa(100);sknr4__Bilateria(100);p__Arthropoda(100);supc__Hexapoda(100);c__Ellipura(100);o__Collembola(100); |
| OTU_883 | 0 | 5 | 0 | 0 | 0 | 0 | 0 | 0 | 0 | 0 | 0 | 0 | All_GROUP:sk__Eukaryota(100);sknr0__Opisthokonta(100);sknr1__Holozoa(100);sknr2__Metazoa_(Animalia)(100);sknr3__Eumetazoa(100);sknr4__Bilateria(100);p__Arthropoda(100);supc__Hexapoda(100);c__Ellipura(100);o__Collembola(100); |
| OTU_34 | 0 | 0 | 0 | 0 | 0 | 0 | 396 | 13 | 356 | 0 | 0 | 0 | All_GROUP:sk__Eukaryota(100);sknr0__Opisthokonta(100);sknr1__Holozoa(100);sknr2__Metazoa_(Animalia)(100);sknr3__Eumetazoa(100);sknr4__Bilateria(100);p__Arthropoda(100);supc__Hexapoda(100);c__Ellipura(100);o__Collembola(100);onr0__Folsomina_onychiurina(97); |
| OTU_47 | 344 | 0 | 5 | 0 | 0 | 0 | 70 | 102 | 90 | 22 | 0 | 7 | All_GROUP:sk__Eukaryota(100);sknr0__Opisthokonta(100);sknr1__Holozoa(100);sknr2__Metazoa_(Animalia)(100);sknr3__Eumetazoa(100);sknr4__Bilateria(100);p__Arthropoda(100);supc__Hexapoda(100);c__Insecta(100);g__Pterygota(100);subc__Neoptera(100);o__Hymenoptera(100); |
| OTU_442 | 0 | 0 | 0 | 10 | 1 | 2 | 36 | 33 | 295 | 2 | 3 | 8 | All_GROUP:sk__Eukaryota(100);sknr0__Opisthokonta(100);sknr1__Holozoa(100);sknr2__Metazoa_(Animalia)(100);sknr3__Eumetazoa(100);sknr4__Bilateria(100);p__Arthropoda(100);supc__Hexapoda(100);c__Ellipura(100);o__Collembola(100);onr0__Podura_aquatica_(water_springtail)(93); |
| OTU_268 | 40 | 35 | 8 | 15 | 3 | 9 | 2 | 1 | 0 | 0 | 1 | 0 | All_GROUP:sk__Eukaryota(100);sknr0__Opisthokonta(100);sknr1__Holozoa(100);sknr2__Metazoa_(Animalia)(100);sknr3__Eumetazoa(100);sknr4__Bilateria(100);p__Arthropoda(100);supc__Hexapoda(100);c__Ellipura(100);o__Collembola(100);oun0__uncultured_eukaryote(99); |
| OTU_270 | 0 | 0 | 34 | 8 | 20 | 19 | 0 | 0 | 0 | 7 | 0 | 0 | All_GROUP:sk__Eukaryota(100);sknr0__Opisthokonta(100);sknr1__Holozoa(100);sknr2__Metazoa_(Animalia)(100);sknr3__Eumetazoa(100);sknr4__Bilateria(100);p__Nematoda(100);c__Chromadorea(100);o__Tylenchida(100);s__Aphelenchoides_bicaudatus(100); |
| OTU_200 | 25 | 6 | 38 | 23 | 10 | 6 | 1 | 0 | 0 | 0 | 0 | 0 | All_GROUP:sk__Eukaryota(100);sknr0__Opisthokonta(100);sknr1__Holozoa(100);sknr2__Metazoa_(Animalia)(100);sknr3__Eumetazoa(100);sknr4__Bilateria(100);p__Nematoda(100);c__Chromadorea(100);o__Tylenchida(100);s__Aphelenchus_avenae(100); |
| OTU_388 | 13 | 2 | 0 | 2 | 1 | 2 | 0 | 0 | 0 | 0 | 0 | 8 | All_GROUP:sk__Eukaryota(100);sknr0__Opisthokonta(100);sknr1__Holozoa(100);sknr2__Metazoa_(Animalia)(100);sknr3__Eumetazoa(100);sknr4__Bilateria(100);p__Nematoda(100);c__Chromadorea(100);o__Rhabditida(100);s__Cephalobus_cubaensis(100); |
| OTU_250 | 7 | 0 | 0 | 8 | 5 | 8 | 0 | 1 | 0 | 0 | 0 | 0 | All_GROUP:sk__Eukaryota(100);sknr0__Opisthokonta(100);sknr1__Holozoa(100);sknr2__Metazoa_(Animalia)(100);sknr3__Eumetazoa(100);sknr4__Bilateria(100);p__Nematoda(100);c__Chromadorea(100);o__Rhabditida(100);s__Ceroglossini_environmental_sample(99); |
| OTU_70 | 0 | 0 | 0 | 27 | 74 | 62 | 0 | 0 | 0 | 0 | 0 | 175 | All_GROUP:sk__Eukaryota(100);sknr0__Opisthokonta(100);sknr1__Holozoa(100);sknr2__Metazoa_(Animalia)(100);sknr3__Eumetazoa(100);sknr4__Bilateria(100);p__Nematoda(100);c__Chromadorea(100);o__Rhabditida(100);s__Diploscapter_sp._PS1897(100); |
| OTU_223 | 0 | 0 | 0 | 0 | 0 | 0 | 0 | 0 | 56 | 0 | 0 | 0 | All_GROUP:sk__Eukaryota(100);sknr0__Opisthokonta(100);sknr1__Holozoa(100);sknr2__Metazoa_(Animalia)(100);sknr3__Eumetazoa(100);sknr4__Bilateria(100);p__Nematoda(100);c__Chromadorea(100);o__Tylenchida(100);s__Ditylenchus_dipsaci(96); |
| OTU_171 | 0 | 1 | 18 | 10 | 0 | 1 | 0 | 187 | 0 | 31 | 22 | 18 | All_GROUP:sk__Eukaryota(100);sknr0__Opisthokonta(100);sknr1__Holozoa(100);sknr2__Metazoa_(Animalia)(100);sknr3__Eumetazoa(100);sknr4__Bilateria(100);p__Nematoda(100);c__Chromadorea(100);o__Tylenchida(100);s__Filenchus_misellus(100); |
| OTU_488 | 0 | 29 | 0 | 0 | 2 | 0 | 0 | 5 | 0 | 0 | 0 | 0 | All_GROUP:sk__Eukaryota(100);sknr0__Opisthokonta(100);sknr1__Holozoa(100);sknr2__Metazoa_(Animalia)(100);sknr3__Eumetazoa(100);sknr4__Bilateria(100);p__Nematoda(100);c__Chromadorea(100);o__Tylenchida(100);s__Filenchus_misellus(100); |
| OTU_1050 | 0 | 0 | 0 | 0 | 0 | 0 | 0 | 0 | 0 | 31 | 0 | 0 | All_GROUP:sk__Eukaryota(100);sknr0__Opisthokonta(100);sknr1__Holozoa(100);sknr2__Metazoa_(Animalia)(100);sknr3__Eumetazoa(100);sknr4__Bilateria(100);p__Nematoda(100);c__Chromadorea(100);o__Monhysterida(100);s__Geomonhystera_sp._1998(100); |
| OTU_71 | 9 | 5 | 38 | 0 | 0 | 0 | 0 | 0 | 3 | 68 | 108 | 53 | All_GROUP:sk__Eukaryota(100);sknr0__Opisthokonta(100);sknr1__Holozoa(100);sknr2__Metazoa_(Animalia)(100);sknr3__Eumetazoa(100);sknr4__Bilateria(100);p__Nematoda(100);c__Chromadorea(100);o__Tylenchida(100);s__Helicotylenchus_pseudorobustus(99); |
| OTU_98 | 0 | 0 | 0 | 61 | 18 | 65 | 0 | 0 | 0 | 0 | 0 | 0 | All_GROUP:sk__Eukaryota(100);sknr0__Opisthokonta(100);sknr1__Holozoa(100);sknr2__Metazoa_(Animalia)(100);sknr3__Eumetazoa(100);sknr4__Bilateria(100);p__Nematoda(100);c__Chromadorea(100);o__Tylenchida(100);s__Heterodera_avenae(88); |
| OTU_40 | 201 | 345 | 297 | 86 | 8 | 41 | 424 | 257 | 314 | 27 | 87 | 248 | All_GROUP:sk__Eukaryota(100);sknr0__Opisthokonta(100);sknr1__Holozoa(100);sknr2__Metazoa_(Animalia)(100);sknr3__Eumetazoa(100);sknr4__Bilateria(100);p__Nematoda(100);c__Chromadorea(100);o__Araeolaimida(100);s__Nematoda_environmental_sample(100); |
| OTU_117 | 0 | 0 | 0 | 0 | 0 | 0 | 76 | 0 | 0 | 0 | 0 | 0 | All_GROUP:sk__Eukaryota(100);sknr0__Opisthokonta(100);sknr1__Holozoa(100);sknr2__Metazoa_(Animalia)(100);sknr3__Eumetazoa(100);sknr4__Bilateria(100);p__Nematoda(100);c__Chromadorea(100);o__Tylenchida(100);s__Nematoda_environmental_sample(88); |
| OTU_160 | 0 | 0 | 0 | 14 | 55 | 14 | 0 | 0 | 0 | 0 | 0 | 0 | All_GROUP:sk__Eukaryota(100);sknr0__Opisthokonta(100);sknr1__Holozoa(100);sknr2__Metazoa_(Animalia)(100);sknr3__Eumetazoa(100);sknr4__Bilateria(100);p__Nematoda(100);c__Chromadorea(100);o__Rhabditida(100);s__Oscheius_tipulae(100); |
| OTU_662 | 0 | 0 | 0 | 4 | 2 | 3 | 0 | 0 | 13 | 0 | 0 | 0 | All_GROUP:sk__Eukaryota(100);sknr0__Opisthokonta(100);sknr1__Holozoa(100);sknr2__Metazoa_(Animalia)(100);sknr3__Eumetazoa(100);sknr4__Bilateria(100);p__Nematoda(100);c__Chromadorea(100);o__Rhabditida(100);s__Panagrolaimus_detritophagus(96); |
| OTU_120 | 11 | 13 | 35 | 2 | 4 | 2 | 67 | 29 | 76 | 32 | 10 | 38 | All_GROUP:sk__Eukaryota(100);sknr0__Opisthokonta(100);sknr1__Holozoa(100);sknr2__Metazoa_(Animalia)(100);sknr3__Eumetazoa(100);sknr4__Bilateria(100);p__Nematoda(100);c__Chromadorea(100);o__Monhysterida(100);s__Paralamyctes_environmental_sample(100); |
| OTU_507 | 0 | 16 | 14 | 0 | 0 | 0 | 0 | 0 | 0 | 0 | 0 | 0 | All_GROUP:sk__Eukaryota(100);sknr0__Opisthokonta(100);sknr1__Holozoa(100);sknr2__Metazoa_(Animalia)(100);sknr3__Eumetazoa(100);sknr4__Bilateria(100);p__Nematoda(100);c__Chromadorea(100);o__Monhysterida(100);s__Paralamyctes_environmental_sample(99); |
| OTU_82 | 0 | 0 | 47 | 0 | 2 | 0 | 70 | 93 | 75 | 134 | 38 | 57 | All_GROUP:sk__Eukaryota(100);sknr0__Opisthokonta(100);sknr1__Holozoa(100);sknr2__Metazoa_(Animalia)(100);sknr3__Eumetazoa(100);sknr4__Bilateria(100);p__Nematoda(100);c__Chromadorea(100);o__Tylenchida(100);s__Paratylenchus_dianthus(86); |
| OTU_729 | 0 | 0 | 0 | 0 | 0 | 0 | 4 | 0 | 16 | 0 | 0 | 0 | All_GROUP:sk__Eukaryota(100);sknr0__Opisthokonta(100);sknr1__Holozoa(100);sknr2__Metazoa_(Animalia)(100);sknr3__Eumetazoa(100);sknr4__Bilateria(100);p__Nematoda(100);c__Chromadorea(100);o__Tylenchida(100);s__Paratylenchus_dianthus(84); |
| OTU_1037 | 0 | 10 | 1 | 0 | 5 | 0 | 0 | 0 | 0 | 0 | 0 | 0 | All_GROUP:sk__Eukaryota(100);sknr0__Opisthokonta(100);sknr1__Holozoa(100);sknr2__Metazoa_(Animalia)(100);sknr3__Eumetazoa(100);sknr4__Bilateria(100);p__Nematoda(100);c__Chromadorea(100);o__Tylenchida(100);s__Pratylenchus_neglectus(100); |
| OTU_193 | 1 | 0 | 5 | 111 | 82 | 45 | 0 | 0 | 0 | 0 | 0 | 2 | All_GROUP:sk__Eukaryota(100);sknr0__Opisthokonta(100);sknr1__Holozoa(100);sknr2__Metazoa_(Animalia)(100);sknr3__Eumetazoa(100);sknr4__Bilateria(100);p__Nematoda(100);c__Chromadorea(100);o__Tylenchida(100);s__Pratylenchus_scribneri(100); |
| OTU_763 | 0 | 0 | 0 | 12 | 8 | 8 | 0 | 0 | 0 | 0 | 0 | 0 | All_GROUP:sk__Eukaryota(100);sknr0__Opisthokonta(100);sknr1__Holozoa(100);sknr2__Metazoa_(Animalia)(100);sknr3__Eumetazoa(100);sknr4__Bilateria(100);p__Nematoda(100);c__Chromadorea(100);o__Tylenchida(100);s__Pratylenchus_scribneri(100); |
| OTU_181 | 0 | 0 | 0 | 47 | 36 | 52 | 0 | 0 | 0 | 0 | 0 | 2 | All_GROUP:sk__Eukaryota(100);sknr0__Opisthokonta(100);sknr1__Holozoa(100);sknr2__Metazoa_(Animalia)(100);sknr3__Eumetazoa(100);sknr4__Bilateria(100);p__Nematoda(100);c__Chromadorea(100);o__Tylenchida(100);s__Rotylenchulus_reniformis(100); |
| OTU_242 | 0 | 0 | 0 | 24 | 28 | 42 | 4 | 0 | 0 | 0 | 0 | 0 | All_GROUP:sk__Eukaryota(100);sknr0__Opisthokonta(100);sknr1__Holozoa(100);sknr2__Metazoa_(Animalia)(100);sknr3__Eumetazoa(100);sknr4__Bilateria(100);p__Nematoda(100);c__Chromadorea(100);o__Tylenchida(100);s__Rotylenchulus_reniformis(100); |
| OTU_213 | 0 | 0 | 0 | 50 | 13 | 4 | 0 | 0 | 0 | 0 | 0 | 0 | All_GROUP:sk__Eukaryota(100);sknr0__Opisthokonta(100);sknr1__Holozoa(100);sknr2__Metazoa_(Animalia)(100);sknr3__Eumetazoa(100);sknr4__Bilateria(100);p__Nematoda(100);c__Chromadorea(100);o__Tylenchida(100);s__Tylenchorhynchus_leviterminalis(83); |
| OTU_750 | 0 | 27 | 0 | 2 | 1 | 4 | 0 | 0 | 0 | 0 | 0 | 0 | All_GROUP:sk__Eukaryota(100);sknr0__Opisthokonta(100);sknr1__Holozoa(100);sknr2__Metazoa_(Animalia)(100);sknr3__Eumetazoa(100);sknr4__Bilateria(100);p__Nematoda(100);c__Chromadorea(100);o__Tylenchida(100);s__Tylenchus_arcuatus(100); |
| OTU_843 | 103 | 145 | 165 | 24 | 2 | 9 | 142 | 174 | 43 | 5 | 32 | 136 | All_GROUP:sk__Eukaryota(100);sknr0__Opisthokonta(100);sknr1__Holozoa(100);sknr2__Metazoa_(Animalia)(100);sknr3__Eumetazoa(100);sknr4__Bilateria(100);p__Nematoda(100);c__Chromadorea(100);o__Araeolaimida(100);s__Tylocephalus_auriculatus(82); |
| OTU_44 | 0 | 0 | 0 | 445 | 23 | 5 | 0 | 0 | 0 | 0 | 0 | 0 | All_GROUP:sk__Eukaryota(100);sknr0__Opisthokonta(100);sknr1__Holozoa(100);sknr2__Metazoa_(Animalia)(100);sknr3__Eumetazoa(100);sknr4__Bilateria(100);p__Nematoda(100);c__Chromadorea(100);o__Diplogasterida(100); |
| OTU_701 | 18 | 0 | 6 | 0 | 0 | 0 | 0 | 0 | 0 | 0 | 0 | 0 | All_GROUP:sk__Eukaryota(100);sknr0__Opisthokonta(100);sknr1__Holozoa(100);sknr2__Metazoa_(Animalia)(100);sknr3__Eumetazoa(100);sknr4__Bilateria(100);p__Nematoda(100);c__Chromadorea(99);o__Monhysterida(99); |
| OTU_18 | 109 | 567 | 40 | 156 | 282 | 156 | 113 | 131 | 31 | 153 | 19 | 571 | All_GROUP:sk__Eukaryota(100);sknr0__Opisthokonta(100);sknr1__Holozoa(100);sknr2__Metazoa_(Animalia)(100);sknr3__Eumetazoa(100);sknr4__Bilateria(100);p__Nematoda(100);c__Chromadorea(100);o__Rhabditida(100); |
| OTU_292 | 0 | 1 | 0 | 41 | 23 | 6 | 0 | 0 | 0 | 3 | 0 | 0 | All_GROUP:sk__Eukaryota(100);sknr0__Opisthokonta(100);sknr1__Holozoa(100);sknr2__Metazoa_(Animalia)(100);sknr3__Eumetazoa(100);sknr4__Bilateria(100);p__Nematoda(100);c__Chromadorea(100);o__Rhabditida(100); |
| OTU_411 | 0 | 0 | 0 | 0 | 0 | 0 | 0 | 26 | 22 | 2 | 13 | 0 | All_GROUP:sk__Eukaryota(100);sknr0__Opisthokonta(100);sknr1__Holozoa(100);sknr2__Metazoa_(Animalia)(100);sknr3__Eumetazoa(100);sknr4__Bilateria(100);p__Nematoda(100);c__Chromadorea(100);o__Rhabditida(100); |
| OTU_486 | 0 | 4 | 0 | 0 | 0 | 0 | 3 | 9 | 1 | 23 | 0 | 0 | All_GROUP:sk__Eukaryota(100);sknr0__Opisthokonta(100);sknr1__Holozoa(100);sknr2__Metazoa_(Animalia)(100);sknr3__Eumetazoa(100);sknr4__Bilateria(100);p__Nematoda(100);c__Chromadorea(100);o__Rhabditida(100); |
| OTU_611 | 0 | 0 | 0 | 0 | 1 | 0 | 4 | 0 | 15 | 5 | 0 | 0 | All_GROUP:sk__Eukaryota(100);sknr0__Opisthokonta(100);sknr1__Holozoa(100);sknr2__Metazoa_(Animalia)(100);sknr3__Eumetazoa(100);sknr4__Bilateria(100);p__Nematoda(100);c__Chromadorea(100);o__Rhabditida(100); |
| OTU_643 | 0 | 0 | 0 | 0 | 0 | 0 | 16 | 0 | 0 | 0 | 0 | 0 | All_GROUP:sk__Eukaryota(100);sknr0__Opisthokonta(100);sknr1__Holozoa(100);sknr2__Metazoa_(Animalia)(100);sknr3__Eumetazoa(100);sknr4__Bilateria(100);p__Nematoda(100);c__Chromadorea(100);o__Rhabditida(99); |
| OTU_681 | 0 | 0 | 0 | 2 | 6 | 5 | 0 | 16 | 2 | 0 | 0 | 0 | All_GROUP:sk__Eukaryota(100);sknr0__Opisthokonta(100);sknr1__Holozoa(100);sknr2__Metazoa_(Animalia)(100);sknr3__Eumetazoa(100);sknr4__Bilateria(100);p__Nematoda(100);c__Chromadorea(100);o__Rhabditida(100); |
| OTU_715 | 0 | 0 | 0 | 0 | 12 | 32 | 0 | 0 | 0 | 0 | 0 | 0 | All_GROUP:sk__Eukaryota(100);sknr0__Opisthokonta(100);sknr1__Holozoa(100);sknr2__Metazoa_(Animalia)(100);sknr3__Eumetazoa(100);sknr4__Bilateria(100);p__Nematoda(100);c__Chromadorea(100);o__Rhabditida(100); |
| OTU_812 | 0 | 0 | 0 | 1 | 5 | 0 | 0 | 0 | 0 | 0 | 0 | 0 | All_GROUP:sk__Eukaryota(100);sknr0__Opisthokonta(100);sknr1__Holozoa(100);sknr2__Metazoa_(Animalia)(100);sknr3__Eumetazoa(100);sknr4__Bilateria(100);p__Nematoda(100);c__Chromadorea(100);o__Rhabditida(100); |
| OTU_822 | 0 | 0 | 0 | 5 | 13 | 18 | 0 | 0 | 0 | 0 | 0 | 0 | All_GROUP:sk__Eukaryota(100);sknr0__Opisthokonta(100);sknr1__Holozoa(100);sknr2__Metazoa_(Animalia)(100);sknr3__Eumetazoa(100);sknr4__Bilateria(100);p__Nematoda(100);c__Chromadorea(100);o__Rhabditida(100); |
| OTU_985 | 0 | 0 | 3 | 2 | 0 | 1 | 0 | 0 | 0 | 0 | 7 | 0 | All_GROUP:sk__Eukaryota(100);sknr0__Opisthokonta(100);sknr1__Holozoa(100);sknr2__Metazoa_(Animalia)(100);sknr3__Eumetazoa(100);sknr4__Bilateria(100);p__Nematoda(100);c__Chromadorea(100);o__Rhabditida(100); |
| OTU_1059 | 0 | 0 | 0 | 0 | 0 | 0 | 0 | 0 | 0 | 16 | 0 | 0 | All_GROUP:sk__Eukaryota(100);sknr0__Opisthokonta(100);sknr1__Holozoa(100);sknr2__Metazoa_(Animalia)(100);sknr3__Eumetazoa(100);sknr4__Bilateria(100);p__Nematoda(100);c__Chromadorea(100);o__Rhabditida(100); |
| OTU_7 | 931 | 316 | 2410 | 0 | 0 | 2 | 977 | 952 | 907 | 157 | 150 | 194 | All_GROUP:sk__Eukaryota(100);sknr0__Opisthokonta(100);sknr1__Holozoa(100);sknr2__Metazoa_(Animalia)(100);sknr3__Eumetazoa(100);sknr4__Bilateria(100);p__Nematoda(100);c__Chromadorea(100);o__Tylenchida(100); |
| OTU_23 | 327 | 113 | 51 | 22 | 7 | 24 | 0 | 2 | 0 | 128 | 257 | 176 | All_GROUP:sk__Eukaryota(100);sknr0__Opisthokonta(100);sknr1__Holozoa(100);sknr2__Metazoa_(Animalia)(100);sknr3__Eumetazoa(100);sknr4__Bilateria(100);p__Nematoda(100);c__Chromadorea(100);o__Tylenchida(100); |
| OTU_46 | 0 | 44 | 5 | 0 | 0 | 0 | 409 | 24 | 32 | 19 | 7 | 167 | All_GROUP:sk__Eukaryota(100);sknr0__Opisthokonta(100);sknr1__Holozoa(100);sknr2__Metazoa_(Animalia)(100);sknr3__Eumetazoa(100);sknr4__Bilateria(100);p__Nematoda(100);c__Chromadorea(100);o__Tylenchida(100); |
| OTU_68 | 11 | 138 | 3 | 5 | 0 | 3 | 2 | 1 | 21 | 0 | 3 | 38 | All_GROUP:sk__Eukaryota(100);sknr0__Opisthokonta(100);sknr1__Holozoa(100);sknr2__Metazoa_(Animalia)(100);sknr3__Eumetazoa(100);sknr4__Bilateria(100);p__Nematoda(100);c__Chromadorea(100);o__Tylenchida(100); |
| OTU_80 | 24 | 31 | 41 | 0 | 0 | 0 | 7 | 0 | 23 | 43 | 0 | 10 | All_GROUP:sk__Eukaryota(100);sknr0__Opisthokonta(100);sknr1__Holozoa(100);sknr2__Metazoa_(Animalia)(100);sknr3__Eumetazoa(100);sknr4__Bilateria(100);p__Nematoda(100);c__Chromadorea(100);o__Tylenchida(100); |
| OTU_101 | 0 | 10 | 22 | 1 | 9 | 23 | 0 | 0 | 3 | 14 | 11 | 80 | All_GROUP:sk__Eukaryota(100);sknr0__Opisthokonta(100);sknr1__Holozoa(100);sknr2__Metazoa_(Animalia)(100);sknr3__Eumetazoa(100);sknr4__Bilateria(100);p__Nematoda(100);c__Chromadorea(100);o__Tylenchida(100); |
| OTU_132 | 0 | 85 | 0 | 0 | 0 | 0 | 0 | 0 | 88 | 0 | 0 | 0 | All_GROUP:sk__Eukaryota(100);sknr0__Opisthokonta(100);sknr1__Holozoa(100);sknr2__Metazoa_(Animalia)(100);sknr3__Eumetazoa(100);sknr4__Bilateria(100);p__Nematoda(100);c__Chromadorea(100);o__Tylenchida(100); |
| OTU_149 | 0 | 0 | 0 | 0 | 0 | 0 | 3 | 13 | 146 | 1 | 0 | 6 | All_GROUP:sk__Eukaryota(100);sknr0__Opisthokonta(100);sknr1__Holozoa(100);sknr2__Metazoa_(Animalia)(100);sknr3__Eumetazoa(100);sknr4__Bilateria(100);p__Nematoda(100);c__Chromadorea(100);o__Tylenchida(100); |
| OTU_158 | 0 | 0 | 0 | 0 | 0 | 0 | 22 | 124 | 46 | 0 | 0 | 0 | All_GROUP:sk__Eukaryota(100);sknr0__Opisthokonta(100);sknr1__Holozoa(100);sknr2__Metazoa_(Animalia)(100);sknr3__Eumetazoa(100);sknr4__Bilateria(100);p__Nematoda(100);c__Chromadorea(100);o__Tylenchida(100); |
| OTU_316 | 0 | 11 | 5 | 0 | 0 | 0 | 0 | 0 | 40 | 0 | 0 | 0 | All_GROUP:sk__Eukaryota(100);sknr0__Opisthokonta(100);sknr1__Holozoa(100);sknr2__Metazoa_(Animalia)(100);sknr3__Eumetazoa(100);sknr4__Bilateria(100);p__Nematoda(100);c__Chromadorea(100);o__Tylenchida(100); |
| OTU_380 | 1 | 4 | 10 | 6 | 3 | 0 | 5 | 7 | 54 | 4 | 8 | 22 | All_GROUP:sk__Eukaryota(100);sknr0__Opisthokonta(100);sknr1__Holozoa(100);sknr2__Metazoa_(Animalia)(100);sknr3__Eumetazoa(100);sknr4__Bilateria(100);p__Nematoda(100);c__Chromadorea(100);o__Tylenchida(100); |
| OTU_390 | 16 | 0 | 1 | 0 | 0 | 0 | 23 | 0 | 0 | 0 | 5 | 0 | All_GROUP:sk__Eukaryota(100);sknr0__Opisthokonta(100);sknr1__Holozoa(100);sknr2__Metazoa_(Animalia)(100);sknr3__Eumetazoa(100);sknr4__Bilateria(100);p__Nematoda(100);c__Chromadorea(100);o__Tylenchida(100); |
| OTU_432 | 13 | 7 | 34 | 2 | 4 | 7 | 0 | 0 | 0 | 0 | 0 | 0 | All_GROUP:sk__Eukaryota(100);sknr0__Opisthokonta(100);sknr1__Holozoa(100);sknr2__Metazoa_(Animalia)(100);sknr3__Eumetazoa(100);sknr4__Bilateria(100);p__Nematoda(100);c__Chromadorea(100);o__Tylenchida(100); |
| OTU_436 | 0 | 38 | 0 | 0 | 0 | 0 | 0 | 0 | 40 | 0 | 1 | 0 | All_GROUP:sk__Eukaryota(100);sknr0__Opisthokonta(100);sknr1__Holozoa(100);sknr2__Metazoa_(Animalia)(100);sknr3__Eumetazoa(100);sknr4__Bilateria(100);p__Nematoda(100);c__Chromadorea(100);o__Tylenchida(100); |
| OTU_440 | 0 | 0 | 0 | 6 | 1 | 0 | 6 | 1 | 21 | 3 | 2 | 0 | All_GROUP:sk__Eukaryota(100);sknr0__Opisthokonta(100);sknr1__Holozoa(100);sknr2__Metazoa_(Animalia)(100);sknr3__Eumetazoa(100);sknr4__Bilateria(100);p__Nematoda(100);c__Chromadorea(100);o__Tylenchida(100); |
| OTU_461 | 0 | 10 | 9 | 0 | 0 | 0 | 3 | 0 | 4 | 7 | 0 | 2 | All_GROUP:sk__Eukaryota(100);sknr0__Opisthokonta(100);sknr1__Holozoa(100);sknr2__Metazoa_(Animalia)(100);sknr3__Eumetazoa(100);sknr4__Bilateria(100);p__Nematoda(100);c__Chromadorea(100);o__Tylenchida(100); |
| OTU_479 | 0 | 0 | 0 | 0 | 0 | 0 | 8 | 27 | 13 | 0 | 0 | 3 | All_GROUP:sk__Eukaryota(100);sknr0__Opisthokonta(100);sknr1__Holozoa(100);sknr2__Metazoa_(Animalia)(100);sknr3__Eumetazoa(100);sknr4__Bilateria(100);p__Nematoda(100);c__Chromadorea(100);o__Tylenchida(100); |
| OTU_515 | 6 | 1 | 3 | 0 | 0 | 2 | 5 | 19 | 5 | 14 | 19 | 1 | All_GROUP:sk__Eukaryota(100);sknr0__Opisthokonta(100);sknr1__Holozoa(100);sknr2__Metazoa_(Animalia)(100);sknr3__Eumetazoa(100);sknr4__Bilateria(100);p__Nematoda(100);c__Chromadorea(100);o__Tylenchida(100); |
| OTU_522 | 1 | 0 | 5 | 0 | 0 | 0 | 0 | 0 | 4 | 0 | 0 | 0 | All_GROUP:sk__Eukaryota(100);sknr0__Opisthokonta(100);sknr1__Holozoa(100);sknr2__Metazoa_(Animalia)(100);sknr3__Eumetazoa(100);sknr4__Bilateria(100);p__Nematoda(100);c__Chromadorea(100);o__Tylenchida(100); |
| OTU_654 | 0 | 0 | 0 | 0 | 0 | 0 | 2 | 6 | 3 | 1 | 1 | 0 | All_GROUP:sk__Eukaryota(100);sknr0__Opisthokonta(100);sknr1__Holozoa(100);sknr2__Metazoa_(Animalia)(100);sknr3__Eumetazoa(100);sknr4__Bilateria(100);p__Nematoda(100);c__Chromadorea(100);o__Tylenchida(100); |
| OTU_749 | 0 | 0 | 0 | 0 | 0 | 0 | 17 | 0 | 2 | 0 | 0 | 0 | All_GROUP:sk__Eukaryota(100);sknr0__Opisthokonta(100);sknr1__Holozoa(100);sknr2__Metazoa_(Animalia)(100);sknr3__Eumetazoa(100);sknr4__Bilateria(100);p__Nematoda(100);c__Chromadorea(100);o__Tylenchida(100); |
| OTU_752 | 0 | 0 | 0 | 10 | 0 | 0 | 0 | 0 | 0 | 0 | 0 | 0 | All_GROUP:sk__Eukaryota(100);sknr0__Opisthokonta(100);sknr1__Holozoa(99);sknr2__Metazoa_(Animalia)(99);sknr3__Eumetazoa(99);sknr4__Bilateria(99);p__Nematoda(94);c__Chromadorea(94);o__Tylenchida(83); |
| OTU_849 | 0 | 0 | 0 | 0 | 0 | 0 | 0 | 0 | 8 | 0 | 0 | 0 | All_GROUP:sk__Eukaryota(100);sknr0__Opisthokonta(100);sknr1__Holozoa(100);sknr2__Metazoa_(Animalia)(100);sknr3__Eumetazoa(100);sknr4__Bilateria(100);p__Nematoda(100);c__Chromadorea(100);o__Tylenchida(100); |
| OTU_873 | 0 | 0 | 0 | 4 | 8 | 2 | 0 | 0 | 0 | 0 | 0 | 0 | All_GROUP:sk__Eukaryota(100);sknr0__Opisthokonta(100);sknr1__Holozoa(100);sknr2__Metazoa_(Animalia)(100);sknr3__Eumetazoa(100);sknr4__Bilateria(100);p__Nematoda(100);c__Chromadorea(100);o__Tylenchida(100); |
| OTU_898 | 0 | 0 | 0 | 0 | 1 | 0 | 0 | 0 | 0 | 14 | 15 | 2 | All_GROUP:sk__Eukaryota(100);sknr0__Opisthokonta(99);sknr1__Holozoa(99);sknr2__Metazoa_(Animalia)(99);sknr3__Eumetazoa(99);sknr4__Bilateria(99);p__Nematoda(95);c__Chromadorea(95);o__Tylenchida(88); |
| OTU_947 | 0 | 0 | 0 | 0 | 0 | 0 | 0 | 28 | 0 | 0 | 0 | 0 | All_GROUP:sk__Eukaryota(100);sknr0__Opisthokonta(100);sknr1__Holozoa(99);sknr2__Metazoa_(Animalia)(99);sknr3__Eumetazoa(99);sknr4__Bilateria(99);p__Nematoda(96);c__Chromadorea(96);o__Tylenchida(86); |
| OTU_17 | 0 | 0 | 0 | 0 | 0 | 0 | 434 | 1309 | 439 | 5 | 2 | 0 | All_GROUP:sk__Eukaryota(100);sknr0__Opisthokonta(100);sknr1__Holozoa(100);sknr2__Metazoa_(Animalia)(100);sknr3__Eumetazoa(100);sknr4__Bilateria(100);p__Nematoda(100);c__Enoplea(100);subc__Dorylaimia(100);o__Dorylaimida(100); |
| OTU_29 | 44 | 13 | 1050 | 2 | 26 | 9 | 0 | 2 | 0 | 2 | 3 | 6 | All_GROUP:sk__Eukaryota(100);sknr0__Opisthokonta(100);sknr1__Holozoa(100);sknr2__Metazoa_(Animalia)(100);sknr3__Eumetazoa(100);sknr4__Bilateria(100);p__Nematoda(100);c__Enoplea(100);subc__Dorylaimia(100);o__Dorylaimida(100); |
| OTU_64 | 86 | 45 | 32 | 1 | 3 | 1 | 83 | 9 | 43 | 63 | 11 | 28 | All_GROUP:sk__Eukaryota(100);sknr0__Opisthokonta(100);sknr1__Holozoa(100);sknr2__Metazoa_(Animalia)(100);sknr3__Eumetazoa(100);sknr4__Bilateria(100);p__Nematoda(100);c__Enoplea(100);subc__Dorylaimia(100);o__Dorylaimida(100); |
| OTU_79 | 0 | 1 | 160 | 0 | 0 | 0 | 0 | 0 | 0 | 0 | 0 | 0 | All_GROUP:sk__Eukaryota(100);sknr0__Opisthokonta(100);sknr1__Holozoa(100);sknr2__Metazoa_(Animalia)(100);sknr3__Eumetazoa(100);sknr4__Bilateria(100);p__Nematoda(97);c__Enoplea(97);subc__Dorylaimia(97);o__Dorylaimida(97); |
| OTU_106 | 10 | 22 | 3 | 12 | 0 | 4 | 11 | 16 | 176 | 0 | 0 | 0 | All_GROUP:sk__Eukaryota(100);sknr0__Opisthokonta(100);sknr1__Holozoa(100);sknr2__Metazoa_(Animalia)(100);sknr3__Eumetazoa(100);sknr4__Bilateria(100);p__Nematoda(100);c__Enoplea(100);subc__Dorylaimia(100);o__Dorylaimida(100); |
| OTU_177 | 0 | 0 | 0 | 0 | 0 | 0 | 59 | 0 | 0 | 0 | 0 | 0 | All_GROUP:sk__Eukaryota(100);sknr0__Opisthokonta(100);sknr1__Holozoa(100);sknr2__Metazoa_(Animalia)(100);sknr3__Eumetazoa(100);sknr4__Bilateria(100);p__Nematoda(100);c__Enoplea(100);subc__Dorylaimia(100);o__Dorylaimida(100); |
| OTU_333 | 15 | 7 | 0 | 0 | 0 | 0 | 7 | 34 | 2 | 0 | 1 | 1 | All_GROUP:sk__Eukaryota(100);sknr0__Opisthokonta(100);sknr1__Holozoa(100);sknr2__Metazoa_(Animalia)(100);sknr3__Eumetazoa(100);sknr4__Bilateria(100);p__Nematoda(100);c__Enoplea(100);subc__Dorylaimia(100);o__Dorylaimida(100); |
| OTU_649 | 0 | 0 | 22 | 0 | 0 | 0 | 0 | 0 | 0 | 0 | 0 | 0 | All_GROUP:sk__Eukaryota(100);sknr0__Opisthokonta(100);sknr1__Holozoa(100);sknr2__Metazoa_(Animalia)(100);sknr3__Eumetazoa(100);sknr4__Bilateria(100);p__Nematoda(99);c__Enoplea(99);subc__Dorylaimia(98);o__Dorylaimida(98); |
| OTU_714 | 1 | 2 | 25 | 0 | 0 | 0 | 4 | 0 | 3 | 4 | 2 | 1 | All_GROUP:sk__Eukaryota(100);sknr0__Opisthokonta(100);sknr1__Holozoa(100);sknr2__Metazoa_(Animalia)(100);sknr3__Eumetazoa(100);sknr4__Bilateria(100);p__Nematoda(100);c__Enoplea(100);subc__Dorylaimia(100);o__Dorylaimida(100); |
| OTU_1021 | 0 | 0 | 0 | 0 | 0 | 0 | 18 | 0 | 0 | 3 | 0 | 0 | All_GROUP:sk__Eukaryota(100);sknr0__Opisthokonta(100);sknr1__Holozoa(100);sknr2__Metazoa_(Animalia)(100);sknr3__Eumetazoa(100);sknr4__Bilateria(100);p__Nematoda(100);c__Enoplea(100);subc__Dorylaimia(100);o__Dorylaimida(100); |
| OTU_67 | 0 | 0 | 0 | 1 | 0 | 3 | 3 | 31 | 249 | 0 | 0 | 0 | All_GROUP:sk__Eukaryota(100);sknr0__Opisthokonta(100);sknr1__Holozoa(100);sknr2__Metazoa_(Animalia)(100);sknr3__Eumetazoa(100);sknr4__Bilateria(100);p__Nematoda(100);c__Enoplea(100);subc__Enoplia(100);o__Enoplida(100);s__Alaimus_parvus(100); |
| OTU_403 | 0 | 0 | 0 | 0 | 0 | 0 | 32 | 0 | 13 | 0 | 8 | 4 | All_GROUP:sk__Eukaryota(100);sknr0__Opisthokonta(100);sknr1__Holozoa(100);sknr2__Metazoa_(Animalia)(100);sknr3__Eumetazoa(100);sknr4__Bilateria(100);p__Nematoda(100);c__Enoplea(100);subc__Enoplia(100);o__Enoplida(100);s__Alaimus_sp._PDL-2005(92); |
| OTU_85 | 0 | 23 | 115 | 74 | 31 | 2 | 0 | 143 | 3 | 8 | 0 | 0 | All_GROUP:sk__Eukaryota(100);sknr0__Opisthokonta(100);sknr1__Holozoa(100);sknr2__Metazoa_(Animalia)(100);sknr3__Eumetazoa(100);sknr4__Bilateria(100);p__Nematoda(100);c__Enoplea(100);subc__Enoplia(100);o__Triplonchida(100);s__Odontolaimus_sp._OdLaSp1(100); |
| OTU_832 | 4 | 0 | 0 | 0 | 0 | 0 | 30 | 0 | 0 | 0 | 0 | 0 | All_GROUP:sk__Eukaryota(100);sknr0__Opisthokonta(100);sknr1__Holozoa(100);sknr2__Metazoa_(Animalia)(100);sknr3__Eumetazoa(100);sknr4__Bilateria(100);p__Nematoda(100);c__Enoplea(100);subc__Enoplia(100);o__Triplonchida(100);s__Trichodorus_nanjingensis(84); |
| OTU_583 | 0 | 0 | 0 | 0 | 0 | 0 | 2 | 3 | 39 | 0 | 0 | 0 | All_GROUP:sk__Eukaryota(100);sknr0__Opisthokonta(100);sknr1__Holozoa(100);sknr2__Metazoa_(Animalia)(100);sknr3__Eumetazoa(100);sknr4__Bilateria(100);p__Nematoda(100);c__Enoplea(100);subc__Enoplia(100);o__Triplonchida(100);s__Tripylina_sp._SAN-2007a(100); |
| OTU_187 | 24 | 9 | 9 | 0 | 0 | 0 | 0 | 1 | 57 | 0 | 2 | 18 | All_GROUP:sk__Eukaryota(100);sknr0__Opisthokonta(100);sknr1__Holozoa(100);sknr2__Metazoa_(Animalia)(100);sknr3__Eumetazoa(100);sknr4__Bilateria(100);p__Nematoda(100);c__Enoplea(100);subc__Enoplia(100);o__Triplonchida(100);s__Tylolaimophorus_typicus(98); |
| OTU_261 | 0 | 87 | 1 | 4 | 0 | 0 | 1 | 1 | 11 | 4 | 3 | 5 | All_GROUP:sk__Eukaryota(100);sknr0__Opisthokonta(100);sknr1__Holozoa(100);sknr2__Metazoa_(Animalia)(100);sknr3__Eumetazoa(100);sknr4__Bilateria(100);p__Nematoda(100);c__Enoplea(100);subc__Enoplia(100);o__Enoplida(100);s__uncultured_Eimeriidae(87); |
| OTU_12 | 737 | 374 | 539 | 9 | 13 | 32 | 301 | 164 | 180 | 31 | 88 | 22 | All_GROUP:sk__Eukaryota(100);sknr0__Opisthokonta(100);sknr1__Holozoa(100);sknr2__Metazoa_(Animalia)(100);sknr3__Eumetazoa(100);sknr4__Bilateria(100);p__Nematoda(100);c__Enoplea(100);subc__Enoplia(100);o__Triplonchida(100); |
| OTU_266 | 0 | 43 | 2 | 4 | 2 | 0 | 10 | 1 | 7 | 0 | 0 | 0 | All_GROUP:sk__Eukaryota(100);sknr0__Opisthokonta(100);sknr1__Holozoa(100);sknr2__Metazoa_(Animalia)(100);sknr3__Eumetazoa(100);sknr4__Bilateria(100);p__Nematoda(100);c__Enoplea(100);subc__Enoplia(100);o__Triplonchida(100); |
| OTU_277 | 1 | 0 | 1 | 0 | 1 | 1 | 0 | 0 | 0 | 0 | 0 | 46 | All_GROUP:sk__Eukaryota(100);sknr0__Opisthokonta(100);sknr1__Holozoa(100);sknr2__Metazoa_(Animalia)(100);sknr3__Eumetazoa(100);sknr4__Bilateria(100);p__Nematoda(91);c__Enoplea(91);subc__Enoplia(91);o__Triplonchida(89); |
| OTU_110 | 12 | 1 | 0 | 1 | 0 | 0 | 20 | 31 | 14 | 0 | 0 | 0 | All_GROUP:sk__Eukaryota(100);sknr0__Opisthokonta(100);sknr1__Holozoa(100);sknr2__Metazoa_(Animalia)(100);sknr3__Eumetazoa(100);sknr4__Bilateria(100);p__Annelida(100);pnr0__Clitellata(100);subc__Oligochaeta(100);o__Haplotaxida(100); |
| OTU_614 | 8 | 2 | 4 | 0 | 0 | 0 | 3 | 10 | 18 | 0 | 1 | 2 | All_GROUP:sk__Eukaryota(100);sknr0__Opisthokonta(100);sknr1__Holozoa(100);sknr2__Metazoa_(Animalia)(100);sknr3__Eumetazoa(100);sknr4__Bilateria(100);p__Annelida(100);pnr0__Clitellata(99);subc__Oligochaeta(99);o__Haplotaxida(99); |
| OTU_21 | 27 | 11 | 31 | 17 | 0 | 2 | 25 | 512 | 129 | 132 | 14 | 171 | All_GROUP:sk__Eukaryota(100);sknr0__Opisthokonta(100);sknr1__Holozoa(100);sknr2__Metazoa_(Animalia)(100);sknr3__Eumetazoa(100);sknr4__Bilateria(100);p__Chordata(100);g__Vertebrata(100);gnr0__Gnathostomata(100);gnr1__Euteleostomi(100);gnr2__Tetrapoda(100);c__Mammalia(100); |
| OTU_53 | 0 | 0 | 0 | 0 | 1 | 0 | 229 | 167 | 9 | 0 | 0 | 0 | All_GROUP:sk__Eukaryota(100);sknr0__Opisthokonta(100);sknr1__Holozoa(100);sknr2__Metazoa_(Animalia)(100);sknr3__Eumetazoa(100);sknr4__Bilateria(100);p__Gastrotricha(99);o__Chaetonotida(99); |
| OTU_781 | 0 | 0 | 1 | 2 | 6 | 0 | 2 | 0 | 0 | 0 | 0 | 0 | All_GROUP:sk__Eukaryota(100);sknr0__Opisthokonta(100);sknr1__Holozoa(100);sknr2__Metazoa_(Animalia)(100);sknr3__Eumetazoa(100);sknr4__Bilateria(100);p__Platyhelminthes(98);c__Catenulida(98);f__Catenulidae(95); |
| OTU_354 | 5 | 19 | 19 | 2 | 1 | 2 | 1 | 4 | 12 | 7 | 1 | 10 | All_GROUP:sk__Eukaryota(100);sknr0__Opisthokonta(100);sknr1__Holozoa(100);sknr2__Metazoa_(Animalia)(100);sknr3__Eumetazoa(100);sknr4__Bilateria(100);p__Rotifera(100);c__Bdelloidea(100);o__Adinetida(100);s__uncultured_bdelloid_rotifer(97); |
| OTU_422 | 0 | 0 | 0 | 0 | 1 | 0 | 0 | 8 | 20 | 27 | 3 | 6 | All_GROUP:sk__Eukaryota(100);sknr0__Opisthokonta(100);sknr1__Holozoa(100);sknr2__Metazoa_(Animalia)(100);sknr3__Eumetazoa(100);sknr4__Bilateria(100);p__Rotifera(100);c__Bdelloidea(100);o__Adinetida(100);s__uncultured_bdelloid_rotifer(100); |
| OTU_487 | 29 | 0 | 0 | 0 | 0 | 0 | 0 | 0 | 0 | 0 | 0 | 0 | All_GROUP:sk__Eukaryota(100);sknr0__Opisthokonta(100);sknr1__Holozoa(100);sknr2__Metazoa_(Animalia)(100);sknr3__Eumetazoa(100);sknr4__Bilateria(100);p__Rotifera(100);c__Bdelloidea(100);o__Adinetida(97);s__uncultured_bdelloid_rotifer(88); |
| OTU_553 | 0 | 4 | 42 | 0 | 0 | 0 | 3 | 1 | 0 | 19 | 0 | 7 | All_GROUP:sk__Eukaryota(100);sknr0__Opisthokonta(100);sknr1__Holozoa(100);sknr2__Metazoa_(Animalia)(100);sknr3__Eumetazoa(100);sknr4__Bilateria(100);p__Rotifera(100);c__Bdelloidea(100);o__Adinetida(94);s__uncultured_bdelloid_rotifer(88); |
| OTU_593 | 0 | 0 | 0 | 0 | 0 | 0 | 0 | 6 | 1 | 3 | 0 | 0 | All_GROUP:sk__Eukaryota(100);sknr0__Opisthokonta(100);sknr1__Holozoa(100);sknr2__Metazoa_(Animalia)(100);sknr3__Eumetazoa(100);sknr4__Bilateria(100);p__Rotifera(100);c__Bdelloidea(100);o__Adinetida(92);s__uncultured_bdelloid_rotifer(88); |
| OTU_608 | 0 | 7 | 31 | 0 | 0 | 1 | 20 | 6 | 0 | 0 | 0 | 0 | All_GROUP:sk__Eukaryota(100);sknr0__Opisthokonta(100);sknr1__Holozoa(100);sknr2__Metazoa_(Animalia)(100);sknr3__Eumetazoa(100);sknr4__Bilateria(100);p__Rotifera(100);c__Bdelloidea(100);o__Adinetida(100);s__uncultured_bdelloid_rotifer(100); |
| OTU_885 | 19 | 0 | 0 | 0 | 0 | 0 | 0 | 0 | 0 | 4 | 0 | 1 | All_GROUP:sk__Eukaryota(100);sknr0__Opisthokonta(100);sknr1__Holozoa(100);sknr2__Metazoa_(Animalia)(100);sknr3__Eumetazoa(100);sknr4__Bilateria(100);p__Rotifera(100);c__Bdelloidea(100);o__Adinetida(91);s__uncultured_bdelloid_rotifer(91); |
| OTU_913 | 0 | 0 | 0 | 0 | 1 | 0 | 8 | 0 | 0 | 0 | 0 | 17 | All_GROUP:sk__Eukaryota(100);sknr0__Opisthokonta(100);sknr1__Holozoa(100);sknr2__Metazoa_(Animalia)(100);sknr3__Eumetazoa(100);sknr4__Bilateria(100);p__Rotifera(100);c__Bdelloidea(100);o__Adinetida(99);s__uncultured_bdelloid_rotifer(90); |
| OTU_100 | 24 | 62 | 87 | 39 | 14 | 20 | 9 | 87 | 70 | 29 | 121 | 93 | All_GROUP:sk__Eukaryota(100);sknr0__Opisthokonta(100);sknr1__Holozoa(100);sknr2__Metazoa_(Animalia)(100);sknr3__Eumetazoa(100);sknr4__Bilateria(100);p__Rotifera(100);c__Bdelloidea(100);o__Adinetida(92); |
| OTU_246 | 0 | 0 | 0 | 0 | 0 | 2 | 51 | 0 | 0 | 0 | 0 | 0 | All_GROUP:sk__Eukaryota(100);sknr0__Opisthokonta(100);sknr1__Holozoa(100);sknr2__Metazoa_(Animalia)(100);sknr3__Eumetazoa(100);sknr4__Bilateria(100);p__Rotifera(100);c__Monogononta(100);o__Ploimida(88); |
| OTU_129 | 0 | 0 | 0 | 9 | 1 | 129 | 0 | 0 | 0 | 0 | 0 | 0 | All_GROUP:sk__Eukaryota(100);sknr0__Opisthokonta(100);sknr1__Holozoa(100);sknr2__Metazoa_(Animalia)(100);sknr3__Eumetazoa(100);sknr4__Bilateria(100);p__Tardigrada(100);c__Eutardigrada(100);o__Parachela(100); |
| OTU_465 | 0 | 0 | 0 | 0 | 3 | 2 | 0 | 0 | 0 | 0 | 0 | 0 | All_GROUP:sk__Eukaryota(100);sknr0__Opisthokonta(100);sknr1__Holozoa(100);sknr2__Metazoa_(Animalia)(100);sknr3__Eumetazoa(100);sknr4__Bilateria(100);p__Tardigrada(100);c__Eutardigrada(100);o__Parachela(100); |
| OTU_1047 | 0 | 0 | 0 | 0 | 0 | 0 | 0 | 0 | 0 | 3 | 0 | 4 | All_GROUP:sk__Eukaryota(100);sknr0__Opisthokonta(100);sknr1__Holozoa(100);sknr2__Metazoa_(Animalia)(100);sknr3__Eumetazoa(100);sknr4__Bilateria(100);p__Tardigrada(100);c__Eutardigrada(100);o__Parachela(100); |
| OTU_69 | 0 | 0 | 0 | 0 | 0 | 0 | 322 | 9 | 127 | 16 | 0 | 1 | All_GROUP:sk__Eukaryota(100);sknr0__Opisthokonta(80); |
| OTU_111 | 6 | 0 | 205 | 0 | 0 | 0 | 1 | 0 | 14 | 70 | 0 | 0 | All_GROUP:sk__Eukaryota(100);sknr0__Opisthokonta(83); |
| OTU_148 | 2 | 0 | 0 | 0 | 0 | 0 | 48 | 190 | 78 | 29 | 9 | 8 | All_GROUP:sk__Eukaryota(100);sknr0__Opisthokonta(83); |
| OTU_244 | 47 | 0 | 0 | 0 | 0 | 0 | 0 | 0 | 0 | 0 | 0 | 0 | All_GROUP:sk__Eukaryota(100);sknr0__Opisthokonta(94); |
| OTU_322 | 85 | 3 | 0 | 0 | 0 | 0 | 26 | 2 | 3 | 34 | 1 | 4 | All_GROUP:sk__Eukaryota(100);sknr0__Opisthokonta(86); |
| OTU_343 | 1 | 44 | 5 | 0 | 0 | 0 | 27 | 11 | 0 | 125 | 2 | 0 | All_GROUP:sk__Eukaryota(100);sknr0__Opisthokonta(81); |
| OTU_369 | 0 | 0 | 5 | 24 | 0 | 0 | 0 | 0 | 0 | 0 | 0 | 0 | All_GROUP:sk__Eukaryota(100);sknr0__Opisthokonta(95); |
| OTU_425 | 4 | 0 | 1 | 0 | 0 | 0 | 21 | 10 | 5 | 12 | 0 | 0 | All_GROUP:sk__Eukaryota(100);sknr0__Opisthokonta(80); |
| OTU_528 | 0 | 0 | 13 | 0 | 0 | 0 | 0 | 0 | 0 | 0 | 0 | 0 | All_GROUP:sk__Eukaryota(100);sknr0__Opisthokonta(84); |
| OTU_538 | 0 | 2 | 0 | 0 | 0 | 0 | 0 | 7 | 5 | 1 | 0 | 0 | All_GROUP:sk__Eukaryota(100);sknr0__Opisthokonta(81); |
| OTU_698 | 9 | 4 | 0 | 0 | 0 | 0 | 8 | 0 | 3 | 0 | 0 | 0 | All_GROUP:sk__Eukaryota(100);sknr0__Opisthokonta(81); |
| OTU_782 | 0 | 0 | 4 | 0 | 0 | 0 | 9 | 0 | 3 | 0 | 0 | 0 | All_GROUP:sk__Eukaryota(100);sknr0__Opisthokonta(84); |
| OTU_787 | 8 | 3 | 20 | 0 | 0 | 0 | 8 | 4 | 5 | 24 | 9 | 7 | All_GROUP:sk__Eukaryota(100);sknr0__Opisthokonta(82); |
| OTU_817 | 0 | 0 | 0 | 0 | 0 | 0 | 7 | 2 | 9 | 0 | 0 | 0 | All_GROUP:sk__Eukaryota(100);sknr0__Opisthokonta(85); |
| OTU_857 | 0 | 0 | 3 | 0 | 0 | 0 | 0 | 0 | 0 | 0 | 0 | 0 | All_GROUP:sk__Eukaryota(100);sknr0__Opisthokonta(87); |
| OTU_918 | 0 | 0 | 0 | 1 | 0 | 0 | 5 | 0 | 0 | 0 | 0 | 0 | All_GROUP:sk__Eukaryota(100);sknr0__Opisthokonta(81); |
| OTU_944 | 16 | 1 | 2 | 0 | 0 | 0 | 18 | 0 | 0 | 0 | 0 | 0 | All_GROUP:sk__Eukaryota(100);sknr0__Opisthokonta(87); |
| OTU_977 | 0 | 0 | 0 | 0 | 0 | 0 | 3 | 7 | 2 | 0 | 0 | 0 | All_GROUP:sk__Eukaryota(100);sknr0__Opisthokonta(93); |
| OTU_1012 | 0 | 2 | 0 | 5 | 0 | 0 | 0 | 0 | 0 | 0 | 1 | 0 | All_GROUP:sk__Eukaryota(100);sknr0__Opisthokonta(94); |
| OTU_759 | 0 | 0 | 0 | 0 | 0 | 0 | 0 | 2 | 0 | 2 | 3 | 3 | All_GROUP:sk__Eukaryota(100);sknr0__Opisthokonta(99);sknr1__Holozoa(83); |
| OTU_978 | 0 | 7 | 0 | 0 | 0 | 0 | 0 | 0 | 0 | 2 | 0 | 0 | All_GROUP:sk__Eukaryota(100);sknr0__Opisthokonta(100);sknr1__Holozoa(100);sknr2__Choanomonada(100);sknr3__Craspedida(100);f__Salpingoecidae(100);s__uncultured_freshwater_eukaryote(85); |
| OTU_341 | 5 | 1 | 13 | 0 | 0 | 0 | 1 | 1 | 2 | 1 | 1 | 29 | All_GROUP:sk__Eukaryota(100);sknr0__Opisthokonta(99);sknr1__Holozoa(99);sknr2__Choanomonada(99);sknr3__Craspedida(99);f__Codonosigidae(94);g__Monosiga(94); |
| OTU_745 | 0 | 0 | 0 | 0 | 0 | 0 | 29 | 1 | 13 | 0 | 0 | 0 | All_GROUP:sk__Eukaryota(100);sknr0__Opisthokonta(100);sknr1__Holozoa(94);sknr2__Choanomonada(89);sknr3__Craspedida(85); |
| OTU_447 | 3 | 5 | 7 | 4 | 0 | 1 | 9 | 2 | 3 | 3 | 1 | 1 | All_GROUP:sk__Eukaryota(100);sknr0__Opisthokonta(100);sknr1__Holozoa(100);sknr2__Choanomonada(100);sknr3__Craspedida(100);f__Salpingoecidae(100);fnr0__Freshwater_Choanoflagellates_2(100);s__uncultured_freshwater_eukaryote(100); |
| OTU_592 | 0 | 0 | 9 | 0 | 0 | 0 | 1 | 0 | 2 | 0 | 0 | 0 | All_GROUP:sk__Eukaryota(100);sknr0__Opisthokonta(100);sknr1__Holozoa(99);c__Ichthyosporea(98);cnr0__Ichthyophonae(98);cnr1__Ichthyophonidae(90); |
| OTU_605 | 11 | 4 | 11 | 0 | 0 | 0 | 8 | 0 | 5 | 4 | 5 | 14 | All_GROUP:sk__Eukaryota(100);sknr0__Opisthokonta(100);sknr1__Holozoa(100);c__Ichthyosporea(100);cnr0__Ichthyophonae(100);cnr1__Pseudoperkinsidae(85); |
| OTU_274 | 35 | 6 | 14 | 0 | 0 | 3 | 4 | 2 | 7 | 1 | 0 | 0 | All_GROUP:sk__Eukaryota(100);sknr0__Opisthokonta(100);sknr1__Holozoa(100);c__Ichthyosporea(100);cnr0__Ichthyophonae(100);cnr1__Pseudoperkinsidae(98);cun2__uncultured_eukaryote(92); |
| OTU_211 | 0 | 0 | 0 | 0 | 0 | 0 | 64 | 0 | 0 | 0 | 0 | 0 | All_GROUP:sk__Eukaryota(100);sknr0__Opisthokonta(85);sknr1__Holozoa(81);sknr2__Metazoa_(Animalia)(81);sknr3__Eumetazoa(80); |
| OTU_113 | 0 | 1 | 0 | 0 | 0 | 0 | 76 | 0 | 57 | 0 | 0 | 0 | All_GROUP:sk__Eukaryota(100);sknr0__Opisthokonta(97);sknr1__Holozoa(95);sknr2__Metazoa_(Animalia)(95);sknr3__Eumetazoa(95);sknr4__Bilateria(94); |
| OTU_541 | 0 | 0 | 0 | 0 | 0 | 0 | 66 | 1 | 1 | 0 | 0 | 0 | All_GROUP:sk__Eukaryota(100);sknr0__Opisthokonta(97);sknr1__Holozoa(96);sknr2__Metazoa_(Animalia)(96);sknr3__Eumetazoa(96);sknr4__Bilateria(92); |
| OTU_919 | 0 | 0 | 0 | 0 | 0 | 0 | 1 | 0 | 0 | 29 | 0 | 0 | All_GROUP:sk__Eukaryota(100);sknr0__Opisthokonta(90);sknr1__Holozoa(87);sknr2__Metazoa_(Animalia)(87);sknr3__Eumetazoa(86);sknr4__Bilateria(84); |
| OTU_1024 | 0 | 0 | 0 | 0 | 0 | 0 | 0 | 0 | 0 | 0 | 17 | 1 | All_GROUP:sk__Eukaryota(100);sknr0__Opisthokonta(100);sknr1__Holozoa(100);sknr2__Metazoa_(Animalia)(100);sknr3__Eumetazoa(100);sknr4__Bilateria(100); |
| OTU_1060 | 0 | 0 | 0 | 0 | 0 | 0 | 0 | 0 | 0 | 0 | 0 | 9 | All_GROUP:sk__Eukaryota(100);sknr0__Opisthokonta(100);sknr1__Holozoa(100);sknr2__Metazoa_(Animalia)(100);sknr3__Eumetazoa(100);sknr4__Bilateria(100); |
| OTU_617 | 2 | 7 | 3 | 2 | 0 | 9 | 5 | 1 | 6 | 0 | 0 | 0 | All_GROUP:sk__Eukaryota(100);sknr0__Opisthokonta(95);sknr1__Nucletmycea(82); |
| OTU_854 | 2 | 0 | 0 | 0 | 0 | 0 | 0 | 0 | 9 | 0 | 0 | 0 | All_GROUP:sk__Eukaryota(100);sknr0__Opisthokonta(100);sknr1__Nucletmycea(94);sknr2__Discicristoidea(92);sknr3__Incertae_Sedis(92); |
| OTU_469 | 1 | 3 | 5 | 0 | 0 | 0 | 2 | 0 | 1 | 2 | 0 | 14 | All_GROUP:sk__Eukaryota(100);sknr0__Opisthokonta(100);sknr1__uncultured(99);s__uncultured_Eimeriidae(99); |
| OTU_446 | 7 | 16 | 13 | 10 | 7 | 9 | 4 | 5 | 4 | 0 | 0 | 0 | All_GROUP:sk__Eukaryota(100);sknr0__SAR(100);sknr1__Rhizaria(100);sknr2__Cercozoa(100);f__Cercomonadidae(100);g__Cercomonas(100);s__Cercomonas_plasmodialis(98); |
| OTU_415 | 0 | 0 | 0 | 6 | 4 | 7 | 0 | 2 | 22 | 0 | 0 | 0 | All_GROUP:sk__Eukaryota(100);sknr0__SAR(100);sknr1__Rhizaria(100);sknr2__Cercozoa(100);f__Cercomonadidae(100);g__Cercomonas(100);s__Cercomonas_sp._HFCC_901(99); |
| OTU_89 | 64 | 24 | 51 | 10 | 6 | 11 | 34 | 9 | 12 | 37 | 9 | 13 | All_GROUP:sk__Eukaryota(100);sknr0__SAR(100);sknr1__Rhizaria(100);sknr2__Cercozoa(100);f__Cercomonadidae(100);g__Cercomonas(100);s__Cercomonas_sp._HFCC_903(97); |
| OTU_663 | 8 | 3 | 1 | 2 | 6 | 0 | 5 | 3 | 0 | 6 | 1 | 5 | All_GROUP:sk__Eukaryota(100);sknr0__SAR(100);sknr1__Rhizaria(100);sknr2__Cercozoa(100);f__Cercomonadidae(100);g__Cercomonas(100);s__Cercomonas_sp._HFCC_906(95); |
| OTU_739 | 0 | 4 | 30 | 2 | 3 | 2 | 2 | 3 | 7 | 2 | 1 | 1 | All_GROUP:sk__Eukaryota(100);sknr0__SAR(100);sknr1__Rhizaria(100);sknr2__Cercozoa(100);f__Cercomonadidae(100);g__Cercomonas(100);s__Cercomonas_sp._SmallSA(99); |
| OTU_850 | 9 | 0 | 0 | 1 | 0 | 0 | 0 | 0 | 0 | 0 | 0 | 0 | All_GROUP:sk__Eukaryota(100);sknr0__SAR(100);sknr1__Rhizaria(100);sknr2__Cercozoa(100);f__Cercomonadidae(98);g__Cercomonas(98);s__Cercomonas_sp._Tempisque(82); |
| OTU_641 | 2 | 0 | 0 | 0 | 2 | 1 | 15 | 7 | 0 | 0 | 0 | 0 | All_GROUP:sk__Eukaryota(100);sknr0__SAR(100);sknr1__Rhizaria(100);sknr2__Cercozoa(100);f__Cercomonadidae(100);g__Cercomonas(100);s__Cercozoa_sp._ATCC_50407(99); |
| OTU_372 | 17 | 11 | 12 | 5 | 5 | 6 | 4 | 0 | 1 | 1 | 5 | 7 | All_GROUP:sk__Eukaryota(100);sknr0__SAR(100);sknr1__Rhizaria(100);sknr2__Cercozoa(100);f__Cercomonadidae(100);g__Eocercomonas(100);s__Eocercomonas_sp._HFCC_907(100); |
| OTU_613 | 14 | 0 | 0 | 0 | 0 | 1 | 1 | 0 | 3 | 0 | 0 | 0 | All_GROUP:sk__Eukaryota(100);sknr0__SAR(100);sknr1__Rhizaria(100);sknr2__Cercozoa(100);f__Cercomonadidae(100);s__Eocercomonas_sp._HFCC_908(100); |
| OTU_373 | 13 | 4 | 13 | 1 | 1 | 2 | 0 | 0 | 19 | 0 | 0 | 0 | All_GROUP:sk__Eukaryota(100);sknr0__SAR(100);sknr1__Rhizaria(100);sknr2__Cercozoa(100);f__Cercomonadidae(100);s__Eocercomonas_sp._HFCC_909(93); |
| OTU_956 | 1 | 0 | 2 | 0 | 0 | 0 | 10 | 0 | 5 | 2 | 0 | 0 | All_GROUP:sk__Eukaryota(100);sknr0__SAR(100);sknr1__Rhizaria(100);sknr2__Cercozoa(100);f__Cercomonadidae(100);g__Cercomonas(100);s__Nucleocercomonas_sp._HFCC_921(100); |
| OTU_491 | 5 | 0 | 1 | 0 | 0 | 0 | 11 | 4 | 19 | 0 | 0 | 0 | All_GROUP:sk__Eukaryota(100);sknr0__SAR(100);sknr1__Rhizaria(100);sknr2__Cercozoa(100);f__Cercomonadidae(99);g__Cercomonas(99);s__Paracercomonas_oxoniensis(87); |
| OTU_239 | 67 | 14 | 10 | 0 | 0 | 0 | 14 | 9 | 24 | 18 | 4 | 7 | All_GROUP:sk__Eukaryota(100);sknr0__SAR(100);sknr1__Rhizaria(100);sknr2__Cercozoa(100);f__Cercomonadidae(97);g__Cercomonas(97);s__uncultured_cercomonad(92); |
| OTU_631 | 16 | 0 | 4 | 3 | 0 | 0 | 0 | 7 | 0 | 2 | 2 | 0 | All_GROUP:sk__Eukaryota(100);sknr0__SAR(100);sknr1__Rhizaria(100);sknr2__Cercozoa(100);f__Cercomonadidae(95);g__Cercomonas(95);s__uncultured_cercomonad(93); |
| OTU_448 | 5 | 12 | 2 | 0 | 1 | 2 | 0 | 0 | 1 | 0 | 0 | 0 | All_GROUP:sk__Eukaryota(100);sknr0__SAR(100);sknr1__Rhizaria(100);sknr2__Cercozoa(100);f__Cercomonadidae(100);g__Cercomonas(100);s__uncultured_cercozoan(100); |
| OTU_633 | 8 | 12 | 2 | 0 | 0 | 1 | 0 | 0 | 0 | 0 | 0 | 0 | All_GROUP:sk__Eukaryota(100);sknr0__SAR(100);sknr1__Rhizaria(100);sknr2__Cercozoa(100);f__Cercomonadidae(100);g__Cercomonas(100);s__uncultured_cercozoan(97); |
| OTU_695 | 4 | 0 | 2 | 0 | 0 | 0 | 0 | 2 | 16 | 0 | 0 | 0 | All_GROUP:sk__Eukaryota(100);sknr0__SAR(100);sknr1__Rhizaria(100);sknr2__Cercozoa(100);f__Cercomonadidae(93);g__Cercomonas(93);s__uncultured_cercozoan(87); |
| OTU_999 | 3 | 6 | 0 | 0 | 2 | 1 | 0 | 0 | 0 | 0 | 1 | 0 | All_GROUP:sk__Eukaryota(100);sknr0__SAR(100);sknr1__Rhizaria(100);sknr2__Cercozoa(100);f__Cercomonadidae(100);g__Cercomonas(100);s__uncultured_cercozoan(100); |
| OTU_474 | 28 | 0 | 0 | 0 | 0 | 0 | 0 | 2 | 9 | 11 | 0 | 0 | All_GROUP:sk__Eukaryota(100);sknr0__SAR(100);sknr1__Rhizaria(100);sknr2__Cercozoa(100);f__Cercomonadidae(100);g__Cercomonas(100);s__uncultured_Eimeriidae(100); |
| OTU_669 | 3 | 3 | 1 | 0 | 0 | 2 | 2 | 0 | 4 | 6 | 3 | 5 | All_GROUP:sk__Eukaryota(100);sknr0__SAR(100);sknr1__Rhizaria(100);sknr2__Cercozoa(100);f__Cercomonadidae(99);g__Cercomonas(99);s__uncultured_Eimeriidae(80); |
| OTU_1053 | 12 | 0 | 1 | 0 | 0 | 0 | 3 | 0 | 0 | 0 | 0 | 0 | All_GROUP:sk__Eukaryota(100);sknr0__SAR(100);sknr1__Rhizaria(100);sknr2__Cercozoa(100);f__Cercomonadidae(100);g__Cercomonas(100);s__uncultured_Eimeriidae(100); |
| OTU_105 | 39 | 24 | 10 | 17 | 16 | 17 | 16 | 6 | 54 | 18 | 5 | 19 | All_GROUP:sk__Eukaryota(100);sknr0__SAR(100);sknr1__Rhizaria(100);sknr2__Cercozoa(100);f__Cercomonadidae(92);g__Cercomonas(92); |
| OTU_135 | 8 | 16 | 4 | 0 | 0 | 0 | 4 | 0 | 1 | 3 | 6 | 22 | All_GROUP:sk__Eukaryota(100);sknr0__SAR(100);sknr1__Rhizaria(100);sknr2__Cercozoa(100);f__Cercomonadidae(90);g__Cercomonas(90); |
| OTU_137 | 79 | 14 | 21 | 12 | 14 | 6 | 2 | 1 | 2 | 0 | 0 | 0 | All_GROUP:sk__Eukaryota(100);sknr0__SAR(100);sknr1__Rhizaria(100);sknr2__Cercozoa(100);f__Cercomonadidae(100);g__Cercomonas(100); |
| OTU_154 | 59 | 12 | 12 | 1 | 1 | 1 | 2 | 18 | 9 | 3 | 8 | 2 | All_GROUP:sk__Eukaryota(100);sknr0__SAR(100);sknr1__Rhizaria(100);sknr2__Cercozoa(100);f__Cercomonadidae(99);g__Cercomonas(99); |
| OTU_168 | 17 | 5 | 1 | 0 | 5 | 5 | 0 | 0 | 0 | 0 | 0 | 0 | All_GROUP:sk__Eukaryota(100);sknr0__SAR(100);sknr1__Rhizaria(100);sknr2__Cercozoa(100);f__Cercomonadidae(100);g__Cercomonas(100); |
| OTU_184 | 29 | 28 | 37 | 2 | 0 | 1 | 10 | 1 | 1 | 2 | 0 | 12 | All_GROUP:sk__Eukaryota(100);sknr0__SAR(100);sknr1__Rhizaria(100);sknr2__Cercozoa(100);f__Cercomonadidae(100);g__Cercomonas(100); |
| OTU_189 | 7 | 1 | 1 | 0 | 1 | 2 | 10 | 21 | 14 | 0 | 0 | 0 | All_GROUP:sk__Eukaryota(100);sknr0__SAR(100);sknr1__Rhizaria(100);sknr2__Cercozoa(100);f__Cercomonadidae(94);g__Cercomonas(94); |
| OTU_195 | 4 | 1 | 11 | 0 | 0 | 0 | 11 | 13 | 10 | 2 | 3 | 26 | All_GROUP:sk__Eukaryota(100);sknr0__SAR(100);sknr1__Rhizaria(100);sknr2__Cercozoa(100);f__Cercomonadidae(97);g__Cercomonas(82); |
| OTU_234 | 1 | 15 | 11 | 1 | 1 | 1 | 0 | 0 | 0 | 0 | 0 | 16 | All_GROUP:sk__Eukaryota(100);sknr0__SAR(100);sknr1__Rhizaria(100);sknr2__Cercozoa(100);f__Cercomonadidae(89);g__Cercomonas(89); |
| OTU_328 | 11 | 16 | 29 | 1 | 1 | 0 | 1 | 2 | 4 | 1 | 0 | 0 | All_GROUP:sk__Eukaryota(100);sknr0__SAR(100);sknr1__Rhizaria(100);sknr2__Cercozoa(100);f__Cercomonadidae(99);g__Cercomonas(99); |
| OTU_361 | 4 | 1 | 3 | 1 | 0 | 0 | 11 | 12 | 18 | 3 | 6 | 3 | All_GROUP:sk__Eukaryota(100);sknr0__SAR(100);sknr1__Rhizaria(99);sknr2__Cercozoa(99);f__Cercomonadidae(91);g__Cercomonas(88); |
| OTU_367 | 14 | 3 | 4 | 4 | 1 | 0 | 2 | 15 | 16 | 13 | 8 | 0 | All_GROUP:sk__Eukaryota(100);sknr0__SAR(100);sknr1__Rhizaria(100);sknr2__Cercozoa(100);f__Cercomonadidae(100);g__Cercomonas(100); |
| OTU_371 | 17 | 14 | 3 | 0 | 0 | 0 | 0 | 0 | 0 | 2 | 1 | 9 | All_GROUP:sk__Eukaryota(100);sknr0__SAR(100);sknr1__Rhizaria(100);sknr2__Cercozoa(100);f__Cercomonadidae(88);g__Cercomonas(88); |
| OTU_384 | 11 | 2 | 0 | 0 | 1 | 0 | 24 | 1 | 18 | 20 | 7 | 7 | All_GROUP:sk__Eukaryota(100);sknr0__SAR(100);sknr1__Rhizaria(100);sknr2__Cercozoa(100);f__Cercomonadidae(100);g__Cercomonas(100); |
| OTU_429 | 0 | 0 | 0 | 0 | 0 | 0 | 0 | 8 | 9 | 0 | 0 | 0 | All_GROUP:sk__Eukaryota(100);sknr0__SAR(100);sknr1__Rhizaria(100);sknr2__Cercozoa(100);f__Cercomonadidae(99);g__Cercomonas(97); |
| OTU_438 | 0 | 1 | 0 | 0 | 0 | 0 | 5 | 2 | 24 | 2 | 1 | 4 | All_GROUP:sk__Eukaryota(100);sknr0__SAR(100);sknr1__Rhizaria(100);sknr2__Cercozoa(100);f__Cercomonadidae(100);g__Cercomonas(100); |
| OTU_445 | 1 | 12 | 0 | 0 | 0 | 0 | 3 | 0 | 5 | 0 | 0 | 0 | All_GROUP:sk__Eukaryota(100);sknr0__SAR(100);sknr1__Rhizaria(100);sknr2__Cercozoa(100);f__Cercomonadidae(100);g__Cercomonas(100); |
| OTU_476 | 0 | 10 | 0 | 0 | 0 | 0 | 2 | 0 | 4 | 3 | 6 | 0 | All_GROUP:sk__Eukaryota(100);sknr0__SAR(100);sknr1__Rhizaria(100);sknr2__Cercozoa(100);f__Cercomonadidae(97);g__Cercomonas(97); |
| OTU_509 | 2 | 2 | 1 | 2 | 0 | 0 | 2 | 0 | 23 | 0 | 0 | 1 | All_GROUP:sk__Eukaryota(100);sknr0__SAR(100);sknr1__Rhizaria(100);sknr2__Cercozoa(100);f__Cercomonadidae(93);g__Cercomonas(93); |
| OTU_552 | 4 | 7 | 1 | 3 | 0 | 3 | 2 | 4 | 0 | 1 | 3 | 0 | All_GROUP:sk__Eukaryota(100);sknr0__SAR(100);sknr1__Rhizaria(100);sknr2__Cercozoa(100);f__Cercomonadidae(90);g__Cercomonas(90); |
| OTU_560 | 0 | 6 | 0 | 5 | 1 | 16 | 0 | 0 | 0 | 0 | 0 | 0 | All_GROUP:sk__Eukaryota(100);sknr0__SAR(100);sknr1__Rhizaria(100);sknr2__Cercozoa(100);f__Cercomonadidae(81);g__Cercomonas(81); |
| OTU_579 | 13 | 1 | 8 | 0 | 1 | 1 | 0 | 0 | 0 | 0 | 0 | 0 | All_GROUP:sk__Eukaryota(100);sknr0__SAR(100);sknr1__Rhizaria(100);sknr2__Cercozoa(100);f__Cercomonadidae(98);g__Cercomonas(98); |
| OTU_590 | 20 | 7 | 5 | 3 | 1 | 1 | 3 | 16 | 0 | 0 | 0 | 0 | All_GROUP:sk__Eukaryota(100);sknr0__SAR(100);sknr1__Rhizaria(100);sknr2__Cercozoa(100);f__Cercomonadidae(98);g__Cercomonas(98); |
| OTU_689 | 7 | 1 | 1 | 0 | 0 | 0 | 0 | 0 | 3 | 4 | 0 | 0 | All_GROUP:sk__Eukaryota(100);sknr0__SAR(100);sknr1__Rhizaria(98);sknr2__Cercozoa(98);f__Cercomonadidae(83);g__Cercomonas(83); |
| OTU_721 | 0 | 0 | 0 | 0 | 0 | 0 | 3 | 0 | 24 | 0 | 0 | 3 | All_GROUP:sk__Eukaryota(100);sknr0__SAR(100);sknr1__Rhizaria(100);sknr2__Cercozoa(100);f__Cercomonadidae(83);g__Cercomonas(83); |
| OTU_801 | 5 | 13 | 8 | 1 | 1 | 0 | 0 | 0 | 0 | 0 | 1 | 6 | All_GROUP:sk__Eukaryota(100);sknr0__SAR(100);sknr1__Rhizaria(100);sknr2__Cercozoa(100);f__Cercomonadidae(100);g__Cercomonas(100); |
| OTU_804 | 1 | 14 | 0 | 0 | 0 | 0 | 3 | 2 | 0 | 1 | 0 | 1 | All_GROUP:sk__Eukaryota(100);sknr0__SAR(100);sknr1__Rhizaria(100);sknr2__Cercozoa(100);f__Cercomonadidae(86);g__Cercomonas(86); |
| OTU_860 | 0 | 0 | 0 | 0 | 6 | 0 | 0 | 0 | 0 | 4 | 2 | 1 | All_GROUP:sk__Eukaryota(100);sknr0__SAR(100);sknr1__Rhizaria(100);sknr2__Cercozoa(100);f__Cercomonadidae(83);g__Cercomonas(83); |
| OTU_866 | 6 | 6 | 8 | 4 | 1 | 3 | 0 | 0 | 0 | 0 | 0 | 0 | All_GROUP:sk__Eukaryota(100);sknr0__SAR(100);sknr1__Rhizaria(100);sknr2__Cercozoa(100);f__Cercomonadidae(100);g__Cercomonas(100); |
| OTU_897 | 6 | 15 | 16 | 0 | 2 | 0 | 0 | 0 | 1 | 1 | 0 | 0 | All_GROUP:sk__Eukaryota(100);sknr0__SAR(100);sknr1__Rhizaria(100);sknr2__Cercozoa(100);f__Cercomonadidae(94);g__Cercomonas(93); |
| OTU_923 | 0 | 4 | 13 | 2 | 2 | 1 | 0 | 0 | 15 | 0 | 0 | 1 | All_GROUP:sk__Eukaryota(100);sknr0__SAR(100);sknr1__Rhizaria(100);sknr2__Cercozoa(100);f__Cercomonadidae(91);g__Cercomonas(91); |
| OTU_929 | 0 | 1 | 0 | 0 | 0 | 0 | 4 | 1 | 2 | 1 | 4 | 3 | All_GROUP:sk__Eukaryota(100);sknr0__SAR(100);sknr1__Rhizaria(100);sknr2__Cercozoa(100);f__Cercomonadidae(99);g__Cercomonas(99); |
| OTU_942 | 0 | 0 | 5 | 0 | 0 | 0 | 1 | 4 | 0 | 4 | 0 | 1 | All_GROUP:sk__Eukaryota(100);sknr0__SAR(100);sknr1__Rhizaria(100);sknr2__Cercozoa(100);f__Cercomonadidae(85);g__Cercomonas(85); |
| OTU_949 | 8 | 0 | 3 | 0 | 0 | 1 | 0 | 14 | 1 | 5 | 3 | 19 | All_GROUP:sk__Eukaryota(100);sknr0__SAR(100);sknr1__Rhizaria(100);sknr2__Cercozoa(100);f__Cercomonadidae(100);g__Cercomonas(100); |
| OTU_964 | 0 | 0 | 2 | 0 | 0 | 0 | 0 | 0 | 0 | 4 | 0 | 0 | All_GROUP:sk__Eukaryota(100);sknr0__SAR(100);sknr1__Rhizaria(100);sknr2__Cercozoa(100);f__Cercomonadidae(99);g__Cercomonas(99); |
| OTU_965 | 6 | 0 | 0 | 0 | 0 | 0 | 0 | 15 | 5 | 0 | 0 | 0 | All_GROUP:sk__Eukaryota(100);sknr0__SAR(100);sknr1__Rhizaria(100);sknr2__Cercozoa(100);f__Cercomonadidae(85);g__Cercomonas(85); |
| OTU_966 | 0 | 1 | 0 | 0 | 0 | 0 | 3 | 9 | 3 | 0 | 0 | 0 | All_GROUP:sk__Eukaryota(100);sknr0__SAR(100);sknr1__Rhizaria(100);sknr2__Cercozoa(100);f__Cercomonadidae(88);g__Cercomonas(88); |
| OTU_1034 | 0 | 3 | 0 | 1 | 1 | 0 | 0 | 0 | 0 | 0 | 0 | 0 | All_GROUP:sk__Eukaryota(100);sknr0__SAR(100);sknr1__Rhizaria(100);sknr2__Cercozoa(100);f__Cercomonadidae(100);g__Cercomonas(100); |
| OTU_1077 | 0 | 0 | 0 | 4 | 0 | 0 | 0 | 0 | 0 | 13 | 0 | 2 | All_GROUP:sk__Eukaryota(100);sknr0__SAR(100);sknr1__Rhizaria(100);sknr2__Cercozoa(100);f__Cercomonadidae(99);g__Cercomonas(99); |
| OTU_392 | 1 | 0 | 1 | 0 | 1 | 0 | 2 | 9 | 4 | 20 | 2 | 19 | All_GROUP:sk__Eukaryota(100);sknr0__SAR(100);sknr1__Rhizaria(100);sknr2__Cercozoa(100);f__Cercomonadidae(96);g__Eocercomonas(84); |
| OTU_104 | 10 | 17 | 40 | 1 | 2 | 0 | 11 | 25 | 0 | 15 | 2 | 18 | All_GROUP:sk__Eukaryota(100);sknr0__SAR(100);sknr1__Rhizaria(100);sknr2__Cercozoa(100);f__Cercomonadidae(83); |
| OTU_423 | 0 | 5 | 0 | 0 | 0 | 0 | 0 | 0 | 0 | 1 | 0 | 0 | All_GROUP:sk__Eukaryota(100);sknr0__SAR(100);sknr1__Rhizaria(100);sknr2__Cercozoa(100);f__Cercomonadidae(84); |
| OTU_718 | 4 | 1 | 0 | 0 | 0 | 0 | 2 | 0 | 0 | 6 | 0 | 0 | All_GROUP:sk__Eukaryota(100);sknr0__SAR(100);sknr1__Rhizaria(100);sknr2__Cercozoa(100);f__Cercomonadidae(97); |
| OTU_915 | 3 | 3 | 7 | 2 | 0 | 4 | 4 | 2 | 1 | 0 | 2 | 2 | All_GROUP:sk__Eukaryota(100);sknr0__SAR(100);sknr1__Rhizaria(100);sknr2__Cercozoa(100);f__Cercomonadidae(97); |
| OTU_946 | 1 | 0 | 0 | 0 | 0 | 0 | 2 | 0 | 2 | 0 | 0 | 5 | All_GROUP:sk__Eukaryota(100);sknr0__SAR(100);sknr1__Rhizaria(100);sknr2__Cercozoa(100);f__Cercomonadidae(91); |
| OTU_950 | 3 | 8 | 7 | 0 | 0 | 0 | 1 | 1 | 0 | 0 | 0 | 1 | All_GROUP:sk__Eukaryota(100);sknr0__SAR(100);sknr1__Rhizaria(100);sknr2__Cercozoa(100);f__Cercomonadidae(98); |
| OTU_1095 | 3 | 6 | 4 | 1 | 2 | 2 | 1 | 0 | 0 | 1 | 0 | 0 | All_GROUP:sk__Eukaryota(100);sknr0__SAR(100);sknr1__Rhizaria(100);sknr2__Cercozoa(100);f__Cercomonadidae(99); |
| OTU_73 | 92 | 66 | 49 | 7 | 6 | 7 | 95 | 84 | 137 | 24 | 9 | 31 | All_GROUP:sk__Eukaryota(100);sknr0__SAR(100);sknr1__Rhizaria(100);sknr2__Cercozoa(100);f__Cercomonadidae(100);g__Cercomonas(100);gun0__uncultured_eukaryote(100); |
| OTU_467 | 13 | 3 | 14 | 0 | 0 | 0 | 0 | 0 | 0 | 0 | 0 | 0 | All_GROUP:sk__Eukaryota(100);sknr0__SAR(100);sknr1__Rhizaria(100);sknr2__Cercozoa(100);f__Cercomonadidae(100);g__Cercomonas(90);gun0__uncultured_eukaryote(83); |
| OTU_290 | 10 | 1 | 0 | 0 | 0 | 0 | 9 | 11 | 35 | 8 | 11 | 33 | All_GROUP:sk__Eukaryota(100);sknr0__SAR(100);sknr1__Rhizaria(100);sknr2__Cercozoa(100);f__Cercomonadidae(98);fun0__uncultured_eukaryote(83); |
| OTU_296 | 0 | 0 | 0 | 0 | 0 | 0 | 0 | 43 | 0 | 0 | 0 | 0 | All_GROUP:sk__Eukaryota(100);sknr0__SAR(100);sknr1__Rhizaria(100);sknr2__Cercozoa(100);o__Glissomonadida(100);g__Allantion(100);s__Allapsa_vibrans(100); |
| OTU_820 | 0 | 0 | 0 | 2 | 2 | 3 | 0 | 0 | 0 | 0 | 0 | 0 | All_GROUP:sk__Eukaryota(100);sknr0__SAR(100);sknr1__Rhizaria(100);sknr2__Cercozoa(100);o__Glissomonadida(96);g__Allantion(93);s__Allapsa_vibrans(93); |
| OTU_599 | 26 | 0 | 0 | 4 | 7 | 10 | 2 | 0 | 0 | 0 | 1 | 0 | All_GROUP:sk__Eukaryota(100);sknr0__SAR(100);sknr1__Rhizaria(100);sknr2__Cercozoa(100);o__Glissomonadida(100);g__Proleptomonas(100);s__Proleptomonas_faecicola(100); |
| OTU_249 | 0 | 2 | 0 | 0 | 1 | 0 | 2 | 5 | 0 | 27 | 4 | 32 | All_GROUP:sk__Eukaryota(100);sknr0__SAR(100);sknr1__Rhizaria(100);sknr2__Cercozoa(100);o__Glissomonadida(100);g__Bodomorpha(100);s__uncultured_cercozoan(100); |
| OTU_359 | 63 | 0 | 0 | 0 | 0 | 0 | 0 | 0 | 0 | 0 | 0 | 0 | All_GROUP:sk__Eukaryota(100);sknr0__SAR(100);sknr1__Rhizaria(100);sknr2__Cercozoa(100);o__Glissomonadida(100);g__Bodomorpha(100);s__uncultured_cercozoan(100); |
| OTU_363 | 17 | 35 | 19 | 7 | 6 | 3 | 3 | 0 | 0 | 0 | 0 | 0 | All_GROUP:sk__Eukaryota(100);sknr0__SAR(100);sknr1__Rhizaria(100);sknr2__Cercozoa(100);o__Glissomonadida(100);g__Bodomorpha(100);s__uncultured_cercozoan(100); |
| OTU_793 | 7 | 0 | 0 | 0 | 0 | 0 | 0 | 0 | 0 | 0 | 0 | 0 | All_GROUP:sk__Eukaryota(100);sknr0__SAR(100);sknr1__Rhizaria(100);sknr2__Cercozoa(100);o__Glissomonadida(100);g__Bodomorpha(99);s__uncultured_cercozoan(99); |
| OTU_51 | 295 | 146 | 219 | 151 | 141 | 97 | 237 | 173 | 260 | 161 | 72 | 221 | All_GROUP:sk__Eukaryota(100);sknr0__SAR(100);sknr1__Rhizaria(100);sknr2__Cercozoa(100);o__Glissomonadida(98);g__Heteromita(98);s__uncultured_cercozoan(95); |
| OTU_340 | 5 | 0 | 1 | 1 | 0 | 4 | 20 | 34 | 18 | 0 | 1 | 7 | All_GROUP:sk__Eukaryota(100);sknr0__SAR(100);sknr1__Rhizaria(100);sknr2__Cercozoa(100);o__Glissomonadida(100);g__Heteromita(100);s__uncultured_cercozoan(100); |
| OTU_342 | 9 | 6 | 5 | 2 | 0 | 0 | 9 | 1 | 8 | 29 | 38 | 18 | All_GROUP:sk__Eukaryota(100);sknr0__SAR(100);sknr1__Rhizaria(100);sknr2__Cercozoa(100);o__Glissomonadida(100);g__Heteromita(100);s__uncultured_cercozoan(93); |
| OTU_391 | 27 | 27 | 5 | 2 | 4 | 1 | 0 | 0 | 2 | 0 | 0 | 0 | All_GROUP:sk__Eukaryota(100);sknr0__SAR(100);sknr1__Rhizaria(100);sknr2__Cercozoa(100);o__Glissomonadida(98);g__Heteromita(98);s__uncultured_cercozoan(89); |
| OTU_732 | 3 | 3 | 1 | 3 | 1 | 0 | 18 | 2 | 0 | 1 | 1 | 2 | All_GROUP:sk__Eukaryota(100);sknr0__SAR(100);sknr1__Rhizaria(100);sknr2__Cercozoa(100);o__Glissomonadida(100);g__Heteromita(100);s__uncultured_cercozoan(100); |
| OTU_768 | 0 | 2 | 0 | 6 | 10 | 3 | 2 | 0 | 0 | 0 | 0 | 0 | All_GROUP:sk__Eukaryota(100);sknr0__SAR(100);sknr1__Rhizaria(100);sknr2__Cercozoa(100);o__Glissomonadida(100);g__Heteromita(100);s__uncultured_cercozoan(100); |
| OTU_815 | 0 | 0 | 0 | 0 | 0 | 0 | 0 | 0 | 0 | 7 | 1 | 11 | All_GROUP:sk__Eukaryota(100);sknr0__SAR(100);sknr1__Rhizaria(100);sknr2__Cercozoa(100);o__Glissomonadida(100);g__Heteromita(100);s__uncultured_cercozoan(100); |
| OTU_827 | 5 | 1 | 1 | 0 | 2 | 1 | 19 | 0 | 4 | 1 | 1 | 0 | All_GROUP:sk__Eukaryota(100);sknr0__SAR(100);sknr1__Rhizaria(100);sknr2__Cercozoa(100);o__Glissomonadida(100);g__Heteromita(100);s__uncultured_cercozoan(94); |
| OTU_851 | 20 | 0 | 5 | 0 | 1 | 0 | 0 | 0 | 12 | 1 | 0 | 0 | All_GROUP:sk__Eukaryota(100);sknr0__SAR(100);sknr1__Rhizaria(100);sknr2__Cercozoa(100);o__Glissomonadida(100);g__Heteromita(100);s__uncultured_cercozoan(99); |
| OTU_899 | 2 | 14 | 6 | 0 | 0 | 0 | 0 | 3 | 9 | 0 | 1 | 0 | All_GROUP:sk__Eukaryota(100);sknr0__SAR(100);sknr1__Rhizaria(100);sknr2__Cercozoa(100);o__Glissomonadida(98);g__Heteromita(98);s__uncultured_cercozoan(96); |
| OTU_1029 | 22 | 3 | 3 | 7 | 1 | 3 | 0 | 0 | 0 | 0 | 0 | 0 | All_GROUP:sk__Eukaryota(100);sknr0__SAR(100);sknr1__Rhizaria(100);sknr2__Cercozoa(100);o__Glissomonadida(100);g__Heteromita(100);s__uncultured_cercozoan(93); |
| OTU_1020 | 9 | 0 | 0 | 0 | 0 | 0 | 0 | 1 | 0 | 0 | 0 | 0 | All_GROUP:sk__Eukaryota(100);sknr0__SAR(99);sknr1__Rhizaria(99);sknr2__Cercozoa(99);o__Glissomonadida(98);g__Viridiraptor(85);s__Viridiraptor_invadens(85); |
| OTU_91 | 91 | 91 | 30 | 19 | 12 | 12 | 9 | 0 | 0 | 1 | 0 | 8 | All_GROUP:sk__Eukaryota(100);sknr0__SAR(100);sknr1__Rhizaria(100);sknr2__Cercozoa(100);o__Glissomonadida(100);g__Heteromita(100); |
| OTU_142 | 25 | 8 | 40 | 1 | 0 | 1 | 1 | 14 | 3 | 0 | 1 | 2 | All_GROUP:sk__Eukaryota(100);sknr0__SAR(100);sknr1__Rhizaria(100);sknr2__Cercozoa(100);o__Glissomonadida(96);g__Heteromita(96); |
| OTU_178 | 39 | 7 | 4 | 0 | 2 | 0 | 2 | 0 | 1 | 2 | 0 | 5 | All_GROUP:sk__Eukaryota(100);sknr0__SAR(100);sknr1__Rhizaria(100);sknr2__Cercozoa(100);o__Glissomonadida(94);g__Heteromita(94); |
| OTU_511 | 1 | 1 | 0 | 0 | 1 | 0 | 3 | 23 | 4 | 2 | 0 | 1 | All_GROUP:sk__Eukaryota(100);sknr0__SAR(100);sknr1__Rhizaria(100);sknr2__Cercozoa(100);o__Glissomonadida(89);g__Heteromita(89); |
| OTU_558 | 13 | 5 | 0 | 0 | 1 | 0 | 0 | 0 | 0 | 1 | 1 | 0 | All_GROUP:sk__Eukaryota(100);sknr0__SAR(100);sknr1__Rhizaria(100);sknr2__Cercozoa(100);o__Glissomonadida(99);g__Heteromita(99); |
| OTU_747 | 3 | 3 | 0 | 0 | 0 | 0 | 5 | 10 | 2 | 0 | 0 | 0 | All_GROUP:sk__Eukaryota(100);sknr0__SAR(100);sknr1__Rhizaria(100);sknr2__Cercozoa(100);o__Glissomonadida(90);g__Heteromita(90); |
| OTU_799 | 2 | 1 | 0 | 1 | 5 | 3 | 0 | 0 | 1 | 1 | 0 | 0 | All_GROUP:sk__Eukaryota(100);sknr0__SAR(100);sknr1__Rhizaria(100);sknr2__Cercozoa(100);o__Glissomonadida(97);g__Heteromita(97); |
| OTU_829 | 3 | 3 | 2 | 0 | 0 | 0 | 1 | 0 | 0 | 0 | 0 | 0 | All_GROUP:sk__Eukaryota(100);sknr0__SAR(100);sknr1__Rhizaria(100);sknr2__Cercozoa(100);o__Glissomonadida(100);g__Heteromita(100); |
| OTU_865 | 0 | 2 | 0 | 11 | 5 | 2 | 0 | 0 | 1 | 0 | 0 | 0 | All_GROUP:sk__Eukaryota(100);sknr0__SAR(100);sknr1__Rhizaria(100);sknr2__Cercozoa(100);o__Glissomonadida(91);g__Heteromita(82); |
| OTU_1028 | 7 | 3 | 0 | 3 | 1 | 1 | 0 | 0 | 0 | 0 | 0 | 0 | All_GROUP:sk__Eukaryota(100);sknr0__SAR(100);sknr1__Rhizaria(100);sknr2__Cercozoa(100);o__Glissomonadida(98);g__Heteromita(98); |
| OTU_1030 | 0 | 0 | 0 | 4 | 0 | 0 | 0 | 0 | 0 | 0 | 0 | 5 | All_GROUP:sk__Eukaryota(100);sknr0__SAR(100);sknr1__Rhizaria(100);sknr2__Cercozoa(100);o__Glissomonadida(100);g__Heteromita(100); |
| OTU_257 | 67 | 45 | 32 | 1 | 13 | 6 | 22 | 44 | 64 | 29 | 7 | 12 | All_GROUP:sk__Eukaryota(100);sknr0__SAR(100);sknr1__Rhizaria(100);sknr2__Cercozoa(100);f__Vampyrellidae(100); |
| OTU_536 | 0 | 3 | 41 | 0 | 0 | 0 | 1 | 0 | 0 | 0 | 1 | 0 | All_GROUP:sk__Eukaryota(100);sknr0__SAR(100);sknr1__Rhizaria(100);sknr2__Cercozoa(100);f__Vampyrellidae(100); |
| OTU_902 | 7 | 0 | 0 | 0 | 0 | 0 | 0 | 0 | 0 | 0 | 0 | 0 | All_GROUP:sk__Eukaryota(100);sknr0__SAR(100);sknr1__Rhizaria(100);sknr2__Cercozoa(100);f__Vampyrellidae(87); |
| OTU_992 | 0 | 0 | 0 | 0 | 0 | 0 | 0 | 0 | 16 | 0 | 0 | 0 | All_GROUP:sk__Eukaryota(100);sknr0__SAR(100);sknr1__Rhizaria(100);sknr2__Cercozoa(100);f__Vampyrellidae(100); |
| OTU_309 | 3 | 0 | 17 | 0 | 0 | 0 | 1 | 0 | 5 | 1 | 1 | 4 | All_GROUP:sk__Eukaryota(100);sknr0__SAR(100);sknr1__Rhizaria(100);sknr2__Cercozoa(100);o__Glissomonadida(84); |
| OTU_402 | 18 | 17 | 5 | 5 | 8 | 12 | 18 | 15 | 35 | 6 | 2 | 18 | All_GROUP:sk__Eukaryota(100);sknr0__SAR(100);sknr1__Rhizaria(100);sknr2__Cercozoa(100);o__Glissomonadida(84); |
| OTU_659 | 0 | 6 | 0 | 0 | 0 | 0 | 0 | 0 | 0 | 3 | 0 | 5 | All_GROUP:sk__Eukaryota(100);sknr0__SAR(100);sknr1__Rhizaria(100);sknr2__Cercozoa(100);o__Glissomonadida(96); |
| OTU_710 | 0 | 0 | 0 | 0 | 0 | 0 | 0 | 0 | 0 | 4 | 1 | 33 | All_GROUP:sk__Eukaryota(100);sknr0__SAR(100);sknr1__Rhizaria(100);sknr2__Cercozoa(100);o__Glissomonadida(84); |
| OTU_772 | 1 | 1 | 0 | 0 | 0 | 1 | 10 | 1 | 0 | 0 | 0 | 0 | All_GROUP:sk__Eukaryota(100);sknr0__SAR(99);sknr1__Rhizaria(99);sknr2__Cercozoa(99);o__Glissomonadida(91); |
| OTU_776 | 0 | 0 | 0 | 0 | 0 | 0 | 0 | 0 | 30 | 0 | 0 | 0 | All_GROUP:sk__Eukaryota(100);sknr0__SAR(97);sknr1__Rhizaria(97);sknr2__Cercozoa(97);o__Glissomonadida(97); |
| OTU_879 | 0 | 0 | 0 | 0 | 0 | 0 | 0 | 3 | 0 | 0 | 0 | 0 | All_GROUP:sk__Eukaryota(100);sknr0__SAR(100);sknr1__Rhizaria(100);sknr2__Cercozoa(100);o__Glissomonadida(84); |
| OTU_52 | 12 | 0 | 0 | 0 | 0 | 0 | 322 | 104 | 128 | 0 | 1 | 0 | All_GROUP:sk__Eukaryota(100);sknr0__SAR(98);sknr1__Rhizaria(98);sknr2__Cercozoa(98); |
| OTU_97 | 1 | 6 | 14 | 0 | 0 | 0 | 10 | 8 | 10 | 9 | 15 | 112 | All_GROUP:sk__Eukaryota(100);sknr0__SAR(99);sknr1__Rhizaria(98);sknr2__Cercozoa(98); |
| OTU_127 | 13 | 0 | 11 | 0 | 0 | 0 | 16 | 4 | 29 | 2 | 7 | 22 | All_GROUP:sk__Eukaryota(100);sknr0__SAR(100);sknr1__Rhizaria(100);sknr2__Cercozoa(100); |
| OTU_141 | 49 | 15 | 8 | 1 | 5 | 6 | 11 | 25 | 28 | 17 | 5 | 45 | All_GROUP:sk__Eukaryota(100);sknr0__SAR(100);sknr1__Rhizaria(100);sknr2__Cercozoa(100); |
| OTU_156 | 0 | 2 | 0 | 2 | 1 | 0 | 8 | 5 | 14 | 11 | 0 | 26 | All_GROUP:sk__Eukaryota(100);sknr0__SAR(100);sknr1__Rhizaria(100);sknr2__Cercozoa(100); |
| OTU_202 | 1 | 0 | 0 | 0 | 0 | 0 | 23 | 20 | 6 | 0 | 0 | 0 | All_GROUP:sk__Eukaryota(100);sknr0__SAR(100);sknr1__Rhizaria(100);sknr2__Cercozoa(100); |
| OTU_215 | 50 | 12 | 11 | 3 | 2 | 2 | 15 | 1 | 0 | 15 | 1 | 0 | All_GROUP:sk__Eukaryota(100);sknr0__SAR(100);sknr1__Rhizaria(100);sknr2__Cercozoa(100); |
| OTU_236 | 2 | 1 | 30 | 1 | 0 | 0 | 6 | 13 | 7 | 0 | 0 | 0 | All_GROUP:sk__Eukaryota(100);sknr0__SAR(100);sknr1__Rhizaria(100);sknr2__Cercozoa(100); |
| OTU_254 | 28 | 0 | 0 | 0 | 0 | 0 | 0 | 0 | 0 | 0 | 0 | 0 | All_GROUP:sk__Eukaryota(100);sknr0__SAR(100);sknr1__Rhizaria(100);sknr2__Cercozoa(100); |
| OTU_283 | 0 | 3 | 0 | 0 | 0 | 0 | 3 | 15 | 11 | 0 | 0 | 9 | All_GROUP:sk__Eukaryota(100);sknr0__SAR(100);sknr1__Rhizaria(99);sknr2__Cercozoa(99); |
| OTU_287 | 30 | 46 | 42 | 16 | 10 | 11 | 28 | 13 | 27 | 5 | 5 | 27 | All_GROUP:sk__Eukaryota(100);sknr0__SAR(100);sknr1__Rhizaria(100);sknr2__Cercozoa(100); |
| OTU_288 | 4 | 6 | 21 | 0 | 0 | 1 | 6 | 5 | 11 | 2 | 0 | 3 | All_GROUP:sk__Eukaryota(100);sknr0__SAR(99);sknr1__Rhizaria(99);sknr2__Cercozoa(99); |
| OTU_302 | 12 | 14 | 18 | 3 | 4 | 9 | 8 | 4 | 2 | 6 | 6 | 0 | All_GROUP:sk__Eukaryota(100);sknr0__SAR(100);sknr1__Rhizaria(100);sknr2__Cercozoa(100); |
| OTU_318 | 0 | 0 | 5 | 0 | 1 | 0 | 0 | 0 | 0 | 23 | 16 | 11 | All_GROUP:sk__Eukaryota(100);sknr0__SAR(92);sknr1__Rhizaria(84);sknr2__Cercozoa(84); |
| OTU_323 | 6 | 0 | 0 | 0 | 0 | 0 | 1 | 12 | 10 | 2 | 2 | 5 | All_GROUP:sk__Eukaryota(100);sknr0__SAR(100);sknr1__Rhizaria(100);sknr2__Cercozoa(100); |
| OTU_375 | 0 | 0 | 0 | 0 | 0 | 0 | 0 | 51 | 0 | 0 | 1 | 0 | All_GROUP:sk__Eukaryota(100);sknr0__SAR(98);sknr1__Rhizaria(98);sknr2__Cercozoa(98); |
| OTU_410 | 3 | 0 | 4 | 0 | 0 | 1 | 0 | 0 | 0 | 12 | 2 | 6 | All_GROUP:sk__Eukaryota(100);sknr0__SAR(100);sknr1__Rhizaria(100);sknr2__Cercozoa(100); |
| OTU_457 | 15 | 4 | 1 | 0 | 0 | 0 | 0 | 0 | 0 | 0 | 0 | 0 | All_GROUP:sk__Eukaryota(100);sknr0__SAR(100);sknr1__Rhizaria(100);sknr2__Cercozoa(100); |
| OTU_463 | 11 | 5 | 13 | 0 | 0 | 0 | 0 | 0 | 0 | 2 | 1 | 21 | All_GROUP:sk__Eukaryota(100);sknr0__SAR(100);sknr1__Rhizaria(99);sknr2__Cercozoa(99); |
| OTU_473 | 0 | 0 | 3 | 0 | 0 | 0 | 0 | 0 | 0 | 4 | 6 | 24 | All_GROUP:sk__Eukaryota(100);sknr0__SAR(100);sknr1__Rhizaria(100);sknr2__Cercozoa(100); |
| OTU_506 | 6 | 0 | 0 | 0 | 0 | 0 | 2 | 2 | 15 | 2 | 0 | 8 | All_GROUP:sk__Eukaryota(100);sknr0__SAR(100);sknr1__Rhizaria(100);sknr2__Cercozoa(100); |
| OTU_525 | 0 | 0 | 0 | 1 | 4 | 7 | 0 | 0 | 2 | 5 | 2 | 1 | All_GROUP:sk__Eukaryota(100);sknr0__SAR(100);sknr1__Rhizaria(100);sknr2__Cercozoa(100); |
| OTU_527 | 8 | 4 | 1 | 0 | 0 | 0 | 0 | 2 | 15 | 0 | 0 | 0 | All_GROUP:sk__Eukaryota(100);sknr0__SAR(95);sknr1__Rhizaria(95);sknr2__Cercozoa(95); |
| OTU_531 | 34 | 0 | 0 | 0 | 0 | 0 | 0 | 0 | 0 | 0 | 1 | 2 | All_GROUP:sk__Eukaryota(100);sknr0__SAR(98);sknr1__Rhizaria(98);sknr2__Cercozoa(98); |
| OTU_551 | 20 | 38 | 10 | 1 | 1 | 2 | 0 | 1 | 2 | 2 | 2 | 5 | All_GROUP:sk__Eukaryota(100);sknr0__SAR(100);sknr1__Rhizaria(100);sknr2__Cercozoa(100); |
| OTU_573 | 12 | 8 | 6 | 3 | 0 | 0 | 12 | 6 | 11 | 1 | 0 | 8 | All_GROUP:sk__Eukaryota(100);sknr0__SAR(100);sknr1__Rhizaria(100);sknr2__Cercozoa(100); |
| OTU_609 | 0 | 0 | 0 | 0 | 0 | 0 | 0 | 0 | 0 | 8 | 0 | 0 | All_GROUP:sk__Eukaryota(100);sknr0__SAR(100);sknr1__Rhizaria(99);sknr2__Cercozoa(99); |
| OTU_622 | 1 | 0 | 0 | 4 | 8 | 1 | 0 | 0 | 0 | 0 | 0 | 0 | All_GROUP:sk__Eukaryota(100);sknr0__SAR(100);sknr1__Rhizaria(100);sknr2__Cercozoa(100); |
| OTU_660 | 9 | 2 | 1 | 0 | 0 | 0 | 14 | 0 | 1 | 4 | 1 | 6 | All_GROUP:sk__Eukaryota(100);sknr0__SAR(100);sknr1__Rhizaria(100);sknr2__Cercozoa(100); |
| OTU_726 | 0 | 12 | 0 | 0 | 0 | 0 | 0 | 0 | 0 | 0 | 0 | 0 | All_GROUP:sk__Eukaryota(100);sknr0__SAR(100);sknr1__Rhizaria(100);sknr2__Cercozoa(100); |
| OTU_742 | 0 | 0 | 10 | 0 | 0 | 0 | 0 | 0 | 0 | 0 | 0 | 0 | All_GROUP:sk__Eukaryota(100);sknr0__SAR(100);sknr1__Rhizaria(100);sknr2__Cercozoa(100); |
| OTU_744 | 0 | 0 | 0 | 0 | 0 | 0 | 0 | 0 | 10 | 0 | 0 | 0 | All_GROUP:sk__Eukaryota(100);sknr0__SAR(100);sknr1__Rhizaria(100);sknr2__Cercozoa(100); |
| OTU_754 | 0 | 0 | 0 | 0 | 0 | 0 | 0 | 7 | 0 | 0 | 0 | 0 | All_GROUP:sk__Eukaryota(100);sknr0__SAR(99);sknr1__Rhizaria(96);sknr2__Cercozoa(96); |
| OTU_758 | 0 | 1 | 4 | 0 | 0 | 0 | 0 | 5 | 0 | 1 | 1 | 6 | All_GROUP:sk__Eukaryota(100);sknr0__SAR(100);sknr1__Rhizaria(100);sknr2__Cercozoa(100); |
| OTU_774 | 4 | 4 | 2 | 0 | 1 | 0 | 0 | 0 | 0 | 2 | 7 | 0 | All_GROUP:sk__Eukaryota(100);sknr0__SAR(100);sknr1__Rhizaria(100);sknr2__Cercozoa(100); |
| OTU_775 | 0 | 11 | 0 | 0 | 0 | 0 | 0 | 0 | 0 | 1 | 0 | 0 | All_GROUP:sk__Eukaryota(100);sknr0__SAR(100);sknr1__Rhizaria(100);sknr2__Cercozoa(100); |
| OTU_794 | 7 | 2 | 0 | 0 | 0 | 0 | 0 | 0 | 0 | 0 | 0 | 0 | All_GROUP:sk__Eukaryota(100);sknr0__SAR(100);sknr1__Rhizaria(100);sknr2__Cercozoa(100); |
| OTU_816 | 0 | 1 | 0 | 0 | 0 | 0 | 0 | 0 | 24 | 0 | 0 | 0 | All_GROUP:sk__Eukaryota(100);sknr0__SAR(99);sknr1__Rhizaria(99);sknr2__Cercozoa(99); |
| OTU_819 | 5 | 5 | 18 | 0 | 0 | 1 | 0 | 0 | 5 | 1 | 1 | 8 | All_GROUP:sk__Eukaryota(100);sknr0__SAR(96);sknr1__Rhizaria(95);sknr2__Cercozoa(95); |
| OTU_841 | 4 | 1 | 9 | 0 | 3 | 0 | 2 | 0 | 0 | 3 | 1 | 3 | All_GROUP:sk__Eukaryota(100);sknr0__SAR(99);sknr1__Rhizaria(99);sknr2__Cercozoa(99); |
| OTU_867 | 4 | 4 | 8 | 0 | 0 | 0 | 0 | 0 | 10 | 0 | 0 | 0 | All_GROUP:sk__Eukaryota(100);sknr0__SAR(100);sknr1__Rhizaria(98);sknr2__Cercozoa(98); |
| OTU_869 | 0 | 0 | 11 | 0 | 0 | 0 | 0 | 0 | 0 | 0 | 0 | 0 | All_GROUP:sk__Eukaryota(100);sknr0__SAR(100);sknr1__Rhizaria(100);sknr2__Cercozoa(100); |
| OTU_896 | 3 | 2 | 5 | 1 | 0 | 0 | 0 | 0 | 0 | 0 | 1 | 0 | All_GROUP:sk__Eukaryota(100);sknr0__SAR(100);sknr1__Rhizaria(100);sknr2__Cercozoa(100); |
| OTU_901 | 0 | 0 | 0 | 0 | 0 | 0 | 0 | 0 | 9 | 0 | 0 | 0 | All_GROUP:sk__Eukaryota(100);sknr0__SAR(100);sknr1__Rhizaria(100);sknr2__Cercozoa(100); |
| OTU_905 | 0 | 2 | 0 | 0 | 0 | 0 | 9 | 0 | 0 | 0 | 0 | 0 | All_GROUP:sk__Eukaryota(100);sknr0__SAR(100);sknr1__Rhizaria(100);sknr2__Cercozoa(100); |
| OTU_936 | 0 | 0 | 0 | 0 | 0 | 0 | 7 | 7 | 5 | 1 | 1 | 0 | All_GROUP:sk__Eukaryota(100);sknr0__SAR(100);sknr1__Rhizaria(100);sknr2__Cercozoa(100); |
| OTU_938 | 3 | 0 | 0 | 0 | 0 | 0 | 1 | 0 | 4 | 0 | 1 | 13 | All_GROUP:sk__Eukaryota(100);sknr0__SAR(100);sknr1__Rhizaria(100);sknr2__Cercozoa(100); |
| OTU_955 | 0 | 8 | 1 | 0 | 2 | 0 | 6 | 7 | 1 | 1 | 0 | 0 | All_GROUP:sk__Eukaryota(100);sknr0__SAR(100);sknr1__Rhizaria(100);sknr2__Cercozoa(100); |
| OTU_962 | 0 | 0 | 1 | 0 | 0 | 0 | 0 | 0 | 0 | 0 | 4 | 0 | All_GROUP:sk__Eukaryota(100);sknr0__SAR(100);sknr1__Rhizaria(100);sknr2__Cercozoa(100); |
| OTU_968 | 0 | 0 | 0 | 0 | 0 | 0 | 2 | 0 | 0 | 0 | 0 | 0 | All_GROUP:sk__Eukaryota(100);sknr0__SAR(100);sknr1__Rhizaria(100);sknr2__Cercozoa(100); |
| OTU_987 | 2 | 0 | 4 | 0 | 0 | 0 | 0 | 0 | 0 | 0 | 6 | 5 | All_GROUP:sk__Eukaryota(100);sknr0__SAR(100);sknr1__Rhizaria(100);sknr2__Cercozoa(100); |
| OTU_990 | 7 | 5 | 13 | 1 | 0 | 0 | 0 | 4 | 0 | 1 | 0 | 0 | All_GROUP:sk__Eukaryota(100);sknr0__SAR(100);sknr1__Rhizaria(100);sknr2__Cercozoa(100); |
| OTU_993 | 0 | 0 | 3 | 0 | 0 | 0 | 0 | 0 | 0 | 0 | 0 | 0 | All_GROUP:sk__Eukaryota(100);sknr0__SAR(100);sknr1__Rhizaria(100);sknr2__Cercozoa(100); |
| OTU_998 | 10 | 0 | 0 | 0 | 0 | 1 | 0 | 0 | 0 | 4 | 3 | 0 | All_GROUP:sk__Eukaryota(100);sknr0__SAR(100);sknr1__Rhizaria(100);sknr2__Cercozoa(100); |
| OTU_1005 | 0 | 0 | 20 | 0 | 0 | 0 | 0 | 0 | 0 | 0 | 0 | 0 | All_GROUP:sk__Eukaryota(100);sknr0__SAR(100);sknr1__Rhizaria(100);sknr2__Cercozoa(100); |
| OTU_1018 | 6 | 0 | 0 | 0 | 0 | 0 | 0 | 0 | 0 | 0 | 0 | 0 | All_GROUP:sk__Eukaryota(100);sknr0__SAR(100);sknr1__Rhizaria(99);sknr2__Cercozoa(99); |
| OTU_1031 | 0 | 0 | 0 | 0 | 0 | 0 | 1 | 0 | 1 | 1 | 0 | 2 | All_GROUP:sk__Eukaryota(100);sknr0__SAR(97);sknr1__Rhizaria(96);sknr2__Cercozoa(96); |
| OTU_1040 | 0 | 2 | 3 | 0 | 0 | 0 | 0 | 0 | 0 | 0 | 0 | 0 | All_GROUP:sk__Eukaryota(100);sknr0__SAR(100);sknr1__Rhizaria(100);sknr2__Cercozoa(100); |
| OTU_1058 | 1 | 10 | 3 | 2 | 0 | 0 | 0 | 0 | 2 | 3 | 0 | 0 | All_GROUP:sk__Eukaryota(100);sknr0__SAR(100);sknr1__Rhizaria(100);sknr2__Cercozoa(100); |
| OTU_1083 | 6 | 0 | 0 | 0 | 0 | 0 | 0 | 0 | 0 | 0 | 0 | 0 | All_GROUP:sk__Eukaryota(100);sknr0__SAR(100);sknr1__Rhizaria(100);sknr2__Cercozoa(100); |
| OTU_1089 | 8 | 8 | 4 | 1 | 4 | 1 | 4 | 5 | 2 | 1 | 1 | 4 | All_GROUP:sk__Eukaryota(100);sknr0__SAR(100);sknr1__Rhizaria(100);sknr2__Cercozoa(100); |
| OTU_922 | 8 | 8 | 0 | 0 | 0 | 0 | 0 | 0 | 0 | 4 | 0 | 0 | All_GROUP:sk__Eukaryota(100);sknr0__SAR(100);sknr1__Rhizaria(100);sknr2__Cercozoa(100);o__Glissomonadida(100);onr0__Amb-18S-1124(100);s__Thaumatomonadida_environmental_sample(100); |
| OTU_124 | 6 | 3 | 9 | 0 | 0 | 0 | 41 | 40 | 16 | 0 | 0 | 0 | All_GROUP:sk__Eukaryota(100);sknr0__SAR(100);sknr1__Rhizaria(100);sknr2__Cercozoa(100);o__Glissomonadida(87);onr0__Amb-18S-1124(83);s__uncultured_cercozoan(82); |
| OTU_147 | 8 | 6 | 25 | 1 | 0 | 0 | 6 | 11 | 18 | 0 | 0 | 0 | All_GROUP:sk__Eukaryota(100);sknr0__SAR(100);sknr1__Rhizaria(100);sknr2__Cercozoa(100);o__Glissomonadida(92);onr0__Amb-18S-1124(88);s__uncultured_cercozoan(88); |
| OTU_151 | 0 | 0 | 0 | 0 | 0 | 0 | 0 | 35 | 27 | 4 | 25 | 21 | All_GROUP:sk__Eukaryota(100);sknr0__SAR(100);sknr1__Rhizaria(100);sknr2__Cercozoa(100);o__Glissomonadida(95);onr0__Amb-18S-1124(93);s__uncultured_cercozoan(91); |
| OTU_604 | 0 | 0 | 0 | 2 | 4 | 1 | 0 | 20 | 1 | 1 | 0 | 12 | All_GROUP:sk__Eukaryota(100);sknr0__SAR(100);sknr1__Rhizaria(100);sknr2__Cercozoa(100);o__Glissomonadida(94);onr0__Amb-18S-1124(91);s__uncultured_cercozoan(89); |
| OTU_731 | 0 | 0 | 4 | 0 | 0 | 0 | 0 | 0 | 0 | 0 | 0 | 0 | All_GROUP:sk__Eukaryota(100);sknr0__SAR(100);sknr1__Rhizaria(99);sknr2__Cercozoa(99);o__Glissomonadida(97);onr0__Amb-18S-1124(91);s__uncultured_cercozoan(91); |
| OTU_890 | 33 | 5 | 3 | 5 | 2 | 0 | 0 | 10 | 7 | 4 | 2 | 0 | All_GROUP:sk__Eukaryota(100);sknr0__SAR(100);sknr1__Rhizaria(100);sknr2__Cercozoa(100);o__Glissomonadida(96);onr0__Amb-18S-1124(96);s__uncultured_cercozoan(96); |
| OTU_1002 | 1 | 15 | 1 | 0 | 0 | 0 | 0 | 0 | 0 | 0 | 0 | 0 | All_GROUP:sk__Eukaryota(100);sknr0__SAR(100);sknr1__Rhizaria(100);sknr2__Cercozoa(100);o__Glissomonadida(89);onr0__Amb-18S-1124(80);s__uncultured_cercozoan(80); |
| OTU_1051 | 2 | 2 | 3 | 1 | 0 | 1 | 8 | 1 | 0 | 6 | 1 | 1 | All_GROUP:sk__Eukaryota(100);sknr0__SAR(100);sknr1__Rhizaria(100);sknr2__Cercozoa(100);o__Glissomonadida(100);onr0__Amb-18S-1124(100);s__uncultured_cercozoan(100); |
| OTU_1036 | 0 | 13 | 3 | 0 | 3 | 1 | 0 | 0 | 0 | 0 | 0 | 0 | All_GROUP:sk__Eukaryota(100);sknr0__SAR(100);sknr1__Rhizaria(100);sknr2__Cercozoa(100);sknr3__Imbricatea(100);g__Nudifila(100);s__Nudifila_producta(100); |
| OTU_974 | 0 | 1 | 0 | 0 | 2 | 0 | 11 | 0 | 3 | 0 | 0 | 0 | All_GROUP:sk__Eukaryota(100);sknr0__SAR(100);sknr1__Rhizaria(100);sknr2__Cercozoa(100);sknr3__Imbricatea(100);g__Nudifila(100);s__Thaumatomastigidae_environmental_sample(100); |
| OTU_245 | 4 | 2 | 13 | 4 | 4 | 3 | 0 | 55 | 14 | 9 | 9 | 2 | All_GROUP:sk__Eukaryota(100);sknr0__SAR(100);sknr1__Rhizaria(100);sknr2__Cercozoa(100);sknr3__Imbricatea(85);g__Nudifila(84); |
| OTU_632 | 18 | 2 | 23 | 2 | 2 | 1 | 0 | 3 | 9 | 1 | 0 | 0 | All_GROUP:sk__Eukaryota(100);sknr0__SAR(100);sknr1__Rhizaria(100);sknr2__Cercozoa(100);sknr3__Imbricatea(80); |
| OTU_325 | 5 | 8 | 1 | 2 | 0 | 2 | 19 | 3 | 32 | 7 | 2 | 3 | All_GROUP:sk__Eukaryota(100);sknr0__SAR(100);sknr1__Rhizaria(100);sknr2__Cercozoa(100);sknr3__Imbricatea(100);sknr4__Silicofilosea(100);o__Thaumatomonadida(100);f__Thaumatomonadidae(100);g__Allas(82);s__Allas_diplophysa(82); |
| OTU_248 | 0 | 0 | 2 | 0 | 0 | 0 | 0 | 1 | 0 | 9 | 26 | 48 | All_GROUP:sk__Eukaryota(100);sknr0__SAR(100);sknr1__Rhizaria(100);sknr2__Cercozoa(100);sknr3__Imbricatea(100);sknr4__Silicofilosea(100);o__Euglyphida(100);f__Euglyphidae(100);g__Euglypha(100);s__Euglypha_rotunda(100); |
| OTU_621 | 27 | 54 | 42 | 3 | 2 | 1 | 21 | 8 | 38 | 7 | 3 | 9 | All_GROUP:sk__Eukaryota(100);sknr0__SAR(100);sknr1__Rhizaria(100);sknr2__Cercozoa(100);sknr3__Imbricatea(100);sknr4__Silicofilosea(100);o__Euglyphida(100);f__Euglyphidae(100);g__Euglypha(100);s__Euglypha_rotunda(100); |
| OTU_693 | 0 | 23 | 0 | 0 | 0 | 0 | 0 | 0 | 0 | 0 | 0 | 0 | All_GROUP:sk__Eukaryota(100);sknr0__SAR(100);sknr1__Rhizaria(100);sknr2__Cercozoa(100);sknr3__Imbricatea(100);sknr4__Silicofilosea(100);o__Euglyphida(100);f__Euglyphidae(100);g__Euglypha(100);s__Euglypha_rotunda(100); |
| OTU_1038 | 5 | 6 | 9 | 0 | 0 | 0 | 0 | 7 | 0 | 0 | 3 | 0 | All_GROUP:sk__Eukaryota(100);sknr0__SAR(100);sknr1__Rhizaria(100);sknr2__Cercozoa(100);sknr3__Imbricatea(100);sknr4__Silicofilosea(100);o__Euglyphida(100);f__Euglyphidae(100);g__Euglypha(100);s__Euglypha_rotunda(100); |
| OTU_1041 | 9 | 0 | 0 | 0 | 0 | 0 | 0 | 0 | 0 | 0 | 0 | 0 | All_GROUP:sk__Eukaryota(100);sknr0__SAR(100);sknr1__Rhizaria(100);sknr2__Cercozoa(100);sknr3__Imbricatea(100);sknr4__Silicofilosea(100);o__Euglyphida(100);f__Euglyphidae(100);g__Euglypha(100);s__Euglypha_rotunda(100); |
| OTU_39 | 187 | 89 | 105 | 13 | 8 | 2 | 28 | 19 | 33 | 116 | 51 | 436 | All_GROUP:sk__Eukaryota(100);sknr0__SAR(100);sknr1__Rhizaria(100);sknr2__Cercozoa(100);sknr3__Imbricatea(100);sknr4__Silicofilosea(100);o__Euglyphida(100);f__Euglyphidae(100);g__Euglypha(100); |
| OTU_103 | 30 | 23 | 63 | 3 | 9 | 4 | 7 | 5 | 20 | 24 | 9 | 54 | All_GROUP:sk__Eukaryota(100);sknr0__SAR(100);sknr1__Rhizaria(100);sknr2__Cercozoa(100);sknr3__Imbricatea(99);sknr4__Silicofilosea(99);o__Thaumatomonadida(99);f__Thaumatomonadidae(98); |
| OTU_537 | 0 | 0 | 0 | 0 | 0 | 0 | 0 | 42 | 1 | 0 | 0 | 0 | All_GROUP:sk__Eukaryota(100);sknr0__SAR(100);sknr1__Rhizaria(100);sknr2__Cercozoa(100);sknr3__Imbricatea(99);sknr4__Silicofilosea(99);o__Thaumatomonadida(99);f__Thaumatomonadidae(99); |
| OTU_907 | 0 | 14 | 0 | 0 | 2 | 0 | 0 | 0 | 0 | 2 | 0 | 0 | All_GROUP:sk__Eukaryota(100);sknr0__SAR(100);sknr1__Rhizaria(100);sknr2__Cercozoa(100);sknr3__Imbricatea(97);sknr4__Silicofilosea(97);o__Thaumatomonadida(97);f__Thaumatomonadidae(97); |
| OTU_28 | 2 | 2 | 16 | 0 | 0 | 0 | 371 | 254 | 332 | 1 | 0 | 0 | All_GROUP:sk__Eukaryota(100);sknr0__SAR(100);sknr1__Rhizaria(100);sknr2__Cercozoa(100);sknr3__Imbricatea(100);sknr4__Silicofilosea(100);o__Euglyphida(100);f__Trinematidae(96); |
| OTU_49 | 123 | 153 | 76 | 5 | 3 | 2 | 50 | 69 | 141 | 28 | 14 | 87 | All_GROUP:sk__Eukaryota(100);sknr0__SAR(100);sknr1__Rhizaria(100);sknr2__Cercozoa(100);sknr3__Imbricatea(100);sknr4__Silicofilosea(100);o__Euglyphida(100);f__Trinematidae(98); |
| OTU_145 | 57 | 110 | 53 | 5 | 10 | 4 | 56 | 45 | 19 | 55 | 7 | 38 | All_GROUP:sk__Eukaryota(100);sknr0__SAR(100);sknr1__Rhizaria(100);sknr2__Cercozoa(100);sknr3__Imbricatea(100);sknr4__Silicofilosea(100);o__Euglyphida(100);f__Trinematidae(81); |
| OTU_837 | 15 | 18 | 7 | 0 | 4 | 2 | 1 | 3 | 2 | 0 | 0 | 0 | All_GROUP:sk__Eukaryota(100);sknr0__SAR(100);sknr1__Rhizaria(100);sknr2__Cercozoa(100);sknr3__Imbricatea(100);sknr4__Silicofilosea(100);o__Euglyphida(100);f__Trinematidae(89); |
| OTU_190 | 32 | 11 | 1 | 1 | 4 | 2 | 10 | 15 | 20 | 6 | 0 | 0 | All_GROUP:sk__Eukaryota(100);sknr0__SAR(100);sknr1__Rhizaria(100);sknr2__Cercozoa(100);sknr3__Imbricatea(85);sknr4__Silicofilosea(83);o__Euglyphida(83); |
| OTU_587 | 28 | 0 | 6 | 0 | 0 | 0 | 0 | 0 | 0 | 4 | 0 | 1 | All_GROUP:sk__Eukaryota(100);sknr0__SAR(100);sknr1__Rhizaria(100);sknr2__Cercozoa(100);sknr3__Imbricatea(81);sknr4__Silicofilosea(80);o__Euglyphida(80); |
| OTU_845 | 8 | 0 | 0 | 0 | 0 | 0 | 0 | 0 | 0 | 0 | 0 | 0 | All_GROUP:sk__Eukaryota(100);sknr0__SAR(100);sknr1__Rhizaria(100);sknr2__Cercozoa(100);sknr3__Imbricatea(98);sknr4__Silicofilosea(97);o__Euglyphida(96); |
| OTU_92 | 14 | 2 | 13 | 4 | 3 | 5 | 3 | 0 | 0 | 58 | 24 | 26 | All_GROUP:sk__Eukaryota(100);sknr0__SAR(100);sknr1__Rhizaria(100);sknr2__Cercozoa(100);sknr3__Imbricatea(100);sknr4__Silicofilosea(100);o__Euglyphida(100);onr0__13-1.8(100);s__uncultured_cercozoan(100); |
| OTU_498 | 33 | 0 | 0 | 0 | 0 | 0 | 0 | 0 | 0 | 0 | 0 | 0 | All_GROUP:sk__Eukaryota(100);sknr0__SAR(100);sknr1__Rhizaria(100);sknr2__Cercozoa(100);sknr3__Imbricatea(95);sknr4__Silicofilosea(95);o__Euglyphida(93);onr0__13-1.8(92);s__uncultured_cercozoan(92); |
| OTU_725 | 12 | 4 | 0 | 0 | 0 | 3 | 2 | 1 | 4 | 7 | 0 | 14 | All_GROUP:sk__Eukaryota(100);sknr0__SAR(100);sknr1__Rhizaria(100);sknr2__Cercozoa(100);sknr3__Imbricatea(100);sknr4__Silicofilosea(100);o__Euglyphida(100);onr0__13-1.8(100);s__uncultured_cercozoan(100); |
| OTU_916 | 0 | 0 | 0 | 0 | 4 | 3 | 0 | 0 | 0 | 0 | 0 | 0 | All_GROUP:sk__Eukaryota(100);sknr0__SAR(100);sknr1__Rhizaria(100);sknr2__Cercozoa(100);sknr3__Imbricatea(98);sknr4__Silicofilosea(98);o__Euglyphida(98);onr0__13-1.8(96);s__uncultured_cercozoan(96); |
| OTU_199 | 18 | 36 | 14 | 1 | 4 | 2 | 6 | 30 | 2 | 19 | 3 | 0 | All_GROUP:sk__Eukaryota(100);sknr0__SAR(100);sknr1__Rhizaria(100);sknr2__Cercozoa(100);sknr3__Imbricatea(100);sknr4__Silicofilosea(100);o__Euglyphida(100);f__Trinematidae(100);fnr0__Amb-18S-1480(99);s__uncultured_cercomonad(99); |
| OTU_908 | 0 | 0 | 0 | 5 | 1 | 4 | 0 | 0 | 0 | 14 | 4 | 0 | All_GROUP:sk__Eukaryota(100);sknr0__SAR(100);sknr1__Rhizaria(100);sknr2__Cercozoa(100);sknr3__Imbricatea(100);sknr4__Silicofilosea(100);o__Euglyphida(100);onr0__Incertae_Sedis(100);g__Tracheleuglypha(100);s__Tracheleuglypha_dentata(100); |
| OTU_678 | 0 | 0 | 5 | 0 | 0 | 0 | 0 | 0 | 7 | 0 | 0 | 0 | All_GROUP:sk__Eukaryota(100);sknr0__SAR(100);sknr1__Rhizaria(100);sknr2__Cercozoa(100);sknr3__Imbricatea(100);sknr4__Silicofilosea(100);o__Euglyphida(100);onr0__Incertae_Sedis(100);g__Trachelocorythion(100);s__Trachelocorythion_pulchellum(100); |
| OTU_253 | 8 | 3 | 14 | 3 | 12 | 13 | 41 | 14 | 4 | 48 | 25 | 0 | All_GROUP:sk__Eukaryota(100);sknr0__SAR(100);sknr1__Rhizaria(100);sknr2__Cercozoa(100);sknr3__Imbricatea(99);sknr4__Silicofilosea(99);o__Thaumatomonadida(99);onr0__Peregriniidae(99);g__Gyromitus(99); |
| OTU_540 | 53 | 4 | 13 | 8 | 0 | 0 | 2 | 8 | 8 | 1 | 1 | 0 | All_GROUP:sk__Eukaryota(100);sknr0__SAR(100);sknr1__Rhizaria(100);sknr2__Cercozoa(100);sknr3__Imbricatea(100);sknr4__Spongomonadida(100);g__Spongomonas(100);s__Spongomonas_sp._CCAP_1971/1(96); |
| OTU_959 | 0 | 0 | 0 | 0 | 0 | 0 | 4 | 0 | 0 | 2 | 3 | 10 | All_GROUP:sk__Eukaryota(100);sknr0__SAR(100);sknr1__Rhizaria(100);sknr2__Cercozoa(100);sknr3__Imbricatea(100);sknr4__Spongomonadida(100);g__Spongomonas(100);s__Spongomonas_sp._CCAP_1971/1(80); |
| OTU_868 | 3 | 8 | 1 | 0 | 0 | 0 | 0 | 0 | 1 | 12 | 1 | 2 | All_GROUP:sk__Eukaryota(100);sknr0__SAR(100);sknr1__Rhizaria(100);sknr2__Cercozoa(100);sknr3__Imbricatea(100);sknr4__Spongomonadida(100);g__Spongomonas(100);s__uncultured_cercozoan(100); |
| OTU_603 | 25 | 0 | 2 | 1 | 1 | 1 | 2 | 2 | 0 | 0 | 0 | 11 | All_GROUP:sk__Eukaryota(100);sknr0__SAR(100);sknr1__Rhizaria(100);sknr2__Cercozoa(100);sknr3__Incertae_Sedis(100);g__Gymnophrys(100);s__Athalamea_environmental_sample(100); |
| OTU_682 | 14 | 0 | 1 | 0 | 0 | 0 | 0 | 0 | 0 | 0 | 0 | 6 | All_GROUP:sk__Eukaryota(100);sknr0__SAR(100);sknr1__Rhizaria(100);sknr2__Cercozoa(100);sknr3__Incertae_Sedis(100);g__Gymnophrys(100);s__Athalamea_environmental_sample(88); |
| OTU_183 | 0 | 0 | 16 | 3 | 1 | 1 | 9 | 1 | 2 | 0 | 0 | 0 | All_GROUP:sk__Eukaryota(100);sknr0__SAR(100);sknr1__Rhizaria(100);sknr2__Cercozoa(100);sknr3__Incertae_Sedis(100);g__Gymnophrys(100); |
| OTU_258 | 11 | 4 | 4 | 2 | 5 | 2 | 0 | 0 | 1 | 24 | 5 | 13 | All_GROUP:sk__Eukaryota(100);sknr0__SAR(100);sknr1__Rhizaria(100);sknr2__Cercozoa(100);sknr3__Incertae_Sedis(100);g__Gymnophrys(100); |
| OTU_394 | 6 | 5 | 6 | 0 | 0 | 0 | 3 | 1 | 10 | 4 | 6 | 30 | All_GROUP:sk__Eukaryota(100);sknr0__SAR(100);sknr1__Rhizaria(100);sknr2__Cercozoa(100);sknr3__Incertae_Sedis(100);g__Gymnophrys(100); |
| OTU_453 | 9 | 14 | 2 | 0 | 0 | 0 | 0 | 1 | 0 | 2 | 1 | 5 | All_GROUP:sk__Eukaryota(100);sknr0__SAR(100);sknr1__Rhizaria(100);sknr2__Cercozoa(100);sknr3__Incertae_Sedis(88);g__Gymnophrys(88); |
| OTU_961 | 1 | 3 | 1 | 0 | 0 | 0 | 4 | 0 | 0 | 1 | 0 | 11 | All_GROUP:sk__Eukaryota(100);sknr0__SAR(98);sknr1__Rhizaria(98);sknr2__Cercozoa(98);sknr3__Incertae_Sedis(83);g__Gymnophrys(83); |
| OTU_975 | 0 | 3 | 3 | 0 | 0 | 0 | 1 | 0 | 0 | 0 | 2 | 1 | All_GROUP:sk__Eukaryota(100);sknr0__SAR(100);sknr1__Rhizaria(100);sknr2__Cercozoa(100);sknr3__Incertae_Sedis(100);g__Gymnophrys(100); |
| OTU_134 | 7 | 31 | 33 | 3 | 11 | 1 | 43 | 23 | 25 | 29 | 11 | 30 | All_GROUP:sk__Eukaryota(100);sknr0__SAR(100);sknr1__Rhizaria(100);sknr2__Cercozoa(100);sknr3__Metromonadea(100);g__Metopion(100);gun0__uncultured_eukaryote(100); |
| OTU_798 | 3 | 0 | 2 | 1 | 0 | 0 | 2 | 10 | 1 | 0 | 2 | 6 | All_GROUP:sk__Eukaryota(100);sknr0__SAR(100);sknr1__Rhizaria(100);sknr2__Cercozoa(100);sknr3__Novel_Clade_Gran-3(85);s__uncultured_freshwater_cercozoan(84); |
| OTU_912 | 1 | 2 | 1 | 0 | 0 | 1 | 0 | 18 | 0 | 1 | 4 | 0 | All_GROUP:sk__Eukaryota(100);sknr0__SAR(100);sknr1__Rhizaria(100);sknr2__Cercozoa(100);sknr3__Novel_Clade_Gran-6(100);s__uncultured_cercozoan(100); |
| OTU_1063 | 11 | 4 | 26 | 0 | 0 | 0 | 0 | 0 | 0 | 0 | 0 | 0 | All_GROUP:sk__Eukaryota(100);sknr0__SAR(100);sknr1__Rhizaria(100);sknr2__Cercozoa(100);sknr3__Phytomyxea(100);g__Ligniera(91);s__Ligniera_junci(91); |
| OTU_976 | 0 | 0 | 11 | 0 | 4 | 2 | 0 | 0 | 0 | 0 | 0 | 0 | All_GROUP:sk__Eukaryota(100);sknr0__SAR(100);sknr1__Rhizaria(100);sknr2__Cercozoa(100);sknr3__Phytomyxea(100);g__Polymyxa(92);s__uncultured_plasmodiophorid(87); |
| OTU_973 | 18 | 9 | 0 | 0 | 0 | 0 | 0 | 0 | 0 | 0 | 0 | 0 | All_GROUP:sk__Eukaryota(100);sknr0__SAR(100);sknr1__Rhizaria(100);sknr2__Cercozoa(100);sknr3__Phytomyxea(100);sknr4__uncultured(100); |
| OTU_723 | 8 | 1 | 0 | 1 | 0 | 0 | 0 | 1 | 0 | 0 | 2 | 6 | All_GROUP:sk__Eukaryota(100);sknr0__SAR(100);sknr1__Rhizaria(100);sknr2__Cercozoa(100);sknr3__RM2-SGM58(99);s__Dimorpha-like_sp._ATCC_50522(99); |
| OTU_770 | 0 | 0 | 0 | 8 | 3 | 5 | 0 | 0 | 0 | 3 | 0 | 0 | All_GROUP:sk__Eukaryota(100);sknr0__SAR(100);sknr1__Rhizaria(100);sknr2__Cercozoa(100);sknr3__RT5iin19(100); |
| OTU_516 | 0 | 6 | 16 | 7 | 11 | 10 | 0 | 0 | 0 | 0 | 0 | 0 | All_GROUP:sk__Eukaryota(100);sknr0__SAR(100);sknr1__Rhizaria(100);sknr2__Cercozoa(100);sknr3__RT5iin19(100);sknr4__Coniochaetales_sp._GMG_C4(100); |
| OTU_1055 | 0 | 2 | 0 | 6 | 2 | 0 | 0 | 0 | 0 | 0 | 0 | 0 | All_GROUP:sk__Eukaryota(100);sknr0__SAR(100);sknr1__Rhizaria(100);sknr2__Cercozoa(100);sknr3__RT5iin19(100);skun4__uncultured_eukaryote(94); |
| OTU_66 | 280 | 166 | 115 | 87 | 81 | 123 | 101 | 86 | 88 | 22 | 16 | 46 | All_GROUP:sk__Eukaryota(100);sknr0__SAR(100);sknr1__Rhizaria(100);sknr2__Cercozoa(100);sknr3__Thecofilosea(100); |
| OTU_72 | 61 | 110 | 77 | 24 | 21 | 19 | 45 | 43 | 39 | 56 | 17 | 15 | All_GROUP:sk__Eukaryota(100);sknr0__SAR(100);sknr1__Rhizaria(100);sknr2__Cercozoa(100);sknr3__Thecofilosea(100); |
| OTU_123 | 3 | 6 | 5 | 4 | 3 | 1 | 12 | 47 | 11 | 11 | 4 | 17 | All_GROUP:sk__Eukaryota(100);sknr0__SAR(100);sknr1__Rhizaria(100);sknr2__Cercozoa(100);sknr3__Thecofilosea(100); |
| OTU_139 | 105 | 20 | 14 | 15 | 4 | 4 | 15 | 2 | 21 | 20 | 7 | 64 | All_GROUP:sk__Eukaryota(100);sknr0__SAR(100);sknr1__Rhizaria(100);sknr2__Cercozoa(100);sknr3__Thecofilosea(100); |
| OTU_229 | 1 | 5 | 8 | 15 | 1 | 3 | 5 | 39 | 6 | 4 | 1 | 3 | All_GROUP:sk__Eukaryota(100);sknr0__SAR(100);sknr1__Rhizaria(100);sknr2__Cercozoa(100);sknr3__Thecofilosea(100); |
| OTU_294 | 0 | 0 | 0 | 0 | 0 | 0 | 31 | 13 | 25 | 2 | 0 | 0 | All_GROUP:sk__Eukaryota(100);sknr0__SAR(100);sknr1__Rhizaria(100);sknr2__Cercozoa(100);sknr3__Thecofilosea(99); |
| OTU_376 | 48 | 3 | 0 | 0 | 0 | 0 | 0 | 0 | 0 | 0 | 0 | 0 | All_GROUP:sk__Eukaryota(100);sknr0__SAR(100);sknr1__Rhizaria(100);sknr2__Cercozoa(100);sknr3__Thecofilosea(100); |
| OTU_397 | 9 | 2 | 2 | 1 | 2 | 7 | 1 | 21 | 2 | 5 | 5 | 4 | All_GROUP:sk__Eukaryota(100);sknr0__SAR(100);sknr1__Rhizaria(100);sknr2__Cercozoa(100);sknr3__Thecofilosea(100); |
| OTU_524 | 3 | 9 | 14 | 0 | 3 | 2 | 0 | 0 | 0 | 0 | 0 | 0 | All_GROUP:sk__Eukaryota(100);sknr0__SAR(100);sknr1__Rhizaria(100);sknr2__Cercozoa(100);sknr3__Thecofilosea(100); |
| OTU_655 | 66 | 2 | 13 | 4 | 0 | 1 | 0 | 0 | 0 | 3 | 4 | 0 | All_GROUP:sk__Eukaryota(100);sknr0__SAR(100);sknr1__Rhizaria(100);sknr2__Cercozoa(100);sknr3__Thecofilosea(100); |
| OTU_910 | 2 | 0 | 0 | 0 | 0 | 1 | 0 | 15 | 0 | 0 | 0 | 0 | All_GROUP:sk__Eukaryota(100);sknr0__SAR(100);sknr1__Rhizaria(100);sknr2__Cercozoa(100);sknr3__Thecofilosea(100);o__Cryomonadida(97);onr0__Rhizaspididae(97);g__Rhogostoma(97);gun0__uncultured_eukaryote(97); |
| OTU_982 | 0 | 0 | 0 | 5 | 1 | 4 | 0 | 0 | 0 | 17 | 14 | 23 | All_GROUP:sk__Eukaryota(100);sknr0__SAR(100);sknr1__Rhizaria(100);sknr2__Cercozoa(100);sknr3__Thecofilosea(90);sknr4__uncultured(90);s__uncultured_Oxytrichidae(90); |
| OTU_293 | 21 | 16 | 11 | 0 | 0 | 0 | 0 | 0 | 0 | 2 | 0 | 0 | All_GROUP:sk__Eukaryota(100);sknr0__SAR(100);sknr1__Rhizaria(100);sknr2__Cercozoa(100);sknr3__Thecofilosea(100);sknr4__uncultured(89);skun5__uncultured_eukaryote(89); |
| OTU_431 | 1 | 6 | 4 | 2 | 1 | 0 | 5 | 19 | 22 | 25 | 4 | 6 | All_GROUP:sk__Eukaryota(100);sknr0__SAR(100);sknr1__Rhizaria(100);sknr2__Cercozoa(100);sknr3__Thecofilosea(100);sknr4__uncultured(93);skun5__uncultured_eukaryote(83); |
| OTU_756 | 0 | 8 | 0 | 3 | 0 | 0 | 7 | 7 | 1 | 2 | 0 | 0 | All_GROUP:sk__Eukaryota(100);sknr0__SAR(100);sknr1__Rhizaria(100);sknr2__Cercozoa(100);sknr3__Thecofilosea(100);sknr4__uncultured(95);skun5__uncultured_eukaryote(85); |
| OTU_932 | 0 | 0 | 2 | 0 | 0 | 0 | 0 | 0 | 9 | 0 | 0 | 0 | All_GROUP:sk__Eukaryota(100);sknr0__SAR(100);sknr1__Rhizaria(100);sknr2__Cercozoa(100);sknr3__uncultured(100);s__Cercomonadida_environmental_sample(100); |
| OTU_921 | 0 | 0 | 0 | 0 | 0 | 2 | 0 | 0 | 0 | 0 | 0 | 0 | All_GROUP:sk__Eukaryota(100);sknr0__SAR(100);sknr1__Rhizaria(100);sknr2__Cercozoa(100);sknr3__uncultured(100);s__Cercozoa_sp._ATCC_50378(100); |
| OTU_63 | 47 | 55 | 92 | 0 | 1 | 0 | 19 | 15 | 24 | 11 | 22 | 97 | All_GROUP:sk__Eukaryota(100);sknr0__SAR(100);sknr1__Rhizaria(100);sknr2__Cercozoa(100);sknr3__uncultured(100);s__uncultured_cercozoan(93); |
| OTU_87 | 41 | 41 | 47 | 0 | 8 | 1 | 3 | 4 | 25 | 12 | 8 | 28 | All_GROUP:sk__Eukaryota(100);sknr0__SAR(100);sknr1__Rhizaria(100);sknr2__Cercozoa(100);sknr3__uncultured(100);s__uncultured_Eimeriidae(100); |
| OTU_462 | 0 | 0 | 0 | 2 | 4 | 0 | 3 | 6 | 6 | 0 | 0 | 0 | All_GROUP:sk__Eukaryota(100);sknr0__SAR(100);sknr1__Rhizaria(100);sknr2__Cercozoa(100);f__Vampyrellidae(100);fnr0__uncultured(100);s__uncultured_Rhizaria(95); |
| OTU_712 | 0 | 8 | 9 | 0 | 0 | 0 | 0 | 0 | 0 | 0 | 0 | 0 | All_GROUP:sk__Eukaryota(100);sknr0__SAR(100);sknr1__Rhizaria(100);sknr2__Cercozoa(100);f__Vampyrellidae(100);fnr0__uncultured(100);s__uncultured_Rhizaria(93); |
| OTU_900 | 0 | 0 | 0 | 0 | 0 | 0 | 0 | 2 | 0 | 17 | 11 | 1 | All_GROUP:sk__Eukaryota(100);sknr0__SAR(100);sknr1__Rhizaria(100);sknr2__Cercozoa(100);f__Vampyrellidae(100);fnr0__uncultured(100);s__uncultured_Rhizaria(99); |
| OTU_1042 | 29 | 0 | 0 | 0 | 0 | 0 | 0 | 0 | 0 | 0 | 0 | 0 | All_GROUP:sk__Eukaryota(100);sknr0__SAR(100);sknr1__Rhizaria(100);sknr2__Cercozoa(100);f__Vampyrellidae(100);fnr0__uncultured(100);s__uncultured_Rhizaria(98); |
| OTU_694 | 0 | 0 | 0 | 0 | 1 | 0 | 0 | 0 | 0 | 9 | 12 | 14 | All_GROUP:sk__Eukaryota(100);sknr0__SAR(100);sknr1__Rhizaria(100);sknr2__Cercozoa(100);f__Vampyrellidae(99);fnr0__uncultured(99); |
| OTU_796 | 0 | 4 | 0 | 1 | 0 | 0 | 0 | 0 | 0 | 0 | 0 | 0 | All_GROUP:sk__Eukaryota(100);sknr0__SAR(100);sknr1__Rhizaria(100);sknr2__Cercozoa(100);f__Vampyrellidae(100);fnr0__uncultured(100); |
| OTU_1081 | 16 | 0 | 1 | 0 | 0 | 0 | 0 | 0 | 0 | 0 | 0 | 0 | All_GROUP:sk__Eukaryota(100);sknr0__SAR(100);sknr1__Rhizaria(100);sknr2__Cercozoa(100);f__Vampyrellidae(100);fnr0__uncultured(100); |
| OTU_192 | 11 | 7 | 13 | 1 | 1 | 2 | 8 | 16 | 17 | 42 | 25 | 30 | All_GROUP:sk__Eukaryota(100);sknr0__SAR(100);sknr1__Rhizaria(100);sknr2__Cercozoa(100);sknr3__uncultured(87); |
| OTU_931 | 0 | 0 | 0 | 0 | 0 | 0 | 0 | 6 | 2 | 0 | 0 | 0 | All_GROUP:sk__Eukaryota(100);sknr0__SAR(100);sknr1__Rhizaria(100);sknr2__Cercozoa(100);sknr3__uncultured(88); |
| OTU_1013 | 8 | 7 | 0 | 3 | 1 | 2 | 3 | 0 | 2 | 0 | 0 | 1 | All_GROUP:sk__Eukaryota(100);sknr0__SAR(100);sknr1__Rhizaria(100);sknr2__Cercozoa(100);f__Vampyrellidae(100);fnr0__uncultured(100);fun1__uncultured_eukaryote(100); |
| OTU_180 | 31 | 25 | 17 | 10 | 20 | 18 | 6 | 8 | 27 | 13 | 3 | 6 | All_GROUP:sk__Eukaryota(100);sknr0__SAR(100);sknr1__Rhizaria(100);sknr2__Cercozoa(100);sknr3__uncultured(100);skun4__uncultured_eukaryote(98); |
| OTU_889 | 0 | 0 | 11 | 2 | 0 | 3 | 0 | 10 | 0 | 0 | 0 | 0 | All_GROUP:sk__Eukaryota(100);sknr0__SAR(100);sknr1__Rhizaria(100);sknr2__Cercozoa(100);sknr3__uncultured(100);skun4__uncultured_eukaryote(100); |
| OTU_943 | 5 | 0 | 2 | 0 | 0 | 0 | 0 | 0 | 0 | 0 | 0 | 0 | All_GROUP:sk__Eukaryota(100);sknr0__SAR(100);sknr1__Rhizaria(100);sknr2__Cercozoa(100);sknr3__uncultured(87);skun4__uncultured_eukaryote(86); |
| OTU_460 | 54 | 41 | 24 | 26 | 30 | 23 | 3 | 4 | 16 | 36 | 7 | 24 | All_GROUP:sk__Eukaryota(100);sknr0__SAR(100);sknr1__Rhizaria(100);sknr2__Cercozoa(100);o__Glissomonadida(100);g__Heteromita(100);gun0__uncultured_eukaryote(99); |
| OTU_927 | 0 | 0 | 0 | 0 | 0 | 0 | 0 | 3 | 0 | 0 | 0 | 17 | All_GROUP:sk__Eukaryota(100);sknr0__SAR(100);sknr1__Rhizaria(100);sknr2__Cercozoa(100);o__Glissomonadida(100);g__Heteromita(100);gun0__uncultured_eukaryote(100); |
| OTU_477 | 4 | 6 | 7 | 0 | 3 | 1 | 1 | 0 | 0 | 7 | 2 | 14 | All_GROUP:sk__Eukaryota(100);sknr0__SAR(100);sknr1__Rhizaria(100);sknr2__Cercozoa(100);o__Glissomonadida(89);oun0__uncultured_eukaryote(84); |
| OTU_571 | 8 | 3 | 10 | 5 | 8 | 3 | 16 | 9 | 15 | 3 | 2 | 9 | All_GROUP:sk__Eukaryota(100);sknr0__SAR(100);sknr1__Rhizaria(100);sknr2__Cercozoa(100);o__Glissomonadida(100);oun0__uncultured_eukaryote(96); |
| OTU_840 | 1 | 5 | 3 | 0 | 0 | 0 | 0 | 0 | 2 | 0 | 0 | 0 | All_GROUP:sk__Eukaryota(100);sknr0__SAR(96);sknr1__Stramenopiles(93);sknr2__Ochrophyta(92);p__Diatomea(91); |
| OTU_547 | 0 | 1 | 2 | 6 | 1 | 9 | 0 | 0 | 0 | 0 | 0 | 0 | All_GROUP:sk__Eukaryota(100);sknr0__SAR(100);sknr1__Stramenopiles(100);sknr2__Ochrophyta(100);p__Diatomea(100);pnr0__Bacillariophytina(97);c__Bacillariophyceae(97);g__Luticola(86);s__Luticola_goeppertiana(86); |
| OTU_567 | 0 | 2 | 0 | 7 | 15 | 5 | 13 | 0 | 3 | 0 | 0 | 0 | All_GROUP:sk__Eukaryota(100);sknr0__SAR(100);sknr1__Stramenopiles(100);sknr2__Ochrophyta(100);p__Diatomea(100);pnr0__Bacillariophytina(100);c__Bacillariophyceae(100);g__Stauroneis(98);s__Stauroneis_acuta(98); |
| OTU_957 | 0 | 0 | 5 | 0 | 0 | 0 | 0 | 0 | 0 | 0 | 0 | 0 | All_GROUP:sk__Eukaryota(100);sknr0__SAR(100);sknr1__Stramenopiles(100);sknr2__Ochrophyta(100);p__Diatomea(99);pnr0__Bacillariophytina(93);c__Bacillariophyceae(93);g__Pinnularia(86); |
| OTU_416 | 10 | 21 | 38 | 11 | 21 | 12 | 0 | 0 | 0 | 3 | 1 | 0 | All_GROUP:sk__Eukaryota(100);sknr0__SAR(100);sknr1__Stramenopiles(100);sknr2__Ochrophyta(100);p__Diatomea(100);pnr0__Bacillariophytina(100);c__Bacillariophyceae(100);g__Hantzschia(100);gnr0__Hantzschia_sp._LFS-2014(83); |
| OTU_327 | 12 | 13 | 17 | 11 | 7 | 8 | 7 | 2 | 5 | 0 | 0 | 0 | All_GROUP:sk__Eukaryota(100);sknr0__SAR(100);sknr1__Stramenopiles(100);sknr2__Ochrophyta(100);p__Eustigmatophyceae(100);o__Eustigmatales(100);g__Eustigmatos(100);s__Eustigmatos_vischeri(100); |
| OTU_861 | 2 | 0 | 0 | 3 | 4 | 3 | 0 | 0 | 0 | 0 | 0 | 0 | All_GROUP:sk__Eukaryota(100);sknr0__SAR(100);sknr1__Stramenopiles(100);sknr2__Ochrophyta(100);p__Xanthophyceae(100);o__Tribonematales(100);g__Chlorellidium(100);s__Chlorellidium_tetrabotrys(100); |
| OTU_217 | 0 | 0 | 0 | 2 | 0 | 3 | 0 | 0 | 0 | 74 | 9 | 16 | All_GROUP:sk__Eukaryota(100);sknr0__SAR(100);sknr1__Stramenopiles(100);o__Bicosoecida(100);g__Adriamonas(100);s__Adriamonas_peritocrescens(100); |
| OTU_405 | 6 | 7 | 18 | 0 | 0 | 0 | 2 | 0 | 3 | 0 | 0 | 4 | All_GROUP:sk__Eukaryota(100);sknr0__SAR(100);sknr1__Stramenopiles(100);o__Bicosoecida(100);s__uncultured_bicosoecid(88); |
| OTU_984 | 0 | 0 | 0 | 2 | 11 | 11 | 0 | 0 | 0 | 0 | 0 | 0 | All_GROUP:sk__Eukaryota(100);sknr0__SAR(100);sknr1__Stramenopiles(100);o__Labyrinthulomycetes(99);f__Thraustochytriaceae(99);g__Aplanochytrium(99); |
| OTU_331 | 0 | 0 | 0 | 0 | 0 | 0 | 0 | 0 | 47 | 1 | 0 | 7 | All_GROUP:sk__Eukaryota(100);sknr0__SAR(100);sknr1__Stramenopiles(100);o__Bicosoecida(100); |
| OTU_550 | 0 | 11 | 7 | 2 | 0 | 0 | 1 | 10 | 6 | 1 | 6 | 14 | All_GROUP:sk__Eukaryota(100);sknr0__SAR(100);sknr1__Stramenopiles(100);o__Bicosoecida(93); |
| OTU_652 | 3 | 0 | 0 | 0 | 0 | 0 | 0 | 0 | 0 | 0 | 0 | 0 | All_GROUP:sk__Eukaryota(100);sknr0__SAR(100);sknr1__Stramenopiles(100);o__Bicosoecida(99); |
| OTU_833 | 0 | 0 | 1 | 0 | 0 | 0 | 0 | 0 | 0 | 1 | 3 | 2 | All_GROUP:sk__Eukaryota(100);sknr0__SAR(100);sknr1__Stramenopiles(100);o__Bicosoecida(100); |
| OTU_970 | 3 | 3 | 8 | 0 | 0 | 1 | 4 | 9 | 9 | 1 | 0 | 13 | All_GROUP:sk__Eukaryota(100);sknr0__SAR(100);sknr1__Stramenopiles(100);o__Bicosoecida(100); |
| OTU_1032 | 1 | 13 | 6 | 2 | 2 | 0 | 0 | 5 | 0 | 0 | 0 | 0 | All_GROUP:sk__Eukaryota(100);sknr0__SAR(100);sknr1__Stramenopiles(100);o__Bicosoecida(100); |
| OTU_262 | 24 | 28 | 17 | 3 | 5 | 1 | 1 | 2 | 10 | 0 | 1 | 0 | All_GROUP:sk__Eukaryota(100);sknr0__SAR(97);sknr1__Stramenopiles(97); |
| OTU_313 | 5 | 4 | 4 | 1 | 0 | 1 | 0 | 0 | 0 | 0 | 1 | 0 | All_GROUP:sk__Eukaryota(100);sknr0__SAR(97);sknr1__Stramenopiles(97); |
| OTU_651 | 0 | 0 | 0 | 0 | 0 | 0 | 0 | 3 | 0 | 2 | 4 | 10 | All_GROUP:sk__Eukaryota(100);sknr0__SAR(100);sknr1__Stramenopiles(98); |
| OTU_692 | 0 | 0 | 4 | 0 | 0 | 0 | 1 | 6 | 0 | 0 | 0 | 0 | All_GROUP:sk__Eukaryota(100);sknr0__SAR(95);sknr1__Stramenopiles(94); |
| OTU_874 | 0 | 0 | 4 | 0 | 0 | 0 | 0 | 2 | 8 | 0 | 0 | 0 | All_GROUP:sk__Eukaryota(100);sknr0__SAR(99);sknr1__Stramenopiles(98); |
| OTU_1057 | 6 | 0 | 2 | 0 | 0 | 0 | 0 | 0 | 0 | 0 | 0 | 0 | All_GROUP:sk__Eukaryota(100);sknr0__SAR(99);sknr1__Stramenopiles(97); |
| OTU_653 | 2 | 0 | 0 | 0 | 0 | 0 | 0 | 0 | 0 | 1 | 1 | 1 | All_GROUP:sk__Eukaryota(100);sknr0__SAR(98);sknr1__Stramenopiles(97);o__Labyrinthulomycetes(91);onr0__Amphitremida(91); |
| OTU_629 | 0 | 5 | 0 | 5 | 2 | 7 | 1 | 0 | 7 | 1 | 0 | 6 | All_GROUP:sk__Eukaryota(100);sknr0__SAR(100);sknr1__Stramenopiles(100);c__Hyphochytriomycetes(100);cnr0__Hyphochytriales(100);g__Hyphochytrium(99);gun0__uncultured_eukaryote(99); |
| OTU_419 | 10 | 0 | 1 | 2 | 10 | 6 | 0 | 0 | 0 | 7 | 2 | 4 | All_GROUP:sk__Eukaryota(100);sknr0__SAR(100);sknr1__Stramenopiles(100);c__Hyphochytriomycetes(100);cnr0__Hyphochytriales(100);g__Rhizidiomyces(100);gun0__uncultured_eukaryote(100); |
| OTU_400 | 1 | 1 | 3 | 3 | 1 | 4 | 0 | 6 | 6 | 1 | 1 | 12 | All_GROUP:sk__Eukaryota(100);sknr0__SAR(100);sknr1__Stramenopiles(100);sknr2__MAST-12(100);sknr3__MAST-12C(100);s__uncultured_Eimeriidae(90); |
| OTU_971 | 0 | 0 | 0 | 0 | 0 | 0 | 3 | 0 | 0 | 0 | 0 | 0 | All_GROUP:sk__Eukaryota(100);sknr0__SAR(100);sknr1__Stramenopiles(100);sknr2__MAST-12(100);sknr3__MAST-12C(100);s__uncultured_Eimeriidae(99); |
| OTU_483 | 5 | 3 | 8 | 1 | 0 | 0 | 2 | 0 | 30 | 1 | 0 | 0 | All_GROUP:sk__Eukaryota(100);sknr0__SAR(100);sknr1__Stramenopiles(100);sknr2__MAST-12(100);sknr3__MAST-12C(100); |
| OTU_948 | 1 | 0 | 0 | 0 | 0 | 0 | 25 | 0 | 0 | 0 | 0 | 0 | All_GROUP:sk__Eukaryota(100);sknr0__SAR(100);sknr1__Stramenopiles(100);sknr2__MAST-12(100);sknr3__MAST-12C(100); |
| OTU_1090 | 21 | 3 | 4 | 0 | 0 | 0 | 0 | 0 | 0 | 0 | 0 | 0 | All_GROUP:sk__Eukaryota(100);sknr0__SAR(100);sknr1__Stramenopiles(100);sknr2__MAST-12(100);sknr3__MAST-12C(100); |
| OTU_771 | 1 | 3 | 1 | 2 | 0 | 0 | 0 | 0 | 0 | 0 | 0 | 0 | All_GROUP:sk__Eukaryota(100);sknr0__SAR(100);sknr1__Stramenopiles(100);sknr2__Ochrophyta(100);c__Chrysophyceae(100);o__Ochromonadales(100);g__Paraphysomonas(100);s__Paraphysomonas_sp._10_JMS-2012(97); |
| OTU_115 | 11 | 1 | 9 | 16 | 12 | 11 | 12 | 57 | 14 | 11 | 1 | 8 | All_GROUP:sk__Eukaryota(100);sknr0__SAR(100);sknr1__Stramenopiles(100);sknr2__Ochrophyta(100);c__Chrysophyceae(100);o__Chromulinales(100);g__Poterioochromonas(100);s__Poterioochromonas_malhamensis(80); |
| OTU_561 | 0 | 3 | 2 | 0 | 1 | 7 | 0 | 2 | 0 | 0 | 0 | 0 | All_GROUP:sk__Eukaryota(100);sknr0__SAR(100);sknr1__Stramenopiles(100);sknr2__Ochrophyta(100);c__Chrysophyceae(100);o__Ochromonadales(100);g__Ochromonas(100);s__uncultured_chrysophyte(92); |
| OTU_454 | 5 | 11 | 1 | 0 | 1 | 1 | 0 | 0 | 0 | 1 | 0 | 0 | All_GROUP:sk__Eukaryota(100);sknr0__SAR(100);sknr1__Stramenopiles(100);sknr2__Ochrophyta(100);c__Chrysophyceae(100);o__Ochromonadales(94);g__Ochromonas(93); |
| OTU_589 | 3 | 1 | 0 | 0 | 0 | 0 | 0 | 0 | 10 | 7 | 3 | 9 | All_GROUP:sk__Eukaryota(100);sknr0__SAR(100);sknr1__Stramenopiles(100);sknr2__Ochrophyta(100);c__Chrysophyceae(100);o__Ochromonadales(84);g__Ochromonas(80); |
| OTU_163 | 34 | 5 | 3 | 0 | 0 | 0 | 1 | 0 | 2 | 10 | 1 | 0 | All_GROUP:sk__Eukaryota(100);sknr0__SAR(100);sknr1__Stramenopiles(100);sknr2__Ochrophyta(100);c__Chrysophyceae(100);o__Ochromonadales(98);g__Paraphysomonas(98); |
| OTU_1000 | 3 | 3 | 1 | 1 | 0 | 1 | 1 | 0 | 3 | 2 | 2 | 0 | All_GROUP:sk__Eukaryota(100);sknr0__SAR(100);sknr1__Stramenopiles(100);sknr2__Ochrophyta(100);c__Chrysophyceae(100);o__Ochromonadales(88);g__Paraphysomonas(85); |
| OTU_1052 | 8 | 4 | 20 | 0 | 0 | 1 | 0 | 0 | 0 | 0 | 0 | 0 | All_GROUP:sk__Eukaryota(100);sknr0__SAR(100);sknr1__Stramenopiles(100);sknr2__Ochrophyta(100);c__Chrysophyceae(100);o__Ochromonadales(98);g__Paraphysomonas(98); |
| OTU_48 | 93 | 81 | 64 | 38 | 24 | 15 | 37 | 28 | 42 | 44 | 21 | 15 | All_GROUP:sk__Eukaryota(100);sknr0__SAR(100);sknr1__Stramenopiles(100);sknr2__Ochrophyta(100);c__Chrysophyceae(100);o__Chromulinales(98);g__Spumella(98); |
| OTU_370 | 9 | 13 | 5 | 34 | 24 | 29 | 3 | 6 | 11 | 11 | 2 | 4 | All_GROUP:sk__Eukaryota(100);sknr0__SAR(100);sknr1__Stramenopiles(100);sknr2__Ochrophyta(100);c__Chrysophyceae(100);o__Chromulinales(90);g__Spumella(88); |
| OTU_437 | 7 | 23 | 8 | 5 | 9 | 4 | 0 | 5 | 4 | 25 | 0 | 5 | All_GROUP:sk__Eukaryota(100);sknr0__SAR(100);sknr1__Stramenopiles(100);sknr2__Ochrophyta(100);c__Chrysophyceae(100);o__Chromulinales(99);g__Spumella(99); |
| OTU_150 | 35 | 17 | 86 | 3 | 1 | 2 | 10 | 16 | 29 | 56 | 27 | 123 | All_GROUP:sk__Eukaryota(100);sknr0__SAR(100);sknr1__Stramenopiles(100);sknr2__Ochrophyta(100);c__Chrysophyceae(100);o__Chromulinales(100); |
| OTU_205 | 30 | 18 | 40 | 11 | 8 | 2 | 27 | 2 | 2 | 26 | 6 | 14 | All_GROUP:sk__Eukaryota(100);sknr0__SAR(100);sknr1__Stramenopiles(100);sknr2__Ochrophyta(100);c__Chrysophyceae(100);o__Chromulinales(86); |
| OTU_1075 | 8 | 3 | 7 | 0 | 0 | 0 | 1 | 7 | 0 | 1 | 0 | 1 | All_GROUP:sk__Eukaryota(100);sknr0__SAR(100);sknr1__Stramenopiles(100);sknr2__Ochrophyta(100);c__Chrysophyceae(100);o__Chromulinales(88); |
| OTU_122 | 8 | 46 | 1 | 0 | 0 | 1 | 6 | 44 | 6 | 20 | 7 | 35 | All_GROUP:sk__Eukaryota(100);sknr0__SAR(100);sknr1__Stramenopiles(100);sknr2__Ochrophyta(100);c__Chrysophyceae(100); |
| OTU_452 | 0 | 0 | 0 | 0 | 0 | 0 | 0 | 0 | 0 | 12 | 36 | 6 | All_GROUP:sk__Eukaryota(100);sknr0__SAR(100);sknr1__Stramenopiles(100);sknr2__Ochrophyta(100);c__Chrysophyceae(100); |
| OTU_514 | 1 | 3 | 4 | 4 | 0 | 1 | 7 | 10 | 6 | 1 | 1 | 4 | All_GROUP:sk__Eukaryota(100);sknr0__SAR(100);sknr1__Stramenopiles(100);sknr2__Ochrophyta(100);c__Chrysophyceae(100); |
| OTU_658 | 3 | 10 | 1 | 0 | 0 | 0 | 0 | 0 | 3 | 0 | 1 | 0 | All_GROUP:sk__Eukaryota(100);sknr0__SAR(100);sknr1__Stramenopiles(100);sknr2__Ochrophyta(100);c__Chrysophyceae(100); |
| OTU_960 | 0 | 0 | 0 | 0 | 1 | 0 | 0 | 0 | 0 | 4 | 22 | 4 | All_GROUP:sk__Eukaryota(100);sknr0__SAR(100);sknr1__Stramenopiles(100);sknr2__Ochrophyta(100);c__Chrysophyceae(95); |
| OTU_519 | 0 | 0 | 6 | 0 | 0 | 0 | 4 | 6 | 9 | 0 | 0 | 0 | All_GROUP:sk__Eukaryota(100);sknr0__SAR(100);sknr1__Stramenopiles(100);sknr2__Ochrophyta(100);c__Chrysophyceae(100);cnr0__Incertae_Sedis(84);g__Chlamydomyxa(84);gun0__uncultured_eukaryote(84); |
| OTU_686 | 0 | 4 | 7 | 1 | 1 | 2 | 3 | 0 | 0 | 0 | 2 | 0 | All_GROUP:sk__Eukaryota(100);sknr0__SAR(100);sknr1__Stramenopiles(100);sknr2__Ochrophyta(100);c__Chrysophyceae(100);cnr0__Incertae_Sedis(98);g__Chlamydomyxa(98);gun0__uncultured_eukaryote(98); |
| OTU_45 | 35 | 47 | 26 | 8 | 11 | 9 | 11 | 15 | 38 | 302 | 67 | 80 | All_GROUP:sk__Eukaryota(100);sknr0__SAR(100);sknr1__Stramenopiles(100);sknr2__Ochrophyta(100);c__Chrysophyceae(100);o__Chromulinales(100);onr0__JBNA46(100); |
| OTU_179 | 29 | 35 | 7 | 0 | 1 | 0 | 6 | 7 | 29 | 5 | 3 | 7 | All_GROUP:sk__Eukaryota(100);sknr0__SAR(100);sknr1__Stramenopiles(100);sknr2__Ochrophyta(100);c__Chrysophyceae(100);cnr0__LG21-05(100);s__Oikomonas_sp._SA-2.1(95); |
| OTU_470 | 0 | 2 | 8 | 1 | 0 | 0 | 17 | 10 | 22 | 0 | 2 | 14 | All_GROUP:sk__Eukaryota(100);sknr0__SAR(100);sknr1__Stramenopiles(100);sknr2__Ochrophyta(100);c__Chrysophyceae(100);cnr0__LG21-05(98); |
| OTU_606 | 11 | 2 | 3 | 0 | 0 | 0 | 2 | 1 | 0 | 0 | 0 | 0 | All_GROUP:sk__Eukaryota(100);sknr0__SAR(100);sknr1__Stramenopiles(100);sknr2__Ochrophyta(100);c__Chrysophyceae(100);cnr0__P34.45(100);s__uncultured_chrysophyte(100); |
| OTU_521 | 3 | 9 | 0 | 4 | 1 | 0 | 0 | 0 | 3 | 1 | 0 | 22 | All_GROUP:sk__Eukaryota(100);sknr0__SAR(100);sknr1__Stramenopiles(100);sknr2__Ochrophyta(100);c__Chrysophyceae(100);cnr0__uncultured(100);s__uncultured_chrysophyte(100); |
| OTU_182 | 0 | 29 | 0 | 3 | 0 | 1 | 1 | 77 | 20 | 1 | 1 | 1 | All_GROUP:sk__Eukaryota(100);sknr0__SAR(100);sknr1__Stramenopiles(100);sknr2__Ochrophyta(100);c__Chrysophyceae(100);o__Chromulinales(100);g__Spumella(100);gun0__uncultured_eukaryote(99); |
| OTU_825 | 0 | 0 | 7 | 0 | 4 | 1 | 0 | 0 | 0 | 0 | 0 | 0 | All_GROUP:sk__Eukaryota(100);sknr0__SAR(100);sknr1__Stramenopiles(100);sknr2__Peronosporomycetes(100);g__Aphanomyces(99);s__Aphanomyces_astaci(92); |
| OTU_934 | 0 | 0 | 0 | 1 | 0 | 3 | 0 | 0 | 2 | 0 | 0 | 3 | All_GROUP:sk__Eukaryota(100);sknr0__SAR(100);sknr1__Stramenopiles(100);sknr2__Peronosporomycetes(100);g__Pythium(90); |
| OTU_99 | 10 | 10 | 0 | 15 | 21 | 16 | 36 | 6 | 15 | 4 | 3 | 2 | All_GROUP:sk__Eukaryota(100);sknr0__SAR(100);sknr1__Stramenopiles(100);sknr2__Peronosporomycetes(100); |
| OTU_251 | 1 | 2 | 1 | 13 | 14 | 7 | 0 | 0 | 0 | 0 | 0 | 0 | All_GROUP:sk__Eukaryota(100);sknr0__SAR(100);sknr1__Stramenopiles(100);sknr2__Peronosporomycetes(100); |
| OTU_834 | 7 | 4 | 6 | 10 | 17 | 24 | 5 | 1 | 3 | 1 | 1 | 0 | All_GROUP:sk__Eukaryota(100);sknr0__SAR(100);sknr1__Stramenopiles(100);sknr2__Peronosporomycetes(100); |
| OTU_859 | 0 | 6 | 0 | 5 | 1 | 0 | 0 | 0 | 0 | 0 | 0 | 0 | All_GROUP:sk__Eukaryota(100);sknr0__SAR(100);sknr1__Stramenopiles(100);sknr2__Peronosporomycetes(100); |
| OTU_671 | 0 | 2 | 2 | 1 | 3 | 0 | 0 | 0 | 0 | 2 | 0 | 5 | All_GROUP:sk__Eukaryota(100)unclassified; |
| OTU_353 | 0 | 1 | 2 | 0 | 0 | 0 | 24 | 21 | 21 | 0 | 0 | 1 | All_GROUP:sk__Eukaryota(100)unclassified; |
| OTU_412 | 41 | 18 | 18 | 4 | 7 | 2 | 4 | 1 | 3 | 2 | 0 | 2 | All_GROUP:sk__Eukaryota(100)unclassified; |
| OTU_581 | 11 | 4 | 7 | 3 | 1 | 3 | 0 | 1 | 4 | 1 | 1 | 0 | All_GROUP:sk__Eukaryota(100)unclassified; |
| OTU_449 | 4 | 4 | 2 | 0 | 0 | 0 | 5 | 0 | 0 | 2 | 0 | 11 | All_GROUP:sk__Eukaryota(100)unclassified; |
| OTU_230 | 1 | 1 | 7 | 2 | 2 | 1 | 4 | 0 | 1 | 20 | 11 | 16 | All_GROUP:sk__Eukaryota(100)unclassified; |
| OTU_684 | 0 | 0 | 0 | 0 | 0 | 0 | 1 | 31 | 2 | 0 | 0 | 6 | All_GROUP:sk__Eukaryota(100)unclassified; |
| OTU_903 | 4 | 0 | 4 | 0 | 0 | 0 | 0 | 0 | 0 | 0 | 0 | 0 | All_GROUP:sk__Eukaryota(100)unclassified; |
| OTU_1045 | 0 | 0 | 1 | 0 | 0 | 0 | 0 | 1 | 1 | 3 | 0 | 6 | All_GROUP:sk__Eukaryota(100)unclassified; |
| OTU_546 | 0 | 0 | 0 | 4 | 6 | 4 | 1 | 0 | 0 | 0 | 0 | 0 | All_GROUP:sk__Eukaryota(100)unclassified; |
| OTU_953 | 0 | 2 | 7 | 1 | 0 | 0 | 0 | 0 | 0 | 6 | 2 | 0 | All_GROUP:sk__Eukaryota(100)unclassified; |
| OTU_668 | 10 | 3 | 0 | 1 | 2 | 1 | 2 | 15 | 1 | 1 | 0 | 2 | All_GROUP:sk__Eukaryota(100)unclassified; |
| OTU_526 | 0 | 5 | 15 | 2 | 0 | 0 | 0 | 0 | 0 | 0 | 0 | 0 | All_GROUP:sk__Eukaryota(100)unclassified; |
| OTU_9 | 10 | 32 | 2323 | 0 | 0 | 0 | 0 | 0 | 0 | 0 | 0 | 0 | All_GROUP:sk__Eukaryota(100)unclassified; |
| OTU_20 | 0 | 0 | 0 | 0 | 1 | 0 | 2 | 0 | 0 | 125 | 423 | 1120 | All_GROUP:sk__Eukaryota(100)unclassified; |
| OTU_58 | 0 | 0 | 0 | 55 | 37 | 32 | 76 | 54 | 135 | 0 | 0 | 21 | All_GROUP:sk__Eukaryota(100)unclassified; |
| OTU_75 | 0 | 0 | 120 | 0 | 0 | 0 | 0 | 0 | 0 | 0 | 0 | 0 | All_GROUP:sk__Eukaryota(99)unclassified; |
| OTU_138 | 3 | 2 | 0 | 1 | 1 | 1 | 7 | 5 | 17 | 13 | 3 | 4 | All_GROUP:sk__Eukaryota(100)unclassified; |
| OTU_152 | 17 | 2 | 0 | 1 | 0 | 1 | 34 | 24 | 21 | 37 | 10 | 34 | All_GROUP:sk__Eukaryota(100)unclassified; |
| OTU_157 | 11 | 0 | 1 | 0 | 0 | 1 | 6 | 71 | 1 | 5 | 3 | 1 | All_GROUP:sk__Eukaryota(100)unclassified; |
| OTU_161 | 3 | 1 | 1 | 5 | 2 | 1 | 9 | 0 | 3 | 0 | 2 | 27 | All_GROUP:sk__Eukaryota(100)unclassified; |
| OTU_165 | 70 | 10 | 1 | 3 | 5 | 0 | 0 | 0 | 0 | 0 | 0 | 0 | All_GROUP:sk__Eukaryota(100)unclassified; |
| OTU_169 | 0 | 0 | 0 | 0 | 8 | 0 | 0 | 0 | 63 | 0 | 0 | 0 | All_GROUP:sk__Eukaryota(100)unclassified; |
| OTU_174 | 0 | 6 | 0 | 0 | 0 | 0 | 0 | 0 | 0 | 0 | 0 | 106 | All_GROUP:sk__Eukaryota(99)unclassified; |
| OTU_175 | 2 | 4 | 17 | 0 | 0 | 0 | 6 | 0 | 2 | 3 | 4 | 63 | All_GROUP:sk__Eukaryota(100)unclassified; |
| OTU_203 | 6 | 7 | 0 | 0 | 0 | 0 | 0 | 0 | 0 | 42 | 17 | 4 | All_GROUP:sk__Eukaryota(97)unclassified; |
| OTU_209 | 68 | 1 | 0 | 0 | 0 | 0 | 0 | 0 | 0 | 0 | 0 | 0 | All_GROUP:sk__Eukaryota(100)unclassified; |
| OTU_212 | 0 | 0 | 0 | 0 | 0 | 0 | 32 | 15 | 25 | 0 | 0 | 0 | All_GROUP:sk__Eukaryota(100)unclassified; |
| OTU_219 | 0 | 0 | 0 | 0 | 0 | 0 | 3 | 31 | 0 | 0 | 0 | 0 | All_GROUP:sk__Eukaryota(100)unclassified; |
| OTU_220 | 9 | 0 | 5 | 0 | 0 | 0 | 0 | 0 | 2 | 3 | 9 | 4 | All_GROUP:sk__Eukaryota(100)unclassified; |
| OTU_226 | 2 | 6 | 6 | 0 | 0 | 0 | 4 | 13 | 4 | 19 | 28 | 29 | All_GROUP:sk__Eukaryota(100)unclassified; |
| OTU_237 | 6 | 2 | 7 | 0 | 0 | 0 | 10 | 41 | 5 | 47 | 16 | 14 | All_GROUP:sk__Eukaryota(100)unclassified; |
| OTU_243 | 2 | 2 | 1 | 5 | 16 | 12 | 1 | 0 | 4 | 2 | 1 | 6 | All_GROUP:sk__Eukaryota(100)unclassified; |
| OTU_281 | 3 | 1 | 2 | 0 | 0 | 0 | 6 | 7 | 9 | 12 | 0 | 6 | All_GROUP:sk__Eukaryota(100)unclassified; |
| OTU_284 | 0 | 0 | 0 | 0 | 0 | 0 | 12 | 62 | 5 | 0 | 0 | 0 | All_GROUP:sk__Eukaryota(100)unclassified; |
| OTU_305 | 18 | 30 | 8 | 0 | 6 | 1 | 7 | 11 | 7 | 2 | 12 | 27 | All_GROUP:sk__Eukaryota(99)unclassified; |
| OTU_306 | 16 | 4 | 2 | 2 | 2 | 1 | 4 | 1 | 1 | 19 | 3 | 12 | All_GROUP:sk__Eukaryota(100)unclassified; |
| OTU_307 | 29 | 57 | 20 | 0 | 0 | 0 | 0 | 0 | 0 | 7 | 4 | 8 | All_GROUP:sk__Eukaryota(100)unclassified; |
| OTU_310 | 12 | 2 | 4 | 11 | 2 | 2 | 0 | 4 | 1 | 0 | 0 | 0 | All_GROUP:sk__Eukaryota(100)unclassified; |
| OTU_311 | 103 | 0 | 0 | 0 | 0 | 0 | 0 | 0 | 1 | 0 | 0 | 0 | All_GROUP:sk__Eukaryota(100)unclassified; |
| OTU_317 | 0 | 0 | 0 | 0 | 0 | 0 | 0 | 21 | 3 | 0 | 0 | 0 | All_GROUP:sk__Eukaryota(99)unclassified; |
| OTU_337 | 0 | 1 | 0 | 0 | 0 | 0 | 2 | 0 | 41 | 0 | 0 | 0 | All_GROUP:sk__Eukaryota(100)unclassified; |
| OTU_339 | 21 | 0 | 46 | 0 | 0 | 0 | 10 | 37 | 84 | 3 | 1 | 2 | All_GROUP:sk__Eukaryota(100)unclassified; |
| OTU_345 | 0 | 0 | 0 | 0 | 0 | 0 | 0 | 0 | 0 | 5 | 47 | 59 | All_GROUP:sk__Eukaryota(100)unclassified; |
| OTU_346 | 3 | 1 | 0 | 0 | 0 | 0 | 0 | 9 | 2 | 25 | 6 | 4 | All_GROUP:sk__Eukaryota(100)unclassified; |
| OTU_348 | 2 | 0 | 4 | 0 | 0 | 0 | 4 | 18 | 7 | 18 | 2 | 6 | All_GROUP:sk__Eukaryota(100)unclassified; |
| OTU_349 | 1 | 0 | 50 | 5 | 2 | 12 | 5 | 4 | 0 | 0 | 2 | 6 | All_GROUP:sk__Eukaryota(100)unclassified; |
| OTU_360 | 0 | 0 | 0 | 0 | 0 | 0 | 0 | 0 | 0 | 53 | 4 | 2 | All_GROUP:sk__Eukaryota(100)unclassified; |
| OTU_364 | 5 | 6 | 3 | 3 | 4 | 6 | 11 | 4 | 10 | 10 | 4 | 18 | All_GROUP:sk__Eukaryota(100)unclassified; |
| OTU_366 | 0 | 2 | 10 | 0 | 0 | 0 | 4 | 3 | 0 | 27 | 20 | 3 | All_GROUP:sk__Eukaryota(100)unclassified; |
| OTU_378 | 0 | 5 | 6 | 6 | 6 | 3 | 8 | 0 | 1 | 8 | 4 | 17 | All_GROUP:sk__Eukaryota(100)unclassified; |
| OTU_379 | 30 | 9 | 14 | 0 | 0 | 0 | 3 | 1 | 1 | 0 | 0 | 1 | All_GROUP:sk__Eukaryota(100)unclassified; |
| OTU_383 | 0 | 3 | 0 | 1 | 0 | 0 | 0 | 3 | 33 | 0 | 1 | 0 | All_GROUP:sk__Eukaryota(100)unclassified; |
| OTU_395 | 0 | 0 | 6 | 0 | 0 | 0 | 0 | 0 | 0 | 0 | 0 | 0 | All_GROUP:sk__Eukaryota(100)unclassified; |
| OTU_406 | 5 | 13 | 0 | 6 | 7 | 8 | 1 | 0 | 0 | 3 | 0 | 20 | All_GROUP:sk__Eukaryota(100)unclassified; |
| OTU_408 | 0 | 1 | 24 | 0 | 0 | 0 | 0 | 0 | 0 | 0 | 0 | 0 | All_GROUP:sk__Eukaryota(100)unclassified; |
| OTU_414 | 8 | 1 | 32 | 0 | 0 | 0 | 1 | 10 | 19 | 0 | 0 | 0 | All_GROUP:sk__Eukaryota(100)unclassified; |
| OTU_424 | 11 | 6 | 2 | 6 | 2 | 7 | 9 | 2 | 4 | 0 | 0 | 2 | All_GROUP:sk__Eukaryota(100)unclassified; |
| OTU_433 | 10 | 26 | 40 | 0 | 1 | 0 | 1 | 0 | 0 | 0 | 0 | 0 | All_GROUP:sk__Eukaryota(100)unclassified; |
| OTU_435 | 0 | 0 | 0 | 0 | 0 | 0 | 4 | 5 | 8 | 1 | 1 | 1 | All_GROUP:sk__Eukaryota(100)unclassified; |
| OTU_439 | 25 | 0 | 79 | 0 | 0 | 0 | 0 | 1 | 0 | 0 | 1 | 51 | All_GROUP:sk__Eukaryota(100)unclassified; |
| OTU_441 | 0 | 0 | 0 | 0 | 0 | 0 | 0 | 0 | 0 | 0 | 2 | 21 | All_GROUP:sk__Eukaryota(100)unclassified; |
| OTU_450 | 2 | 8 | 37 | 0 | 0 | 0 | 0 | 0 | 0 | 0 | 0 | 0 | All_GROUP:sk__Eukaryota(100)unclassified; |
| OTU_458 | 7 | 3 | 2 | 9 | 0 | 1 | 0 | 0 | 2 | 0 | 0 | 0 | All_GROUP:sk__Eukaryota(99)unclassified; |
| OTU_464 | 40 | 1 | 0 | 1 | 0 | 0 | 5 | 0 | 19 | 0 | 1 | 3 | All_GROUP:sk__Eukaryota(100)unclassified; |
| OTU_466 | 0 | 0 | 2 | 4 | 1 | 0 | 3 | 1 | 1 | 1 | 1 | 0 | All_GROUP:sk__Eukaryota(100)unclassified; |
| OTU_468 | 3 | 4 | 4 | 5 | 3 | 18 | 4 | 1 | 1 | 2 | 1 | 16 | All_GROUP:sk__Eukaryota(100)unclassified; |
| OTU_472 | 11 | 7 | 6 | 2 | 2 | 3 | 1 | 0 | 0 | 0 | 0 | 0 | All_GROUP:sk__Eukaryota(100)unclassified; |
| OTU_475 | 0 | 0 | 0 | 0 | 0 | 0 | 0 | 0 | 0 | 0 | 0 | 25 | All_GROUP:sk__Eukaryota(100)unclassified; |
| OTU_480 | 2 | 2 | 20 | 4 | 1 | 0 | 3 | 0 | 0 | 8 | 1 | 0 | All_GROUP:sk__Eukaryota(100)unclassified; |
| OTU_485 | 0 | 19 | 4 | 0 | 0 | 1 | 0 | 0 | 0 | 0 | 0 | 0 | All_GROUP:sk__Eukaryota(100)unclassified; |
| OTU_490 | 0 | 10 | 5 | 0 | 0 | 1 | 4 | 0 | 3 | 1 | 1 | 21 | All_GROUP:sk__Eukaryota(100)unclassified; |
| OTU_492 | 2 | 0 | 0 | 0 | 0 | 0 | 19 | 5 | 3 | 0 | 0 | 0 | All_GROUP:sk__Eukaryota(100)unclassified; |
| OTU_494 | 10 | 3 | 2 | 4 | 2 | 1 | 8 | 3 | 9 | 16 | 9 | 10 | All_GROUP:sk__Eukaryota(100)unclassified; |
| OTU_501 | 4 | 3 | 0 | 1 | 0 | 1 | 6 | 6 | 10 | 1 | 0 | 12 | All_GROUP:sk__Eukaryota(100)unclassified; |
| OTU_502 | 0 | 0 | 0 | 1 | 1 | 1 | 0 | 2 | 0 | 11 | 2 | 6 | All_GROUP:sk__Eukaryota(100)unclassified; |
| OTU_504 | 0 | 0 | 0 | 1 | 4 | 1 | 9 | 9 | 0 | 4 | 3 | 25 | All_GROUP:sk__Eukaryota(100)unclassified; |
| OTU_517 | 11 | 0 | 6 | 4 | 0 | 0 | 13 | 1 | 0 | 1 | 0 | 10 | All_GROUP:sk__Eukaryota(100)unclassified; |
| OTU_523 | 19 | 5 | 0 | 7 | 9 | 4 | 0 | 0 | 0 | 0 | 4 | 0 | All_GROUP:sk__Eukaryota(100)unclassified; |
| OTU_535 | 0 | 1 | 0 | 1 | 0 | 1 | 1 | 5 | 10 | 1 | 3 | 4 | All_GROUP:sk__Eukaryota(100)unclassified; |
| OTU_549 | 0 | 0 | 10 | 0 | 0 | 0 | 0 | 0 | 0 | 0 | 0 | 0 | All_GROUP:sk__Eukaryota(100)unclassified; |
| OTU_555 | 0 | 0 | 0 | 0 | 0 | 0 | 10 | 0 | 0 | 0 | 0 | 0 | All_GROUP:sk__Eukaryota(100)unclassified; |
| OTU_563 | 1 | 0 | 30 | 0 | 0 | 0 | 0 | 0 | 0 | 2 | 0 | 0 | All_GROUP:sk__Eukaryota(100)unclassified; |
| OTU_564 | 4 | 8 | 3 | 0 | 0 | 0 | 1 | 13 | 6 | 0 | 4 | 22 | All_GROUP:sk__Eukaryota(100)unclassified; |
| OTU_575 | 4 | 3 | 0 | 3 | 0 | 0 | 1 | 0 | 0 | 1 | 0 | 15 | All_GROUP:sk__Eukaryota(100)unclassified; |
| OTU_577 | 13 | 5 | 3 | 0 | 0 | 1 | 0 | 0 | 0 | 0 | 0 | 0 | All_GROUP:sk__Eukaryota(100)unclassified; |
| OTU_580 | 0 | 0 | 9 | 0 | 0 | 0 | 0 | 8 | 0 | 0 | 0 | 0 | All_GROUP:sk__Eukaryota(100)unclassified; |
| OTU_582 | 0 | 1 | 1 | 1 | 1 | 0 | 1 | 18 | 7 | 3 | 3 | 0 | All_GROUP:sk__Eukaryota(100)unclassified; |
| OTU_585 | 0 | 0 | 0 | 0 | 0 | 0 | 26 | 13 | 22 | 8 | 5 | 2 | All_GROUP:sk__Eukaryota(100)unclassified; |
| OTU_586 | 0 | 0 | 5 | 0 | 0 | 0 | 2 | 0 | 0 | 12 | 1 | 9 | All_GROUP:sk__Eukaryota(99)unclassified; |
| OTU_594 | 8 | 0 | 2 | 0 | 0 | 0 | 6 | 7 | 3 | 56 | 4 | 6 | All_GROUP:sk__Eukaryota(100)unclassified; |
| OTU_597 | 0 | 0 | 0 | 0 | 0 | 0 | 0 | 0 | 0 | 1 | 0 | 4 | All_GROUP:sk__Eukaryota(100)unclassified; |
| OTU_601 | 5 | 9 | 12 | 0 | 0 | 0 | 0 | 4 | 4 | 0 | 0 | 0 | All_GROUP:sk__Eukaryota(100)unclassified; |
| OTU_602 | 0 | 0 | 0 | 0 | 0 | 0 | 0 | 16 | 0 | 0 | 0 | 0 | All_GROUP:sk__Eukaryota(100)unclassified; |
| OTU_610 | 7 | 0 | 0 | 0 | 0 | 0 | 0 | 0 | 0 | 0 | 0 | 0 | All_GROUP:sk__Eukaryota(100)unclassified; |
| OTU_619 | 0 | 0 | 0 | 0 | 0 | 0 | 16 | 3 | 11 | 6 | 4 | 0 | All_GROUP:sk__Eukaryota(100)unclassified; |
| OTU_620 | 0 | 3 | 0 | 0 | 0 | 0 | 0 | 0 | 0 | 0 | 0 | 0 | All_GROUP:sk__Eukaryota(100)unclassified; |
| OTU_638 | 0 | 5 | 0 | 0 | 0 | 0 | 1 | 0 | 0 | 1 | 0 | 0 | All_GROUP:sk__Eukaryota(100)unclassified; |
| OTU_640 | 5 | 1 | 0 | 0 | 0 | 0 | 0 | 0 | 0 | 0 | 0 | 0 | All_GROUP:sk__Eukaryota(100)unclassified; |
| OTU_642 | 5 | 0 | 3 | 0 | 1 | 0 | 0 | 0 | 0 | 0 | 0 | 0 | All_GROUP:sk__Eukaryota(100)unclassified; |
| OTU_647 | 0 | 0 | 0 | 0 | 0 | 0 | 10 | 0 | 6 | 0 | 0 | 0 | All_GROUP:sk__Eukaryota(99)unclassified; |
| OTU_674 | 0 | 0 | 0 | 0 | 1 | 0 | 1 | 1 | 4 | 0 | 4 | 0 | All_GROUP:sk__Eukaryota(100)unclassified; |
| OTU_691 | 3 | 0 | 2 | 1 | 0 | 4 | 6 | 0 | 0 | 0 | 0 | 2 | All_GROUP:sk__Eukaryota(100)unclassified; |
| OTU_700 | 9 | 10 | 32 | 0 | 0 | 0 | 0 | 0 | 0 | 0 | 0 | 3 | All_GROUP:sk__Eukaryota(100)unclassified; |
| OTU_703 | 0 | 0 | 0 | 0 | 0 | 0 | 3 | 8 | 2 | 0 | 0 | 0 | All_GROUP:sk__Eukaryota(100)unclassified; |
| OTU_704 | 1 | 3 | 0 | 0 | 0 | 0 | 8 | 3 | 10 | 0 | 2 | 0 | All_GROUP:sk__Eukaryota(100)unclassified; |
| OTU_708 | 1 | 0 | 0 | 0 | 0 | 0 | 7 | 0 | 0 | 0 | 0 | 0 | All_GROUP:sk__Eukaryota(100)unclassified; |
| OTU_709 | 0 | 0 | 0 | 2 | 0 | 2 | 0 | 0 | 0 | 7 | 0 | 19 | All_GROUP:sk__Eukaryota(100)unclassified; |
| OTU_711 | 0 | 0 | 0 | 0 | 0 | 0 | 1 | 0 | 0 | 0 | 0 | 3 | All_GROUP:sk__Eukaryota(100)unclassified; |
| OTU_716 | 0 | 0 | 0 | 0 | 0 | 0 | 0 | 6 | 0 | 0 | 0 | 0 | All_GROUP:sk__Eukaryota(100)unclassified; |
| OTU_722 | 0 | 0 | 4 | 0 | 0 | 0 | 2 | 10 | 3 | 11 | 9 | 6 | All_GROUP:sk__Eukaryota(100)unclassified; |
| OTU_724 | 0 | 0 | 0 | 0 | 0 | 0 | 0 | 0 | 1 | 0 | 3 | 6 | All_GROUP:sk__Eukaryota(100)unclassified; |
| OTU_727 | 2 | 0 | 1 | 0 | 0 | 0 | 0 | 0 | 0 | 1 | 0 | 2 | All_GROUP:sk__Eukaryota(100)unclassified; |
| OTU_730 | 3 | 0 | 4 | 0 | 0 | 0 | 0 | 0 | 0 | 1 | 0 | 0 | All_GROUP:sk__Eukaryota(100)unclassified; |
| OTU_738 | 7 | 3 | 0 | 1 | 2 | 3 | 11 | 3 | 2 | 3 | 0 | 3 | All_GROUP:sk__Eukaryota(100)unclassified; |
| OTU_748 | 2 | 0 | 2 | 0 | 0 | 0 | 9 | 1 | 4 | 0 | 0 | 0 | All_GROUP:sk__Eukaryota(100)unclassified; |
| OTU_757 | 0 | 0 | 5 | 0 | 0 | 0 | 2 | 3 | 6 | 6 | 1 | 0 | All_GROUP:sk__Eukaryota(100)unclassified; |
| OTU_762 | 0 | 0 | 0 | 0 | 0 | 0 | 0 | 0 | 0 | 0 | 0 | 5 | All_GROUP:sk__Eukaryota(100)unclassified; |
| OTU_767 | 0 | 0 | 0 | 3 | 0 | 0 | 0 | 0 | 0 | 0 | 0 | 0 | All_GROUP:sk__Eukaryota(100)unclassified; |
| OTU_777 | 5 | 0 | 1 | 0 | 0 | 0 | 4 | 5 | 4 | 3 | 1 | 5 | All_GROUP:sk__Eukaryota(100)unclassified; |
| OTU_783 | 1 | 0 | 2 | 0 | 0 | 3 | 2 | 0 | 0 | 1 | 0 | 2 | All_GROUP:sk__Eukaryota(100)unclassified; |
| OTU_784 | 6 | 0 | 17 | 1 | 1 | 4 | 0 | 0 | 0 | 0 | 1 | 0 | All_GROUP:sk__Eukaryota(100)unclassified; |
| OTU_810 | 0 | 0 | 0 | 0 | 0 | 0 | 0 | 1 | 0 | 7 | 3 | 6 | All_GROUP:sk__Eukaryota(100)unclassified; |
| OTU_811 | 0 | 3 | 0 | 0 | 0 | 0 | 3 | 0 | 1 | 3 | 0 | 0 | All_GROUP:sk__Eukaryota(100)unclassified; |
| OTU_813 | 10 | 3 | 7 | 3 | 4 | 13 | 2 | 0 | 0 | 14 | 0 | 0 | All_GROUP:sk__Eukaryota(100)unclassified; |
| OTU_823 | 0 | 0 | 0 | 0 | 0 | 0 | 6 | 1 | 1 | 0 | 1 | 6 | All_GROUP:sk__Eukaryota(100)unclassified; |
| OTU_830 | 0 | 6 | 0 | 2 | 0 | 0 | 0 | 0 | 1 | 0 | 1 | 9 | All_GROUP:sk__Eukaryota(100)unclassified; |
| OTU_838 | 4 | 1 | 2 | 1 | 0 | 0 | 10 | 9 | 21 | 13 | 19 | 0 | All_GROUP:sk__Eukaryota(100)unclassified; |
| OTU_846 | 0 | 0 | 0 | 0 | 0 | 0 | 2 | 0 | 0 | 0 | 0 | 0 | All_GROUP:sk__Eukaryota(100)unclassified; |
| OTU_847 | 12 | 1 | 1 | 0 | 0 | 0 | 0 | 0 | 0 | 1 | 0 | 1 | All_GROUP:sk__Eukaryota(100)unclassified; |
| OTU_855 | 0 | 3 | 0 | 5 | 3 | 1 | 0 | 0 | 0 | 0 | 0 | 0 | All_GROUP:sk__Eukaryota(100)unclassified; |
| OTU_858 | 2 | 3 | 11 | 0 | 0 | 0 | 0 | 1 | 0 | 1 | 1 | 0 | All_GROUP:sk__Eukaryota(100)unclassified; |
| OTU_864 | 0 | 0 | 0 | 0 | 0 | 0 | 0 | 0 | 6 | 0 | 0 | 0 | All_GROUP:sk__Eukaryota(100)unclassified; |
| OTU_872 | 0 | 0 | 0 | 6 | 1 | 1 | 0 | 0 | 0 | 0 | 0 | 0 | All_GROUP:sk__Eukaryota(100)unclassified; |
| OTU_875 | 0 | 0 | 0 | 8 | 1 | 2 | 3 | 0 | 0 | 0 | 0 | 0 | All_GROUP:sk__Eukaryota(100)unclassified; |
| OTU_881 | 1 | 0 | 0 | 0 | 0 | 0 | 23 | 0 | 9 | 0 | 1 | 0 | All_GROUP:sk__Eukaryota(100)unclassified; |
| OTU_884 | 15 | 8 | 1 | 0 | 0 | 0 | 0 | 0 | 0 | 0 | 0 | 0 | All_GROUP:sk__Eukaryota(100)unclassified; |
| OTU_886 | 9 | 0 | 0 | 0 | 2 | 4 | 0 | 0 | 0 | 0 | 0 | 0 | All_GROUP:sk__Eukaryota(100)unclassified; |
| OTU_894 | 0 | 0 | 0 | 0 | 1 | 0 | 0 | 1 | 0 | 11 | 0 | 0 | All_GROUP:sk__Eukaryota(100)unclassified; |
| OTU_895 | 0 | 0 | 0 | 0 | 0 | 0 | 0 | 0 | 0 | 0 | 2 | 0 | All_GROUP:sk__Eukaryota(100)unclassified; |
| OTU_917 | 1 | 1 | 0 | 0 | 0 | 0 | 5 | 0 | 0 | 5 | 1 | 4 | All_GROUP:sk__Eukaryota(100)unclassified; |
| OTU_924 | 0 | 0 | 0 | 0 | 0 | 0 | 12 | 4 | 1 | 0 | 0 | 0 | All_GROUP:sk__Eukaryota(100)unclassified; |
| OTU_928 | 0 | 0 | 0 | 0 | 0 | 0 | 1 | 0 | 1 | 0 | 6 | 15 | All_GROUP:sk__Eukaryota(100)unclassified; |
| OTU_937 | 0 | 3 | 7 | 0 | 0 | 0 | 1 | 7 | 2 | 0 | 0 | 0 | All_GROUP:sk__Eukaryota(100)unclassified; |
| OTU_951 | 0 | 0 | 0 | 0 | 0 | 0 | 1 | 0 | 1 | 2 | 1 | 7 | All_GROUP:sk__Eukaryota(100)unclassified; |
| OTU_954 | 0 | 0 | 0 | 0 | 0 | 0 | 0 | 10 | 4 | 0 | 0 | 0 | All_GROUP:sk__Eukaryota(100)unclassified; |
| OTU_958 | 0 | 3 | 0 | 7 | 17 | 5 | 0 | 0 | 0 | 0 | 0 | 0 | All_GROUP:sk__Eukaryota(99)unclassified; |
| OTU_983 | 0 | 0 | 0 | 0 | 0 | 0 | 0 | 0 | 14 | 4 | 3 | 12 | All_GROUP:sk__Eukaryota(100)unclassified; |
| OTU_986 | 0 | 1 | 16 | 0 | 0 | 0 | 0 | 0 | 0 | 0 | 0 | 0 | All_GROUP:sk__Eukaryota(100)unclassified; |
| OTU_1009 | 2 | 4 | 10 | 0 | 0 | 1 | 1 | 2 | 17 | 5 | 2 | 8 | All_GROUP:sk__Eukaryota(100)unclassified; |
| OTU_1011 | 0 | 11 | 3 | 0 | 0 | 0 | 0 | 0 | 0 | 0 | 0 | 0 | All_GROUP:sk__Eukaryota(100)unclassified; |
| OTU_1033 | 0 | 8 | 0 | 1 | 0 | 0 | 0 | 0 | 0 | 0 | 0 | 0 | All_GROUP:sk__Eukaryota(100)unclassified; |
| OTU_1049 | 0 | 0 | 0 | 0 | 0 | 0 | 4 | 0 | 0 | 0 | 0 | 0 | All_GROUP:sk__Eukaryota(100)unclassified; |
| OTU_1065 | 4 | 0 | 0 | 0 | 0 | 0 | 0 | 0 | 2 | 0 | 8 | 3 | All_GROUP:sk__Eukaryota(100)unclassified; |
| OTU_1067 | 8 | 0 | 0 | 0 | 1 | 0 | 0 | 0 | 0 | 0 | 0 | 0 | All_GROUP:sk__Eukaryota(100)unclassified; |
| OTU_1070 | 0 | 0 | 17 | 0 | 0 | 1 | 0 | 0 | 0 | 0 | 0 | 0 | All_GROUP:sk__Eukaryota(100)unclassified; |
| OTU_1071 | 0 | 0 | 0 | 0 | 0 | 0 | 0 | 0 | 8 | 0 | 0 | 0 | All_GROUP:sk__Eukaryota(100)unclassified; |
| OTU_1073 | 5 | 3 | 0 | 0 | 0 | 2 | 0 | 0 | 0 | 0 | 0 | 2 | All_GROUP:sk__Eukaryota(100)unclassified; |
| OTU_1074 | 5 | 0 | 0 | 0 | 0 | 0 | 0 | 0 | 0 | 0 | 4 | 0 | All_GROUP:sk__Eukaryota(100)unclassified; |
| OTU_1076 | 16 | 0 | 0 | 0 | 0 | 0 | 0 | 0 | 0 | 0 | 0 | 0 | All_GROUP:sk__Eukaryota(99)unclassified; |
| OTU_1078 | 0 | 0 | 0 | 2 | 0 | 0 | 3 | 0 | 0 | 0 | 0 | 0 | All_GROUP:sk__Eukaryota(100)unclassified; |
| OTU_1082 | 0 | 0 | 5 | 0 | 3 | 0 | 0 | 0 | 0 | 0 | 0 | 0 | All_GROUP:sk__Eukaryota(100)unclassified; |
| OTU_1086 | 14 | 0 | 0 | 0 | 0 | 0 | 0 | 0 | 0 | 0 | 0 | 0 | All_GROUP:sk__Eukaryota(100)unclassified; |
| OTU_1091 | 0 | 1 | 0 | 0 | 0 | 0 | 0 | 0 | 5 | 0 | 1 | 0 | All_GROUP:sk__Eukaryota(100)unclassified; |
