# Supplementary figures and images for: Drivers and assemblies of soil eukaryotic microbes among different soil habitat types in a semi-arid mountain in China
Source: PeerJ. 2018 Dec 5;6:e6042. doi: 10.7717/peerj.6042 (PMC6286657; doi:10.7717/peerj.6042)

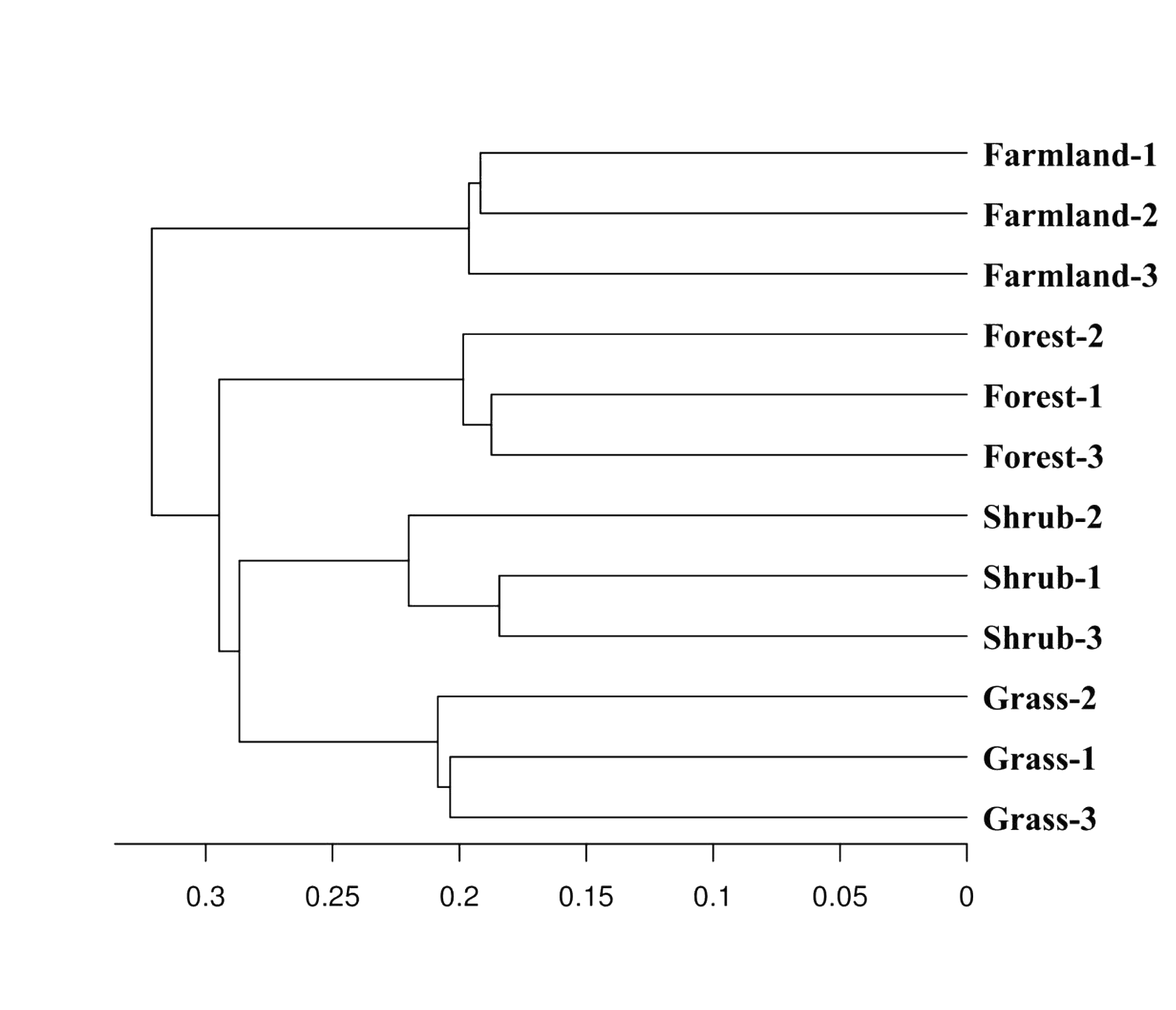

Supplement: Supplemental Information 6 [file peerj-06-6042-s006.png]
